# The effect of stir‐frying on the aging of oat flour during storage: A study based on lipidomics

**DOI:** 10.1002/fsn3.3985

**Published:** 2024-02-20

**Authors:** Yuanyuan Zhang, Minjun Sun, Rui Huo, Qixin Gu, Ying Miao, Meili Zhang

**Affiliations:** ^1^ College of Food Science and Engineering Inner Mongolia Agriculture University Hohhot China

**Keywords:** ether lipid metabolism, lipidomics, oat flour, sphingolipid metabolism, stir‐frying

## Abstract

In this study, we used the LC‐ESI‐MS/MS technique to elucidate the effects of stir‐frying on the lipidomics of oat flour before and after storage. We detected 1540 lipids in 54 subclasses; triglycerides were the most abundant, followed by diacylglycerol, ceramide (Cer), digalactosyldiacylglycerol, cardiolipin, and phosphatidylcholine. Principal component analysis and orthogonal least squares discriminant analysis analyses showed that oat flour lipids were significantly different before and after storage in stir‐fried oat flour and raw oat flour. After oat flour was stir‐fried, most of the lipid metabolites in it were significantly downregulated, and the changes in lipids during storage were reduced. Sphingolipid metabolism and ether lipid metabolism were the key metabolic pathways, and Cer, PC, and lyso‐phosphatidylcholine were the key lipid metabolites identified in the related metabolic pathways during oat flour storage. Frying inhibits lipid metabolic pathways during storage of oat flour, thereby improving lipid stability and quality during storage. This study laid the foundation for further investigating quality control and the mechanism of changes in lipids during the storage of oat flour.

## Graphical abstract

In this study, we used the LC‐ESI‐MS/MS technique to elucidate the effects of stir frying on the lipidomics of oat flour before and after storage. Frying inhibits lipid metabolic pathways during the storage of oat flour, thereby improving lipid stability and quality during storage.

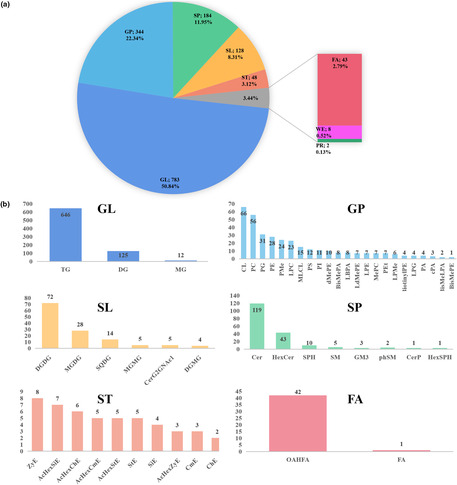

## 1 INTRODUCTION

Oats are nutrient‐rich, healthy, and contain proteins, unsaturated fatty acids, and polyphenols (Yang et al., 2021). These factors make oats attractive for developing new food items. Oats are popular because of their health‐promoting properties. New methods have been implemented to increase the use of oats beyond traditional products, such as oat β‐glucan preparations (Gudej et al., 2021), functional oat beverages (Márquez‐Villacorta et al., 2021), and modified oat biscuits (Ruthann & Jennifer, 2007). For producing oat flour, oat grains are usually stir‐fried. Stir‐frying is performed before oats are ground, which increases the milling rate, enhances the flavor and processing performance, and prolongs the shelf life. A number of problems can occur when oats are used as a food ingredient, one of the main problems being the creation of undesirable lipid odors. High lipid content (about 5%–8%), especially unsaturated fatty acids, can lead to lipid deterioration in oats (Leonova et al., 2008; Tosh & Bordenave, 2020). Oats are rich in nutrients because they have high levels of unsaturated fatty acids, but they are also highly prone to rancidity. Additionally, lipid‐modifying enzymes (e.g., peroxidase) in oat flour are closely related to lipid degradation (Decker et al., 2014; Yang et al., 2017).

Rancidity occurs generally because of lipid deterioration, although protein deterioration and reactions with phenolic acids are also responsible. During storage, oat lipids undergo two types of reactions, which include hydrolytic deterioration of triacylglycerols or phospholipids and oxidative deterioration of polyunsaturated fatty acids (Heiniö et al., 2002). As oxidation proceeds, flavor development significantly compromises the sensory quality of food until consumers reject it. Lipid stability is essential for maintaining the quality of food and its products; lipid oxidation mainly shortens the shelf life of food (Sun et al., 2020). Traditional heat treatment (Wang, Cui, et al., 2021), infrared radiation (Wang et al., 2017), and ozone treatment (Naito, 2009) before the storage of many cereals can inactivate lipase and prevent the invasion of mold, thus improving storage quality.

Stir‐frying, which is similar to baking, is a traditional Chinese heat treatment and is one of the most important ways of thermal processing treatments for oats. Based on rheological studies, Qian et al. (2020) found that stir‐fried oat flour formed a more stable network structure, and the oat flour paste had higher elasticity compared to not stir‐fried oat flour. Zhang et al. (2021) successfully developed a new modification method for oat flour by investigating the effects of differential pressure explosion puffing parameters (heating time, expansion temperature, and differential pressure) on the degree of pasting, thermodynamic properties, and crystal structure of oat flour. Moreover, Jokinen et al. (2021) predicted the functional properties of industrially produced (heat‐treated) oat flour on an industrial scale based on near‐infrared transmission using the properties of natural oat particles. Previous studies on oat frying treatments have focused on the influence of heat treatment and the functional properties of oat flour. However, they did not explain why stir‐frying should be performed, nor did they clarify the effects of stir‐frying on the storage quality of oat flour from the perspective of changes in lipid composition.

Lipidomics is widely used in molecular physiology, nutrition, and environmental health. The changes in lipid composition during the processing and storage of food can be investigated at the level of lipid molecules (Napolitano et al., 2018; Vigor et al., 2022; Xie et al., 2019). Therefore, in this study, we used stir‐fried oat flour as the raw material and raw oat flour as a control to determine the effects of stir‐frying on the main lipid components of oat flour and its mechanism of action based on changes in the lipids before and after oat flour storage. Our study might provide a theoretical basis for further improving the processing and storage of oats.

## 2 EXPERIMENTALSECTION

### 2.1 Materials and reagents

Oat grain was obtained from Inner Mongolia Xibei Huitong Agricultural Science and Technology Development Co., Ltd. Chromatographically pure methanol, acetonitrile, formic acid, and isopropanol were purchased from Fisher. Chromatographically pure ammonium acetate was purchased from Sigma‐Aldrich, and methyl tert‐butyl ether (MTBE) was purchased from Adamas‐beta.

### 2.2 Instruments

Ultra‐high performance liquid chromatography‐tandem Fourier transform mass spectrometry UHPLC‐Q Exactive HF‐X system (Thermo Fisher Scientific); Vanquish Horizon system UHPLC liquid chromatography system (Thermo Scientific); Wonbio‐96c multi‐sample freezing grinder (Shanghai Wonbio Biotech Co., Ltd.); HORIBA HR Evolution Raman Spectrometer (Shanghai Sunnao Technology Co., Ltd.); LNG‐T88 table‐top rapid centrifugal concentration dryer (Taicang Huamei Biochemical Instrument Factory); and Centge 5430R high‐speed refrigerated centrifuge (Eppendorf, Germany).

### 2.3 Experimental methods

#### 2.3.1 Preparation and storage of sample

Stir‐fried oat flour and raw oat flour were prepared as described by Zhang et al. (2023). High‐temperature frying of oat seeds (temperature 250–280°C, time 50s ± 5 s, in‐line processing), repeating the crushing and sieving (80 mesh) steps 4 times, was collected to stir‐frying oat flour. Raw oat seeds were crushed in a pulverizer and sieved through an 80‐mesh sieve. The prepared samples were put in PE‐sealed pockets (500 g per bag) and stored at room temperature (25°C) for 9 months in the dark. Then, the samples were kept at −80°C until testing. Fresh samples of stir‐fried and raw oat flour were used as controls. The four samples were C0 (fresh stir‐fried oat flour), S0 (fresh raw oat flour), C9 (stir‐fried oat flour stored for 9 months), and S9 (raw oat flour stored for 9 months).

#### 2.3.2 Oat lipid extraction

First, 50 mg of the sample was weighed and placed in a 2 mL centrifuge tube with a bead. Then, 280 μL of extract (methanol: water = 2:5) and 400 μL of MTBE were added. After grinding the frozen samples for 6 min, cryogenic ultrasonic extraction was performed for 30 min (5°C, 40 KHz), and the samples were left undisturbed at −20°C for 30 min. The samples were centrifuged for 15 min (13,000 g, 4°C), and then, 350 μL of the supernatant was transferred into an EP tube and blown dry with nitrogen. The samples were reconstituted by adding 100 μL of the extract (isopropanol: acetonitrile = 1:1), vortexed for 30 s, and sonicated at a low temperature for 5 min (5°C, 40 KHz). Then, the samples were centrifuged for 10 min (13,000 g, 4°C), and the supernatant was transferred to an injection vial with an inner cannula for analysis. Additionally, 20 μL of each sample was pipetted and mixed as a quality control sample (QC) to assess the repeatability throughout the analysis. Six replicates were prepared per sample.

#### 2.3.3 Determination of the lipid structure of oats

The structure of 4 groups of oat lipids was determined by a LabRAM HR evolution Raman spectrometer using a 532 nm laser, a 10 × objective lens, a scanning range of 100–3200 cm^−1^, a scanning time of 10 s, and a cumulative number of 3 times.

#### 2.3.4 Analytical chromatographic and mass spectrometric conditions for LC–MS/MS

The samples (2 μL) were separated using an Accucore C30 column (100 mm × 2.1 mm i.d., 2.6 μm; Thermo) and analyzed via mass spectrometry. Mobile phase A consisted of 50% acetonitrile in water containing 0.1% formic acid and 10 mmol/L ammonium acetate, and mobile phase B consisted of acetonitrile/isopropanol/water (10/88/2) containing 0.02% formic acid and 2 mmol/L ammonium acetate. The flow rate was set to 0.4 mL/min, and the column temperature was maintained at 40°C. The separation gradient was as follows: 0–4 min, 35%–60%B; 4–12 min, 60%–85%B; 12–21 min, 85%–100%B; 21–24 min, 100%B; 24–24.1 min, 100%–35%B; 24.1–28 min, 35%B for equilibrating the systems.

The positive and negative ion scanning modes were used for acquiring the mass spectrum signal of the samples; the mass scanning range was 200–2000 m/z. Other conditions included ion spray voltage and positive ion voltage of 3000 V, negative ion voltage of 3000 V, sheath gas of 60 psi, auxiliary heating gas of 20 psi, ion source heating temperature of 370°C, and cycle collision energy of 20–60 V.

#### 2.3.5 Statistical analysis

After the samples were analyzed, the LC–MS raw data were imported into the lipidomics processing software LipidSearch (Thermo, CA) for baseline filtering, peak identification, integration, retention time correction, peak alignment, and identification. The preprocessed data were uploaded to the Majorbio cloud platform (https://cloud.majorbio.com) for principal component analysis (PCA) and orthogonal least squares discriminant analysis (OPLS‐DA). Seven cross‐validations were performed to assess the stability of the model. A Student's *t*‐test and fold difference analysis were performed. Significant differential metabolites were selected based on variable weight values (VIP) obtained from the OPLS‐DA model and the *p*‐values obtained from Student's *t*‐tests. The pathways involved in differential substances were obtained from the metabolic pathway annotations in the KEGG database.

## 3 RESULTS AND DISCUSSION

### 3.1 Oat flour lipid profile

Each molecular structure or group in oils and fats has a corresponding characteristic peak in Raman spectroscopy, and the changes in the stretching vibration region of the C=C double bond can be measured by Raman spectroscopy to evaluate the degree of lipid oxidation. The Raman spectra of oat flour fat were concentrated in the regions of wave numbers 800–1800 cm^−1^ and 2800–3050 cm^−1^, which is consistent with the results of the previous study (Carmona et al., 2014). The Raman spectra of the oils and fats of raw oat flour during storage were basically the same; the only difference was that S0 did not have a peak at 1008 cm^−1^, while the ones after storage had a small peak here. With the extension of the storage period, the peaks of C0 at 3008 cm^−1^ did not appear, and the peaks of C9 and C12 at 1158 cm^−1^ and 1524 cm^−1^ disappeared after storage (Figure S1). This indicates that lipid oxidation reaction occurs in oats after frying treatment; the C=C group is reduced, and unsaturated fatty acids are converted to saturated fatty acids, which is consistent with the fatty acid composition results of our previous study (Zhang et al., 2023). In addition, in our previous study on the flavor of oat flour, it was found that the quality of fried oat flour gradually deteriorated after 9 months. Therefore, samples stored for 9 months were selected for lipidomics studies.

Under the LC‐ESI‐MS/MS analytical conditions, the total ion chromatograms of the QC samples showed uniform peak distribution with consistent retention time and peak intensity (Figure S2). This indicates that the mass spectra were stable even when the same substance was detected at different times. Therefore, the lipidomics method used in this study is highly reproducible and reliable.

**FIGURE 1:**
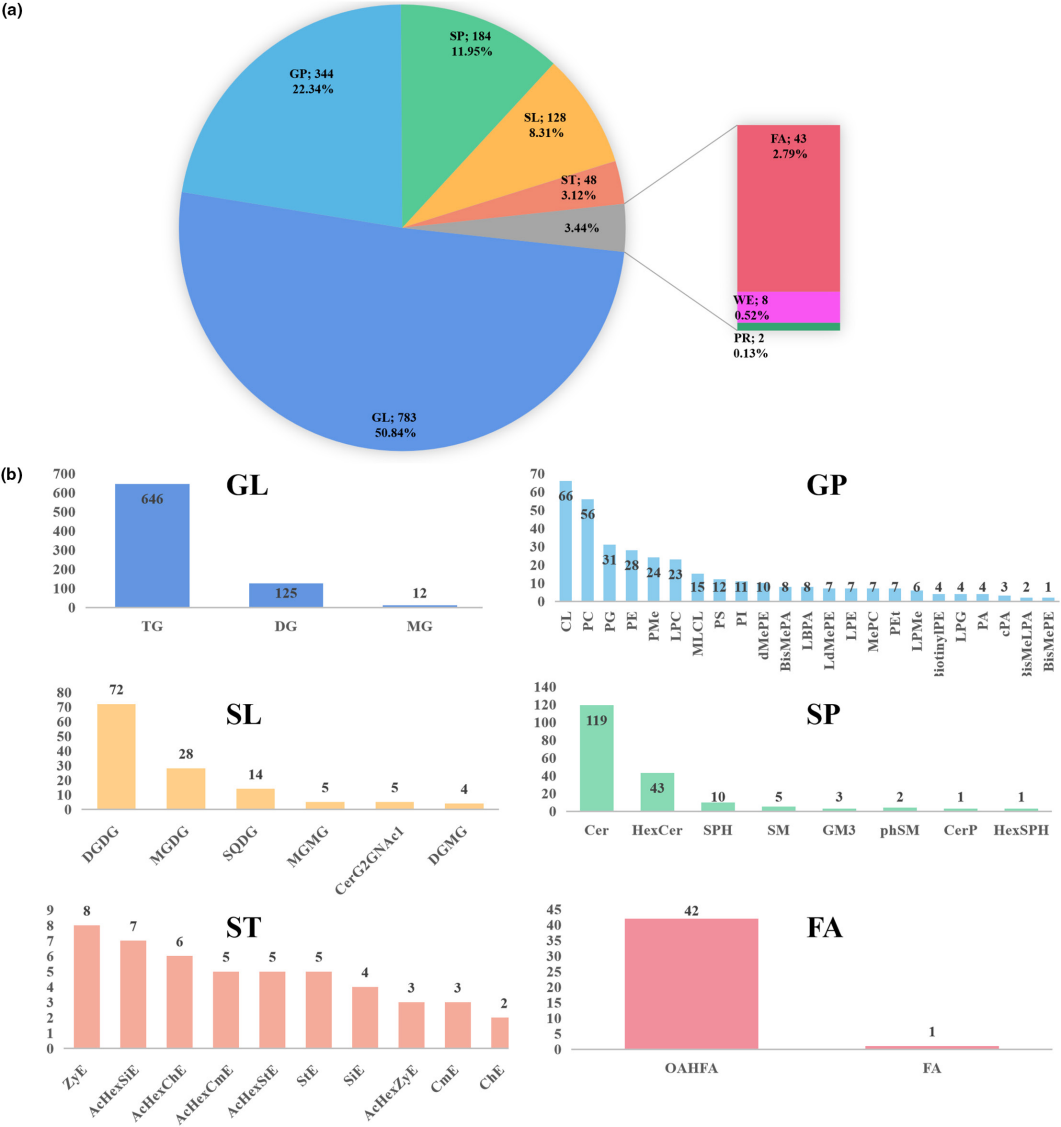
(a) The number and relative percentage of different lipids in oat flour. (b) The type and number of lipid subclasses.

In total, 1540 lipids were identified in samples from four groups of oat flour (S0, C0, S9, and C9). Among them, 1176 were in the positive mode and 364 were in the negative mode (Table S1). The identified lipids were divided into glycerides (GL), glycerophospholipids (GP), sphingolipids (SP), saccharolipids (SL), sterols (ST), fatty acyls (FA), wax esters (WE), and prenol lipids (PR) (Figure 1a). To elaborate, GL had three subclasses (triradylcglycerol [TG], diglyceride [DG], and monoglyceride [MG]); GP had 23 subclasses (cardiolipins [CL], phosphatidylcholine [PC], phosphatidylglycerol [PG], phosphatidylethanolamine [PE], phosphatidylmethanol [PMe], lyso‐phosphatidylcholine [LPC], etc.); SP had eight subclasses (ceramide [Cer], hexosylceramide [HexCer], sphingosine [SPH], etc.). SL had six subclasses (ceramide [Cer], hexosylceramide [HexCer], sphingosine [SPH], etc.); ST had 10 subclasses (zymosteryl [ZyE], acyl hexosyl sitosterol ester [AcHexSiE], acyl hexosyl cholesteryl ester [AcHexChE], etc.); and FA had two subclasses ((O‐acyl)‐1‐hydroxy fatty acid [OAHFA], and fatty acyls [FA]) (Figure 1b). The different types of lipids in the same subclass were summed to obtain the percentage of lipid subclasses. In the oat flour samples, TG showed the highest content (41.95%), followed by DG, Cer, digalactosyldiacylglycerol (DGDG), CL, PC, HexCer, OAHFA, and PG, which accounted for 8.12%, 7.73%, 4.68%, 4.29%, 3.64%, 2.79%, 2.73%, and 2.01%, respectively.

### 3.2 Multivariate statistical analysis of oat flour lipids before and after storage

**FIGURE 2:**
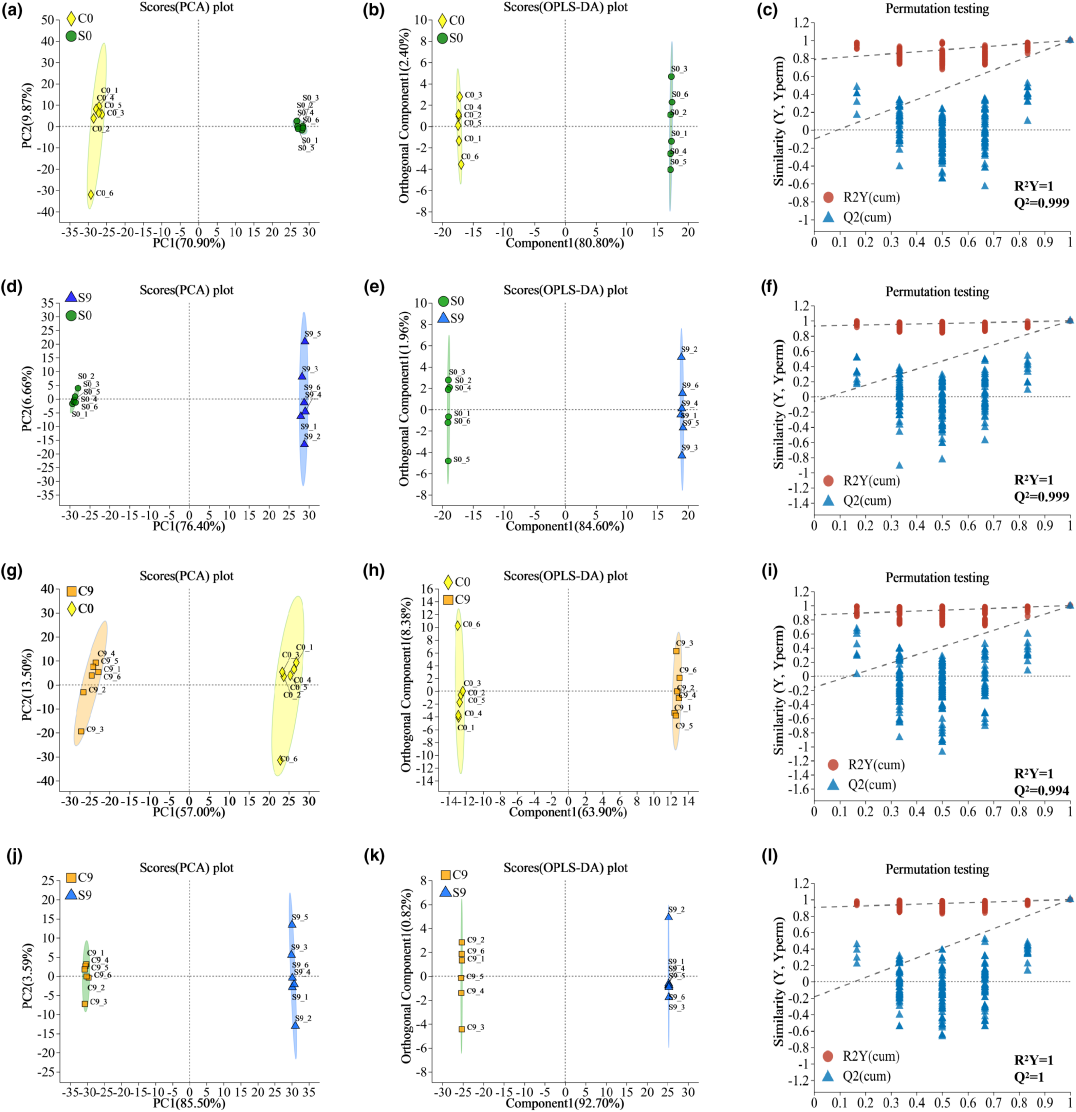
Multivariate statistical analysis of cationic lipids in oat flour before and after storage. (a–c) C0 versus S0 group; (d–f) S9 versus S0 group; (g–i) C9 versus C0 group; (j–l) PCA score plot of lipid classes, OPLS‐DA score plot of lipid classes, and substitution test model of C9 versus S9 group, respectively. OPLS‐DA, orthogonal least squares discriminant analysis; PCA, principal component analysis.

To further investigate the effect of stir‐frying on the storage of oat flour, we performed a group comparison (C0 vs. S0, S9 vs. S0, C9 vs. C0, and C9 vs. S9) multivariate statistical analysis of the lipidomic data of oat flour and visualized the data. The PCA score plots of the overall samples for C0 versus S0, S9 versus S0, C9 versus C0, and C9 versus S9 are shown in Figure 2a,d,g,j, respectively; the differences between all pairs of samples were prominent. Although principal component analysis can effectively extract critical information, it is not sensitive to low correlation variables. OPLS‐DA can solve this problem (How et al., 2023). OPLS‐DA was used to evaluate the differences between samples from different types of oat flour. The abscissa represented the predicted principal component and showed the gap between groups. The ordinate represented the orthogonal principal component and showed the gap within groups. The results of OPLS‐DA also showed the differences between each group of samples (Figure 2). The OPLS‐DA scores showed high variability in the *x*‐axis between the sample groups. Additionally, six replicates of the same sample were scattered on the *y*‐axis, indicating within‐group variability. These results showed that there were significant differences in the cationic lipid metabolites between C0 and S0, S9 and S0, C9 and C0, and C9 and S9; the anionic lipid metabolites showed the same pattern (Figure S3). The *R* ^2^ and *Q* ^2^ values of the OPLS‐DA model were higher in each group, which indicated that this model had good predictive ability and could be used to identify differentially accumulating metabolites. The lipids of oat flour changed significantly after the stir‐frying treatment. Additionally, the lipids of raw oat flour and stir‐fried oat flour changed significantly after storage.

### 3.3 Screening and identification of differential metabolites

**FIGURE 3:**
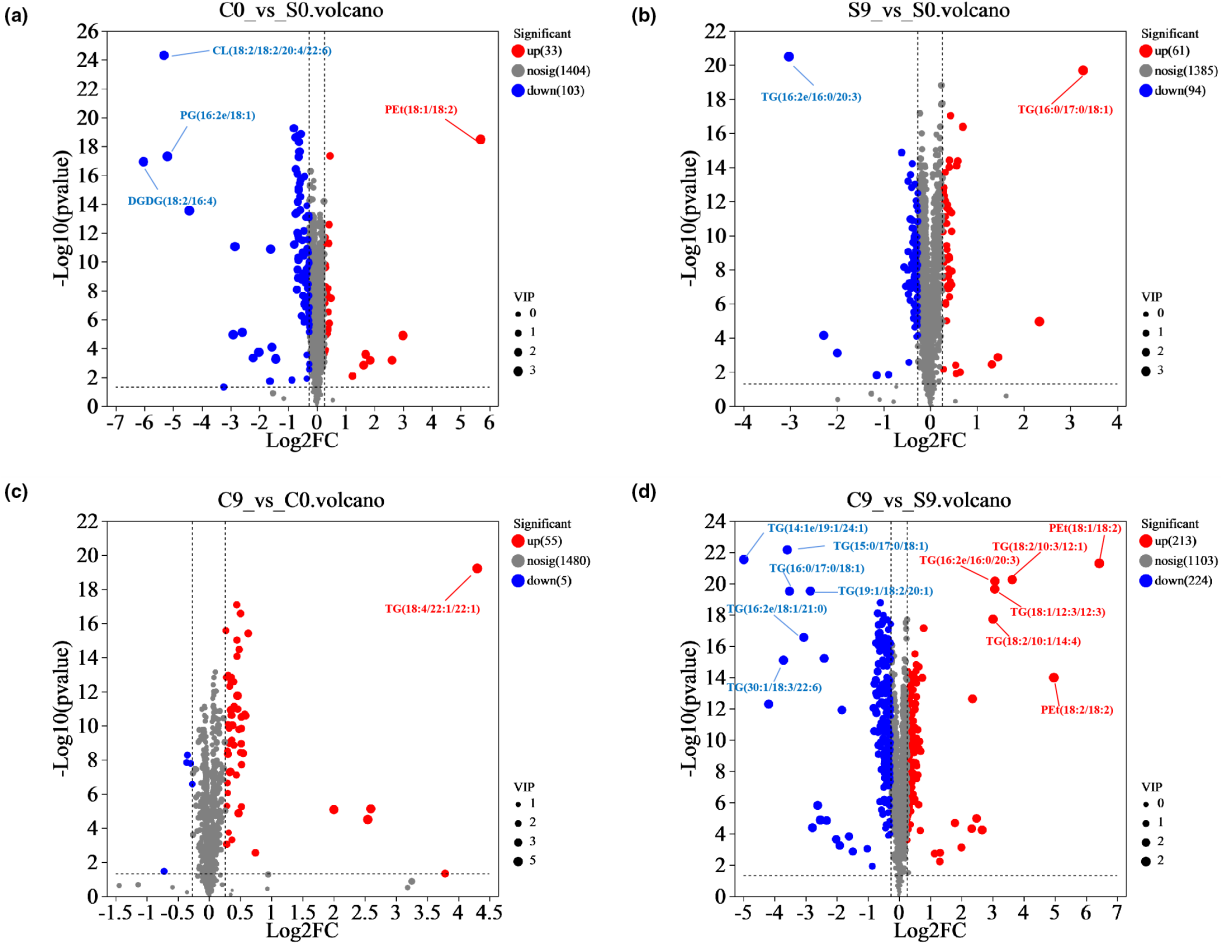
Volcano plots for (a) C0 versus S0, (b) S9 versus S0, (c) C9 versus C0, and (d) C9 versus S9.

To determine metabolite differences in C0 versus S0, S9 versus S0, C9 versus C0, and C9 versus S9, differential metabolite screening was performed for lipids with FC ≥1.2 or FC ≤0.83, and VIP ≥1. The volcano plots obtained by conducting pairwise comparisons of the data on the lipid composition of the four groups are shown in Figure 3. Each dot represents a lipid molecule; blue dots represent differential metabolite downregulation, red dots represent differential metabolite upregulation, and gray dots represent lipid classes that are not significantly different. The abscissa represents the fold change value (log2FC) of the difference in metabolite expression between the groups. The size of the dot represents the contribution value (VIP) of the difference between the two groups, that is, a larger VIP indicates that the metabolite has a larger difference between the two groups.

**FIGURE 4:**
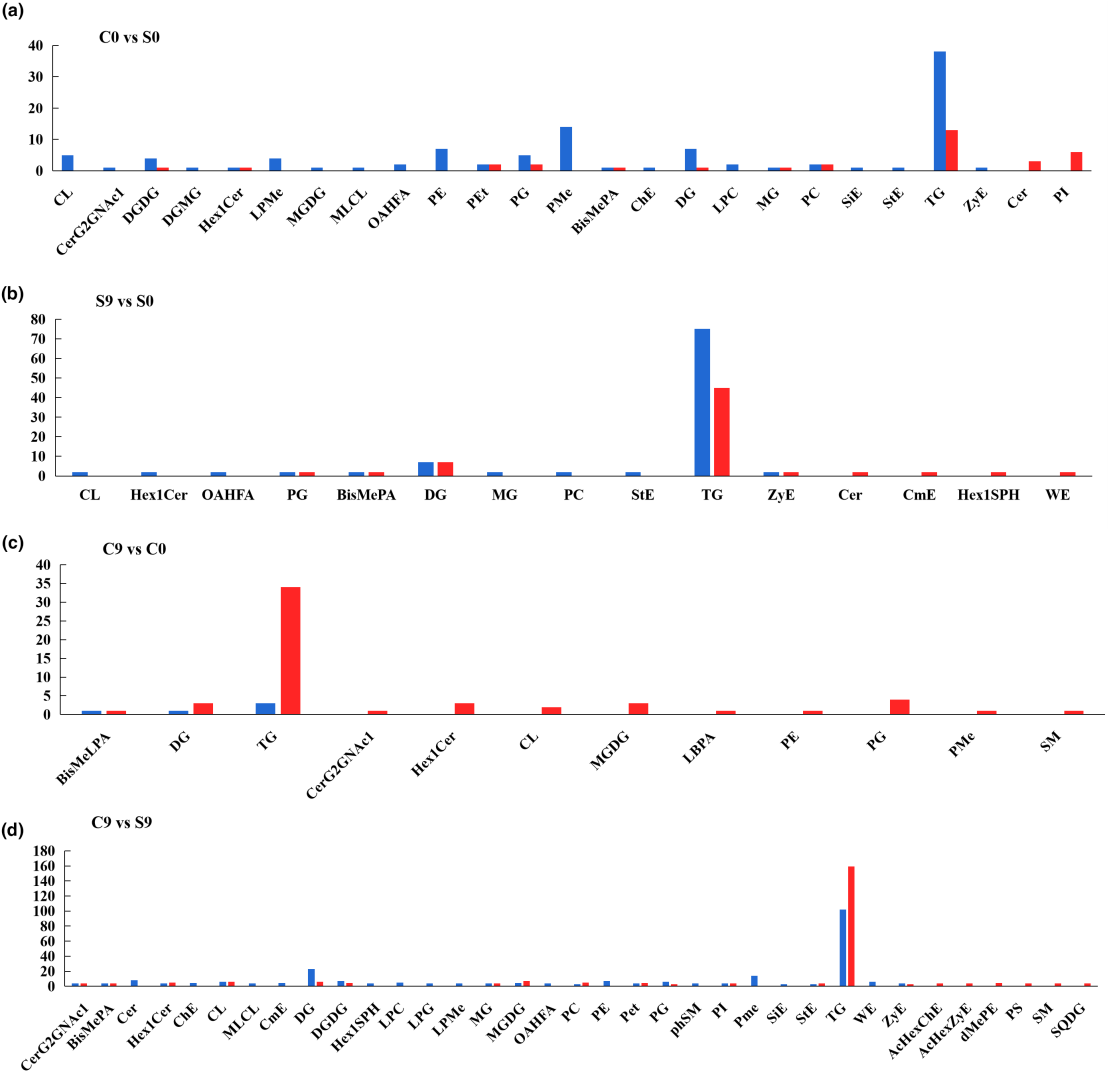
Differences between groups for each metabolite lipid class are presented (blue represents downregulation and red represents upregulation) in groups C0 versus S0 (a), S9 versus S0 (b), C9 versus C0 (c), and C9 versus S9 (d).

In total, 136 differential metabolites were identified in the C0 versus. S0 group, 33 of which were upregulated and 103 were downregulated (Table S2). These results indicated that stir‐frying treatment downregulated most of the lipid metabolites of oat flour. The classification of lipids is shown in Figure 4a. The upregulated lipid metabolites were phosphatidylethanol (PEt) (18:1/18:2), PEt (18:2/18:2), phosphatidylinositol (PI) (18:3/18:2), TG (18:1/12:3/12:3), TG (18:2/10:1/14:4), TG (6:0/18:1/20:2), and hexosylceramide (Hex1Cer) (d18:2/24:2); among them, PEt (18:1/18:2) showed the highest increase of 51.86 fold. A large amount of PEt was accumulated by the thermal decomposition of phosphatidylethanolamine (PE) after oats were stir‐fried at a high temperature. The downregulated lipid metabolites were DGDG (18:2/16:4), PG (16:2e/18:1), CL (18:2/18:2/20:4/22:6), and TG (14:1e/19:1/24:1); DGDG decreased by 0.02 fold (18:2/16:4). Stir‐frying degrades phospholipids and glycolipids in oat flour while oxidizing lipids (Liu et al., 2023). Thus, phospholipids, glycolipids, and TG were significantly lower in C0. Ceramide is ubiquitous in plant plasma membranes. The content of glycosylceramide CerG2GNAc1 (d18:1/18:0) was lower in C0 than that in S0, whereas the content of ceramide Cer (d16:1/26:0), Cer (d16:2/26:0), and Cer (d18:1/26:0) were higher. The disruption of hydrogen bonding under high‐temperature stir‐frying conditions provides additional conformational degrees of freedom, allowing the glycosidic bond to be more readily protonated and the β‐glycosidic bond between the hydroxyl group at the 1‐position of the ceramide and the sugar moiety (D‐galactose or D‐glucose) to be cleaved with a much lower hurdle in turn (Paajanen et al., 2021). Plant sterol esters are generally synthesized from plant sterol and fatty acids by esterification or transesterification (Nagpal et al., 2022). As sitosterol ester (SiE) (20:5) and stigmasterol ester (StE) (19:2) were lower in C0, our findings indicated that stir‐frying treatment also destroyed sterol esters.

In total, 155 differential metabolites were identified in the S9 versus S0 group, of which 61 were upregulated, and 94 were downregulated (Table S3). The metabolites that were significantly upregulated in raw oat flour after storage for 9 months were TG (15:0/17:0/18:1), TG (16:0/17:0/18:1), TG (16:2e/18:1/21:0), and TG (18:1/18:2/21:0) of GL. The significantly downregulated metabolites were TG (16:2e/16:0/20:3), TG (26:0/18:1/18:3), TG (26:0/18:1/24:0), and CL (18:2/18:1/18:2/18:2) of GP. The differential metabolites that showed the most prominent change were in the TG classes (Figure 4b). These changes occurred probably because of the oxidative decomposition of TG during the storage of oat flour (Borén & Taskinen, 2022). The wax lipids WE (3:0/20:1) and WE (3:0/22:2) increased in S9. This indicated that the oil extracted from stored raw oat flour was more coagulable, and this finding matched the results of the experiment. Phospholipids such as PG (28:0/18:3), PC (9:0/18:2), and BisMePA (18:0/12:0) decreased in S9.

In total, 60 differential metabolites were identified in the C9 versus C0 group, 55 of which were upregulated and five downregulated (Table S4). After 9 months of storage, the metabolites significantly upregulated in stir‐fried oat flour were TG (18:2/10:3/12:1), TG (18:2/11:1/11:4), TG (18:4/22:1/22:1), PG (15:0/18:2), and CL (18:2/18:2/20:4/22:6) of GL. The downregulated metabolites were TG (12:0e/12:4/18:1), DG (36:0/18:3), bismethyl lysophosphatidic acid (BisMeLPA) (22:0), TG (19:1/18:4/18:4), and TG (18:4/18:3/20:5) (Figure 4c). Fewer differential metabolites were identified in the C9 versus C0 group than in the S9 versus S0 group, which indicated that changes in lipids during the storage of stir‐fried oat flour were smaller than the changes in lipids during the storage of raw oat flour. These findings are similar to those of published studies on the effects of frying or other high‐temperature treatments of plant seeds on their lipid stability during storage (Huang et al., 2022; Wang, Chen, et al., 2021). Cardiolipin (CL) is a phospholipid dimer consisting of two phosphatidyl residues linked by glycerol, which is implicated in the assembly and activity of a variety of protein complexes in mitochondria and stabilizes protein structures (Schlame & Greenberg, 2017). CL (22:2/15:0/16:0/15:1) and CL (18:2/18:2/20:4/22:6) were upregulated in C9, which indicated that PA was metabolized to PG by glycerophospholipids when stir‐fried oat flour was stored. The glycosylceramide series (CerG2GNAc) (d19:0/16:0), hexosylceramide (Hex1Cer) (d18:1/20:1), lysobisophosphatidic acid (LBPA) (16:0/18:0), and monogalactosyldiglyceride (MGDG) (16:0/23:5), were also upregulated in C9.

In total, 437 differential metabolites were identified in the C9 versus S9 group, of which 213 were upregulated and 224 were downregulated (Table S5). The differential metabolites upregulated in C9, relative to their respective content in S9, were mainly PEt (18:1/18:2), PEt (18:2/18:2), TG (18:2/10:3/12:1), TG (16:2e/16:0/20:3), TG (18:1/12:3/12:3), and TG (18:2/10:1/14:4). The significantly downregulated differential metabolites were mainly TG (15:0/17:0/18:1), TG (14:1e/19:1/24:1), TG (16:0/17:0/18:1), TG (19:1/18:2/20:1), TG (16:2e/18:1/21:0), and TG (30:1/18:3/22:6) (Figure 4d). Our results indicated that there were significantly more differential metabolites in the C9 versus S9 group than in the C0 versus S0 group. Thus, the difference in lipid composition was more pronounced after the two types of oat flours were stored, and different kinds of differential lipid metabolites were produced during the storage of the two types of oat flours.

### 3.4 Lipid metabolic pathway analysis

Pathway enrichment analysis of differential metabolites in different groups of oat flour can help elucidate the mechanism by which stir‐frying affects oat flour aging in storage. The identified differential lipid metabolites were uploaded to the KEGG database to collect relevant information, and the metabolic pathways were topologically analyzed using MetaboAnalyst 5.0. The results were presented using a bubble diagram to show the lipid changes in the metabolic pathways during oat flour storage. Each bubble represented a metabolic pathway, and the size and color of the bubble represented the impact value (impact value) of the enriched pathway and the enrichment significance‐log10 (*p*‐value) of the metabolite‐involved pathway, respectively.

**FIGURE 5:**
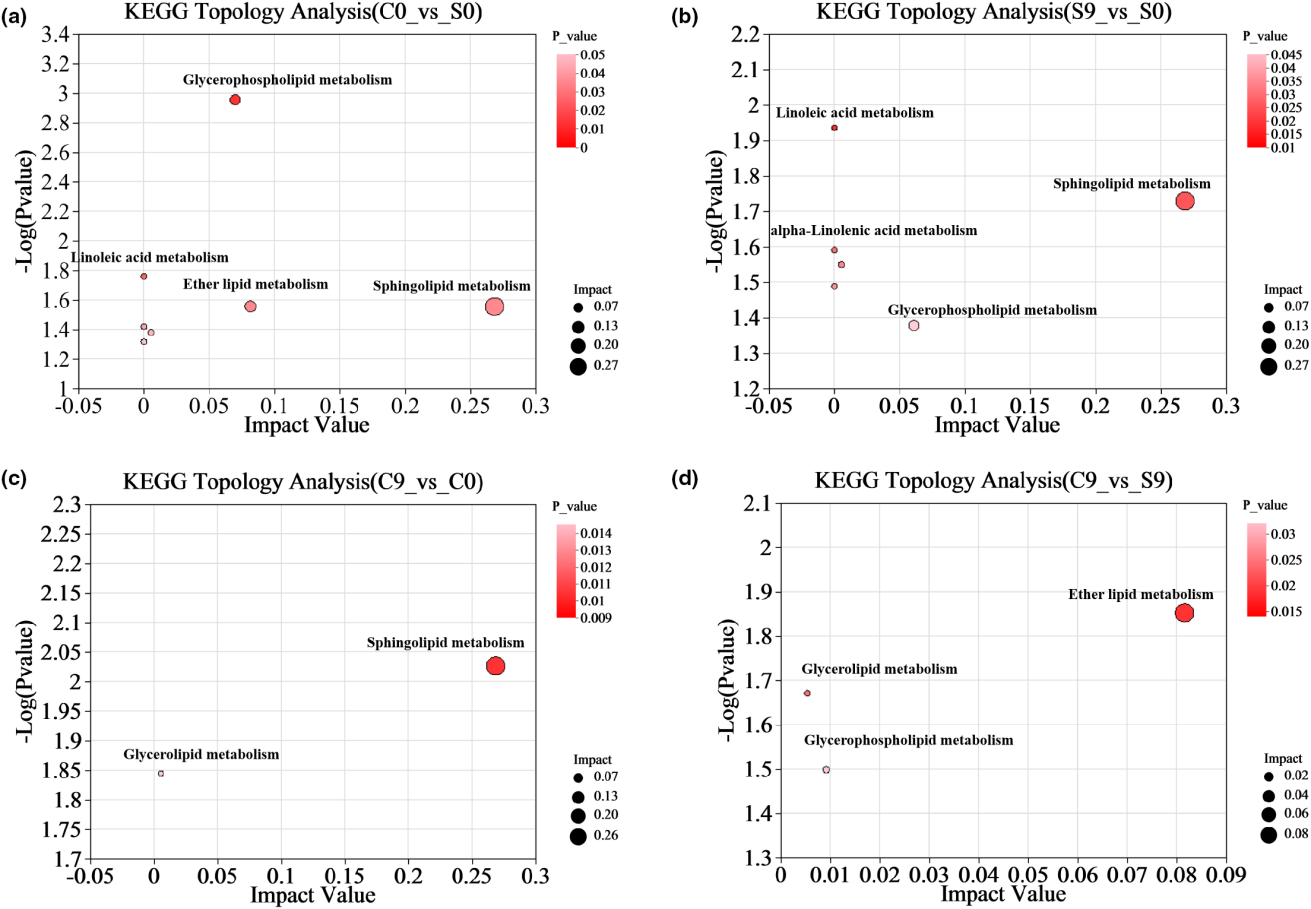
Bubble diagrams of the KEGG metabolic pathway enrichment topology analysis for differential metabolites in groups C0 versus S0 (a), S9 versus S0 (b), C9 versus C0 (c), and C9 versus S9 (d).

The KEGG metabolic pathway enrichment bubble map of differential metabolites in the C0 versus S0 group is shown in Figure 5a. Differential metabolites were enriched in seven metabolic pathways, including the metabolism of sphingolipids, ether lipids, glycerophospholipids, glycerides, linoleic acid, α‐linolenic acid, and arachidonic acid. Our results indicated that stir‐frying treatment influenced the above metabolic pathways of oat flour. Among them, the most important metabolic pathway was sphingolipid metabolism, followed by ether lipid metabolism and glycerophospholipid metabolism.

The KEGG metabolic pathway enrichment bubble plots of differential metabolites in the S9 versus S0 group are shown in Figure 5b. The differential metabolites were enriched in six metabolic pathways, including the metabolism of sphingolipids, glycerophospholipids, glycerides, α‐linolenic acid, linoleic acid, and arachidonic acid. Our results indicated that the above metabolic pathways changed during the storage of raw oat flour. However, the differential metabolites in the C9 versus C0 group (Figure 5c) were enriched only in the sphingolipid metabolism and glyceride metabolism pathways. These results showed that the metabolic pathways of glycerophospholipid metabolism, α‐linolenic acid metabolism, linoleic acid metabolism, and arachidonic acid metabolism in oat flour during storage were slowed down by stir‐frying treatment. These changes increased lipid stability during storage and prolonged the storage period of oat flour. Sphingolipid metabolism was the most important metabolic pathway in the S9 versus S0 and C9 versus C0 groups, which indicated that sphingolipid metabolism was an important lipid metabolic pathway during the storage of oat flour.

At the end of the storage period (C9 vs. S9), ether lipid metabolism had the largest bubble size and darkest color, which indicated that it was an important pathway during oat flour storage (Figure 5d). The differential metabolites were also enriched in the glycerophospholipid metabolism and glyceride metabolism pathways. Sphingolipid metabolism, linoleic acid metabolism, α‐linolenic acid metabolism, and arachidonic acid metabolism were absent in the C9 versus S9 group but present in the C0 versus S0 group.

**FIGURE 6:**
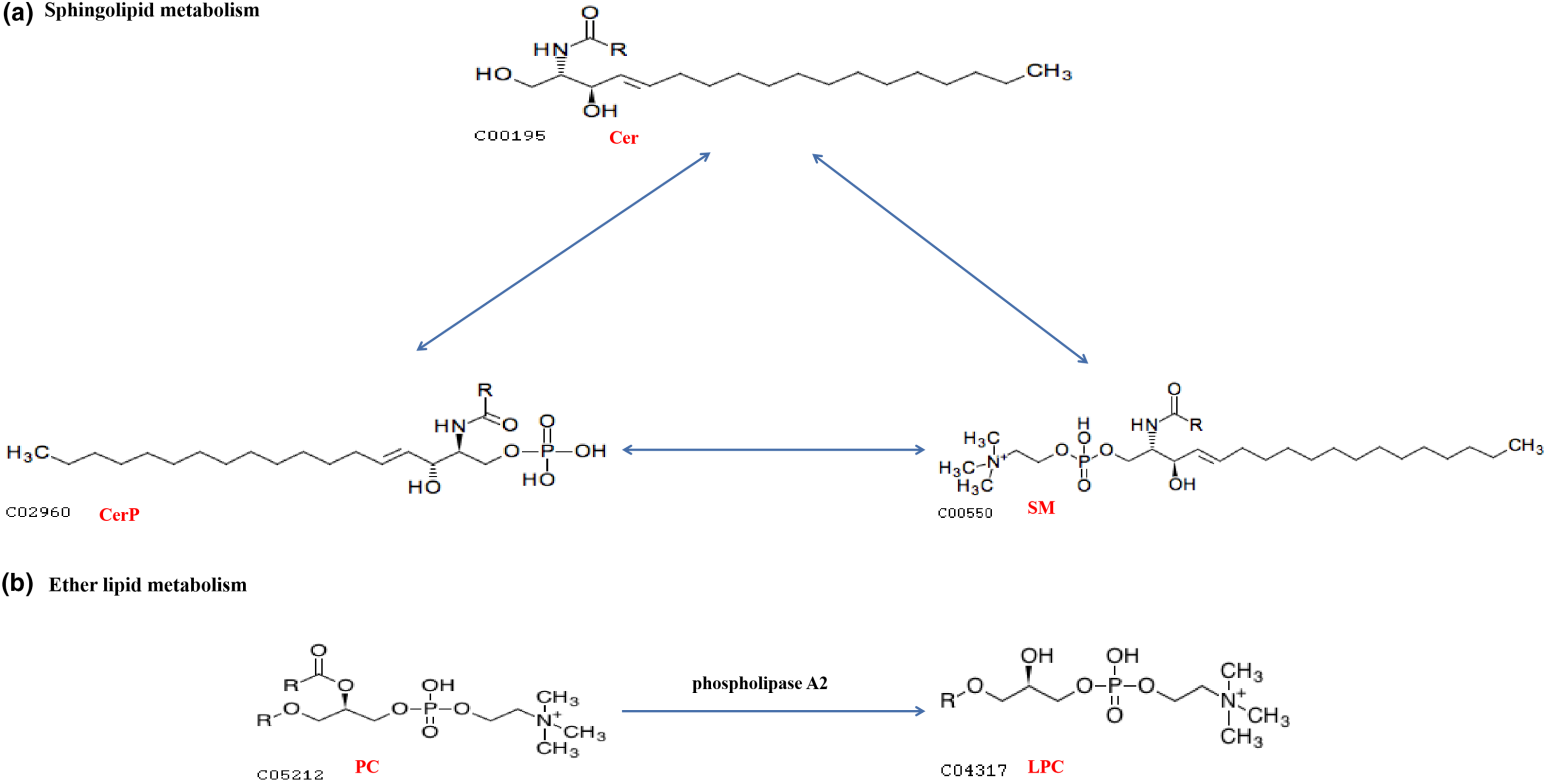
Important lipid metabolism pathways during the storage of oat flour (“c + numbers” indicate the ID of each lipid subclass). Sphingolipid metabolism (a), Ether lipid metabolism (b).

Sphingolipids have a variety of functions in organisms. Sphingolipid metabolism involves several processes, such as the synthesis, degradation, transport, and utilization of sphingolipid molecules by organisms in vivo (Körner & Fröhlich, 2022). The differential metabolite involved in sphingolipid metabolism in the C0 versus S0 group was Cer (d18:1/26:0), which was upregulated 1.307‐fold. A conversion reaction occurred among sphingomyelin, ceramide phosphate (CerP), glucosylceramide, and ceramide (Figure 6a) (Haslam & Feussner, 2022).

PC can be converted to LPC by phospholipase A2 (PLA2) (Figure 6b) (Gao et al., 2022; Vittos et al., 2012). Lipase activity was measured with reference to GB/T 5523‐2008, the enzyme activity of C0 was 4.89 mg/g (in KOH), compared with the enzyme activity of S0 of 43.76 mg/g (in KOH), which was reduced to 11%. Therefore, we argued that the inactivation of phospholipase A2 in oat flour after stir‐frying significantly degraded LPC. The differential metabolite involved in ether lipid metabolism in the C0 versus S0 group was lysophospholipid LPC (18:0), which was downregulated 0.8624‐fold. We also found that 15 differential metabolites were involved in glycerophospholipid metabolism, including 12 LPC classes, which were downregulated, and three PC classes, which were upregulated. LPC (18:0) was likewise a significantly downregulated metabolite in the C9 versus S9 group.

## 4 CONCLUSIONS

In this study, we investigated the differences in the lipid profiles between raw and stir‐fried oat flour before and after storage. During storage, 1540 lipids were identified. The comparison of lipids before and after storage showed that the lipids in raw and stir‐fried oat flour changed significantly. After stir‐frying treatment, most lipid metabolites of oat flour were significantly downregulated, and the changes in lipids during storage were reduced. Sphingolipid metabolism and ether lipid metabolism were the key metabolic pathways, and Cer, PC, and LPC were identified as key lipid metabolites during storage. Stir‐frying inhibited lipid metabolic pathways such as glycerophospholipid metabolism, α‐linolenic acid metabolism, linoleic acid metabolism, and arachidonic acid metabolism during storage of oat flour and improved lipid stability and quality during storage. Our findings might provide a theoretical basis for the processing and storage of oat flour. Although the study of the physical properties of fried oats is necessary, lipid molecules are also of research value, and further quantitative analysis of Cer, PC, and LPC‐targeted lipids could be used as an indicator of the biological evaluation of stir‐frying oat flour for processing and storage.

## AUTHOR CONTRIBUTIONS

**Yuanyuan Zhang:** Conceptualization, methodology, software. **Minjun Sun:** Data curation, writing—original draft preparation. **Rui Huo:** Visualization, investigation. **Qixin Gu:** Supervision. **Ying Miao:** Software, validation. **Meili Zhang:** Writing—reviewing and editing. All authors read and approved the final manuscript.

## CONFLICT OF INTEREST STATEMENT

There are no conflicts to declare.

## Supporting information


Data S1.

## Data Availability

The authors confirm that the data supporting the findings of this study are available within the article [and/or its supplementary materials].
