# Supplementary material for: The effect of stir‐frying on the aging of oat flour during storage: A study based on lipidomics
Source: Food Sci Nutr. 2024 Feb 20;12(5):3188–98. doi: 10.1002/fsn3.3985 (PMC11077182; doi:10.1002/fsn3.3985)
Supplement: Supplementary file 1 — Data S1. [file FSN3-12-3188-s001.docx]

**Supplementary materials**

**
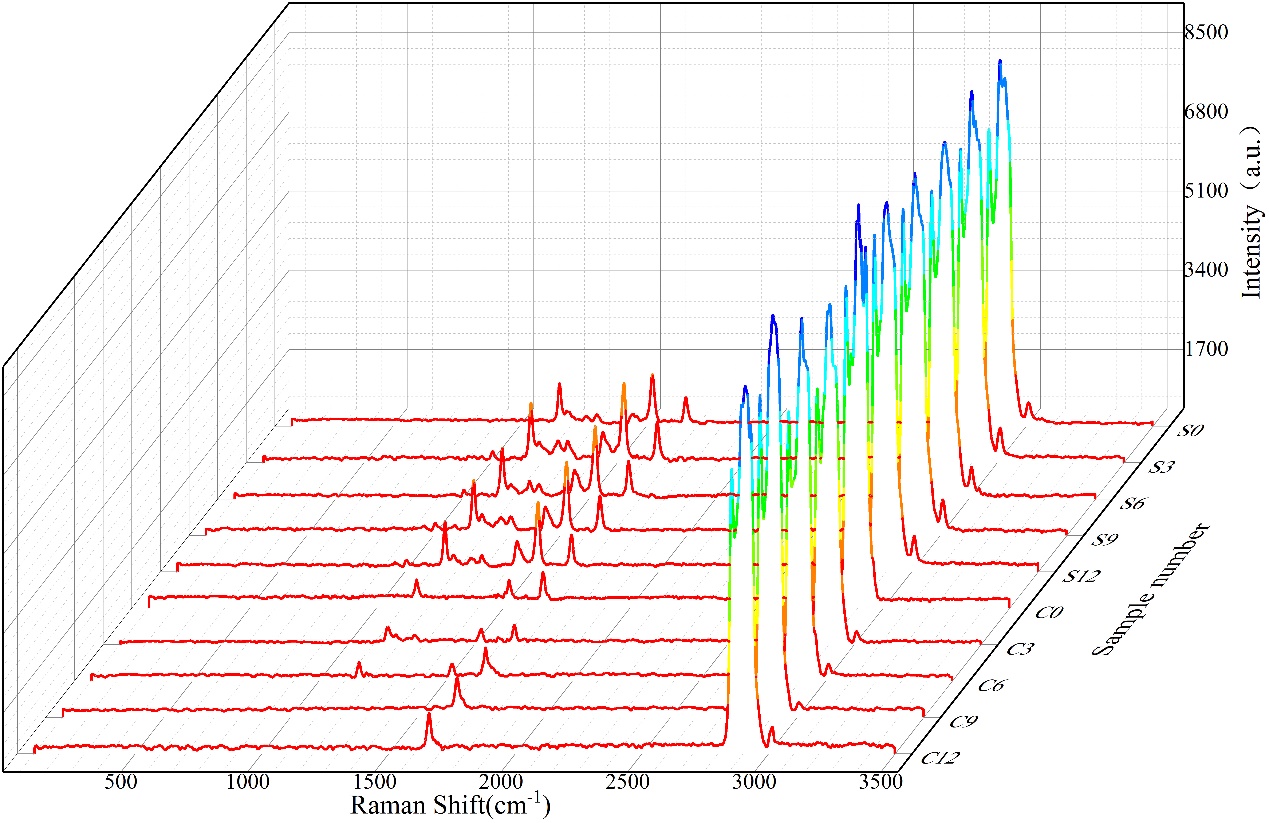
**

Fig.S1 Raman spectra 3D waterfall chart of oat oil

**
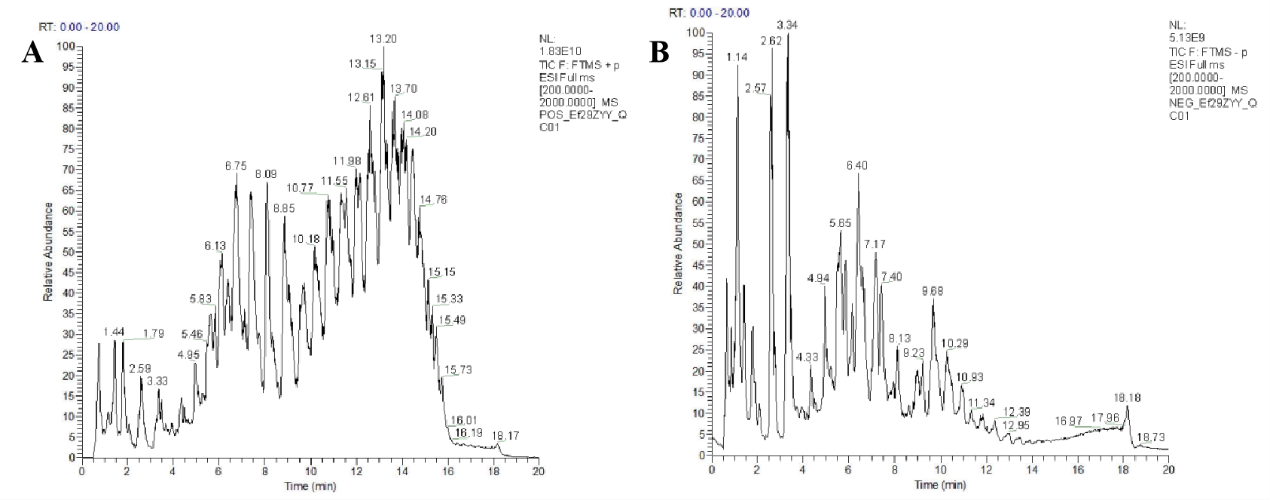
**

Fig.S2 (A) Total ion flow diagram of quality control samples ESI (+); (B) Total ion flow diagram of quality control sample ESI (-).

**
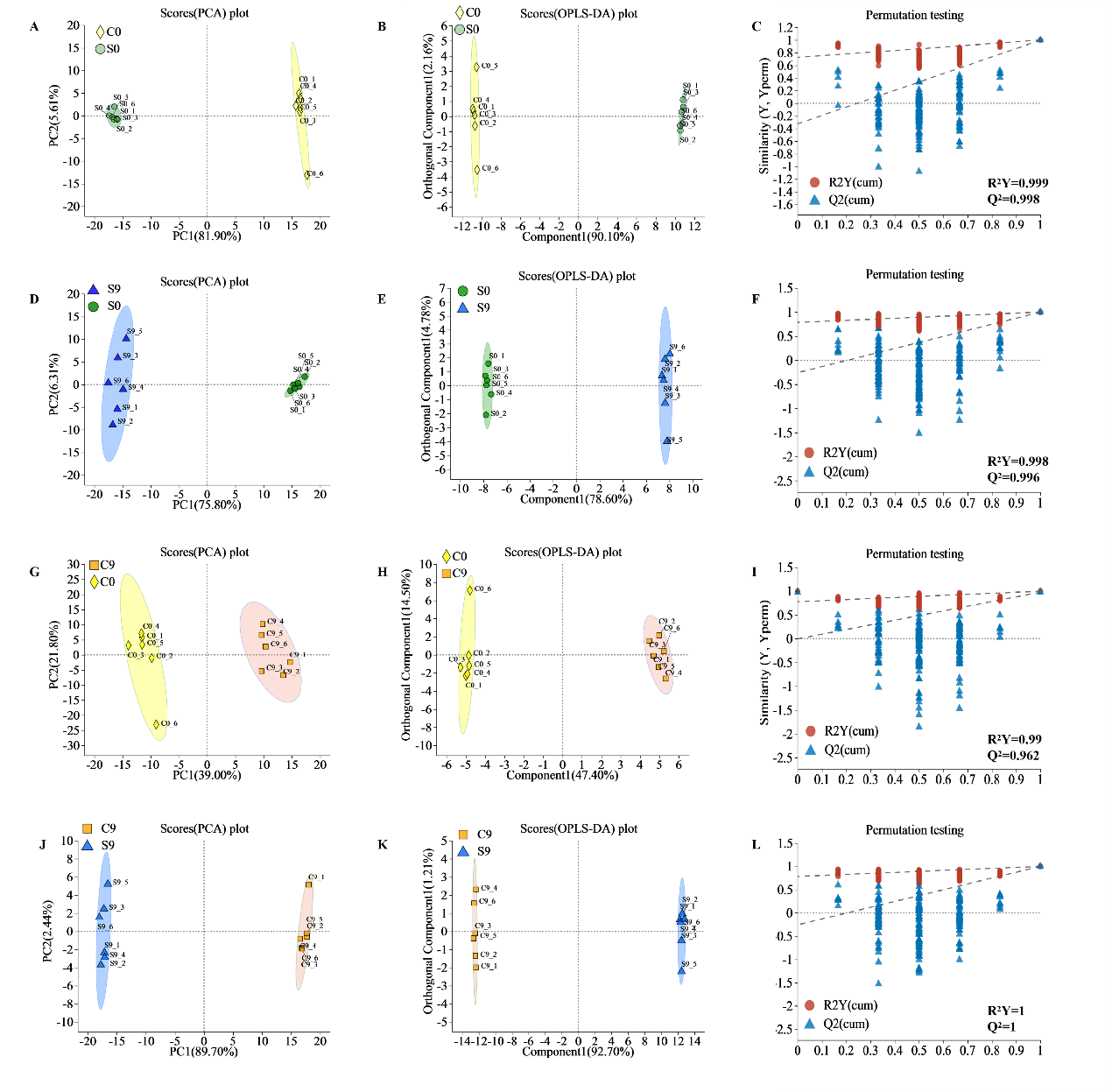
**

Fig. S3 Multivariate statistical analysis of anionic lipids in oat flour before and after storage. (A), (B), and (C) C0 vs. S0 group; (D), (E), and (F) S9 vs. S0 group; (G), (H), and (I) C9 vs. C0 group; (J), (K), and (L) PCA score plot of lipid classes, OPLS-DA score plot of lipid classes, and substitution test model of C9 vs. S9 group, respectively.

Table S1 Classification of lipid metabolites in oat meal

| ID | Formula | Metabolite | Class | Subclass | M/Z | Retention time |
| --- | --- | --- | --- | --- | --- | --- |
| pos_1 | C51 H92 O7 N1 | AcHexChE(18:1) | Sterol Lipids, ST | Acyl hexosyl cholesteryl Ester | 830.6868305 | 10.3433 |
| pos_2 | C52 H90 O7 N1 | AcHexChE(19:3) | Sterol Lipids, ST | Acyl hexosyl cholesteryl Ester | 840.6711805 | 9.276 |
| pos_3 | C55 H99 O7 | AcHexChE(22:0) | Sterol Lipids, ST | Acyl hexosyl cholesteryl Ester | 871.7385315 | 9.5513 |
| pos_4 | C57 H101 O7 | AcHexChE(24:1) | Sterol Lipids, ST | Acyl hexosyl cholesteryl Ester | 897.7541815 | 9.5539 |
| pos_5 | C59 H108 O7 N1 | AcHexChE(26:1) | Sterol Lipids, ST | Acyl hexosyl cholesteryl Ester | 942.8120305 | 12.251 |
| pos_7 | C61 H110 O7 N1 | AcHexChE(28:2) | Sterol Lipids, ST | Acyl hexosyl cholesteryl Ester | 968.8276805 | 12.1328 |
| pos_8 | C50 H92 O7 N1 | AcHexCmE(16:0) | Sterol Lipids, ST | Acyl hexosyl campesterol | 818.6868305 | 10.6367 |
| pos_9 | C50 H90 O7 N1 | AcHexCmE(16:1) | Sterol Lipids, ST | Acyl hexosyl campesterol | 816.6711805 | 9.962 |
| pos_10 | C52 H94 O7 N1 | AcHexCmE(18:1) | Sterol Lipids, ST | Acyl hexosyl campesterol | 844.7024805 | 10.6626 |
| pos_11 | C52 H92 O7 N1 | AcHexCmE(18:2) | Sterol Lipids, ST | Acyl hexosyl campesterol | 842.6868305 | 10.0018 |
| pos_12 | C52 H90 O7 N1 | AcHexCmE(18:3) | Sterol Lipids, ST | Acyl hexosyl campesterol | 840.6711805 | 9.2734 |
| pos_13 | C50 H90 O7 N1 | AcHexSiE(15:1) | Sterol Lipids, ST | Acyl hexosyl sitosterol ester | 816.6711805 | 9.9468 |
| pos_14 | C51 H94 O7 N1 | AcHexSiE(16:0) | Sterol Lipids, ST | Acyl hexosyl sitosterol ester | 832.7024805 | 10.8948 |
| pos_15 | C53 H98 O7 N1 | AcHexSiE(18:0) | Sterol Lipids, ST | Acyl hexosyl sitosterol ester | 860.7337805 | 11.7483 |
| pos_16 | C53 H96 O7 N1 | AcHexSiE(18:1) | Sterol Lipids, ST | Acyl hexosyl sitosterol ester | 858.7181305 | 10.945 |
| pos_17 | C53 H94 O7 N1 | AcHexSiE(18:2) | Sterol Lipids, ST | Acyl hexosyl sitosterol ester | 856.7024805 | 10.2918 |
| pos_18 | C53 H92 O7 N1 | AcHexSiE(18:3) | Sterol Lipids, ST | Acyl hexosyl sitosterol ester | 854.6868305 | 9.6953 |
| pos_21 | C57 H106 O7 N1 | AcHexSiE(22:0) | Sterol Lipids, ST | Acyl hexosyl sitosterol ester | 916.7963805 | 13.3034 |
| pos_23 | C35 H62 O6 N1 | AcHexStE(0:0) | Sterol Lipids, ST | Acyl hexosyl stimasterol ester | 592.4571655 | 4.347 |
| pos_24 | C51 H92 O7 N1 | AcHexStE(16:0) | Sterol Lipids, ST | Acyl hexosyl stimasterol ester | 830.6868305 | 10.3435 |
| pos_25 | C53 H96 O7 N1 | AcHexStE(18:0) | Sterol Lipids, ST | Acyl hexosyl stimasterol ester | 858.7181305 | 10.948 |
| pos_26 | C53 H94 O7 N1 | AcHexStE(18:1) | Sterol Lipids, ST | Acyl hexosyl stimasterol ester | 856.7024805 | 10.2909 |
| pos_27 | C53 H92 O7 N1 | AcHexStE(18:2) | Sterol Lipids, ST | Acyl hexosyl stimasterol ester | 854.6868305 | 9.6935 |
| pos_29 | C50 H90 O7 N1 | AcHexZyE(17:0) | Sterol Lipids, ST | Acyl hexosyl zymosteryl | 816.6711805 | 9.939 |
| pos_30 | C51 H92 O7 N1 | AcHexZyE(18:0) | Sterol Lipids, ST | Acyl hexosyl zymosteryl | 830.6868305 | 10.595 |
| pos_33 | C57 H97 O7 | AcHexZyE(24:2) | Sterol Lipids, ST | Acyl hexosyl zymosteryl | 893.7228815 | 7.7531 |
| pos_34 | C52 H94 O10 N3 S1 P1 Na1 | BiotinylPE(18:2/19:0) | Glycerophospholipids, GP | Biotinyl phosphatidylethanolamine | 1006.628978 | 2.2379 |
| pos_35 | C54 H95 O10 N3 S1 P1 | BiotinylPE(18:3/21:1) | Glycerophospholipids, GP | Biotinyl phosphatidylethanolamine | 1008.647033 | 2.1837 |
| pos_36 | C24 H50 O7 N0 P1 | BisMeLPA(19:0) | Glycerophospholipids, GP | Bis-Methyl Lyso-phosphatidic acid | 481.3288695 | 3.339 |
| pos_38 | C27 H56 O7 N0 P1 | BisMeLPA(22:0) | Glycerophospholipids, GP | Bis-Methyl Lyso-phosphatidic acid | 523.3758195 | 3.788 |
| pos_39 | C35 H69 O8 N0 P1 Na1 | BisMePA(18:0/12:0) | Glycerophospholipids, GP | Bis-Methyl Phosphatidic acid | 671.4622295 | 5.596 |
| pos_40 | C38 H75 O8 N1 P1 | BisMePA(15:0/18:2) | Glycerophospholipids, GP | Bis-Methyl Phosphatidic acid | 704.5224835 | 3.879 |
| pos_41 | C38 H73 O8 N1 P1 | BisMePA(15:0/18:3) | Glycerophospholipids, GP | Bis-Methyl Phosphatidic acid | 702.5068335 | 4.46 |
| pos_42 | C39 H77 O8 N1 P1 | BisMePA(16:0/18:2) | Glycerophospholipids, GP | Bis-Methyl Phosphatidic acid | 718.5381335 | 6.0189 |
| pos_43 | C39 H75 O8 N1 P1 | BisMePA(16:0/18:3) | Glycerophospholipids, GP | Bis-Methyl Phosphatidic acid | 716.5224835 | 5.4375 |
| pos_44 | C41 H79 O8 N1 P1 | BisMePA(18:1/18:2) | Glycerophospholipids, GP | Bis-Methyl Phosphatidic acid | 744.5537835 | 6.1662 |
| pos_45 | C41 H77 O8 N1 P1 | BisMePA(18:2/18:2) | Glycerophospholipids, GP | Bis-Methyl Phosphatidic acid | 742.5381335 | 5.5151 |
| pos_46 | C41 H75 O8 N1 P1 | BisMePA(18:3/18:2) | Glycerophospholipids, GP | Bis-Methyl Phosphatidic acid | 740.5224835 | 4.9725 |
| pos_47 | C43 H79 O8 N1 P1 | BisMePE(18:2/18:2) | Glycerophospholipids, GP | Bis-Methyl Phosphatidylethanolamine | 768.5537835 | 6.0919 |
| pos_48 | C28 H58 O3 N1 | Cer(d12:0/16:0) | Sphingolipids, SP | Ceramides | 456.4411205 | 3.2362 |
| pos_49 | C30 H62 O3 N1 | Cer(d16:0/14:0) | Sphingolipids, SP | Ceramides | 484.4724205 | 3.9403 |
| pos_50 | C30 H62 O3 N1 | Cer(d12:0/18:0) | Sphingolipids, SP | Ceramides | 484.4724205 | 4.2094 |
| pos_51 | C32 H66 O3 N1 | Cer(d16:0/16:0) | Sphingolipids, SP | Ceramides | 512.5037205 | 4.8316 |
| pos_52 | C34 H70 O3 N1 | Cer(d16:0/18:0) | Sphingolipids, SP | Ceramides | 540.5350205 | 5.828 |
| pos_53 | C34 H70 O4 N1 | Cer(d16:0/18:0+O) | Sphingolipids, SP | Ceramides | 556.5299355 | 4.6679 |
| pos_54 | C34 H68 O3 N1 | Cer(d16:0/18:1) | Sphingolipids, SP | Ceramides | 538.5193705 | 4.9093 |
| pos_55 | C34 H66 O3 N1 | Cer(d16:0/18:2) | Sphingolipids, SP | Ceramides | 536.5037205 | 4.2383 |
| pos_56 | C36 H74 O3 N1 | Cer(d20:0/16:0) | Sphingolipids, SP | Ceramides | 568.5663205 | 6.7816 |
| pos_57 | C36 H72 O4 N1 | Cer(d18:1/18:0+O) | Sphingolipids, SP | Ceramides | 582.5455855 | 6.6438 |
| pos_58 | C36 H70 O3 N1 | Cer(d20:2/16:0) | Sphingolipids, SP | Ceramides | 564.5350205 | 8.1434 |
| pos_59 | C36 H70 O4 N1 | Cer(d18:1/18:1+O) | Sphingolipids, SP | Ceramides | 580.5299355 | 5.9611 |
| pos_60 | C36 H70 O4 N1 | Cer(d18:2/18:0+O) | Sphingolipids, SP | Ceramides | 580.5299355 | 6.1993 |
| pos_61 | C36 H68 O3 N1 | Cer(d18:2/18:1) | Sphingolipids, SP | Ceramides | 562.5193705 | 6.5879 |
| pos_63 | C36 H68 O4 N1 | Cer(d18:2/18:1+O) | Sphingolipids, SP | Ceramides | 578.5142855 | 5.5401 |
| pos_64 | C36 H65 O5 N1 Na1 | Cer(d18:2/18:2+2O) | Sphingolipids, SP | Ceramides | 614.4754955 | 1.2786 |
| pos_65 | C38 H78 O3 N1 | Cer(d22:0/16:0) | Sphingolipids, SP | Ceramides | 596.5976205 | 7.9266 |
| pos_66 | C38 H76 O3 N1 | Cer(d20:0/18:1) | Sphingolipids, SP | Ceramides | 594.5819705 | 6.8582 |
| pos_67 | C38 H76 O4 N1 | Cer(d18:1/20:0+O) | Sphingolipids, SP | Ceramides | 610.5768855 | 7.8906 |
| pos_68 | C38 H74 O3 N1 | Cer(d20:0/18:2) | Sphingolipids, SP | Ceramides | 592.5663205 | 6.0617 |
| pos_70 | C38 H74 O4 N1 | Cer(d18:2/20:0+O) | Sphingolipids, SP | Ceramides | 608.5612355 | 7.5885 |
| pos_71 | C38 H72 O3 N1 | Cer(d18:2/20:1) | Sphingolipids, SP | Ceramides | 590.5506705 | 7.5902 |
| pos_72 | C38 H72 O3 N1 | Cer(d20:2/18:1) | Sphingolipids, SP | Ceramides | 590.5506705 | 8.2133 |
| pos_73 | C38 H70 O3 N1 | Cer(d20:2/18:2) | Sphingolipids, SP | Ceramides | 588.5350205 | 7.4878 |
| pos_75 | C40 H82 O3 N1 | Cer(d22:0/18:0) | Sphingolipids, SP | Ceramides | 624.6289205 | 9.1373 |
| pos_76 | C40 H80 O3 N1 | Cer(d22:0/18:1) | Sphingolipids, SP | Ceramides | 622.6132705 | 7.9973 |
| pos_77 | C40 H78 O3 N1 | Cer(d22:0/18:2) | Sphingolipids, SP | Ceramides | 620.5976205 | 7.1493 |
| pos_78 | C40 H76 O3 N1 | Cer(d18:2/22:1) | Sphingolipids, SP | Ceramides | 618.5819705 | 8.6023 |
| pos_79 | C42 H84 O3 N1 | Cer(d20:0/22:1) | Sphingolipids, SP | Ceramides | 650.6445705 | 9.0286 |
| pos_81 | C42 H84 O4 N1 | Cer(d18:1/24:0+O) | Sphingolipids, SP | Ceramides | 666.6394855 | 9.4166 |
| pos_82 | C42 H82 O4 N1 | Cer(d18:2/24:0+O) | Sphingolipids, SP | Ceramides | 664.6238355 | 9.0084 |
| pos_83 | C42 H80 O3 N1 | Cer(d18:2/24:1) | Sphingolipids, SP | Ceramides | 646.6132705 | 9.602 |
| pos_84 | C42 H78 O3 N1 | Cer(d18:2/24:2) | Sphingolipids, SP | Ceramides | 644.5976205 | 8.4658 |
| pos_85 | C43 H86 O4 N1 | Cer(d18:1/25:0+O) | Sphingolipids, SP | Ceramides | 680.6551355 | 9.8792 |
| pos_86 | C34 H70 O4 N1 | Cer(t18:0/16:0) | Sphingolipids, SP | Ceramides | 556.5299355 | 4.6697 |
| pos_87 | C36 H74 O4 N1 | Cer(t18:0/18:0) | Sphingolipids, SP | Ceramides | 584.5612355 | 5.6165 |
| pos_88 | C36 H70 O3 N1 | Cer(t18:0/18:1) | Sphingolipids, SP | Ceramides | 564.5350205 | 8.132 |
| pos_89 | C36 H70 O3 N1 | Cer(t20:1/16:0) | Sphingolipids, SP | Ceramides | 564.5350205 | 8.3499 |
| pos_90 | C36 H66 O2 N1 | Cer(t18:1/18:1) | Sphingolipids, SP | Ceramides | 544.5088055 | 6.5841 |
| pos_92 | C38 H78 O4 N1 | Cer(t18:0/20:0) | Sphingolipids, SP | Ceramides | 612.5925355 | 8.5367 |
| pos_93 | C38 H74 O3 N1 | Cer(t20:1/18:0) | Sphingolipids, SP | Ceramides | 592.5663205 | 9.1509 |
| pos_94 | C38 H76 O5 N1 | Cer(t18:1/20:0+O) | Sphingolipids, SP | Ceramides | 626.5718005 | 7.2459 |
| pos_95 | C38 H72 O3 N1 | Cer(t18:1/20:1) | Sphingolipids, SP | Ceramides | 590.5506705 | 7.5909 |
| pos_96 | C38 H72 O3 N1 | Cer(t20:1/18:1) | Sphingolipids, SP | Ceramides | 590.5506705 | 8.2053 |
| pos_97 | C38 H70 O3 N1 | Cer(t20:1/18:2) | Sphingolipids, SP | Ceramides | 588.5350205 | 7.4885 |
| pos_98 | C38 H70 O3 N1 | Cer(t18:1/20:2) | Sphingolipids, SP | Ceramides | 588.5350205 | 7.6858 |
| pos_101 | C40 H82 O4 N1 | Cer(t18:0/22:0) | Sphingolipids, SP | Ceramides | 640.6238355 | 9.5519 |
| pos_102 | C40 H82 O5 N1 | Cer(t18:0/22:0+O) | Sphingolipids, SP | Ceramides | 656.6187505 | 9.1951 |
| pos_103 | C40 H80 O4 N1 | Cer(t18:1/22:0) | Sphingolipids, SP | Ceramides | 638.6081855 | 9.157 |
| pos_104 | C40 H80 O5 N1 | Cer(t18:1/22:0+O) | Sphingolipids, SP | Ceramides | 654.6031005 | 8.2378 |
| pos_105 | C40 H76 O3 N1 | Cer(t20:1/20:1) | Sphingolipids, SP | Ceramides | 618.5819705 | 9.1086 |
| pos_106 | C40 H70 O3 N1 | Cer(t18:0/22:5) | Sphingolipids, SP | Ceramides | 612.5350205 | 8.441 |
| pos_107 | C41 H84 O4 N1 | Cer(t18:0/23:0) | Sphingolipids, SP | Ceramides | 654.6394855 | 10.0436 |
| pos_108 | C41 H82 O4 N1 | Cer(t18:1/23:0) | Sphingolipids, SP | Ceramides | 652.6238355 | 9.636 |
| pos_109 | C41 H82 O5 N1 | Cer(t18:1/23:0+O) | Sphingolipids, SP | Ceramides | 668.6187505 | 9.278 |
| pos_110 | C42 H86 O4 N1 | Cer(t18:0/24:0) | Sphingolipids, SP | Ceramides | 668.6551355 | 10.526 |
| pos_111 | C42 H86 O5 N1 | Cer(t18:0/24:0+O) | Sphingolipids, SP | Ceramides | 684.6500505 | 10.1755 |
| pos_112 | C42 H84 O4 N1 | Cer(t18:1/24:0) | Sphingolipids, SP | Ceramides | 666.6394855 | 10.1271 |
| pos_113 | C42 H84 O5 N1 | Cer(t18:1/24:0+O) | Sphingolipids, SP | Ceramides | 682.6344005 | 9.2403 |
| pos_114 | C42 H80 O4 N1 | Cer(t18:1/24:2) | Sphingolipids, SP | Ceramides | 662.6081855 | 8.1316 |
| pos_115 | C43 H88 O4 N1 | Cer(t18:0/25:0) | Sphingolipids, SP | Ceramides | 682.6707855 | 10.9845 |
| pos_116 | C43 H86 O4 N1 | Cer(t18:1/25:0) | Sphingolipids, SP | Ceramides | 680.6551355 | 10.6081 |
| pos_117 | C44 H90 O4 N1 | Cer(t18:0/26:0) | Sphingolipids, SP | Ceramides | 696.6864355 | 11.431 |
| pos_118 | C44 H88 O4 N1 | Cer(t18:1/26:0) | Sphingolipids, SP | Ceramides | 694.6707855 | 11.0558 |
| pos_119 | C44 H88 O5 N1 | Cer(t18:1/26:0+O) | Sphingolipids, SP | Ceramides | 710.6657005 | 10.1845 |
| pos_120 | C33 H68 O8 N2 P1 | CerP(d15:1/18:1+2O) | Sphingolipids, SP | Ceramides phosphate | 651.4707825 | 0.871 |
| pos_121 | C27 H45 O0 | ChE(0:0) | Sterol Lipids, ST | Cholesteryl Ester | 369.3515765 | 14.3545 |
| pos_123 | C47 H75 O2 | ChE(20:5) | Sterol Lipids, ST | Cholesteryl Ester | 671.5761565 | 14.3471 |
| pos_124 | C28 H47 O0 | CmE(0:0) | Sterol Lipids, ST | Campesterol | 383.3672265 | 4.6059 |
| pos_126 | C48 H77 O2 | CmE(20:5) | Sterol Lipids, ST | Campesterol | 685.5918065 | 14.5319 |
| pos_127 | C36 H63 O2 | CmE(8:0) | Sterol Lipids, ST | Campesterol | 527.4822565 | 7.8951 |
| pos_128 | C59 H94 O4 N1 | Co(Q10) | Prenol Lipids, PR | Coenzyme | 880.7177355 | 12.4067 |
| pos_129 | C54 H86 O4 N1 | Co(Q9) | Prenol Lipids, PR | Coenzyme | 812.6551355 | 10.9625 |
| pos_130 | C18 H38 O5 N1 | DG(6:0/9:0) | Glycerolipids, GL | Diglyceride | 348.2744505 | 0.6346 |
| pos_131 | C18 H33 O5 | DG(4:0/11:1) | Glycerolipids, GL | Diglyceride | 329.2322515 | 0.6389 |
| pos_132 | C18 H29 O5 | DG(4:0/11:3) | Glycerolipids, GL | Diglyceride | 325.2009515 | 0.854 |
| pos_133 | C19 H40 O5 N1 | DG(6:0/10:0) | Glycerolipids, GL | Diglyceride | 362.2901005 | 1.0611 |
| pos_134 | C21 H41 O5 | DG(6:0/12:0) | Glycerolipids, GL | Diglyceride | 373.2948515 | 0.8659 |
| pos_135 | C21 H39 O5 | DG(6:0/12:1) | Glycerolipids, GL | Diglyceride | 371.2792015 | 0.698 |
| pos_136 | C21 H41 O4 | DG(8:1e/10:0) | Glycerolipids, GL | Diglyceride | 357.2999365 | 1.7877 |
| pos_137 | C21 H37 O5 | DG(6:0/12:2) | Glycerolipids, GL | Diglyceride | 369.2635515 | 0.6164 |
| pos_138 | C21 H39 O4 | DG(8:1e/10:1) | Glycerolipids, GL | Diglyceride | 355.2842865 | 1.3954 |
| pos_139 | C21 H35 O5 | DG(6:0/12:3) | Glycerolipids, GL | Diglyceride | 367.2479015 | 0.814 |
| pos_141 | C24 H41 O5 | DG(10:0/11:3) | Glycerolipids, GL | Diglyceride | 409.2948515 | 1.8866 |
| pos_142 | C24 H39 O5 | DG(9:0/12:4) | Glycerolipids, GL | Diglyceride | 407.2792015 | 1.4379 |
| pos_145 | C33 H68 O5 N2 | DG(16:0/8:0) | Glycerolipids, GL | Diglyceride | 572.5122745 | 7.981 |
| pos_146 | C27 H51 O4 | DG(18:2e/6:0) | Glycerolipids, GL | Diglyceride | 439.3781865 | 4.8676 |
| pos_148 | C27 H52 O4 N1 | DG(18:3e/6:0) | Glycerolipids, GL | Diglyceride | 454.3890855 | 4.1676 |
| pos_149 | C28 H54 O5 Na1 | DG(19:0/6:0) | Glycerolipids, GL | Diglyceride | 493.3863465 | 6.311 |
| pos_150 | C28 H52 O5 Na1 | DG(19:1/6:0) | Glycerolipids, GL | Diglyceride | 491.3706965 | 6.129 |
| pos_151 | C29 H49 O5 | DG(6:0/20:4) | Glycerolipids, GL | Diglyceride | 477.3574515 | 7.346 |
| pos_152 | C30 H53 O5 | DG(16:0/11:3) | Glycerolipids, GL | Diglyceride | 493.3887515 | 3.6859 |
| pos_153 | C30 H51 O5 | DG(16:0/11:4) | Glycerolipids, GL | Diglyceride | 491.3731015 | 3.297 |
| pos_154 | C30 H51 O5 | DG(18:4/9:0) | Glycerolipids, GL | Diglyceride | 491.3731015 | 4.1944 |
| pos_155 | C31 H58 O5 Na1 | DG(6:0/22:1) | Glycerolipids, GL | Diglyceride | 533.4176465 | 3.9183 |
| pos_156 | C31 H55 O5 | DG(16:0/12:3) | Glycerolipids, GL | Diglyceride | 507.4044015 | 3.8769 |
| pos_158 | C31 H53 O5 | DG(18:3/10:1) | Glycerolipids, GL | Diglyceride | 505.3887515 | 5.853 |
| pos_160 | C31 H50 O4 Li1 | DG(18:3e/10:3) | Glycerolipids, GL | Diglyceride | 493.3863615 | 8.065 |
| pos_161 | C32 H66 O5 N1 | DG(15:0/14:0) | Glycerolipids, GL | Diglyceride | 544.4935505 | 5.4028 |
| pos_162 | C32 H55 O5 | DG(18:3/11:1) | Glycerolipids, GL | Diglyceride | 519.4044015 | 7.3599 |
| pos_163 | C32 H55 O5 | DG(18:1/11:3) | Glycerolipids, GL | Diglyceride | 519.4044015 | 9.609 |
| pos_164 | C32 H55 O4 | DG(18:3e/11:2) | Glycerolipids, GL | Diglyceride | 503.4094865 | 8.124 |
| pos_165 | C33 H64 O5 K1 | DG(6:0/24:0) | Glycerolipids, GL | Diglyceride | 579.4385345 | 5.7878 |
| pos_166 | C33 H64 O5 Na1 | DG(16:0/14:0) | Glycerolipids, GL | Diglyceride | 563.4645965 | 7.826 |
| pos_168 | C33 H57 O5 | DG(18:3/12:1) | Glycerolipids, GL | Diglyceride | 533.4200515 | 5.5791 |
| pos_169 | C33 H57 O5 | DG(20:3/10:1) | Glycerolipids, GL | Diglyceride | 533.4200515 | 7.351 |
| pos_170 | C33 H55 O5 | DG(18:2/12:3) | Glycerolipids, GL | Diglyceride | 531.4044015 | 3.2763 |
| pos_172 | C34 H66 O5 Na1 | DG(25:0/6:0) | Glycerolipids, GL | Diglyceride | 577.4802465 | 4.5133 |
| pos_173 | C34 H66 O5 Na1 | DG(15:0/16:0) | Glycerolipids, GL | Diglyceride | 577.4802465 | 5.981 |
| pos_174 | C34 H68 O5 N1 | DG(25:1/6:0) | Glycerolipids, GL | Diglyceride | 570.5092005 | 2.018 |
| pos_175 | C34 H68 O5 N1 | DG(15:0/16:1) | Glycerolipids, GL | Diglyceride | 570.5092005 | 5.271 |
| pos_176 | C34 H66 O4 Na1 | DG(20:0e/11:1) | Glycerolipids, GL | Diglyceride | 561.4853315 | 7.091 |
| pos_177 | C34 H63 O5 | DG(18:2/13:0) | Glycerolipids, GL | Diglyceride | 551.4670015 | 3.8941 |
| pos_179 | C34 H64 O4 Na1 | DG(20:0e/11:2) | Glycerolipids, GL | Diglyceride | 559.4696815 | 6.388 |
| pos_180 | C34 H64 O4 Na1 | DG(20:1e/11:1) | Glycerolipids, GL | Diglyceride | 559.4696815 | 6.956 |
| pos_182 | C34 H62 O4 Na1 | DG(20:2e/11:1) | Glycerolipids, GL | Diglyceride | 557.4540315 | 4.3375 |
| pos_185 | C35 H68 O5 Na1 | DG(16:0/16:0) | Glycerolipids, GL | Diglyceride | 591.4958965 | 4.3179 |
| pos_186 | C35 H66 O5 Na1 | DG(16:0/16:1) | Glycerolipids, GL | Diglyceride | 589.4802465 | 5.256 |
| pos_187 | C35 H70 O5 N1 | DG(26:1/6:0) | Glycerolipids, GL | Diglyceride | 584.5248505 | 5.553 |
| pos_188 | C35 H70 O5 N1 | DG(18:1/14:0) | Glycerolipids, GL | Diglyceride | 584.5248505 | 7.8392 |
| pos_189 | C35 H68 O4 Na1 | DG(16:1e/16:0) | Glycerolipids, GL | Diglyceride | 575.5009815 | 6.0641 |
| pos_190 | C35 H68 O5 N1 | DG(14:0/18:2) | Glycerolipids, GL | Diglyceride | 582.5092005 | 7.0732 |
| pos_191 | C35 H66 O4 Na1 | DG(16:1e/16:1) | Glycerolipids, GL | Diglyceride | 573.4853315 | 5.47 |
| pos_193 | C35 H63 O5 | DG(14:0/18:3) | Glycerolipids, GL | Diglyceride | 563.4670015 | 4.8158 |
| pos_194 | C36 H74 O5 N1 | DG(27:0/6:0) | Glycerolipids, GL | Diglyceride | 600.5561505 | 7.112 |
| pos_195 | C36 H72 O5 N1 | DG(15:0/18:1) | Glycerolipids, GL | Diglyceride | 598.5405005 | 6.1497 |
| pos_196 | C36 H70 O5 N1 | DG(15:0/18:2) | Glycerolipids, GL | Diglyceride | 596.5248505 | 5.4936 |
| pos_197 | C36 H65 O5 | DG(15:0/18:3) | Glycerolipids, GL | Diglyceride | 577.4826515 | 3.5039 |
| pos_198 | C36 H63 O5 | DG(18:4/15:0) | Glycerolipids, GL | Diglyceride | 575.4670015 | 2.918 |
| pos_199 | C36 H63 O5 | DG(23:1/10:3) | Glycerolipids, GL | Diglyceride | 575.4670015 | 4.9181 |
| pos_200 | C36 H61 O5 | DG(22:4/11:1) | Glycerolipids, GL | Diglyceride | 573.4513515 | 4.1369 |
| pos_201 | C36 H64 O5 N1 | DG(19:1/14:4) | Glycerolipids, GL | Diglyceride | 590.4779005 | 4.721 |
| pos_202 | C36 H59 O5 | DG(22:3/11:3) | Glycerolipids, GL | Diglyceride | 571.4357015 | 3.1879 |
| pos_203 | C37 H76 O5 N1 | DG(18:0/16:0) | Glycerolipids, GL | Diglyceride | 614.5718005 | 9.7907 |
| pos_205 | C37 H70 O5 Na1 | DG(28:1/6:0) | Glycerolipids, GL | Diglyceride | 617.5115465 | 4.919 |
| pos_206 | C37 H70 O5 Na1 | DG(24:0/10:1) | Glycerolipids, GL | Diglyceride | 617.5115465 | 7.359 |
| pos_208 | C37 H68 O5 Na1 | DG(17:1/17:1) | Glycerolipids, GL | Diglyceride | 615.4958965 | 11.298 |
| pos_209 | C37 H70 O4 Na1 | DG(16:1e/18:1) | Glycerolipids, GL | Diglyceride | 601.5166315 | 6.839 |
| pos_210 | C37 H67 O5 | DG(16:0/18:3) | Glycerolipids, GL | Diglyceride | 591.4983015 | 4.0393 |
| pos_211 | C37 H70 O5 N1 | DG(16:1/18:2) | Glycerolipids, GL | Diglyceride | 608.5248505 | 7.167 |
| pos_212 | C37 H68 O4 Na1 | DG(16:2e/18:1) | Glycerolipids, GL | Diglyceride | 599.5009815 | 6.1269 |
| pos_213 | C37 H68 O4 Na1 | DG(16:1e/18:2) | Glycerolipids, GL | Diglyceride | 599.5009815 | 7.363 |
| pos_214 | C37 H65 O5 | DG(18:4/16:0) | Glycerolipids, GL | Diglyceride | 589.4826515 | 4.4583 |
| pos_215 | C37 H65 O5 | DG(16:1/18:3) | Glycerolipids, GL | Diglyceride | 589.4826515 | 4.9299 |
| pos_217 | C37 H66 O4 Na1 | DG(16:2e/18:2) | Glycerolipids, GL | Diglyceride | 597.4853315 | 5.2838 |
| pos_219 | C37 H65 O4 | DG(16:1e/18:4) | Glycerolipids, GL | Diglyceride | 573.4877365 | 4.287 |
| pos_221 | C38 H76 O5 N1 | DG(15:0/20:1) | Glycerolipids, GL | Diglyceride | 626.5718005 | 7.243 |
| pos_223 | C38 H74 O5 N1 | DG(17:1/18:1) | Glycerolipids, GL | Diglyceride | 624.5561505 | 8.0162 |
| pos_225 | C38 H72 O5 N1 | DG(17:1/18:2) | Glycerolipids, GL | Diglyceride | 622.5405005 | 7.6254 |
| pos_226 | C39 H80 O5 N1 | DG(18:0/18:0) | Glycerolipids, GL | Diglyceride | 642.6031005 | 10.7076 |
| pos_227 | C39 H78 O5 N1 | DG(18:0/18:1) | Glycerolipids, GL | Diglyceride | 640.5874505 | 9.8027 |
| pos_228 | C39 H74 O5 Na1 | DG(30:1/6:0) | Glycerolipids, GL | Diglyceride | 645.5428465 | 12.763 |
| pos_229 | C39 H76 O4 Na1 | DG(16:1e/20:0) | Glycerolipids, GL | Diglyceride | 631.5635815 | 10.038 |
| pos_231 | C39 H73 O5 | DG(18:0/18:2) | Glycerolipids, GL | Diglyceride | 621.5452515 | 7.4292 |
| pos_232 | C39 H74 O4 Na1 | DG(16:1e/20:1) | Glycerolipids, GL | Diglyceride | 629.5479315 | 9.048 |
| pos_234 | C39 H71 O5 | DG(18:0/18:3) | Glycerolipids, GL | Diglyceride | 619.5296015 | 6.517 |
| pos_235 | C39 H72 O4 Na1 | DG(16:1e/20:2) | Glycerolipids, GL | Diglyceride | 627.5322815 | 8.278 |
| pos_237 | C39 H72 O5 N1 | DG(18:2/18:2) | Glycerolipids, GL | Diglyceride | 634.5405005 | 7.3473 |
| pos_238 | C39 H71 O4 | DG(18:3e/18:1) | Glycerolipids, GL | Diglyceride | 603.5346865 | 7.1342 |
| pos_239 | C39 H74 O4 N1 | DG(18:2e/18:2) | Glycerolipids, GL | Diglyceride | 620.5612355 | 9.7085 |
| pos_240 | C39 H67 O5 | DG(18:3/18:2) | Glycerolipids, GL | Diglyceride | 615.4983015 | 3.736 |
| pos_241 | C39 H69 O4 | DG(18:3e/18:2) | Glycerolipids, GL | Diglyceride | 601.5190365 | 6.165 |
| pos_242 | C39 H65 O5 | DG(18:4/18:2) | Glycerolipids, GL | Diglyceride | 613.4826515 | 1.851 |
| pos_244 | C39 H67 O4 | DG(18:3e/18:3) | Glycerolipids, GL | Diglyceride | 599.5033865 | 4.7582 |
| pos_246 | C39 H60 O5 Li1 | DG(18:4/18:4) | Glycerolipids, GL | Diglyceride | 615.4595265 | 3.8722 |
| pos_247 | C40 H80 O5 N1 | DG(19:0/18:1) | Glycerolipids, GL | Diglyceride | 654.6031005 | 9.799 |
| pos_248 | C40 H78 O5 N1 | DG(19:1/18:1) | Glycerolipids, GL | Diglyceride | 652.5874505 | 8.8896 |
| pos_249 | C40 H72 O5 Li1 | DG(19:1/18:2) | Glycerolipids, GL | Diglyceride | 639.5534265 | 7.763 |
| pos_250 | C40 H74 O5 N1 | DG(19:1/18:3) | Glycerolipids, GL | Diglyceride | 648.5561505 | 7.595 |
| pos_252 | C41 H80 O5 N1 | DG(20:1/18:1) | Glycerolipids, GL | Diglyceride | 666.6031005 | 9.7604 |
| pos_254 | C41 H75 O5 | DG(18:1/20:2) | Glycerolipids, GL | Diglyceride | 647.5609015 | 7.5819 |
| pos_256 | C41 H73 O5 | DG(20:1/18:3) | Glycerolipids, GL | Diglyceride | 645.5452515 | 6.5271 |
| pos_258 | C41 H75 O4 | DG(20:3e/18:1) | Glycerolipids, GL | Diglyceride | 631.5659865 | 14.364 |
| pos_260 | C41 H73 O4 | DG(20:3e/18:2) | Glycerolipids, GL | Diglyceride | 629.5503365 | 9.0479 |
| pos_261 | C47 H84 O5 N2 | DG(16:0/22:6) | Glycerolipids, GL | Diglyceride | 756.6374745 | 9.084 |
| pos_262 | C41 H74 O4 N1 | DG(20:4e/18:2) | Glycerolipids, GL | Diglyceride | 644.5612355 | 4.5496 |
| pos_264 | C43 H88 O5 N1 | DG(16:0/24:0) | Glycerolipids, GL | Diglyceride | 698.6657005 | 4.344 |
| pos_265 | C43 H86 O5 N1 | DG(18:1/22:0) | Glycerolipids, GL | Diglyceride | 696.6500505 | 11.6338 |
| pos_266 | C43 H84 O5 N1 | DG(18:1/22:1) | Glycerolipids, GL | Diglyceride | 694.6344005 | 10.6141 |
| pos_267 | C43 H84 O5 N1 | DG(22:0/18:2) | Glycerolipids, GL | Diglyceride | 694.6344005 | 11.0125 |
| pos_268 | C43 H79 O5 | DG(22:1/18:2) | Glycerolipids, GL | Diglyceride | 675.5922015 | 8.671 |
| pos_270 | C49 H86 O5 N2 | DG(18:1/22:6) | Glycerolipids, GL | Diglyceride | 782.6531245 | 9.191 |
| pos_272 | C43 H66 O5 Na1 | DG(18:3/22:6) | Glycerolipids, GL | Diglyceride | 685.4802465 | 4.46 |
| pos_273 | C45 H90 O5 N1 | DG(18:1/24:0) | Glycerolipids, GL | Diglyceride | 724.6813505 | 12.4205 |
| pos_274 | C45 H88 O5 N1 | DG(18:1/24:1) | Glycerolipids, GL | Diglyceride | 722.6657005 | 11.4569 |
| pos_275 | C45 H88 O5 N1 | DG(24:0/18:2) | Glycerolipids, GL | Diglyceride | 722.6657005 | 11.8682 |
| pos_276 | C45 H86 O5 N1 | DG(24:1/18:2) | Glycerolipids, GL | Diglyceride | 720.6500505 | 10.8406 |
| pos_281 | C56 H110 O5 Li1 | DG(37:0/16:0) | Glycerolipids, GL | Diglyceride | 869.8507765 | 18.183 |
| pos_284 | C56 H110 O5 N1 | DG(35:0/18:2) | Glycerolipids, GL | Diglyceride | 876.8378505 | 12.85 |
| pos_285 | C56 H104 O5 K1 | DG(35:1/18:2) | Glycerolipids, GL | Diglyceride | 895.7515345 | 9.671 |
| pos_286 | C56 H104 O5 K1 | DG(35:0/18:3) | Glycerolipids, GL | Diglyceride | 895.7515345 | 9.8798 |
| pos_287 | C57 H108 O5 Na1 | DG(36:0/18:2) | Glycerolipids, GL | Diglyceride | 895.8088965 | 12.517 |
| pos_289 | C57 H106 O5 K1 | DG(36:0/18:3) | Glycerolipids, GL | Diglyceride | 909.7671845 | 12.181 |
| pos_291 | C57 H104 O5 Na1 | DG(36:1/18:3) | Glycerolipids, GL | Diglyceride | 891.7775965 | 11.317 |
| pos_294 | C59 H112 O5 K1 | DG(38:1/18:1) | Glycerolipids, GL | Diglyceride | 939.8141345 | 13.1005 |
| pos_295 | C59 H110 O5 K1 | DG(38:0/18:3) | Glycerolipids, GL | Diglyceride | 937.7984845 | 12.6437 |
| pos_297 | C61 H114 O5 Na1 | DG(38:1/20:2) | Glycerolipids, GL | Diglyceride | 949.8558465 | 13.745 |
| pos_298 | C33 H62 O14 Na1 | DGDG(8:0e/10:0) | Saccharolipids, SL | Digalactosyldiacylglycerol | 705.4031815 | 2.5237 |
| pos_299 | C33 H60 O14 Na1 | DGDG(8:0e/10:1) | Saccharolipids, SL | Digalactosyldiacylglycerol | 703.3875315 | 1.6632 |
| pos_300 | C33 H58 O14 Na1 | DGDG(8:0e/10:2) | Saccharolipids, SL | Digalactosyldiacylglycerol | 701.3718815 | 1.343 |
| pos_301 | C33 H56 O14 Na1 | DGDG(8:0e/10:3) | Saccharolipids, SL | Digalactosyldiacylglycerol | 699.3562315 | 1.0619 |
| pos_302 | C35 H61 O14 | DGDG(8:0e/12:3) | Saccharolipids, SL | Digalactosyldiacylglycerol | 705.4055865 | 2.4965 |
| pos_303 | C35 H59 O14 | DGDG(8:0e/12:4) | Saccharolipids, SL | Digalactosyldiacylglycerol | 703.3899365 | 1.8074 |
| pos_304 | C35 H57 O14 | DGDG(8:0e/12:5) | Saccharolipids, SL | Digalactosyldiacylglycerol | 701.3742865 | 1.3366 |
| pos_305 | C35 H55 O14 | DGDG(8:1e/12:5) | Saccharolipids, SL | Digalactosyldiacylglycerol | 699.3586365 | 1.0749 |
| pos_306 | C47 H88 O15 Na1 | DGDG(16:0/16:0) | Saccharolipids, SL | Digalactosyldiacylglycerol | 915.6015465 | 7.0256 |
| pos_307 | C47 H86 O15 Na1 | DGDG(18:1/14:0) | Saccharolipids, SL | Digalactosyldiacylglycerol | 913.5858965 | 6.2068 |
| pos_308 | C47 H84 O15 Na1 | DGDG(18:2/14:0) | Saccharolipids, SL | Digalactosyldiacylglycerol | 911.5702465 | 5.5373 |
| pos_309 | C49 H92 O15 Na1 | DGDG(16:0/18:0) | Saccharolipids, SL | Digalactosyldiacylglycerol | 943.6328465 | 8.0039 |
| pos_310 | C49 H90 O15 Na1 | DGDG(16:0/18:1) | Saccharolipids, SL | Digalactosyldiacylglycerol | 941.6171965 | 7.1104 |
| pos_311 | C49 H88 O15 Na1 | DGDG(16:0/18:2) | Saccharolipids, SL | Digalactosyldiacylglycerol | 939.6015465 | 5.479 |
| pos_312 | C49 H86 O15 Na1 | DGDG(16:1/18:2) | Saccharolipids, SL | Digalactosyldiacylglycerol | 937.5858965 | 5.6308 |
| pos_313 | C49 H86 O15 Na1 | DGDG(16:0/18:3) | Saccharolipids, SL | Digalactosyldiacylglycerol | 937.5858965 | 5.8955 |
| pos_314 | C50 H90 O15 Na1 | DGDG(17:0/18:2) | Saccharolipids, SL | Digalactosyldiacylglycerol | 953.6171965 | 6.9161 |
| pos_315 | C50 H92 O15 N1 | DGDG(17:1/18:2) | Saccharolipids, SL | Digalactosyldiacylglycerol | 946.6461505 | 6.0561 |
| pos_316 | C51 H94 O15 Na1 | DGDG(18:0/18:1) | Saccharolipids, SL | Digalactosyldiacylglycerol | 969.6484965 | 8.0605 |
| pos_317 | C51 H92 O15 Na1 | DGDG(18:1/18:1) | Saccharolipids, SL | Digalactosyldiacylglycerol | 967.6328465 | 7.1864 |
| pos_318 | C51 H92 O15 Na1 | DGDG(18:0/18:2) | Saccharolipids, SL | Digalactosyldiacylglycerol | 967.6328465 | 7.3886 |
| pos_319 | C51 H90 O15 Na1 | DGDG(18:1/18:2) | Saccharolipids, SL | Digalactosyldiacylglycerol | 965.6171965 | 6.5049 |
| pos_320 | C51 H88 O15 Na1 | DGDG(18:2/18:2) | Saccharolipids, SL | Digalactosyldiacylglycerol | 963.6015465 | 4.9993 |
| pos_321 | C51 H86 O15 Na1 | DGDG(18:2/18:3) | Saccharolipids, SL | Digalactosyldiacylglycerol | 961.5858965 | 5.3011 |
| pos_322 | C51 H84 O15 Na1 | DGDG(18:3/18:3) | Saccharolipids, SL | Digalactosyldiacylglycerol | 959.5702465 | 4.807 |
| pos_323 | C52 H98 O15 N1 | DGDG(18:1/19:1) | Saccharolipids, SL | Digalactosyldiacylglycerol | 976.6931005 | 7.1813 |
| pos_324 | C53 H100 O15 N1 | DGDG(18:1/20:1) | Saccharolipids, SL | Digalactosyldiacylglycerol | 990.7087505 | 8.0233 |
| pos_325 | C53 H98 O15 N1 | DGDG(20:1/18:2) | Saccharolipids, SL | Digalactosyldiacylglycerol | 988.6931005 | 7.3414 |
| pos_326 | C53 H96 O15 N1 | DGDG(18:2/20:2) | Saccharolipids, SL | Digalactosyldiacylglycerol | 986.6774505 | 6.6209 |
| pos_327 | C54 H101 O22 N2 | GM3(d13:0/18:0+O) | Sphingolipids, SP | Ganglioside | 1129.684054 | 6.8507 |
| pos_328 | C36 H73 O8 N2 | Hex1Cer(d12:0/18:1) | Sphingolipids, SP | Hexosylceramide | 661.5361445 | 7.547 |
| pos_330 | C42 H80 O8 N1 | Hex1Cer(d18:2/18:0) | Sphingolipids, SP | Hexosylceramide | 726.5878455 | 6.9107 |
| pos_331 | C42 H80 O9 N1 | Hex1Cer(d18:2/18:0+O) | Sphingolipids, SP | Hexosylceramide | 742.5827605 | 6.5878 |
| pos_332 | C44 H86 O9 N1 | Hex1Cer(d18:1/20:0+O) | Sphingolipids, SP | Hexosylceramide | 772.6297105 | 7.9376 |
| pos_333 | C44 H84 O8 N1 | Hex1Cer(d18:2/20:0) | Sphingolipids, SP | Hexosylceramide | 754.6191455 | 7.9143 |
| pos_334 | C44 H84 O8 N1 | Hex1Cer(d18:1/20:1) | Sphingolipids, SP | Hexosylceramide | 754.6191455 | 8.512 |
| pos_335 | C44 H84 O9 N1 | Hex1Cer(d18:2/20:0+O) | Sphingolipids, SP | Hexosylceramide | 770.6140605 | 7.5875 |
| pos_336 | C44 H82 O8 N1 | Hex1Cer(d18:2/20:1) | Sphingolipids, SP | Hexosylceramide | 752.6034955 | 7.5898 |
| pos_337 | C46 H88 O10 N1 | Hex1Cer(d22:2/18:0+2O) | Sphingolipids, SP | Hexosylceramide | 814.6402755 | 7.206 |
| pos_338 | C46 H88 O9 N1 | Hex1Cer(d18:2/22:0+O) | Sphingolipids, SP | Hexosylceramide | 798.6453605 | 8.6047 |
| pos_339 | C46 H86 O8 N1 | Hex1Cer(d18:2/22:1) | Sphingolipids, SP | Hexosylceramide | 780.6347955 | 8.6149 |
| pos_340 | C48 H94 O10 N1 | Hex1Cer(d24:1/18:0+2O) | Sphingolipids, SP | Hexosylceramide | 844.6872255 | 8.5436 |
| pos_341 | C48 H92 O8 N1 | Hex1Cer(d18:1/24:1) | Sphingolipids, SP | Hexosylceramide | 810.6817455 | 10.0316 |
| pos_342 | C48 H92 O10 N1 | Hex1Cer(d24:2/18:0+2O) | Sphingolipids, SP | Hexosylceramide | 842.6715755 | 8.1406 |
| pos_343 | C48 H92 O9 N1 | Hex1Cer(d18:2/24:0+O) | Sphingolipids, SP | Hexosylceramide | 826.6766605 | 9.6012 |
| pos_344 | C48 H90 O8 N1 | Hex1Cer(d18:2/24:1) | Sphingolipids, SP | Hexosylceramide | 808.6660955 | 9.191 |
| pos_345 | C48 H88 O8 N1 | Hex1Cer(d18:2/24:2) | Sphingolipids, SP | Hexosylceramide | 806.6504455 | 8.215 |
| pos_346 | C42 H80 O8 N1 | Hex1Cer(t18:0/18:1) | Sphingolipids, SP | Hexosylceramide | 726.5878455 | 7.073 |
| pos_347 | C42 H78 O8 N1 | Hex1Cer(t18:1/18:1) | Sphingolipids, SP | Hexosylceramide | 724.5721955 | 6.5878 |
| pos_348 | C44 H84 O8 N1 | Hex1Cer(t18:0/20:1) | Sphingolipids, SP | Hexosylceramide | 754.6191455 | 7.9221 |
| pos_349 | C44 H86 O10 N1 | Hex1Cer(t18:1/20:0+O) | Sphingolipids, SP | Hexosylceramide | 788.6246255 | 7.2403 |
| pos_350 | C44 H82 O8 N1 | Hex1Cer(t18:1/20:1) | Sphingolipids, SP | Hexosylceramide | 752.6034955 | 7.5906 |
| pos_351 | C44 H80 O8 N1 | Hex1Cer(t18:1/20:2) | Sphingolipids, SP | Hexosylceramide | 750.5878455 | 6.645 |
| pos_352 | C46 H90 O10 N1 | Hex1Cer(t18:1/22:0+O) | Sphingolipids, SP | Hexosylceramide | 816.6559255 | 8.2398 |
| pos_353 | C46 H86 O8 N1 | Hex1Cer(t18:1/22:1) | Sphingolipids, SP | Hexosylceramide | 780.6347955 | 8.6096 |
| pos_354 | C46 H88 O10 N1 | Hex1Cer(t20:1/20:1+O) | Sphingolipids, SP | Hexosylceramide | 814.6402755 | 7.2196 |
| pos_355 | C48 H92 O8 N1 | Hex1Cer(t18:0/24:1) | Sphingolipids, SP | Hexosylceramide | 810.6817455 | 10.0454 |
| pos_356 | C48 H94 O10 N1 | Hex1Cer(t18:1/24:0+O) | Sphingolipids, SP | Hexosylceramide | 844.6872255 | 8.545 |
| pos_357 | C48 H90 O8 N1 | Hex1Cer(t18:1/24:1) | Sphingolipids, SP | Hexosylceramide | 808.6660955 | 9.191 |
| pos_358 | C48 H88 O8 N1 | Hex1Cer(t18:1/24:2) | Sphingolipids, SP | Hexosylceramide | 806.6504455 | 8.4793 |
| pos_359 | C50 H98 O10 N1 | Hex1Cer(t18:1/26:0+O) | Sphingolipids, SP | Hexosylceramide | 872.7185255 | 10.1851 |
| pos_360 | C24 H48 O8 N1 | Hex1SPH(t18:1) | Sphingolipids, SP | Hexosylsphingosine | 478.3374455 | 0.9017 |
| pos_361 | C38 H76 O10 P1 | LBPA(16:0/16:0) | Glycerophospholipids, GP | Lysobisphosphatidic acid | 723.5170645 | 6.1973 |
| pos_362 | C40 H78 O10 P1 | LBPA(16:0/18:1) | Glycerophospholipids, GP | Lysobisphosphatidic acid | 749.5327145 | 6.2623 |
| pos_363 | C40 H79 O10 P1 N1 | LBPA(16:0/18:2) | Glycerophospholipids, GP | Lysobisphosphatidic acid | 764.5436135 | 5.6606 |
| pos_364 | C42 H81 O10 P1 N1 | LBPA(18:1/18:2) | Glycerophospholipids, GP | Lysobisphosphatidic acid | 790.5592635 | 5.7378 |
| pos_365 | C42 H79 O10 P1 N1 | LBPA(18:2/18:2) | Glycerophospholipids, GP | Lysobisphosphatidic acid | 788.5436135 | 5.179 |
| pos_366 | C22 H47 O7 N1 P1 | LPC(14:0) | Glycerophospholipids, GP | Lyso-phosphatidylcholine | 468.3084685 | 1.1917 |
| pos_367 | C23 H49 O7 N1 P1 | LPC(15:0) | Glycerophospholipids, GP | Lyso-phosphatidylcholine | 482.3241185 | 1.4651 |
| pos_368 | C24 H50 O7 N1 P1 Na1 | LPC(16:0) | Glycerophospholipids, GP | Lyso-phosphatidylcholine | 518.3217135 | 1.6412 |
| pos_369 | C24 H49 O7 N1 P1 | LPC(16:1) | Glycerophospholipids, GP | Lyso-phosphatidylcholine | 494.3241185 | 1.2782 |
| pos_370 | C24 H50 O6 N1 P1 Na1 | LPC(16:1e) | Glycerophospholipids, GP | Lyso-phosphatidylcholine | 502.3267985 | 1.4294 |
| pos_372 | C25 H53 O7 N1 P1 | LPC(17:0) | Glycerophospholipids, GP | Lyso-phosphatidylcholine | 510.3554185 | 2.1905 |
| pos_373 | C25 H51 O7 N1 P1 | LPC(17:1) | Glycerophospholipids, GP | Lyso-phosphatidylcholine | 508.3397685 | 1.5597 |
| pos_375 | C26 H52 O7 N1 P1 Na1 | LPC(18:1) | Glycerophospholipids, GP | Lyso-phosphatidylcholine | 544.3373635 | 1.7517 |
| pos_376 | C26 H51 O7 N1 P1 | LPC(18:2) | Glycerophospholipids, GP | Lyso-phosphatidylcholine | 520.3397685 | 1.4315 |
| pos_377 | C26 H53 O6 N1 P1 | LPC(18:2e) | Glycerophospholipids, GP | Lyso-phosphatidylcholine | 506.3605035 | 2.621 |
| pos_378 | C26 H49 O7 N1 P1 | LPC(18:3) | Glycerophospholipids, GP | Lyso-phosphatidylcholine | 518.3241185 | 0.7719 |
| pos_379 | C26 H51 O6 N1 P1 | LPC(18:3e) | Glycerophospholipids, GP | Lyso-phosphatidylcholine | 504.3448535 | 1.8872 |
| pos_380 | C28 H59 O7 N1 P1 | LPC(20:0) | Glycerophospholipids, GP | Lyso-phosphatidylcholine | 552.4023685 | 3.697 |
| pos_381 | C28 H57 O7 N1 P1 | LPC(20:1) | Glycerophospholipids, GP | Lyso-phosphatidylcholine | 550.3867185 | 2.5286 |
| pos_382 | C28 H55 O7 N1 P1 | LPC(20:2) | Glycerophospholipids, GP | Lyso-phosphatidylcholine | 548.3710685 | 2.0473 |
| pos_383 | C28 H53 O7 N1 P1 | LPC(20:3) | Glycerophospholipids, GP | Lyso-phosphatidylcholine | 546.3554185 | 2.6205 |
| pos_384 | C28 H51 O7 N1 P1 | LPC(20:4) | Glycerophospholipids, GP | Lyso-phosphatidylcholine | 544.3397685 | 1.8738 |
| pos_385 | C28 H49 O7 N1 P1 | LPC(20:5) | Glycerophospholipids, GP | Lyso-phosphatidylcholine | 542.3241185 | 1.4154 |
| pos_386 | C30 H63 O7 N1 P1 | LPC(22:0) | Glycerophospholipids, GP | Lyso-phosphatidylcholine | 580.4336685 | 4.754 |
| pos_387 | C30 H55 O7 N1 P1 | LPC(22:4) | Glycerophospholipids, GP | Lyso-phosphatidylcholine | 572.3710685 | 2.7037 |
| pos_388 | C32 H67 O7 N1 P1 | LPC(24:0) | Glycerophospholipids, GP | Lyso-phosphatidylcholine | 608.4649685 | 5.8059 |
| pos_389 | C34 H71 O7 N1 P1 | LPC(26:0) | Glycerophospholipids, GP | Lyso-phosphatidylcholine | 636.4962685 | 7.0049 |
| pos_390 | C21 H44 O7 N1 P1 Na1 | LPE(16:0) | Glycerophospholipids, GP | Lyso-phosphatidylethanolamine | 476.2747635 | 1.6634 |
| pos_391 | C23 H49 O7 N1 P1 | LPE(18:0) | Glycerophospholipids, GP | Lyso-phosphatidylethanolamine | 482.3241185 | 2.6367 |
| pos_393 | C23 H44 O7 N1 P1 Na1 | LPE(18:2) | Glycerophospholipids, GP | Lyso-phosphatidylethanolamine | 500.2747635 | 1.4379 |
| pos_394 | C23 H43 O7 N1 P1 | LPE(18:3) | Glycerophospholipids, GP | Lyso-phosphatidylethanolamine | 476.2771685 | 1.1306 |
| pos_395 | C27 H57 O7 N1 P1 | LPE(22:0) | Glycerophospholipids, GP | Lyso-phosphatidylethanolamine | 538.3867185 | 4.702 |
| pos_396 | C22 H45 O9 N0 P1 Na1 | LPG(16:0) | Glycerophospholipids, GP | Lyso-phosphatidylglycerol | 507.2693445 | 1.5978 |
| pos_397 | C24 H47 O9 N0 P1 Na1 | LPG(18:1) | Glycerophospholipids, GP | Lyso-phosphatidylglycerol | 533.2849945 | 1.691 |
| pos_398 | C24 H45 O9 N0 P1 Na1 | LPG(18:2) | Glycerophospholipids, GP | Lyso-phosphatidylglycerol | 531.2693445 | 1.2697 |
| pos_399 | C20 H41 O7 N0 P1 Na1 | LPMe(16:0) | Glycerophospholipids, GP | Lyso-phosphatidylmethanol | 447.2482145 | 1.7635 |
| pos_400 | C22 H45 O7 N0 P1 Na1 | LPMe(18:0) | Glycerophospholipids, GP | Lyso-phosphatidylmethanol | 475.2795145 | 2.5818 |
| pos_401 | C22 H43 O7 N0 P1 Na1 | LPMe(18:1) | Glycerophospholipids, GP | Lyso-phosphatidylmethanol | 473.2638645 | 1.866 |
| pos_402 | C25 H51 O7 N1 P1 | LdMePE(18:1) | Glycerophospholipids, GP | Lysodimethylphosphatidylethanolamine | 508.3397685 | 1.5735 |
| pos_403 | C25 H49 O7 N1 P1 | LdMePE(18:2) | Glycerophospholipids, GP | Lysodimethylphosphatidylethanolamine | 506.3241185 | 1.1962 |
| pos_404 | C18 H40 O4 N1 | MG(15:0) | Glycerolipids, GL | Monoglyceride | 334.2951855 | 0.976 |
| pos_405 | C19 H39 O4 | MG(16:0) | Glycerolipids, GL | Monoglyceride | 331.2842865 | 1.6885 |
| pos_406 | C19 H37 O4 | MG(16:1) | Glycerolipids, GL | Monoglyceride | 329.2686365 | 1.0955 |
| pos_407 | C21 H41 O4 | MG(18:1) | Glycerolipids, GL | Monoglyceride | 357.2999365 | 1.661 |
| pos_408 | C21 H39 O4 | MG(18:2) | Glycerolipids, GL | Monoglyceride | 355.2842865 | 0.878 |
| pos_409 | C21 H37 O4 | MG(18:3) | Glycerolipids, GL | Monoglyceride | 353.2686365 | 0.949 |
| pos_410 | C21 H35 O4 | MG(18:4) | Glycerolipids, GL | Monoglyceride | 351.2529865 | 0.8195 |
| pos_411 | C23 H43 O4 | MG(20:2) | Glycerolipids, GL | Monoglyceride | 383.3155865 | 1.9403 |
| pos_412 | C23 H39 O4 | MG(20:4) | Glycerolipids, GL | Monoglyceride | 379.2842865 | 7.96 |
| pos_413 | C23 H41 O3 | MG(20:4e) | Glycerolipids, GL | Monoglyceride | 365.3050215 | 9.021 |
| pos_414 | C23 H37 O4 | MG(20:5) | Glycerolipids, GL | Monoglyceride | 377.2686365 | 8.142 |
| pos_415 | C31 H64 O4 N2 | MG(22:1) | Glycerolipids, GL | Monoglyceride | 528.4860595 | 7.897 |
| pos_418 | C41 H82 O10 N1 | MGDG(16:0/16:0) | Saccharolipids, SL | Monogalactosyldiacylglycerol | 748.5933255 | 7.6615 |
| pos_419 | C43 H80 O10 Na1 | MGDG(16:0/18:1) | Saccharolipids, SL | Monogalactosyldiacylglycerol | 779.5643715 | 7.7286 |
| pos_420 | C43 H78 O10 Na1 | MGDG(16:0/18:2) | Saccharolipids, SL | Monogalactosyldiacylglycerol | 777.5487215 | 7.0108 |
| pos_421 | C45 H88 O10 N1 | MGDG(18:0/18:1) | Saccharolipids, SL | Monogalactosyldiacylglycerol | 802.6402755 | 8.7053 |
| pos_422 | C45 H82 O10 Na1 | MGDG(18:1/18:1) | Saccharolipids, SL | Monogalactosyldiacylglycerol | 805.5800215 | 7.521 |
| pos_423 | C45 H80 O10 Na1 | MGDG(18:1/18:2) | Saccharolipids, SL | Monogalactosyldiacylglycerol | 803.5643715 | 6.863 |
| pos_424 | C45 H78 O10 Na1 | MGDG(18:2/18:2) | Saccharolipids, SL | Monogalactosyldiacylglycerol | 801.5487215 | 6.3543 |
| pos_425 | C45 H76 O10 Na1 | MGDG(18:2/18:3) | Saccharolipids, SL | Monogalactosyldiacylglycerol | 799.5330715 | 5.7862 |
| pos_426 | C45 H74 O10 Na1 | MGDG(18:3/18:3) | Saccharolipids, SL | Monogalactosyldiacylglycerol | 797.5174215 | 5.268 |
| pos_427 | C47 H86 O10 Na1 | MGDG(18:1/20:1) | Saccharolipids, SL | Monogalactosyldiacylglycerol | 833.6113215 | 8.659 |
| pos_428 | C48 H88 O10 N1 | MGDG(22:1/17:3) | Saccharolipids, SL | Monogalactosyldiacylglycerol | 838.6402755 | 8.2411 |
| pos_429 | C50 H92 O10 N1 | MGDG(24:1/17:3) | Saccharolipids, SL | Monogalactosyldiacylglycerol | 866.6715755 | 9.251 |
| pos_430 | C50 H90 O10 N1 | MGDG(24:2/17:3) | Saccharolipids, SL | Monogalactosyldiacylglycerol | 864.6559255 | 8.1567 |
| pos_431 | C41 H78 O8 N2 P1 | MePC(10:0/20:4) | Glycerophospholipids, GP | Methylphosphatidylcholine | 757.5490325 | 4.978 |
| pos_432 | C41 H78 O8 N2 P1 | MePC(12:0/20:4) | Glycerophospholipids, GP | Methylphosphatidylcholine | 757.5490325 | 4.969 |
| pos_434 | C42 H80 O8 N2 P1 | MePC(11:0/22:4) | Glycerophospholipids, GP | Methylphosphatidylcholine | 771.5646825 | 4.721 |
| pos_435 | C44 H82 O8 N2 P1 | MePC(11:0/22:5) | Glycerophospholipids, GP | Methylphosphatidylcholine | 797.5803325 | 4.765 |
| pos_436 | C44 H80 O8 N2 P1 | MePC(11:0/22:6) | Glycerophospholipids, GP | Methylphosphatidylcholine | 795.5646825 | 4.173 |
| pos_437 | C43 H82 O8 N2 P1 | MePC(16:1/18:3) | Glycerophospholipids, GP | Methylphosphatidylcholine | 785.5803325 | 6.021 |
| pos_438 | C44 H82 O8 N2 P1 | MePC(15:0/20:5) | Glycerophospholipids, GP | Methylphosphatidylcholine | 797.5803325 | 4.752 |
| pos_440 | C37 H73 O8 N1 P1 | PA(16:0/18:2) | Glycerophospholipids, GP | Phosphatidic acid | 690.5068335 | 6.357 |
| pos_441 | C39 H77 O8 N1 P1 | PA(18:1/18:1) | Glycerophospholipids, GP | Phosphatidic acid | 718.5381335 | 7.438 |
| pos_442 | C39 H75 O8 N1 P1 | PA(18:1/18:2) | Glycerophospholipids, GP | Phosphatidic acid | 716.5224835 | 6.709 |
| pos_443 | C39 H73 O8 N1 P1 | PA(18:2/18:2) | Glycerophospholipids, GP | Phosphatidic acid | 714.5068335 | 5.7953 |
| pos_444 | C26 H53 O8 N1 P1 | PC(4:0/14:0) | Glycerophospholipids, GP | Phosphatidylcholine | 538.3503335 | 1.0116 |
| pos_445 | C26 H51 O8 N1 P1 | PC(4:0/14:1) | Glycerophospholipids, GP | Phosphatidylcholine | 536.3346835 | 0.7086 |
| pos_447 | C28 H53 O8 N1 P1 | PC(6:0/14:2) | Glycerophospholipids, GP | Phosphatidylcholine | 562.3503335 | 1.5994 |
| pos_448 | C30 H63 O7 N1 P1 | PC(16:0e/6:0) | Glycerophospholipids, GP | Phosphatidylcholine | 580.4336685 | 4.764 |
| pos_449 | C32 H67 O7 N1 P1 | PC(8:0e/16:0) | Glycerophospholipids, GP | Phosphatidylcholine | 608.4649685 | 5.8181 |
| pos_450 | C35 H69 O8 N1 P1 | PC(11:0/16:1) | Glycerophospholipids, GP | Phosphatidylcholine | 662.4755335 | 4.3462 |
| pos_451 | C35 H67 O8 N1 P1 | PC(9:0/18:2) | Glycerophospholipids, GP | Phosphatidylcholine | 660.4598835 | 3.6924 |
| pos_452 | C36 H73 O8 N1 P1 | PC(16:0/12:0) | Glycerophospholipids, GP | Phosphatidylcholine | 678.5068335 | 5.462 |
| pos_453 | C38 H77 O8 N1 P1 | PC(16:0/14:0) | Glycerophospholipids, GP | Phosphatidylcholine | 706.5381335 | 6.3703 |
| pos_454 | C38 H75 O8 N1 P1 | PC(16:1/14:0) | Glycerophospholipids, GP | Phosphatidylcholine | 704.5224835 | 5.5348 |
| pos_455 | C38 H73 O8 N1 P1 | PC(16:0/14:2) | Glycerophospholipids, GP | Phosphatidylcholine | 702.5068335 | 4.9098 |
| pos_457 | C40 H80 O8 N1 P1 Na1 | PC(16:0/16:0) | Glycerophospholipids, GP | Phosphatidylcholine | 756.5513785 | 7.3528 |
| pos_458 | C40 H81 O8 N1 P1 | PC(18:0/14:0) | Glycerophospholipids, GP | Phosphatidylcholine | 734.5694335 | 7.9741 |
| pos_459 | C40 H79 O8 N1 P1 | PC(16:0/16:1) | Glycerophospholipids, GP | Phosphatidylcholine | 732.5537835 | 6.4852 |
| pos_460 | C40 H79 O8 N1 P1 | PC(18:1/14:0) | Glycerophospholipids, GP | Phosphatidylcholine | 732.5537835 | 7.0825 |
| pos_461 | C40 H76 O8 N1 P1 Na1 | PC(14:0/18:2) | Glycerophospholipids, GP | Phosphatidylcholine | 752.5200785 | 5.7457 |
| pos_462 | C40 H77 O8 N1 P1 | PC(16:1/16:1) | Glycerophospholipids, GP | Phosphatidylcholine | 730.5381335 | 5.9124 |
| pos_463 | C40 H75 O8 N1 P1 | PC(10:0/22:3) | Glycerophospholipids, GP | Phosphatidylcholine | 728.5224835 | 5.1885 |
| pos_464 | C40 H75 O8 N1 P1 | PC(18:2/14:1) | Glycerophospholipids, GP | Phosphatidylcholine | 728.5224835 | 5.761 |
| pos_465 | C41 H83 O8 N1 P1 | PC(16:0/17:0) | Glycerophospholipids, GP | Phosphatidylcholine | 748.5850835 | 7.9217 |
| pos_466 | C41 H81 O8 N1 P1 | PC(15:0/18:1) | Glycerophospholipids, GP | Phosphatidylcholine | 746.5694335 | 6.9536 |
| pos_467 | C41 H79 O8 N1 P1 | PC(15:0/18:2) | Glycerophospholipids, GP | Phosphatidylcholine | 744.5537835 | 6.1377 |
| pos_468 | C41 H77 O8 N1 P1 | PC(15:0/18:3) | Glycerophospholipids, GP | Phosphatidylcholine | 742.5381335 | 5.5145 |
| pos_470 | C42 H82 O8 N1 P1 Na1 | PC(16:0/18:1) | Glycerophospholipids, GP | Phosphatidylcholine | 782.5670285 | 7.4362 |
| pos_471 | C42 H80 O8 N1 P1 Na1 | PC(16:0/18:2) | Glycerophospholipids, GP | Phosphatidylcholine | 780.5513785 | 6.6685 |
| pos_472 | C42 H79 O8 N1 P1 | PC(16:1/18:2) | Glycerophospholipids, GP | Phosphatidylcholine | 756.5537835 | 4.5731 |
| pos_473 | C42 H78 O8 N1 P1 Na1 | PC(16:0/18:3) | Glycerophospholipids, GP | Phosphatidylcholine | 778.5357285 | 6.1101 |
| pos_474 | C42 H77 O8 N1 P1 | PC(16:1/18:3) | Glycerophospholipids, GP | Phosphatidylcholine | 754.5381335 | 5.2887 |
| pos_475 | C43 H85 O8 N1 P1 | PC(17:0/18:1) | Glycerophospholipids, GP | Phosphatidylcholine | 774.6007335 | 7.9799 |
| pos_476 | C43 H83 O8 N1 P1 | PC(17:0/18:2) | Glycerophospholipids, GP | Phosphatidylcholine | 772.5850835 | 7.2341 |
| pos_477 | C43 H83 O8 N1 P1 | PC(17:1/18:1) | Glycerophospholipids, GP | Phosphatidylcholine | 772.5850835 | 7.484 |
| pos_478 | C43 H81 O8 N1 P1 | PC(17:1/18:2) | Glycerophospholipids, GP | Phosphatidylcholine | 770.5694335 | 6.306 |
| pos_479 | C43 H81 O8 N1 P1 | PC(17:0/18:3) | Glycerophospholipids, GP | Phosphatidylcholine | 770.5694335 | 6.5196 |
| pos_480 | C43 H79 O8 N1 P1 | PC(15:0/20:4) | Glycerophospholipids, GP | Phosphatidylcholine | 768.5537835 | 5.6346 |
| pos_481 | C44 H89 O8 N1 P1 | PC(20:0/16:0) | Glycerophospholipids, GP | Phosphatidylcholine | 790.6320335 | 9.561 |
| pos_482 | C44 H86 O8 N1 P1 Na1 | PC(18:0/18:1) | Glycerophospholipids, GP | Phosphatidylcholine | 810.5983285 | 8.517 |
| pos_483 | C44 H84 O8 N1 P1 Na1 | PC(18:1/18:1) | Glycerophospholipids, GP | Phosphatidylcholine | 808.5826785 | 7.5073 |
| pos_484 | C44 H85 O8 N1 P1 | PC(18:0/18:2) | Glycerophospholipids, GP | Phosphatidylcholine | 786.6007335 | 7.5378 |
| pos_485 | C44 H82 O8 N1 P1 Na1 | PC(18:1/18:2) | Glycerophospholipids, GP | Phosphatidylcholine | 806.5670285 | 6.7546 |
| pos_487 | C44 H80 O8 N1 P1 Na1 | PC(18:2/18:2) | Glycerophospholipids, GP | Phosphatidylcholine | 804.5513785 | 6.0312 |
| pos_488 | C44 H81 O8 N1 P1 | PC(18:1/18:3) | Glycerophospholipids, GP | Phosphatidylcholine | 782.5694335 | 7.4707 |
| pos_489 | C44 H79 O8 N1 P1 | PC(16:1/20:4) | Glycerophospholipids, GP | Phosphatidylcholine | 780.5537835 | 4.0104 |
| pos_490 | C44 H78 O8 N1 P1 Na1 | PC(18:3/18:2) | Glycerophospholipids, GP | Phosphatidylcholine | 802.5357285 | 5.4738 |
| pos_491 | C44 H77 O8 N1 P1 | PC(16:1/20:5) | Glycerophospholipids, GP | Phosphatidylcholine | 778.5381335 | 4.9499 |
| pos_492 | C46 H88 O8 N1 P1 Na1 | PC(20:1/18:1) | Glycerophospholipids, GP | Phosphatidylcholine | 836.6139785 | 8.4594 |
| pos_493 | C46 H89 O8 N1 P1 | PC(20:0/18:2) | Glycerophospholipids, GP | Phosphatidylcholine | 814.6320335 | 8.7352 |
| pos_494 | C46 H86 O8 N1 P1 Na1 | PC(20:1/18:2) | Glycerophospholipids, GP | Phosphatidylcholine | 834.5983285 | 7.7004 |
| pos_495 | C46 H85 O8 N1 P1 | PC(18:1/20:3) | Glycerophospholipids, GP | Phosphatidylcholine | 810.6007335 | 6.9175 |
| pos_496 | C46 H77 O8 N1 P1 | PC(18:4/20:4) | Glycerophospholipids, GP | Phosphatidylcholine | 802.5381335 | 5.476 |
| pos_497 | C47 H91 O8 N1 P1 | PC(18:2/21:0) | Glycerophospholipids, GP | Phosphatidylcholine | 828.6476835 | 9.3686 |
| pos_499 | C21 H45 O7 N1 P1 | PE(10:0e/6:0) | Glycerophospholipids, GP | Phosphatidylethanolamine | 454.2928185 | 1.7961 |
| pos_500 | C23 H45 O8 N1 P1 | PE(6:0/12:1) | Glycerophospholipids, GP | Phosphatidylethanolamine | 494.2877335 | 0.838 |
| pos_501 | C23 H47 O7 N1 P1 | PE(8:1e/10:0) | Glycerophospholipids, GP | Phosphatidylethanolamine | 480.3084685 | 1.775 |
| pos_502 | C23 H44 O7 N1 P1 Na1 | PE(8:0e/10:2) | Glycerophospholipids, GP | Phosphatidylethanolamine | 500.2747635 | 1.4605 |
| pos_503 | C37 H74 O8 N1 P1 Na1 | PE(16:0/16:0) | Glycerophospholipids, GP | Phosphatidylethanolamine | 714.5044285 | 5.8682 |
| pos_504 | C37 H71 O8 N1 P1 | PE(14:0/18:2) | Glycerophospholipids, GP | Phosphatidylethanolamine | 688.4911835 | 5.751 |
| pos_505 | C38 H74 O8 N1 P1 Na1 | PE(17:1/16:0) | Glycerophospholipids, GP | Phosphatidylethanolamine | 726.5044285 | 3.9157 |
| pos_506 | C39 H79 O8 N1 P1 | PE(18:0/16:0) | Glycerophospholipids, GP | Phosphatidylethanolamine | 720.5537835 | 8.345 |
| pos_507 | C39 H76 O8 N1 P1 Na1 | PE(16:0/18:1) | Glycerophospholipids, GP | Phosphatidylethanolamine | 740.5200785 | 6.0744 |
| pos_510 | C39 H73 O8 N1 P1 | PE(16:1/18:2) | Glycerophospholipids, GP | Phosphatidylethanolamine | 714.5068335 | 5.8883 |
| pos_511 | C40 H77 O8 N1 P1 | PE(17:1/18:1) | Glycerophospholipids, GP | Phosphatidylethanolamine | 730.5381335 | 6.3928 |
| pos_512 | C40 H75 O8 N1 P1 | PE(17:1/18:2) | Glycerophospholipids, GP | Phosphatidylethanolamine | 728.5224835 | 5.7687 |
| pos_513 | C40 H73 O8 N1 P1 | PE(17:1/18:3) | Glycerophospholipids, GP | Phosphatidylethanolamine | 726.5068335 | 5.283 |
| pos_514 | C41 H81 O8 N1 P1 | PE(18:0/18:1) | Glycerophospholipids, GP | Phosphatidylethanolamine | 746.5694335 | 8.3936 |
| pos_515 | C41 H79 O8 N1 P1 | PE(18:1/18:1) | Glycerophospholipids, GP | Phosphatidylethanolamine | 744.5537835 | 7.4871 |
| pos_516 | C41 H76 O8 N1 P1 Li1 | PE(18:1/18:2) | Glycerophospholipids, GP | Phosphatidylethanolamine | 748.5463085 | 5.3826 |
| pos_517 | C41 H75 O8 N1 P1 | PE(18:1/18:3) | Glycerophospholipids, GP | Phosphatidylethanolamine | 740.5224835 | 4.6588 |
| pos_518 | C41 H74 O8 N1 P1 Na1 | PE(18:2/18:2) | Glycerophospholipids, GP | Phosphatidylethanolamine | 762.5044285 | 4.9656 |
| pos_519 | C41 H76 O7 N1 P1 Li1 | PE(18:3e/18:1) | Glycerophospholipids, GP | Phosphatidylethanolamine | 732.5513935 | 7.083 |
| pos_520 | C41 H73 O8 N1 P1 | PE(18:3/18:2) | Glycerophospholipids, GP | Phosphatidylethanolamine | 738.5068335 | 4.0742 |
| pos_521 | C41 H70 O8 N1 P1 Li1 | PE(18:4/18:2) | Glycerophospholipids, GP | Phosphatidylethanolamine | 742.4993585 | 3.9347 |
| pos_524 | C38 H76 O8 N0 P1 | PEt(16:0/17:0) | Glycerophospholipids, GP | Phosphatidylethanol | 691.5272345 | 2.588 |
| pos_525 | C40 H78 O8 N0 P1 | PEt(17:0/18:1) | Glycerophospholipids, GP | Phosphatidylethanol | 717.5428845 | 2.5752 |
| pos_526 | C42 H80 O8 N0 P1 | PEt(19:1/18:1) | Glycerophospholipids, GP | Phosphatidylethanol | 743.5585345 | 2.7336 |
| pos_527 | C22 H45 O9 N0 P1 Na1 | PG(8:0e/8:0) | Glycerophospholipids, GP | Phosphatidylglycerol | 507.2693445 | 1.6266 |
| pos_528 | C33 H63 O10 N0 P1 Li1 | PG(16:0/11:1) | Glycerophospholipids, GP | Phosphatidylglycerol | 657.4313395 | 4.645 |
| pos_529 | C41 H85 O10 N2 P1 | PG(16:0/13:0) | Glycerophospholipids, GP | Phosphatidylglycerol | 796.5936375 | 6.1273 |
| pos_530 | C38 H79 O10 N1 P1 | PG(16:0/16:0) | Glycerophospholipids, GP | Phosphatidylglycerol | 740.5436135 | 6.5874 |
| pos_531 | C39 H74 O10 N0 P1 | PG(15:0/18:2) | Glycerophospholipids, GP | Phosphatidylglycerol | 733.5014145 | 3.521 |
| pos_532 | C40 H81 O10 N1 P1 | PG(16:0/18:1) | Glycerophospholipids, GP | Phosphatidylglycerol | 766.5592635 | 6.6553 |
| pos_533 | C40 H75 O10 N0 P1 Na1 | PG(16:0/18:2) | Glycerophospholipids, GP | Phosphatidylglycerol | 769.4990095 | 5.6618 |
| pos_534 | C40 H74 O10 N0 P1 | PG(16:0/18:3) | Glycerophospholipids, GP | Phosphatidylglycerol | 745.5014145 | 3.5001 |
| pos_535 | C40 H72 O10 N0 P1 | PG(18:4/16:0) | Glycerophospholipids, GP | Phosphatidylglycerol | 743.4857645 | 3.329 |
| pos_536 | C42 H83 O10 N1 P1 | PG(18:1/18:1) | Glycerophospholipids, GP | Phosphatidylglycerol | 792.5749135 | 4.085 |
| pos_537 | C42 H81 O10 N1 P1 | PG(18:1/18:2) | Glycerophospholipids, GP | Phosphatidylglycerol | 790.5592635 | 6.0815 |
| pos_538 | C42 H79 O10 N1 P1 | PG(18:2/18:2) | Glycerophospholipids, GP | Phosphatidylglycerol | 788.5436135 | 5.1811 |
| pos_539 | C50 H103 O10 N2 P1 | PG(18:0/20:0) | Glycerophospholipids, GP | Phosphatidylglycerol | 922.7344875 | 13.792 |
| pos_542 | C47 H92 O10 N0 P1 | PG(25:1/16:0) | Glycerophospholipids, GP | Phosphatidylglycerol | 847.6422645 | 9.983 |
| pos_545 | C50 H101 O10 N1 P1 | PG(28:1/16:0) | Glycerophospholipids, GP | Phosphatidylglycerol | 906.7157635 | 8.6632 |
| pos_547 | C52 H102 O10 N0 P1 | PG(28:0/18:1) | Glycerophospholipids, GP | Phosphatidylglycerol | 917.7205145 | 10.1144 |
| pos_549 | C52 H98 O10 N0 P1 | PG(28:0/18:3) | Glycerophospholipids, GP | Phosphatidylglycerol | 913.6892145 | 9.2382 |
| pos_550 | C52 H98 O10 N0 P1 | PG(28:1/18:2) | Glycerophospholipids, GP | Phosphatidylglycerol | 913.6892145 | 9.9272 |
| pos_551 | C56 H107 O10 N0 P1 Na1 | PG(32:0/18:2) | Glycerophospholipids, GP | Phosphatidylglycerol | 993.7494095 | 10.493 |
| pos_552 | C56 H105 O10 N0 P1 Na1 | PG(32:1/18:2) | Glycerophospholipids, GP | Phosphatidylglycerol | 991.7337595 | 9.9314 |
| pos_553 | C43 H83 O13 N1 P1 | PI(16:0/18:2) | Glycerophospholipids, GP | Phosphatidylinositol | 852.5596585 | 5.8031 |
| pos_554 | C45 H85 O13 N1 P1 | PI(18:1/18:2) | Glycerophospholipids, GP | Phosphatidylinositol | 878.5753085 | 5.925 |
| pos_555 | C45 H83 O13 N1 P1 | PI(18:2/18:2) | Glycerophospholipids, GP | Phosphatidylinositol | 876.5596585 | 5.288 |
| pos_557 | C51 H95 O13 N1 P1 | PI(24:2/18:2) | Glycerophospholipids, GP | Phosphatidylinositol | 960.6535585 | 5.8278 |
| pos_558 | C57 H109 O13 N0 P1 Li1 | PI(31:0/17:1) | Glycerophospholipids, GP | Phosphatidylinositol | 1039.776034 | 11.3501 |
| pos_559 | C22 H41 O8 N0 P1 Na1 | PMe(4:0/14:1) | Glycerophospholipids, GP | Phosphatidylmethanol | 487.2431295 | 0.8331 |
| pos_560 | C38 H73 O8 N0 P1 Na1 | PMe(16:0/18:1) | Glycerophospholipids, GP | Phosphatidylmethanol | 711.4935295 | 7.0322 |
| pos_561 | C38 H71 O8 N0 P1 Na1 | PMe(16:0/18:2) | Glycerophospholipids, GP | Phosphatidylmethanol | 709.4778795 | 3.9314 |
| pos_562 | C40 H73 O8 N0 P1 Na1 | PMe(18:1/18:2) | Glycerophospholipids, GP | Phosphatidylmethanol | 735.4935295 | 6.418 |
| pos_563 | C40 H71 O8 N0 P1 Na1 | PMe(16:0/20:4) | Glycerophospholipids, GP | Phosphatidylmethanol | 733.4778795 | 5.7632 |
| pos_564 | C40 H72 O8 N0 P1 | PMe(18:1/18:3) | Glycerophospholipids, GP | Phosphatidylmethanol | 711.4959345 | 7.029 |
| pos_565 | C40 H70 O8 N0 P1 | PMe(18:3/18:2) | Glycerophospholipids, GP | Phosphatidylmethanol | 709.4802845 | 3.9226 |
| pos_566 | C40 H68 O8 N0 P1 | PMe(18:3/18:3) | Glycerophospholipids, GP | Phosphatidylmethanol | 707.4646345 | 2.259 |
| pos_567 | C41 H69 O8 N0 P1 Li1 | PMe(17:1/20:5) | Glycerophospholipids, GP | Phosphatidylmethanol | 727.4884595 | 5.3551 |
| pos_568 | C42 H76 O8 N0 P1 | PMe(20:2/18:2) | Glycerophospholipids, GP | Phosphatidylmethanol | 739.5272345 | 2.0766 |
| pos_570 | C42 H70 O8 N0 P1 | PMe(20:5/18:2) | Glycerophospholipids, GP | Phosphatidylmethanol | 733.4802845 | 5.7578 |
| pos_571 | C43 H71 O8 N0 P1 Li1 | PMe(17:1/22:6) | Glycerophospholipids, GP | Phosphatidylmethanol | 753.5041095 | 5.3824 |
| pos_572 | C38 H74 O10 N3 P1 | PS(16:0/10:2) | Glycerophospholipids, GP | Phosphatidylserine | 763.5106365 | 4.3602 |
| pos_574 | C66 H132 O10 N3 P1 | PS(36:0/18:1) | Glycerophospholipids, GP | Phosphatidylserine | 1157.964486 | 13.021 |
| pos_575 | C43 H75 O6 N2 P1 Li1 | SM(d18:2/20:5) | Sphingolipids, SP | Sphingomyelin | 753.5517275 | 7.6422 |
| pos_576 | C45 H78 O6 N2 P1 | SM(d18:2/22:6) | Sphingolipids, SP | Sphingomyelin | 773.5592025 | 4.63 |
| pos_577 | C47 H84 O6 N2 P1 | SM(d18:1/24:6) | Sphingolipids, SP | Sphingomyelin | 803.6061525 | 6.551 |
| pos_578 | C44 H82 O7 N2 P1 | SM(t18:1/21:4) | Sphingolipids, SP | Sphingomyelin | 781.5854175 | 6.514 |
| pos_579 | C44 H80 O7 N2 P1 | SM(t18:1/21:5) | Sphingolipids, SP | Sphingomyelin | 779.5697675 | 5.8301 |
| pos_580 | C16 H34 O2 N1 | SPH(d16:1) | Sphingolipids, SP | Sphingosine | 272.2584055 | 0.7822 |
| pos_581 | C18 H40 O2 N1 | SPH(d18:0) | Sphingolipids, SP | Sphingosine | 302.3053555 | 1.4653 |
| pos_582 | C18 H38 O2 N1 | SPH(d18:1) | Sphingolipids, SP | Sphingosine | 300.2897055 | 1.219 |
| pos_584 | C20 H42 O2 N1 | SPH(d20:1) | Sphingolipids, SP | Sphingosine | 328.3210055 | 1.5537 |
| pos_585 | C22 H46 O2 N1 | SPH(d22:1) | Sphingolipids, SP | Sphingosine | 356.3523055 | 2.3329 |
| pos_586 | C18 H40 O1 N1 | SPH(m18:0) | Sphingolipids, SP | Sphingosine | 286.3104405 | 1.4807 |
| pos_587 | C16 H36 O3 N1 | SPH(t16:0) | Sphingolipids, SP | Sphingosine | 290.2689705 | 0.8002 |
| pos_588 | C18 H40 O3 N1 | SPH(t18:0) | Sphingolipids, SP | Sphingosine | 318.3002705 | 1.2203 |
| pos_589 | C18 H38 O3 N1 | SPH(t18:1) | Sphingolipids, SP | Sphingosine | 316.2846205 | 1.0331 |
| pos_590 | C20 H44 O3 N1 | SPH(t20:0) | Sphingolipids, SP | Sphingosine | 346.3315705 | 1.5975 |
| pos_591 | C45 H82 O12 S1 N1 | SQDG(18:2/18:2) | Saccharolipids, SL | Sulfoquinovosyldiacylglycerol | 860.5552275 | 5.186 |
| pos_592 | C29 H49 O0 | SiE(0:0) | Sterol Lipids, ST | Sitosteryl ester | 397.3828765 | 4.9426 |
| pos_593 | C47 H86 O2 N1 | SiE(18:1) | Sterol Lipids, ST | Sitosteryl ester | 696.6653055 | 15.0103 |
| pos_594 | C47 H84 O2 N1 | SiE(18:2) | Sterol Lipids, ST | Sitosteryl ester | 694.6496555 | 14.6224 |
| pos_597 | C49 H79 O2 | SiE(20:5) | Sterol Lipids, ST | Sitosteryl ester | 699.6074565 | 14.6224 |
| pos_598 | C29 H47 O0 | StE(0:0) | Sterol Lipids, ST | Stigmasteryl ester | 395.3672265 | 3.247 |
| pos_600 | C47 H77 O2 | StE(18:3) | Sterol Lipids, ST | Stigmasteryl ester | 673.5918065 | 11.3751 |
| pos_601 | C48 H84 O2 N1 | StE(19:2) | Sterol Lipids, ST | Stigmasteryl ester | 706.6496555 | 14.28 |
| pos_602 | C49 H77 O2 | StE(20:5) | Sterol Lipids, ST | Stigmasteryl ester | 697.5918065 | 14.2929 |
| pos_603 | C66 H112 O2 N1 | StE(37:6) | Sterol Lipids, ST | Stigmasteryl ester | 950.8687555 | 13.7508 |
| pos_604 | C24 H44 O6 K1 | TG(6:0/6:0/9:0) | Glycerolipids, GL | Triglyceride | 467.2769495 | 1.0067 |
| pos_605 | C24 H42 O6 K1 | TG(4:0/6:0/11:1) | Glycerolipids, GL | Triglyceride | 465.2612995 | 0.8384 |
| pos_606 | C24 H41 O6 | TG(4:0/6:0/11:2) | Glycerolipids, GL | Triglyceride | 425.2897665 | 1.5402 |
| pos_607 | C31 H62 O6 N2 | TG(6:0/8:0/8:0) | Glycerolipids, GL | Triglyceride | 558.4602395 | 4.6228 |
| pos_610 | C28 H52 O6 Na1 | TG(6:0/6:0/13:0) | Glycerolipids, GL | Triglyceride | 507.3656115 | 3.1021 |
| pos_612 | C28 H51 O6 | TG(6:0/8:0/11:1) | Glycerolipids, GL | Triglyceride | 483.3680165 | 3.4559 |
| pos_613 | C29 H58 O6 N1 | TG(8:0/8:0/10:0) | Glycerolipids, GL | Triglyceride | 516.4258655 | 3.2905 |
| pos_614 | C29 H51 O6 | TG(6:0/6:0/14:2) | Glycerolipids, GL | Triglyceride | 495.3680165 | 3.244 |
| pos_616 | C29 H48 O6 N1 | TG(6:0/10:2/10:3) | Glycerolipids, GL | Triglyceride | 506.3476155 | 2.2734 |
| pos_617 | C29 H44 O6 N1 | TG(6:0/10:3/10:4) | Glycerolipids, GL | Triglyceride | 502.3163155 | 1.6298 |
| pos_618 | C30 H58 O6 N1 | TG(11:0/6:0/10:1) | Glycerolipids, GL | Triglyceride | 528.4258655 | 3.6778 |
| pos_619 | C30 H53 O6 | TG(6:0/10:1/11:1) | Glycerolipids, GL | Triglyceride | 509.3836665 | 3.3699 |
| pos_620 | C30 H53 O6 | TG(11:0/6:0/10:2) | Glycerolipids, GL | Triglyceride | 509.3836665 | 6.3023 |
| pos_622 | C30 H53 O6 | TG(6:0/10:0/11:2) | Glycerolipids, GL | Triglyceride | 509.3836665 | 10.7987 |
| pos_626 | C30 H51 O6 | TG(11:0/6:0/10:3) | Glycerolipids, GL | Triglyceride | 507.3680165 | 3.1006 |
| pos_627 | C30 H51 O6 | TG(8:0/9:0/10:3) | Glycerolipids, GL | Triglyceride | 507.3680165 | 3.611 |
| pos_628 | C30 H51 O6 | TG(6:0/9:0/12:3) | Glycerolipids, GL | Triglyceride | 507.3680165 | 5.6519 |
| pos_631 | C31 H53 O5 | TG(12:1e/6:0/10:3) | Glycerolipids, GL | Triglyceride | 505.3887515 | 7.3529 |
| pos_632 | C32 H63 O5 | TG(14:0e/6:0/9:0) | Glycerolipids, GL | Triglyceride | 527.4670015 | 5.2541 |
| pos_634 | C32 H53 O6 | TG(6:0/10:4/13:0) | Glycerolipids, GL | Triglyceride | 533.3836665 | 3.659 |
| pos_635 | C32 H55 O5 | TG(12:1e/6:0/11:3) | Glycerolipids, GL | Triglyceride | 519.4044015 | 7.3565 |
| pos_637 | C33 H57 O5 | TG(12:1e/6:0/12:3) | Glycerolipids, GL | Triglyceride | 533.4200515 | 3.928 |
| pos_638 | C33 H57 O5 | TG(12:1e/8:0/10:3) | Glycerolipids, GL | Triglyceride | 533.4200515 | 5.5736 |
| pos_639 | C39 H68 O6 N2 | TG(6:0/12:2/12:3) | Glycerolipids, GL | Triglyceride | 660.5071895 | 3.921 |
| pos_640 | C33 H55 O5 | TG(12:1e/6:0/12:4) | Glycerolipids, GL | Triglyceride | 531.4044015 | 3.2835 |
| pos_641 | C39 H66 O6 N2 | TG(6:0/12:3/12:3) | Glycerolipids, GL | Triglyceride | 658.4915395 | 3.9982 |
| pos_642 | C34 H68 O6 N1 | TG(15:0/6:0/10:0) | Glycerolipids, GL | Triglyceride | 586.5041155 | 3.8061 |
| pos_644 | C34 H64 O6 Na1 | TG(16:0/6:0/9:0) | Glycerolipids, GL | Triglyceride | 591.4595115 | 4.3368 |
| pos_645 | C34 H67 O5 | TG(14:0e/8:0/9:0) | Glycerolipids, GL | Triglyceride | 555.4983015 | 5.978 |
| pos_646 | C34 H62 O6 Na1 | TG(16:1/6:0/9:0) | Glycerolipids, GL | Triglyceride | 589.4438615 | 3.5083 |
| pos_647 | C34 H62 O6 Na1 | TG(4:0/11:1/16:0) | Glycerolipids, GL | Triglyceride | 589.4438615 | 4.2723 |
| pos_649 | C34 H65 O5 | TG(12:0e/9:0/10:1) | Glycerolipids, GL | Triglyceride | 553.4826515 | 5.2601 |
| pos_650 | C34 H65 O5 | TG(14:1e/8:0/9:0) | Glycerolipids, GL | Triglyceride | 553.4826515 | 5.5396 |
| pos_652 | C34 H65 O5 | TG(14:1e/6:0/11:0) | Glycerolipids, GL | Triglyceride | 553.4826515 | 5.8397 |
| pos_653 | C34 H62 O5 Na1 | TG(12:0e/8:0/11:2) | Glycerolipids, GL | Triglyceride | 573.4489465 | 3.5531 |
| pos_654 | C34 H63 O5 | TG(12:0e/9:0/10:2) | Glycerolipids, GL | Triglyceride | 551.4670015 | 4.33 |
| pos_655 | C34 H57 O6 | TG(6:0/11:1/14:3) | Glycerolipids, GL | Triglyceride | 561.4149665 | 5.2198 |
| pos_656 | C34 H58 O6 N1 | TG(6:0/11:3/14:2) | Glycerolipids, GL | Triglyceride | 576.4258655 | 3.4947 |
| pos_657 | C35 H70 O6 N1 | TG(16:0/8:0/8:0) | Glycerolipids, GL | Triglyceride | 600.5197655 | 5.8935 |
| pos_658 | C35 H64 O6 Na1 | TG(6:0/12:1/14:0) | Glycerolipids, GL | Triglyceride | 603.4595115 | 4.811 |
| pos_659 | C35 H66 O5 Na1 | TG(12:1e/6:0/14:0) | Glycerolipids, GL | Triglyceride | 589.4802465 | 7.8505 |
| pos_660 | C35 H65 O5 | TG(12:0e/10:0/10:2) | Glycerolipids, GL | Triglyceride | 565.4826515 | 11.286 |
| pos_661 | C35 H62 O6 N1 | TG(16:0/6:0/10:4) | Glycerolipids, GL | Triglyceride | 592.4571655 | 4.335 |
| pos_662 | C41 H70 O6 N2 | TG(6:0/12:2/14:4) | Glycerolipids, GL | Triglyceride | 686.5228395 | 3.9377 |
| pos_663 | C41 H70 O6 N2 | TG(8:0/10:2/14:4) | Glycerolipids, GL | Triglyceride | 686.5228395 | 4.2815 |
| pos_665 | C36 H69 O6 | TG(15:0/9:0/9:0) | Glycerolipids, GL | Triglyceride | 597.5088665 | 3.3445 |
| pos_666 | C36 H69 O6 | TG(15:0/6:0/12:0) | Glycerolipids, GL | Triglyceride | 597.5088665 | 3.4973 |
| pos_667 | C36 H72 O6 N1 | TG(17:0/6:0/10:0) | Glycerolipids, GL | Triglyceride | 614.5354155 | 4.6311 |
| pos_668 | C36 H68 O6 Li1 | TG(6:0/6:0/21:0) | Glycerolipids, GL | Triglyceride | 603.5170415 | 5.124 |
| pos_669 | C36 H67 O6 | TG(15:0/6:0/12:1) | Glycerolipids, GL | Triglyceride | 595.4932165 | 2.8645 |
| pos_671 | C36 H70 O6 N1 | TG(6:0/9:0/18:1) | Glycerolipids, GL | Triglyceride | 612.5197655 | 4.3558 |
| pos_672 | C36 H69 O5 | TG(14:0e/9:0/10:1) | Glycerolipids, GL | Triglyceride | 581.5139515 | 6.1499 |
| pos_673 | C36 H68 O5 K1 | TG(12:1e/6:0/15:0) | Glycerolipids, GL | Triglyceride | 619.4698345 | 6.5093 |
| pos_674 | C36 H65 O6 | TG(9:0/12:1/12:1) | Glycerolipids, GL | Triglyceride | 593.4775665 | 2.2951 |
| pos_676 | C36 H68 O6 N1 | TG(16:0/6:0/11:2) | Glycerolipids, GL | Triglyceride | 610.5041155 | 2.9553 |
| pos_677 | C36 H68 O6 N1 | TG(6:0/9:0/18:2) | Glycerolipids, GL | Triglyceride | 610.5041155 | 3.0861 |
| pos_679 | C36 H67 O5 | TG(14:0e/9:0/10:2) | Glycerolipids, GL | Triglyceride | 579.4983015 | 3.8976 |
| pos_680 | C36 H67 O5 | TG(14:0e/8:0/11:2) | Glycerolipids, GL | Triglyceride | 579.4983015 | 4.632 |
| pos_681 | C36 H63 O6 | TG(6:0/10:2/17:1) | Glycerolipids, GL | Triglyceride | 591.4619165 | 2.047 |
| pos_683 | C36 H63 O6 | TG(4:0/11:1/18:2) | Glycerolipids, GL | Triglyceride | 591.4619165 | 3.1646 |
| pos_684 | C36 H62 O6 Na1 | TG(6:0/9:0/18:3) | Glycerolipids, GL | Triglyceride | 613.4438615 | 3.2856 |
| pos_685 | C36 H66 O6 N1 | TG(17:0/6:0/10:3) | Glycerolipids, GL | Triglyceride | 608.4884655 | 4.6484 |
| pos_686 | C36 H63 O6 | TG(16:0/6:0/11:3) | Glycerolipids, GL | Triglyceride | 591.4619165 | 4.9144 |
| pos_687 | C36 H65 O5 | TG(14:0e/8:0/11:3) | Glycerolipids, GL | Triglyceride | 577.4826515 | 3.4976 |
| pos_689 | C36 H65 O5 | TG(16:1e/6:0/11:2) | Glycerolipids, GL | Triglyceride | 577.4826515 | 4.5087 |
| pos_691 | C36 H61 O6 | TG(18:4/6:0/9:0) | Glycerolipids, GL | Triglyceride | 589.4462665 | 2.2417 |
| pos_694 | C36 H61 O6 | TG(6:0/10:3/17:1) | Glycerolipids, GL | Triglyceride | 589.4462665 | 3.2204 |
| pos_695 | C36 H61 O6 | TG(4:0/11:2/18:2) | Glycerolipids, GL | Triglyceride | 589.4462665 | 3.6056 |
| pos_697 | C36 H63 O5 | TG(14:1e/8:0/11:3) | Glycerolipids, GL | Triglyceride | 575.4670015 | 3.5063 |
| pos_699 | C36 H63 O5 | TG(12:1e/9:0/12:3) | Glycerolipids, GL | Triglyceride | 575.4670015 | 4.9547 |
| pos_700 | C36 H63 O5 | TG(12:0e/10:3/11:1) | Glycerolipids, GL | Triglyceride | 575.4670015 | 5.5632 |
| pos_702 | C36 H61 O5 | TG(16:2e/6:0/11:3) | Glycerolipids, GL | Triglyceride | 573.4513515 | 2.0538 |
| pos_703 | C36 H61 O5 | TG(14:1e/9:0/10:4) | Glycerolipids, GL | Triglyceride | 573.4513515 | 4.769 |
| pos_705 | C37 H71 O6 | TG(16:0/8:0/10:0) | Glycerolipids, GL | Triglyceride | 611.5245165 | 5.8603 |
| pos_706 | C37 H71 O6 | TG(16:0/9:0/9:0) | Glycerolipids, GL | Triglyceride | 611.5245165 | 6.9745 |
| pos_708 | C37 H72 O6 N1 | TG(8:0/8:0/18:1) | Glycerolipids, GL | Triglyceride | 626.5354155 | 4.5361 |
| pos_709 | C37 H72 O6 N1 | TG(6:0/10:0/18:1) | Glycerolipids, GL | Triglyceride | 626.5354155 | 5.121 |
| pos_710 | C37 H72 O6 N1 | TG(16:0/6:0/12:1) | Glycerolipids, GL | Triglyceride | 626.5354155 | 5.404 |
| pos_711 | C37 H74 O5 N1 | TG(12:1e/11:0/11:0) | Glycerolipids, GL | Triglyceride | 612.5561505 | 2.573 |
| pos_712 | C37 H74 O5 N1 | TG(12:1e/6:0/16:0) | Glycerolipids, GL | Triglyceride | 612.5561505 | 6.1574 |
| pos_713 | C37 H70 O5 K1 | TG(18:0e/6:0/10:1) | Glycerolipids, GL | Triglyceride | 633.4854845 | 8.8056 |
| pos_715 | C37 H70 O6 N1 | TG(8:0/8:0/18:2) | Glycerolipids, GL | Triglyceride | 624.5197655 | 3.8747 |
| pos_716 | C37 H70 O6 N1 | TG(6:0/6:0/22:2) | Glycerolipids, GL | Triglyceride | 624.5197655 | 4.207 |
| pos_718 | C37 H70 O6 N1 | TG(6:0/10:0/18:2) | Glycerolipids, GL | Triglyceride | 624.5197655 | 5.3976 |
| pos_719 | C37 H67 O6 | TG(16:0/8:0/10:2) | Glycerolipids, GL | Triglyceride | 607.4932165 | 5.8934 |
| pos_720 | C37 H68 O5 K1 | TG(14:0e/10:1/10:1) | Glycerolipids, GL | Triglyceride | 631.4698345 | 8.0368 |
| pos_721 | C37 H72 O5 N1 | TG(14:0e/8:0/12:2) | Glycerolipids, GL | Triglyceride | 610.5405005 | 8.2956 |
| pos_722 | C37 H69 O5 | TG(14:0e/10:0/10:2) | Glycerolipids, GL | Triglyceride | 593.5139515 | 9.573 |
| pos_723 | C37 H69 O5 | TG(14:0e/9:0/11:2) | Glycerolipids, GL | Triglyceride | 593.5139515 | 11.9944 |
| pos_724 | C37 H68 O6 N1 | TG(16:0/8:0/10:3) | Glycerolipids, GL | Triglyceride | 622.5041155 | 3.0707 |
| pos_725 | C37 H68 O6 N1 | TG(16:0/6:0/12:3) | Glycerolipids, GL | Triglyceride | 622.5041155 | 3.5544 |
| pos_726 | C37 H65 O6 | TG(4:0/14:3/16:0) | Glycerolipids, GL | Triglyceride | 605.4775665 | 4.4525 |
| pos_728 | C37 H70 O5 N1 | TG(16:1e/6:0/12:2) | Glycerolipids, GL | Triglyceride | 608.5248505 | 4.941 |
| pos_730 | C37 H67 O5 | TG(14:1e/10:1/10:1) | Glycerolipids, GL | Triglyceride | 591.4983015 | 8.765 |
| pos_731 | C37 H63 O6 | TG(14:0/10:2/10:2) | Glycerolipids, GL | Triglyceride | 603.4619165 | 4.797 |
| pos_732 | C37 H68 O5 N1 | TG(14:0e/10:2/10:2) | Glycerolipids, GL | Triglyceride | 606.5092005 | 4.194 |
| pos_735 | C37 H59 O6 | TG(4:0/12:3/18:3) | Glycerolipids, GL | Triglyceride | 599.4306165 | 4.9175 |
| pos_736 | C37 H57 O6 | TG(4:0/12:4/18:3) | Glycerolipids, GL | Triglyceride | 597.4149665 | 4.32 |
| pos_737 | C38 H76 O6 N1 | TG(16:0/9:0/10:0) | Glycerolipids, GL | Triglyceride | 642.5667155 | 6.7985 |
| pos_739 | C38 H73 O5 | TG(14:0e/9:0/12:1) | Glycerolipids, GL | Triglyceride | 609.5452515 | 6.9888 |
| pos_740 | C38 H76 O5 N1 | TG(12:1e/6:0/17:0) | Glycerolipids, GL | Triglyceride | 626.5718005 | 8.795 |
| pos_742 | C38 H72 O6 N1 | TG(11:0/6:0/18:2) | Glycerolipids, GL | Triglyceride | 638.5354155 | 5.707 |
| pos_743 | C38 H71 O5 | TG(16:0e/9:0/10:2) | Glycerolipids, GL | Triglyceride | 607.5296015 | 6.0677 |
| pos_745 | C38 H71 O5 | TG(14:1e/9:0/12:1) | Glycerolipids, GL | Triglyceride | 607.5296015 | 6.551 |
| pos_746 | C38 H67 O6 | TG(6:0/11:1/18:2) | Glycerolipids, GL | Triglyceride | 619.4932165 | 4.639 |
| pos_747 | C38 H67 O6 | TG(6:0/11:2/18:1) | Glycerolipids, GL | Triglyceride | 619.4932165 | 5.3477 |
| pos_748 | C38 H72 O5 N1 | TG(16:1e/9:0/10:2) | Glycerolipids, GL | Triglyceride | 622.5405005 | 5.6405 |
| pos_749 | C38 H65 O6 | TG(6:0/11:1/18:3) | Glycerolipids, GL | Triglyceride | 617.4775665 | 3.5609 |
| pos_750 | C38 H63 O6 | TG(6:0/11:2/18:3) | Glycerolipids, GL | Triglyceride | 615.4619165 | 2.9424 |
| pos_751 | C38 H63 O6 | TG(6:0/11:3/18:2) | Glycerolipids, GL | Triglyceride | 615.4619165 | 3.4951 |
| pos_752 | C38 H63 O6 | TG(18:4/6:0/11:1) | Glycerolipids, GL | Triglyceride | 615.4619165 | 4.2984 |
| pos_754 | C38 H61 O6 | TG(18:4/6:0/11:2) | Glycerolipids, GL | Triglyceride | 613.4462665 | 3.313 |
| pos_755 | C38 H63 O5 | TG(18:3e/6:0/11:3) | Glycerolipids, GL | Triglyceride | 599.4670015 | 5.36 |
| pos_756 | C38 H59 O6 | TG(6:0/11:4/18:3) | Glycerolipids, GL | Triglyceride | 611.4306165 | 3.2692 |
| pos_758 | C39 H78 O6 N1 | TG(4:0/16:0/16:0) | Glycerolipids, GL | Triglyceride | 656.5823655 | 7.542 |
| pos_759 | C39 H78 O6 N1 | TG(18:0/8:0/10:0) | Glycerolipids, GL | Triglyceride | 656.5823655 | 7.7684 |
| pos_760 | C39 H78 O6 N1 | TG(18:0/9:0/9:0) | Glycerolipids, GL | Triglyceride | 656.5823655 | 7.9775 |
| pos_763 | C39 H73 O6 | TG(8:0/10:0/18:1) | Glycerolipids, GL | Triglyceride | 637.5401665 | 5.8134 |
| pos_764 | C39 H73 O6 | TG(18:0/6:0/12:1) | Glycerolipids, GL | Triglyceride | 637.5401665 | 6.8391 |
| pos_766 | C39 H74 O5 Na1 | TG(12:1e/6:0/18:0) | Glycerolipids, GL | Triglyceride | 645.5428465 | 9.8124 |
| pos_767 | C39 H71 O6 | TG(8:0/10:0/18:2) | Glycerolipids, GL | Triglyceride | 635.5245165 | 4.751 |
| pos_768 | C39 H71 O6 | TG(6:0/12:1/18:1) | Glycerolipids, GL | Triglyceride | 635.5245165 | 5.0519 |
| pos_769 | C39 H71 O6 | TG(8:0/10:1/18:1) | Glycerolipids, GL | Triglyceride | 635.5245165 | 5.152 |
| pos_770 | C39 H74 O6 N1 | TG(16:0/10:1/10:1) | Glycerolipids, GL | Triglyceride | 652.5510655 | 8.7128 |
| pos_772 | C39 H71 O6 | TG(9:0/9:0/18:2) | Glycerolipids, GL | Triglyceride | 635.5245165 | 10.1697 |
| pos_774 | C39 H73 O5 | TG(12:1e/6:0/18:1) | Glycerolipids, GL | Triglyceride | 621.5452515 | 7.1973 |
| pos_775 | C39 H69 O6 | TG(6:0/12:1/18:2) | Glycerolipids, GL | Triglyceride | 633.5088665 | 3.5117 |
| pos_776 | C39 H69 O6 | TG(9:0/9:0/18:3) | Glycerolipids, GL | Triglyceride | 633.5088665 | 4.1926 |
| pos_778 | C39 H69 O6 | TG(16:0/6:0/14:3) | Glycerolipids, GL | Triglyceride | 633.5088665 | 6.8031 |
| pos_779 | C39 H69 O6 | TG(4:0/14:0/18:3) | Glycerolipids, GL | Triglyceride | 633.5088665 | 6.955 |
| pos_782 | C39 H71 O5 | TG(14:0e/10:0/12:3) | Glycerolipids, GL | Triglyceride | 619.5296015 | 5.3811 |
| pos_783 | C39 H71 O5 | TG(12:1e/6:0/18:2) | Glycerolipids, GL | Triglyceride | 619.5296015 | 6.1242 |
| pos_784 | C39 H71 O5 | TG(18:3e/9:0/9:0) | Glycerolipids, GL | Triglyceride | 619.5296015 | 6.5158 |
| pos_785 | C39 H71 O5 | TG(18:3e/8:0/10:0) | Glycerolipids, GL | Triglyceride | 619.5296015 | 6.8344 |
| pos_786 | C39 H71 O5 | TG(20:3e/8:0/8:0) | Glycerolipids, GL | Triglyceride | 619.5296015 | 8.3308 |
| pos_787 | C39 H71 O5 | TG(12:0e/6:0/18:3) | Glycerolipids, GL | Triglyceride | 619.5296015 | 10.0152 |
| pos_789 | C39 H67 O6 | TG(6:0/12:2/18:2) | Glycerolipids, GL | Triglyceride | 631.4932165 | 3.265 |
| pos_790 | C39 H67 O6 | TG(6:0/12:1/18:3) | Glycerolipids, GL | Triglyceride | 631.4932165 | 3.7284 |
| pos_791 | C39 H67 O6 | TG(6:0/12:3/18:1) | Glycerolipids, GL | Triglyceride | 631.4932165 | 10.6665 |
| pos_792 | C39 H67 O6 | TG(8:0/10:3/18:1) | Glycerolipids, GL | Triglyceride | 631.4932165 | 11.05 |
| pos_794 | C39 H69 O5 | TG(20:4e/8:0/8:0) | Glycerolipids, GL | Triglyceride | 617.5139515 | 5.6617 |
| pos_795 | C39 H69 O5 | TG(18:3e/8:0/10:1) | Glycerolipids, GL | Triglyceride | 617.5139515 | 5.824 |
| pos_797 | C39 H65 O6 | TG(16:1/10:2/10:2) | Glycerolipids, GL | Triglyceride | 629.4775665 | 5.2178 |
| pos_799 | C39 H67 O5 | TG(12:1e/6:0/18:4) | Glycerolipids, GL | Triglyceride | 615.4983015 | 3.735 |
| pos_800 | C39 H67 O5 | TG(14:1e/10:3/12:1) | Glycerolipids, GL | Triglyceride | 615.4983015 | 3.9114 |
| pos_801 | C39 H67 O5 | TG(20:4e/6:0/10:1) | Glycerolipids, GL | Triglyceride | 615.4983015 | 4.1685 |
| pos_802 | C39 H67 O5 | TG(14:1e/11:1/11:3) | Glycerolipids, GL | Triglyceride | 615.4983015 | 10.9587 |
| pos_803 | C39 H63 O6 | TG(18:4/6:0/12:2) | Glycerolipids, GL | Triglyceride | 627.4619165 | 2.066 |
| pos_804 | C39 H62 O6 Li1 | TG(6:0/12:4/18:2) | Glycerolipids, GL | Triglyceride | 633.4700915 | 2.7929 |
| pos_805 | C39 H62 O6 Li1 | TG(18:4/8:0/10:2) | Glycerolipids, GL | Triglyceride | 633.4700915 | 3.2423 |
| pos_806 | C39 H65 O5 | TG(14:1e/11:2/11:3) | Glycerolipids, GL | Triglyceride | 613.4826515 | 3.726 |
| pos_807 | C39 H60 O6 Li1 | TG(6:0/12:4/18:3) | Glycerolipids, GL | Triglyceride | 631.4544415 | 2.0726 |
| pos_808 | C39 H58 O6 Li1 | TG(18:4/6:0/12:4) | Glycerolipids, GL | Triglyceride | 629.4387915 | 2.0923 |
| pos_809 | C39 H58 O6 Li1 | TG(6:0/10:3/20:5) | Glycerolipids, GL | Triglyceride | 629.4387915 | 3.5192 |
| pos_810 | C39 H56 O6 Li1 | TG(6:0/10:4/20:5) | Glycerolipids, GL | Triglyceride | 627.4231415 | 3.176 |
| pos_811 | C40 H78 O6 N1 | TG(9:0/10:0/18:1) | Glycerolipids, GL | Triglyceride | 668.5823655 | 6.8611 |
| pos_812 | C40 H74 O6 K1 | TG(6:0/13:0/18:1) | Glycerolipids, GL | Triglyceride | 689.5116995 | 7.0288 |
| pos_813 | C40 H76 O6 N1 | TG(9:0/10:1/18:1) | Glycerolipids, GL | Triglyceride | 666.5667155 | 5.71 |
| pos_814 | C40 H76 O6 N1 | TG(9:0/10:0/18:2) | Glycerolipids, GL | Triglyceride | 666.5667155 | 6.1596 |
| pos_815 | C40 H72 O6 K1 | TG(6:0/13:0/18:2) | Glycerolipids, GL | Triglyceride | 687.4960495 | 6.3507 |
| pos_817 | C40 H70 O6 K1 | TG(6:0/13:0/18:3) | Glycerolipids, GL | Triglyceride | 685.4803995 | 4.443 |
| pos_818 | C40 H74 O6 N1 | TG(8:0/11:2/18:1) | Glycerolipids, GL | Triglyceride | 664.5510655 | 5.0515 |
| pos_819 | C40 H69 O6 | TG(8:0/11:1/18:3) | Glycerolipids, GL | Triglyceride | 645.5088665 | 4.2163 |
| pos_820 | C40 H72 O6 N1 | TG(9:0/10:2/18:2) | Glycerolipids, GL | Triglyceride | 662.5354155 | 5.1494 |
| pos_821 | C40 H68 O6 Li1 | TG(6:0/11:1/20:3) | Glycerolipids, GL | Triglyceride | 651.5170415 | 5.281 |
| pos_822 | C40 H68 O6 Li1 | TG(8:0/11:2/18:2) | Glycerolipids, GL | Triglyceride | 651.5170415 | 5.4687 |
| pos_824 | C40 H66 O6 Li1 | TG(16:0/10:2/11:3) | Glycerolipids, GL | Triglyceride | 649.5013915 | 4.5369 |
| pos_826 | C40 H66 O6 Li1 | TG(6:0/11:2/20:3) | Glycerolipids, GL | Triglyceride | 649.5013915 | 5.3562 |
| pos_829 | C40 H62 O6 Li1 | TG(16:0/10:4/11:3) | Glycerolipids, GL | Triglyceride | 645.4700915 | 4.4376 |
| pos_830 | C41 H82 O6 N1 | TG(20:0/6:0/12:0) | Glycerolipids, GL | Triglyceride | 684.6136655 | 8.7457 |
| pos_831 | C41 H77 O6 | TG(6:0/14:0/18:1) | Glycerolipids, GL | Triglyceride | 665.5714665 | 7.5744 |
| pos_832 | C41 H77 O6 | TG(20:1/6:0/12:0) | Glycerolipids, GL | Triglyceride | 665.5714665 | 7.7283 |
| pos_833 | C41 H80 O6 N1 | TG(18:0/10:0/10:1) | Glycerolipids, GL | Triglyceride | 682.5980155 | 10.4121 |
| pos_835 | C41 H78 O6 N1 | TG(4:0/16:0/18:2) | Glycerolipids, GL | Triglyceride | 680.5823655 | 6.8676 |
| pos_837 | C41 H78 O6 N1 | TG(9:0/11:1/18:1) | Glycerolipids, GL | Triglyceride | 680.5823655 | 9.4531 |
| pos_838 | C41 H78 O6 N1 | TG(18:0/10:1/10:1) | Glycerolipids, GL | Triglyceride | 680.5823655 | 9.6919 |
| pos_839 | C41 H77 O5 | TG(14:1e/6:0/18:1) | Glycerolipids, GL | Triglyceride | 649.5765515 | 9.7552 |
| pos_840 | C41 H76 O6 N1 | TG(6:0/14:0/18:3) | Glycerolipids, GL | Triglyceride | 678.5667155 | 5.033 |
| pos_841 | C41 H73 O6 | TG(18:1/10:1/10:1) | Glycerolipids, GL | Triglyceride | 661.5401665 | 8.7184 |
| pos_842 | C41 H76 O6 N1 | TG(10:0/10:1/18:2) | Glycerolipids, GL | Triglyceride | 678.5667155 | 8.9255 |
| pos_844 | C41 H75 O5 | TG(12:1e/6:0/20:2) | Glycerolipids, GL | Triglyceride | 647.5609015 | 7.58 |
| pos_845 | C41 H75 O5 | TG(14:0e/6:0/18:3) | Glycerolipids, GL | Triglyceride | 647.5609015 | 7.7261 |
| pos_846 | C41 H75 O5 | TG(12:1e/8:0/18:2) | Glycerolipids, GL | Triglyceride | 647.5609015 | 9.0418 |
| pos_847 | C41 H71 O6 | TG(4:0/16:1/18:3) | Glycerolipids, GL | Triglyceride | 659.5245165 | 5.931 |
| pos_848 | C41 H74 O6 N1 | TG(18:2/10:1/10:1) | Glycerolipids, GL | Triglyceride | 676.5510655 | 7.9565 |
| pos_851 | C41 H73 O5 | TG(18:3e/10:0/10:1) | Glycerolipids, GL | Triglyceride | 645.5452515 | 8.271 |
| pos_852 | C41 H73 O5 | TG(12:1e/6:0/20:3) | Glycerolipids, GL | Triglyceride | 645.5452515 | 9.8163 |
| pos_854 | C41 H72 O6 N1 | TG(8:0/12:2/18:3) | Glycerolipids, GL | Triglyceride | 674.5354155 | 7.308 |
| pos_855 | C41 H67 O6 | TG(6:0/14:4/18:2) | Glycerolipids, GL | Triglyceride | 655.4932165 | 4.7898 |
| pos_856 | C41 H65 O6 | TG(18:2/10:1/10:4) | Glycerolipids, GL | Triglyceride | 653.4775665 | 5.139 |
| pos_857 | C41 H65 O6 | TG(6:0/14:4/18:3) | Glycerolipids, GL | Triglyceride | 653.4775665 | 5.373 |
| pos_859 | C42 H84 O5 N1 | TG(14:0e/9:0/16:1) | Glycerolipids, GL | Triglyceride | 682.6344005 | 9.2402 |
| pos_860 | C42 H76 O6 K1 | TG(11:0/10:1/18:1) | Glycerolipids, GL | Triglyceride | 715.5273495 | 7.0759 |
| pos_861 | C42 H82 O5 N1 | TG(14:1e/9:0/16:1) | Glycerolipids, GL | Triglyceride | 680.6187505 | 8.1213 |
| pos_862 | C42 H74 O6 K1 | TG(18:1/10:1/11:1) | Glycerolipids, GL | Triglyceride | 713.5116995 | 6.2749 |
| pos_864 | C42 H72 O6 K1 | TG(18:1/10:1/11:2) | Glycerolipids, GL | Triglyceride | 711.4960495 | 4.9038 |
| pos_865 | C42 H72 O6 K1 | TG(9:0/12:2/18:2) | Glycerolipids, GL | Triglyceride | 711.4960495 | 5.7073 |
| pos_866 | C42 H70 O6 K1 | TG(16:0/11:3/12:2) | Glycerolipids, GL | Triglyceride | 709.4803995 | 3.9192 |
| pos_867 | C42 H70 O6 K1 | TG(18:2/10:1/11:2) | Glycerolipids, GL | Triglyceride | 709.4803995 | 4.3393 |
| pos_868 | C42 H70 O6 K1 | TG(10:0/11:3/18:2) | Glycerolipids, GL | Triglyceride | 709.4803995 | 4.4854 |
| pos_869 | C42 H68 O6 K1 | TG(18:2/10:1/11:3) | Glycerolipids, GL | Triglyceride | 707.4647495 | 3.9349 |
| pos_870 | C48 H84 O6 N2 | TG(10:0/11:4/18:2) | Glycerolipids, GL | Triglyceride | 784.6323895 | 6.3385 |
| pos_871 | C42 H69 O6 | TG(18:3/10:1/11:2) | Glycerolipids, GL | Triglyceride | 669.5088665 | 10.146 |
| pos_872 | C42 H66 O6 Li1 | TG(18:2/10:1/11:4) | Glycerolipids, GL | Triglyceride | 673.5013915 | 4.4364 |
| pos_873 | C42 H64 O6 Li1 | TG(18:2/10:2/11:4) | Glycerolipids, GL | Triglyceride | 671.4857415 | 3.826 |
| pos_875 | C43 H86 O6 N1 | TG(16:0/8:0/16:0) | Glycerolipids, GL | Triglyceride | 712.6449655 | 11.6787 |
| pos_876 | C43 H84 O6 N1 | TG(6:0/12:0/22:1) | Glycerolipids, GL | Triglyceride | 710.6293155 | 8.6537 |
| pos_878 | C43 H84 O5 N1 | TG(14:1e/9:0/17:1) | Glycerolipids, GL | Triglyceride | 694.6344005 | 8.578 |
| pos_879 | C43 H79 O5 | TG(18:3e/11:0/11:0) | Glycerolipids, GL | Triglyceride | 675.5922015 | 9.9615 |
| pos_880 | C43 H70 O6 Na1 | TG(18:2/10:2/12:2) | Glycerolipids, GL | Triglyceride | 705.5064615 | 5.3577 |
| pos_881 | C49 H86 O6 N2 | TG(18:2/10:3/12:1) | Glycerolipids, GL | Triglyceride | 798.6480395 | 6.3073 |
| pos_882 | C49 H86 O6 N2 | TG(4:0/18:3/18:3) | Glycerolipids, GL | Triglyceride | 798.6480395 | 6.5243 |
| pos_883 | C49 H86 O6 N2 | TG(18:2/11:2/11:2) | Glycerolipids, GL | Triglyceride | 798.6480395 | 7.2646 |
| pos_884 | C43 H68 O6 Na1 | TG(18:3/11:2/11:2) | Glycerolipids, GL | Triglyceride | 703.4908115 | 4.8749 |
| pos_885 | C49 H84 O6 N2 | TG(18:2/11:1/11:4) | Glycerolipids, GL | Triglyceride | 796.6323895 | 6.1197 |
| pos_886 | C49 H84 O6 N2 | TG(18:2/11:2/11:3) | Glycerolipids, GL | Triglyceride | 796.6323895 | 6.579 |
| pos_888 | C43 H66 O6 Na1 | TG(18:4/10:2/12:2) | Glycerolipids, GL | Triglyceride | 701.4751615 | 4.468 |
| pos_890 | C44 H74 O6 K1 | TG(18:1/11:1/12:3) | Glycerolipids, GL | Triglyceride | 737.5116995 | 5.7381 |
| pos_891 | C44 H72 O6 K1 | TG(18:2/11:2/12:2) | Glycerolipids, GL | Triglyceride | 735.4960495 | 5.1798 |
| pos_892 | C50 H88 O6 N2 | TG(16:0/11:3/14:3) | Glycerolipids, GL | Triglyceride | 812.6636895 | 8.5148 |
| pos_896 | C50 H86 O6 N2 | TG(18:1/11:2/12:4) | Glycerolipids, GL | Triglyceride | 810.6480395 | 7.0947 |
| pos_897 | C50 H88 O5 N2 | TG(12:1e/11:2/18:4) | Glycerolipids, GL | Triglyceride | 796.6687745 | 7.301 |
| pos_898 | C44 H68 O6 K1 | TG(18:2/11:2/12:4) | Glycerolipids, GL | Triglyceride | 731.4647495 | 5.2888 |
| pos_900 | C50 H86 O5 N2 | TG(12:1e/11:3/18:4) | Glycerolipids, GL | Triglyceride | 794.6531245 | 6.635 |
| pos_901 | C45 H88 O6 N1 | TG(16:0/8:0/18:1) | Glycerolipids, GL | Triglyceride | 738.6606155 | 11.6542 |
| pos_902 | C45 H66 O6 Na1 | TG(18:4/10:3/14:3) | Glycerolipids, GL | Triglyceride | 725.4751615 | 3.9376 |
| pos_903 | C45 H67 O6 | TG(18:3/12:3/12:4) | Glycerolipids, GL | Triglyceride | 703.4932165 | 4.884 |
| pos_905 | C45 H83 O6 | TG(16:0/10:2/16:0) | Glycerolipids, GL | Triglyceride | 719.6184165 | 9.2284 |
| pos_907 | C45 H86 O6 N1 | TG(16:0/8:0/18:2) | Glycerolipids, GL | Triglyceride | 736.6449655 | 10.98 |
| pos_909 | C45 H78 O5 Na1 | TG(12:0e/12:4/18:1) | Glycerolipids, GL | Triglyceride | 721.5741465 | 8.753 |
| pos_910 | C45 H76 O5 Na1 | TG(12:1e/10:3/20:2) | Glycerolipids, GL | Triglyceride | 719.5584965 | 3.932 |
| pos_911 | C45 H73 O6 | TG(16:0/12:3/14:4) | Glycerolipids, GL | Triglyceride | 709.5401665 | 2.574 |
| pos_912 | C45 H72 O6 Na1 | TG(18:2/12:2/12:3) | Glycerolipids, GL | Triglyceride | 731.5221115 | 5.387 |
| pos_913 | C51 H88 O6 N2 | TG(18:1/12:3/12:3) | Glycerolipids, GL | Triglyceride | 824.6636895 | 6.3061 |
| pos_914 | C51 H88 O6 N2 | TG(18:2/10:1/14:4) | Glycerolipids, GL | Triglyceride | 824.6636895 | 6.5291 |
| pos_915 | C51 H88 O6 N2 | TG(18:1/10:2/14:4) | Glycerolipids, GL | Triglyceride | 824.6636895 | 7.2859 |
| pos_916 | C51 H88 O6 N2 | TG(18:1/10:3/14:3) | Glycerolipids, GL | Triglyceride | 824.6636895 | 8.4756 |
| pos_918 | C45 H70 O6 Na1 | TG(18:1/10:3/14:4) | Glycerolipids, GL | Triglyceride | 729.5064615 | 4.8965 |
| pos_919 | C45 H70 O6 Na1 | TG(18:2/10:2/14:4) | Glycerolipids, GL | Triglyceride | 729.5064615 | 5.663 |
| pos_921 | C51 H86 O6 N2 | TG(18:4/6:0/18:4) | Glycerolipids, GL | Triglyceride | 822.6480395 | 6.583 |
| pos_922 | C45 H68 O6 Na1 | TG(18:3/12:3/12:3) | Glycerolipids, GL | Triglyceride | 727.4908115 | 4.3405 |
| pos_924 | C52 H104 O6 N2 | TG(11:0/16:0/16:0) | Glycerolipids, GL | Triglyceride | 852.7888895 | 14.0641 |
| pos_925 | C52 H102 O6 N2 | TG(16:0/11:1/16:0) | Glycerolipids, GL | Triglyceride | 850.7732395 | 13.4951 |
| pos_926 | C52 H100 O6 N2 | TG(16:0/9:0/18:2) | Glycerolipids, GL | Triglyceride | 848.7575895 | 13.0198 |
| pos_927 | C52 H100 O6 N2 | TG(16:0/11:2/16:0) | Glycerolipids, GL | Triglyceride | 848.7575895 | 13.2809 |
| pos_928 | C46 H83 O6 | TG(16:0/11:3/16:0) | Glycerolipids, GL | Triglyceride | 731.6184165 | 11.9791 |
| pos_929 | C46 H81 O6 | TG(16:0/11:4/16:0) | Glycerolipids, GL | Triglyceride | 729.6027665 | 9.5554 |
| pos_930 | C52 H90 O6 N2 | TG(18:1/11:3/14:3) | Glycerolipids, GL | Triglyceride | 838.6793395 | 8.5248 |
| pos_933 | C47 H92 O6 N1 | TG(16:0/10:0/18:1) | Glycerolipids, GL | Triglyceride | 766.6919155 | 12.301 |
| pos_934 | C53 H104 O6 N2 | TG(17:0/9:0/18:1) | Glycerolipids, GL | Triglyceride | 864.7888895 | 13.8925 |
| pos_935 | C47 H70 O6 Na1 | TG(18:2/12:4/14:4) | Glycerolipids, GL | Triglyceride | 753.5064615 | 5.1811 |
| pos_936 | C47 H90 O6 N1 | TG(8:0/18:1/18:1) | Glycerolipids, GL | Triglyceride | 764.6762655 | 11.6865 |
| pos_937 | C47 H85 O6 | TG(6:0/18:1/20:2) | Glycerolipids, GL | Triglyceride | 745.6340665 | 9.2198 |
| pos_938 | C47 H88 O6 N1 | TG(8:0/18:1/18:2) | Glycerolipids, GL | Triglyceride | 762.6606155 | 10.9773 |
| pos_939 | C47 H83 O6 | TG(8:0/18:2/18:2) | Glycerolipids, GL | Triglyceride | 743.6184165 | 6.5322 |
| pos_942 | C47 H80 O6 Li1 | TG(6:0/18:1/20:4) | Glycerolipids, GL | Triglyceride | 747.6109415 | 12.1653 |
| pos_943 | C47 H78 O6 Li1 | TG(16:0/10:4/18:2) | Glycerolipids, GL | Triglyceride | 745.5952915 | 6.5348 |
| pos_945 | C47 H72 O6 Na1 | TG(18:2/12:3/14:4) | Glycerolipids, GL | Triglyceride | 755.5221115 | 5.739 |
| pos_946 | C54 H108 O6 N2 | TG(11:0/16:0/18:0) | Glycerolipids, GL | Triglyceride | 880.8201895 | 14.4797 |
| pos_947 | C54 H108 O6 N2 | TG(16:0/6:0/23:0) | Glycerolipids, GL | Triglyceride | 880.8201895 | 14.6193 |
| pos_948 | C54 H106 O6 N2 | TG(11:0/16:0/18:1) | Glycerolipids, GL | Triglyceride | 878.8045395 | 14.0678 |
| pos_949 | C54 H104 O6 N2 | TG(11:0/16:0/18:2) | Glycerolipids, GL | Triglyceride | 876.7888895 | 13.6893 |
| pos_950 | C48 H87 O6 | TG(18:1/10:1/17:1) | Glycerolipids, GL | Triglyceride | 759.6497165 | 12.4962 |
| pos_952 | C54 H102 O6 N2 | TG(11:0/16:1/18:2) | Glycerolipids, GL | Triglyceride | 874.7732395 | 13.1842 |
| pos_953 | C54 H102 O6 N2 | TG(16:0/11:1/18:2) | Glycerolipids, GL | Triglyceride | 874.7732395 | 13.2967 |
| pos_955 | C54 H100 O6 N2 | TG(16:0/11:2/18:2) | Glycerolipids, GL | Triglyceride | 872.7575895 | 13.292 |
| pos_956 | C48 H82 O6 Li1 | TG(16:0/11:4/18:1) | Glycerolipids, GL | Triglyceride | 761.6265915 | 6.5367 |
| pos_957 | C48 H83 O6 | TG(18:4/9:0/18:1) | Glycerolipids, GL | Triglyceride | 755.6184165 | 9.5557 |
| pos_958 | C48 H83 O6 | TG(9:0/18:2/18:3) | Glycerolipids, GL | Triglyceride | 755.6184165 | 11.4092 |
| pos_959 | C48 H80 O6 Li1 | TG(9:0/18:3/18:3) | Glycerolipids, GL | Triglyceride | 759.6109415 | 4.4494 |
| pos_962 | C54 H92 O6 N2 | TG(18:4/9:0/18:4) | Glycerolipids, GL | Triglyceride | 864.6949895 | 8.6594 |
| pos_964 | C49 H98 O6 N1 | TG(16:0/14:0/16:0) | Glycerolipids, GL | Triglyceride | 796.7388655 | 13.5249 |
| pos_965 | C49 H96 O6 N1 | TG(16:0/12:0/18:1) | Glycerolipids, GL | Triglyceride | 794.7232155 | 12.9428 |
| pos_967 | C49 H78 O6 N1 | TG(18:2/14:4/14:4) | Glycerolipids, GL | Triglyceride | 776.5823655 | 5.612 |
| pos_968 | C49 H67 O6 | TG(22:6/12:4/12:4) | Glycerolipids, GL | Triglyceride | 751.4932165 | 4.501 |
| pos_969 | C49 H94 O6 N1 | TG(10:0/18:1/18:1) | Glycerolipids, GL | Triglyceride | 792.7075655 | 12.3802 |
| pos_970 | C55 H106 O6 N2 | TG(11:0/17:0/18:2) | Glycerolipids, GL | Triglyceride | 890.8045395 | 13.463 |
| pos_971 | C55 H106 O6 N2 | TG(16:0/12:1/18:1) | Glycerolipids, GL | Triglyceride | 890.8045395 | 13.8835 |
| pos_973 | C49 H92 O6 N1 | TG(10:0/18:1/18:2) | Glycerolipids, GL | Triglyceride | 790.6919155 | 11.6877 |
| pos_974 | C55 H104 O6 N2 | TG(17:0/11:1/18:2) | Glycerolipids, GL | Triglyceride | 888.7888895 | 13.449 |
| pos_975 | C49 H86 O6 Na1 | TG(18:1/10:1/18:2) | Glycerolipids, GL | Triglyceride | 793.6316615 | 7.729 |
| pos_976 | C49 H90 O6 N1 | TG(10:0/18:2/18:2) | Glycerolipids, GL | Triglyceride | 788.6762655 | 11.0548 |
| pos_977 | C49 H87 O6 | TG(16:0/12:2/18:2) | Glycerolipids, GL | Triglyceride | 771.6497165 | 11.526 |
| pos_978 | C49 H85 O6 | TG(16:0/10:0/20:5) | Glycerolipids, GL | Triglyceride | 769.6340665 | 8.3357 |
| pos_982 | C49 H80 O6 Li1 | TG(18:1/10:3/18:3) | Glycerolipids, GL | Triglyceride | 771.6109415 | 6.3211 |
| pos_983 | C49 H81 O6 | TG(18:3/10:2/18:2) | Glycerolipids, GL | Triglyceride | 765.6027665 | 10.1433 |
| pos_984 | C49 H80 O6 Li1 | TG(18:2/10:3/18:2) | Glycerolipids, GL | Triglyceride | 771.6109415 | 11.4112 |
| pos_986 | C49 H78 O6 Li1 | TG(18:2/10:4/18:2) | Glycerolipids, GL | Triglyceride | 769.5952915 | 11.0051 |
| pos_988 | C50 H100 O6 N1 | TG(15:0/16:0/16:0) | Glycerolipids, GL | Triglyceride | 810.7545155 | 12.3055 |
| pos_989 | C56 H112 O6 N2 | TG(11:0/18:0/18:0) | Glycerolipids, GL | Triglyceride | 908.8514895 | 14.8453 |
| pos_991 | C50 H98 O6 N1 | TG(15:0/14:0/18:1) | Glycerolipids, GL | Triglyceride | 808.7388655 | 11.4993 |
| pos_994 | C56 H110 O6 N2 | TG(11:0/18:0/18:1) | Glycerolipids, GL | Triglyceride | 906.8358395 | 14.5777 |
| pos_995 | C50 H92 O6 Na1 | TG(16:0/13:0/18:2) | Glycerolipids, GL | Triglyceride | 811.6786115 | 9.9107 |
| pos_997 | C56 H108 O6 N2 | TG(11:0/18:1/18:1) | Glycerolipids, GL | Triglyceride | 904.8201895 | 14.012 |
| pos_998 | C56 H106 O6 N2 | TG(11:0/18:1/18:2) | Glycerolipids, GL | Triglyceride | 902.8045395 | 13.761 |
| pos_999 | C56 H104 O6 N2 | TG(11:0/18:2/18:2) | Glycerolipids, GL | Triglyceride | 900.7888895 | 13.1442 |
| pos_1000 | C56 H102 O6 N2 | TG(18:1/11:2/18:2) | Glycerolipids, GL | Triglyceride | 898.7732395 | 9.369 |
| pos_1001 | C56 H102 O6 N2 | TG(18:2/11:1/18:2) | Glycerolipids, GL | Triglyceride | 898.7732395 | 12.643 |
| pos_1002 | C56 H104 O5 N2 | TG(18:2e/11:2/18:1) | Glycerolipids, GL | Triglyceride | 884.7939745 | 11.07 |
| pos_1004 | C56 H100 O6 N2 | TG(18:1/11:2/18:3) | Glycerolipids, GL | Triglyceride | 896.7575895 | 12.19 |
| pos_1006 | C50 H83 O6 | TG(18:3/11:2/18:2) | Glycerolipids, GL | Triglyceride | 779.6184165 | 10.1637 |
| pos_1007 | C56 H100 O5 N2 | TG(18:3e/11:1/18:3) | Glycerolipids, GL | Triglyceride | 880.7626745 | 10.016 |
| pos_1010 | C51 H96 O6 Na1 | TG(16:0/14:0/18:1) | Glycerolipids, GL | Triglyceride | 827.7099115 | 13.5047 |
| pos_1011 | C51 H94 O6 Na1 | TG(16:0/14:0/18:2) | Glycerolipids, GL | Triglyceride | 825.6942615 | 13.0295 |
| pos_1012 | C51 H93 O6 | TG(16:0/14:0/18:3) | Glycerolipids, GL | Triglyceride | 801.6966665 | 10.5601 |
| pos_1013 | C51 H92 O6 Na1 | TG(18:1/12:0/18:2) | Glycerolipids, GL | Triglyceride | 823.6786115 | 12.372 |
| pos_1015 | C51 H94 O6 N1 | TG(12:0/18:2/18:2) | Glycerolipids, GL | Triglyceride | 816.7075655 | 11.7863 |
| pos_1016 | C51 H89 O6 | TG(18:1/12:1/18:3) | Glycerolipids, GL | Triglyceride | 797.6653665 | 11.5277 |
| pos_1017 | C57 H104 O6 N2 | TG(27:0/10:3/11:2) | Glycerolipids, GL | Triglyceride | 912.7888895 | 12.672 |
| pos_1018 | C51 H87 O6 | TG(18:1/10:1/20:4) | Glycerolipids, GL | Triglyceride | 795.6497165 | 8.3344 |
| pos_1019 | C51 H87 O6 | TG(18:1/12:3/18:2) | Glycerolipids, GL | Triglyceride | 795.6497165 | 8.964 |
| pos_1020 | C51 H87 O6 | TG(18:2/12:2/18:2) | Glycerolipids, GL | Triglyceride | 795.6497165 | 10.925 |
| pos_1021 | C51 H85 O6 | TG(18:3/12:2/18:2) | Glycerolipids, GL | Triglyceride | 793.6340665 | 7.7356 |
| pos_1023 | C57 H98 O5 N2 | TG(12:1e/18:4/18:4) | Glycerolipids, GL | Triglyceride | 890.7470245 | 8.6137 |
| pos_1025 | C52 H104 O6 N1 | TG(16:0/16:0/17:0) | Glycerolipids, GL | Triglyceride | 838.7858155 | 14.3107 |
| pos_1026 | C58 H116 O6 N2 | TG(11:0/16:0/22:0) | Glycerolipids, GL | Triglyceride | 936.8827895 | 15.178 |
| pos_1027 | C52 H102 O6 N1 | TG(15:0/16:0/18:1) | Glycerolipids, GL | Triglyceride | 836.7701655 | 12.238 |
| pos_1028 | C58 H114 O6 N2 | TG(11:0/16:0/22:1) | Glycerolipids, GL | Triglyceride | 934.8671395 | 14.8177 |
| pos_1031 | C52 H96 O6 Na1 | TG(18:1/13:0/18:1) | Glycerolipids, GL | Triglyceride | 839.7099115 | 10.2992 |
| pos_1032 | C52 H96 O6 Na1 | TG(15:0/16:0/18:2) | Glycerolipids, GL | Triglyceride | 839.7099115 | 11.6689 |
| pos_1033 | C58 H112 O6 N2 | TG(11:0/18:1/20:1) | Glycerolipids, GL | Triglyceride | 932.8514895 | 14.3863 |
| pos_1038 | C52 H94 O6 Na1 | TG(18:1/13:0/18:2) | Glycerolipids, GL | Triglyceride | 837.6942615 | 9.682 |
| pos_1041 | C52 H100 O5 N1 | TG(14:0e/17:1/18:2) | Glycerolipids, GL | Triglyceride | 818.7596005 | 10.884 |
| pos_1042 | C52 H93 O6 | TG(18:3/10:1/21:0) | Glycerolipids, GL | Triglyceride | 813.6966665 | 8.6148 |
| pos_1045 | C52 H93 O6 | TG(16:0/11:3/22:1) | Glycerolipids, GL | Triglyceride | 813.6966665 | 10.4209 |
| pos_1046 | C52 H93 O6 | TG(16:0/11:2/22:2) | Glycerolipids, GL | Triglyceride | 813.6966665 | 10.6222 |
| pos_1047 | C58 H108 O6 N2 | TG(20:0/11:2/18:2) | Glycerolipids, GL | Triglyceride | 928.8201895 | 13.708 |
| pos_1048 | C58 H108 O6 N2 | TG(18:2/13:0/18:2) | Glycerolipids, GL | Triglyceride | 928.8201895 | 13.8737 |
| pos_1050 | C52 H91 O6 | TG(16:0/11:1/22:4) | Glycerolipids, GL | Triglyceride | 811.6810165 | 9.9228 |
| pos_1052 | C58 H104 O6 N2 | TG(18:3/13:0/18:3) | Glycerolipids, GL | Triglyceride | 924.7888895 | 9.4796 |
| pos_1054 | C58 H102 O6 N2 | TG(18:4/13:0/18:3) | Glycerolipids, GL | Triglyceride | 922.7732395 | 8.2587 |
| pos_1055 | C58 H102 O6 N2 | TG(18:2/14:4/17:1) | Glycerolipids, GL | Triglyceride | 922.7732395 | 8.742 |
| pos_1057 | C58 H102 O6 N2 | TG(18:1/11:2/20:4) | Glycerolipids, GL | Triglyceride | 922.7732395 | 10.4036 |
| pos_1058 | C58 H104 O5 N2 | TG(18:3e/14:4/17:0) | Glycerolipids, GL | Triglyceride | 908.7939745 | 10.779 |
| pos_1060 | C58 H100 O6 N2 | TG(18:4/13:0/18:4) | Glycerolipids, GL | Triglyceride | 920.7575895 | 7.291 |
| pos_1061 | C58 H100 O6 N2 | TG(18:3/11:3/20:2) | Glycerolipids, GL | Triglyceride | 920.7575895 | 8.0349 |
| pos_1066 | C58 H102 O5 N2 | TG(20:3e/11:2/18:3) | Glycerolipids, GL | Triglyceride | 906.7783245 | 9.565 |
| pos_1067 | C58 H102 O5 N2 | TG(20:4e/10:3/19:1) | Glycerolipids, GL | Triglyceride | 906.7783245 | 10.3389 |
| pos_1070 | C58 H100 O5 N2 | TG(16:2e/11:3/22:4) | Glycerolipids, GL | Triglyceride | 904.7626745 | 9.0744 |
| pos_1072 | C53 H102 O6 Na1 | TG(18:0/16:0/16:0) | Glycerolipids, GL | Triglyceride | 857.7568615 | 14.5329 |
| pos_1074 | C53 H104 O6 N1 | TG(15:0/17:0/18:1) | Glycerolipids, GL | Triglyceride | 850.7858155 | 12.2415 |
| pos_1075 | C59 H98 O6 N2 | TG(18:3/14:4/18:3) | Glycerolipids, GL | Triglyceride | 930.7419395 | 9.466 |
| pos_1077 | C53 H99 O6 | TG(16:0/16:1/18:1) | Glycerolipids, GL | Triglyceride | 831.7436165 | 11.6931 |
| pos_1078 | C53 H102 O6 N1 | TG(18:1/14:0/18:1) | Glycerolipids, GL | Triglyceride | 848.7701655 | 13.4953 |
| pos_1079 | C53 H96 O6 Na1 | TG(16:0/16:0/18:3) | Glycerolipids, GL | Triglyceride | 851.7099115 | 8.3633 |
| pos_1082 | C53 H97 O6 | TG(18:1/14:0/18:2) | Glycerolipids, GL | Triglyceride | 829.7279665 | 13.0205 |
| pos_1083 | C53 H98 O5 Na1 | TG(20:1e/11:2/19:0) | Glycerolipids, GL | Triglyceride | 837.7306465 | 11.416 |
| pos_1085 | C53 H95 O6 | TG(16:0/16:1/18:3) | Glycerolipids, GL | Triglyceride | 827.7123165 | 8.7571 |
| pos_1087 | C53 H95 O6 | TG(18:1/14:0/18:3) | Glycerolipids, GL | Triglyceride | 827.7123165 | 10.5967 |
| pos_1088 | C53 H95 O6 | TG(18:4/16:0/16:0) | Glycerolipids, GL | Triglyceride | 827.7123165 | 11.0031 |
| pos_1089 | C53 H94 O6 Na1 | TG(14:0/18:2/18:2) | Glycerolipids, GL | Triglyceride | 849.6942615 | 12.451 |
| pos_1092 | C54 H106 O6 N1 | TG(16:0/17:0/18:1) | Glycerolipids, GL | Triglyceride | 864.8014655 | 12.707 |
| pos_1093 | C60 H118 O6 N2 | TG(11:0/18:1/22:0) | Glycerolipids, GL | Triglyceride | 962.8984395 | 15.508 |
| pos_1094 | C60 H100 O6 N2 | TG(18:3/11:1/22:6) | Glycerolipids, GL | Triglyceride | 944.7575895 | 6.1781 |
| pos_1095 | C60 H100 O6 N2 | TG(22:5/11:3/18:2) | Glycerolipids, GL | Triglyceride | 944.7575895 | 6.5824 |
| pos_1097 | C60 H100 O6 N2 | TG(18:2/11:2/22:6) | Glycerolipids, GL | Triglyceride | 944.7575895 | 7.6103 |
| pos_1099 | C60 H102 O5 N2 | TG(18:3e/11:1/22:6) | Glycerolipids, GL | Triglyceride | 930.7783245 | 9.728 |
| pos_1100 | C60 H98 O6 N2 | TG(18:3/11:3/22:5) | Glycerolipids, GL | Triglyceride | 942.7419395 | 6.3153 |
| pos_1103 | C54 H104 O6 N1 | TG(15:0/18:1/18:1) | Glycerolipids, GL | Triglyceride | 862.7858155 | 12.1803 |
| pos_1104 | C54 H104 O6 N1 | TG(16:0/17:1/18:1) | Glycerolipids, GL | Triglyceride | 862.7858155 | 13.7713 |
| pos_1106 | C60 H116 O6 N2 | TG(11:0/18:1/22:1) | Glycerolipids, GL | Triglyceride | 960.8827895 | 14.7446 |
| pos_1109 | C54 H98 O6 Li1 | TG(16:1/17:1/18:1) | Glycerolipids, GL | Triglyceride | 849.7517915 | 11.7899 |
| pos_1110 | C54 H98 O6 Li1 | TG(16:0/17:1/18:2) | Glycerolipids, GL | Triglyceride | 849.7517915 | 12.5422 |
| pos_1115 | C54 H96 O6 Na1 | TG(16:0/17:1/18:3) | Glycerolipids, GL | Triglyceride | 863.7099115 | 11.1887 |
| pos_1116 | C54 H102 O5 N1 | TG(18:3e/15:0/18:1) | Glycerolipids, GL | Triglyceride | 844.7752505 | 10.9499 |
| pos_1118 | C54 H102 O5 N1 | TG(16:2e/17:0/18:2) | Glycerolipids, GL | Triglyceride | 844.7752505 | 11.6997 |
| pos_1121 | C54 H95 O6 | TG(29:1/10:2/12:2) | Glycerolipids, GL | Triglyceride | 839.7123165 | 9.6838 |
| pos_1122 | C54 H94 O6 Na1 | TG(15:0/18:2/18:3) | Glycerolipids, GL | Triglyceride | 861.6942615 | 10.3355 |
| pos_1124 | C54 H100 O5 N1 | TG(16:2e/17:1/18:2) | Glycerolipids, GL | Triglyceride | 842.7596005 | 10.3723 |
| pos_1127 | C54 H93 O6 | TG(18:3/11:1/22:2) | Glycerolipids, GL | Triglyceride | 837.6966665 | 7.9415 |
| pos_1128 | C54 H93 O6 | TG(22:2/11:2/18:2) | Glycerolipids, GL | Triglyceride | 837.6966665 | 8.6025 |
| pos_1129 | C60 H108 O6 N2 | TG(18:4/15:0/18:2) | Glycerolipids, GL | Triglyceride | 952.8201895 | 9.1244 |
| pos_1130 | C54 H93 O6 | TG(18:1/11:1/22:4) | Glycerolipids, GL | Triglyceride | 837.6966665 | 9.8841 |
| pos_1131 | C54 H98 O5 N1 | TG(20:4e/13:0/18:2) | Glycerolipids, GL | Triglyceride | 840.7439505 | 9.9919 |
| pos_1132 | C54 H91 O6 | TG(18:3/11:1/22:3) | Glycerolipids, GL | Triglyceride | 835.6810165 | 7.0539 |
| pos_1134 | C54 H91 O6 | TG(22:3/11:2/18:2) | Glycerolipids, GL | Triglyceride | 835.6810165 | 9.1694 |
| pos_1137 | C54 H91 O6 | TG(18:3/13:0/20:4) | Glycerolipids, GL | Triglyceride | 835.6810165 | 10.4177 |
| pos_1138 | C54 H91 O6 | TG(18:3/10:3/23:1) | Glycerolipids, GL | Triglyceride | 835.6810165 | 10.622 |
| pos_1140 | C60 H108 O5 N2 | TG(20:4e/13:0/18:3) | Glycerolipids, GL | Triglyceride | 936.8252745 | 11.557 |
| pos_1142 | C60 H104 O6 N2 | TG(18:4/15:0/18:4) | Glycerolipids, GL | Triglyceride | 948.7888895 | 7.7754 |
| pos_1144 | C60 H104 O6 N2 | TG(18:1/11:3/22:4) | Glycerolipids, GL | Triglyceride | 948.7888895 | 8.8804 |
| pos_1147 | C60 H106 O5 N2 | TG(18:3e/11:2/22:3) | Glycerolipids, GL | Triglyceride | 934.8096245 | 10.7805 |
| pos_1148 | C60 H102 O6 N2 | TG(18:3/11:1/22:5) | Glycerolipids, GL | Triglyceride | 946.7732395 | 6.5942 |
| pos_1149 | C60 H102 O6 N2 | TG(18:4/11:2/22:3) | Glycerolipids, GL | Triglyceride | 946.7732395 | 7.2673 |
| pos_1152 | C54 H86 O6 Li1 | TG(11:0/18:3/22:6) | Glycerolipids, GL | Triglyceride | 837.6578915 | 10.89 |
| pos_1153 | C54 H86 O6 Li1 | TG(11:0/18:4/22:5) | Glycerolipids, GL | Triglyceride | 837.6578915 | 11.139 |
| pos_1155 | C55 H110 O6 N1 | TG(18:0/16:0/18:0) | Glycerolipids, GL | Triglyceride | 880.8327655 | 14.9067 |
| pos_1156 | C55 H108 O6 N1 | TG(18:0/16:0/18:1) | Glycerolipids, GL | Triglyceride | 878.8171155 | 14.4812 |
| pos_1157 | C55 H88 O5 Li1 | TG(12:1e/18:3/22:6) | Glycerolipids, GL | Triglyceride | 835.6786265 | 10.416 |
| pos_1158 | C61 H100 O6 N2 | TG(18:2/12:3/22:6) | Glycerolipids, GL | Triglyceride | 956.7575895 | 8.722 |
| pos_1159 | C55 H84 O6 Li1 | TG(18:3/12:2/22:6) | Glycerolipids, GL | Triglyceride | 847.6422415 | 10.008 |
| pos_1164 | C55 H101 O6 | TG(18:0/16:0/18:3) | Glycerolipids, GL | Triglyceride | 857.7592665 | 12.06 |
| pos_1166 | C55 H98 O6 Na1 | TG(16:1/18:1/18:2) | Glycerolipids, GL | Triglyceride | 877.7255615 | 8.3512 |
| pos_1169 | C55 H96 O6 Na1 | TG(16:1/18:2/18:2) | Glycerolipids, GL | Triglyceride | 875.7099115 | 7.7984 |
| pos_1170 | C55 H96 O6 Li1 | TG(6:0/22:3/24:2) | Glycerolipids, GL | Triglyceride | 859.7361415 | 7.9936 |
| pos_1172 | C55 H97 O6 | TG(16:0/18:2/18:3) | Glycerolipids, GL | Triglyceride | 853.7279665 | 9.5505 |
| pos_1173 | C55 H100 O6 N1 | TG(18:3/17:1/17:1) | Glycerolipids, GL | Triglyceride | 870.7545155 | 10.438 |
| pos_1174 | C55 H99 O5 | TG(16:2e/16:0/20:3) | Glycerolipids, GL | Triglyceride | 839.7487015 | 11.836 |
| pos_1176 | C55 H94 O6 Li1 | TG(6:0/22:6/24:0) | Glycerolipids, GL | Triglyceride | 857.7204915 | 7.2672 |
| pos_1177 | C55 H95 O6 | TG(16:0/18:3/18:3) | Glycerolipids, GL | Triglyceride | 851.7123165 | 8.3567 |
| pos_1178 | C55 H98 O6 N1 | TG(18:4/16:0/18:2) | Glycerolipids, GL | Triglyceride | 868.7388655 | 9.6672 |
| pos_1179 | C55 H95 O6 | TG(16:1/18:2/18:3) | Glycerolipids, GL | Triglyceride | 851.7123165 | 9.8985 |
| pos_1180 | C55 H97 O5 | TG(18:3e/16:0/18:3) | Glycerolipids, GL | Triglyceride | 837.7330515 | 11.3589 |
| pos_1181 | C55 H92 O6 Li1 | TG(6:0/22:6/24:1) | Glycerolipids, GL | Triglyceride | 855.7048415 | 6.5705 |
| pos_1183 | C55 H93 O6 | TG(18:4/16:0/18:3) | Glycerolipids, GL | Triglyceride | 849.6966665 | 7.9754 |
| pos_1184 | C55 H96 O6 N1 | TG(16:1/18:3/18:3) | Glycerolipids, GL | Triglyceride | 866.7232155 | 11.5177 |
| pos_1185 | C55 H93 O6 | TG(14:0/18:2/20:5) | Glycerolipids, GL | Triglyceride | 849.6966665 | 12.438 |
| pos_1186 | C55 H95 O5 | TG(16:2e/18:2/18:3) | Glycerolipids, GL | Triglyceride | 835.7174015 | 10.6594 |
| pos_1189 | C61 H106 O5 N2 | TG(20:3e/14:3/18:3) | Glycerolipids, GL | Triglyceride | 946.8096245 | 9.531 |
| pos_1190 | C56 H108 O6 K1 | TG(29:0/9:0/15:0) | Glycerolipids, GL | Triglyceride | 915.7777495 | 10.782 |
| pos_1192 | C56 H110 O6 N1 | TG(20:0/15:0/18:1) | Glycerolipids, GL | Triglyceride | 892.8327655 | 13.5091 |
| pos_1193 | C56 H106 O6 Li1 | TG(16:0/18:1/19:0) | Glycerolipids, GL | Triglyceride | 881.8143915 | 14.0182 |
| pos_1194 | C56 H110 O6 N1 | TG(18:0/17:0/18:1) | Glycerolipids, GL | Triglyceride | 892.8327655 | 14.6528 |
| pos_1195 | C56 H88 O6 Li1 | TG(18:4/13:0/22:6) | Glycerolipids, GL | Triglyceride | 863.6735415 | 10.9311 |
| pos_1199 | C62 H120 O6 N2 | TG(11:0/18:1/24:1) | Glycerolipids, GL | Triglyceride | 988.9140895 | 15.0631 |
| pos_1201 | C56 H106 O6 N1 | TG(15:0/18:2/20:1) | Glycerolipids, GL | Triglyceride | 888.8014655 | 12.3112 |
| pos_1204 | C62 H118 O6 N2 | TG(18:1/11:1/24:1) | Glycerolipids, GL | Triglyceride | 986.8984395 | 14.807 |
| pos_1208 | C56 H104 O6 N1 | TG(17:0/18:1/18:3) | Glycerolipids, GL | Triglyceride | 886.7858155 | 13.071 |
| pos_1210 | C56 H98 O6 Na1 | TG(17:0/18:2/18:3) | Glycerolipids, GL | Triglyceride | 889.7255615 | 11.1599 |
| pos_1211 | C56 H102 O6 N1 | TG(18:1/17:1/18:3) | Glycerolipids, GL | Triglyceride | 884.7701655 | 11.3399 |
| pos_1212 | C56 H96 O6 Li1 | TG(18:3/17:1/18:2) | Glycerolipids, GL | Triglyceride | 871.7361415 | 9.5503 |
| pos_1213 | C56 H100 O6 N1 | TG(24:1/11:2/18:3) | Glycerolipids, GL | Triglyceride | 882.7545155 | 9.653 |
| pos_1215 | C56 H96 O6 Li1 | TG(16:0/17:1/20:5) | Glycerolipids, GL | Triglyceride | 871.7361415 | 12.5487 |
| pos_1217 | C56 H94 O6 Li1 | TG(18:4/17:1/18:2) | Glycerolipids, GL | Triglyceride | 869.7204915 | 9.867 |
| pos_1219 | C56 H92 O6 Li1 | TG(18:4/17:1/18:3) | Glycerolipids, GL | Triglyceride | 867.7048415 | 7.2848 |
| pos_1220 | C56 H92 O6 Li1 | TG(18:4/17:0/18:4) | Glycerolipids, GL | Triglyceride | 867.7048415 | 11.2475 |
| pos_1223 | C56 H90 O6 Li1 | TG(18:3/11:4/24:2) | Glycerolipids, GL | Triglyceride | 865.6891915 | 9.9518 |
| pos_1225 | C56 H90 O6 Li1 | TG(18:4/17:1/18:4) | Glycerolipids, GL | Triglyceride | 865.6891915 | 11.7526 |
| pos_1227 | C57 H114 O6 N1 | TG(18:0/16:0/20:0) | Glycerolipids, GL | Triglyceride | 908.8640655 | 15.2566 |
| pos_1228 | C57 H114 O6 N1 | TG(18:0/18:0/18:0) | Glycerolipids, GL | Triglyceride | 908.8640655 | 15.3988 |
| pos_1230 | C57 H112 O6 N1 | TG(20:0/16:0/18:1) | Glycerolipids, GL | Triglyceride | 906.8484155 | 14.848 |
| pos_1231 | C57 H112 O6 N1 | TG(18:0/18:0/18:1) | Glycerolipids, GL | Triglyceride | 906.8484155 | 14.9898 |
| pos_1232 | C57 H90 O6 Li1 | TG(18:4/18:3/18:3) | Glycerolipids, GL | Triglyceride | 877.6891915 | 7.6541 |
| pos_1233 | C57 H90 O6 Li1 | TG(18:4/18:2/18:4) | Glycerolipids, GL | Triglyceride | 877.6891915 | 7.9343 |
| pos_1235 | C57 H88 O6 Li1 | TG(18:4/18:3/18:4) | Glycerolipids, GL | Triglyceride | 875.6735415 | 7.0381 |
| pos_1236 | C57 H106 O6 Na1 | TG(18:0/18:1/18:1) | Glycerolipids, GL | Triglyceride | 909.7881615 | 11.4678 |
| pos_1242 | C57 H101 O6 | TG(18:1/18:1/18:3) | Glycerolipids, GL | Triglyceride | 881.7592665 | 9.2301 |
| pos_1243 | C57 H101 O6 | TG(18:4/18:0/18:1) | Glycerolipids, GL | Triglyceride | 881.7592665 | 10.7381 |
| pos_1246 | C57 H99 O6 | TG(18:4/18:1/18:1) | Glycerolipids, GL | Triglyceride | 879.7436165 | 8.7699 |
| pos_1247 | C57 H99 O6 | TG(18:1/18:2/18:3) | Glycerolipids, GL | Triglyceride | 879.7436165 | 9.5634 |
| pos_1248 | C57 H99 O6 | TG(16:0/18:1/20:5) | Glycerolipids, GL | Triglyceride | 879.7436165 | 13.698 |
| pos_1249 | C57 H101 O5 | TG(18:3e/18:1/18:2) | Glycerolipids, GL | Triglyceride | 865.7643515 | 11.2 |
| pos_1250 | C57 H97 O6 | TG(18:4/18:1/18:2) | Glycerolipids, GL | Triglyceride | 877.7279665 | 8.3515 |
| pos_1252 | C57 H97 O6 | TG(18:1/18:3/18:3) | Glycerolipids, GL | Triglyceride | 877.7279665 | 10.1521 |
| pos_1253 | C57 H99 O5 | TG(18:3e/18:1/18:3) | Glycerolipids, GL | Triglyceride | 863.7487015 | 13.1364 |
| pos_1254 | C57 H95 O6 | TG(18:3/18:2/18:3) | Glycerolipids, GL | Triglyceride | 875.7123165 | 7.7466 |
| pos_1255 | C57 H95 O6 | TG(18:4/18:2/18:2) | Glycerolipids, GL | Triglyceride | 875.7123165 | 9.7793 |
| pos_1258 | C57 H93 O6 | TG(18:3/18:3/18:3) | Glycerolipids, GL | Triglyceride | 873.6966665 | 5.6706 |
| pos_1259 | C57 H93 O6 | TG(18:4/18:2/18:3) | Glycerolipids, GL | Triglyceride | 873.6966665 | 7.2911 |
| pos_1260 | C57 H95 O5 | TG(18:3e/18:2/18:4) | Glycerolipids, GL | Triglyceride | 859.7174015 | 10.2106 |
| pos_1261 | C57 H94 O5 Na1 | TG(18:3e/18:3/18:3) | Glycerolipids, GL | Triglyceride | 881.6993465 | 11.6095 |
| pos_1262 | C58 H114 O6 N1 | TG(20:0/17:0/18:1) | Glycerolipids, GL | Triglyceride | 920.8640655 | 14.8542 |
| pos_1263 | C58 H114 O6 N1 | TG(16:0/18:1/21:0) | Glycerolipids, GL | Triglyceride | 920.8640655 | 15.0187 |
| pos_1264 | C64 H126 O6 N2 | TG(26:0/11:0/18:1) | Glycerolipids, GL | Triglyceride | 1018.961039 | 15.7243 |
| pos_1266 | C58 H92 O6 Li1 | TG(22:4/11:1/22:5) | Glycerolipids, GL | Triglyceride | 891.7048415 | 6.5867 |
| pos_1269 | C58 H92 O6 Li1 | TG(22:6/10:4/23:0) | Glycerolipids, GL | Triglyceride | 891.7048415 | 10.0014 |
| pos_1271 | C58 H112 O6 N1 | TG(19:0/18:1/18:1) | Glycerolipids, GL | Triglyceride | 918.8484155 | 13.4155 |
| pos_1273 | C64 H124 O6 N2 | TG(11:0/22:1/22:1) | Glycerolipids, GL | Triglyceride | 1016.945389 | 15.3333 |
| pos_1274 | C58 H110 O6 N1 | TG(15:0/18:2/22:1) | Glycerolipids, GL | Triglyceride | 916.8327655 | 12.9456 |
| pos_1275 | C58 H106 O6 Li1 | TG(19:0/18:1/18:2) | Glycerolipids, GL | Triglyceride | 905.8143915 | 13.5662 |
| pos_1276 | C58 H110 O6 N1 | TG(19:1/18:1/18:1) | Glycerolipids, GL | Triglyceride | 916.8327655 | 13.9522 |
| pos_1277 | C58 H112 O5 N1 | TG(16:2e/18:1/21:0) | Glycerolipids, GL | Triglyceride | 902.8535005 | 13.221 |
| pos_1278 | C58 H108 O6 N1 | TG(19:0/18:2/18:2) | Glycerolipids, GL | Triglyceride | 914.8171155 | 12.068 |
| pos_1279 | C58 H108 O6 N1 | TG(19:0/18:1/18:3) | Glycerolipids, GL | Triglyceride | 914.8171155 | 12.2 |
| pos_1280 | C58 H104 O6 Li1 | TG(19:1/18:1/18:2) | Glycerolipids, GL | Triglyceride | 903.7987415 | 12.484 |
| pos_1281 | C64 H120 O6 N2 | TG(15:0/18:3/22:1) | Glycerolipids, GL | Triglyceride | 1012.914089 | 14.89 |
| pos_1283 | C58 H102 O6 Li1 | TG(19:1/18:1/18:3) | Glycerolipids, GL | Triglyceride | 901.7830915 | 10.6599 |
| pos_1284 | C58 H102 O6 Li1 | TG(19:1/18:2/18:2) | Glycerolipids, GL | Triglyceride | 901.7830915 | 12.5468 |
| pos_1285 | C58 H100 O6 Li1 | TG(26:0/11:4/18:2) | Glycerolipids, GL | Triglyceride | 899.7674415 | 9.555 |
| pos_1288 | C58 H100 O6 Li1 | TG(19:1/18:1/18:4) | Glycerolipids, GL | Triglyceride | 899.7674415 | 11.9633 |
| pos_1291 | C58 H98 O6 Li1 | TG(19:1/18:3/18:3) | Glycerolipids, GL | Triglyceride | 897.7517915 | 9.5561 |
| pos_1292 | C58 H96 O6 Li1 | TG(11:0/22:4/22:4) | Glycerolipids, GL | Triglyceride | 895.7361415 | 6.9289 |
| pos_1296 | C58 H96 O6 Li1 | TG(20:5/17:1/18:2) | Glycerolipids, GL | Triglyceride | 895.7361415 | 9.705 |
| pos_1297 | C58 H96 O6 Li1 | TG(18:3/17:1/20:4) | Glycerolipids, GL | Triglyceride | 895.7361415 | 9.8238 |
| pos_1298 | C58 H100 O6 N1 | TG(15:0/18:2/22:6) | Glycerolipids, GL | Triglyceride | 906.7545155 | 11.6788 |
| pos_1301 | C64 H114 O5 N2 | TG(18:3e/18:4/19:1) | Glycerolipids, GL | Triglyceride | 990.8722245 | 13.511 |
| pos_1302 | C58 H94 O6 Li1 | TG(11:0/22:4/22:5) | Glycerolipids, GL | Triglyceride | 893.7204915 | 6.5978 |
| pos_1303 | C58 H94 O6 Li1 | TG(18:3/17:1/20:5) | Glycerolipids, GL | Triglyceride | 893.7204915 | 7.2763 |
| pos_1304 | C58 H94 O6 Li1 | TG(22:4/11:1/22:4) | Glycerolipids, GL | Triglyceride | 893.7204915 | 7.7527 |
| pos_1305 | C58 H94 O6 Li1 | TG(19:1/18:4/18:4) | Glycerolipids, GL | Triglyceride | 893.7204915 | 8.9302 |
| pos_1306 | C58 H94 O6 Li1 | TG(18:4/15:0/22:5) | Glycerolipids, GL | Triglyceride | 893.7204915 | 9.992 |
| pos_1308 | C59 H114 O6 Na1 | TG(16:0/16:0/24:0) | Glycerolipids, GL | Triglyceride | 941.8507615 | 15.592 |
| pos_1309 | C59 H112 O6 K1 | TG(16:0/16:0/24:1) | Glycerolipids, GL | Triglyceride | 955.8090495 | 10.7589 |
| pos_1311 | C59 H92 O6 K1 | TG(18:4/18:2/20:5) | Glycerolipids, GL | Triglyceride | 935.6525495 | 6.4351 |
| pos_1312 | C59 H93 O6 | TG(18:3/18:3/20:5) | Glycerolipids, GL | Triglyceride | 897.6966665 | 11.6842 |
| pos_1313 | C59 H91 O6 | TG(18:4/18:3/20:5) | Glycerolipids, GL | Triglyceride | 895.6810165 | 11.157 |
| pos_1314 | C59 H110 O6 K1 | TG(18:0/18:1/20:1) | Glycerolipids, GL | Triglyceride | 953.7933995 | 10.1387 |
| pos_1317 | C59 H114 O6 N1 | TG(20:0/18:1/18:1) | Glycerolipids, GL | Triglyceride | 932.8640655 | 14.8117 |
| pos_1318 | C59 H108 O6 K1 | TG(20:1/18:1/18:1) | Glycerolipids, GL | Triglyceride | 951.7777495 | 10.4633 |
| pos_1323 | C59 H110 O6 N1 | TG(20:0/18:2/18:2) | Glycerolipids, GL | Triglyceride | 928.8327655 | 14.2418 |
| pos_1324 | C59 H105 O6 | TG(20:0/18:2/18:3) | Glycerolipids, GL | Triglyceride | 909.7905665 | 10.0066 |
| pos_1325 | C59 H105 O6 | TG(18:1/18:2/20:2) | Glycerolipids, GL | Triglyceride | 909.7905665 | 11.667 |
| pos_1326 | C59 H105 O6 | TG(20:1/18:1/18:3) | Glycerolipids, GL | Triglyceride | 909.7905665 | 11.884 |
| pos_1328 | C59 H104 O6 Na1 | TG(20:1/18:2/18:2) | Glycerolipids, GL | Triglyceride | 931.7725115 | 13.8768 |
| pos_1329 | C59 H107 O5 | TG(18:3e/18:2/20:0) | Glycerolipids, GL | Triglyceride | 895.8113015 | 13.236 |
| pos_1331 | C59 H103 O6 | TG(20:0/18:3/18:3) | Glycerolipids, GL | Triglyceride | 907.7749165 | 10.3366 |
| pos_1333 | C59 H106 O6 N1 | TG(18:1/18:1/20:4) | Glycerolipids, GL | Triglyceride | 924.8014655 | 12.631 |
| pos_1334 | C59 H106 O6 N1 | TG(20:2/18:2/18:2) | Glycerolipids, GL | Triglyceride | 924.8014655 | 13.1258 |
| pos_1335 | C59 H101 O6 | TG(20:1/18:3/18:3) | Glycerolipids, GL | Triglyceride | 905.7592665 | 10.613 |
| pos_1336 | C59 H101 O6 | TG(18:4/18:2/20:1) | Glycerolipids, GL | Triglyceride | 905.7592665 | 11.0361 |
| pos_1337 | C59 H101 O6 | TG(18:1/18:1/20:5) | Glycerolipids, GL | Triglyceride | 905.7592665 | 13.8382 |
| pos_1339 | C59 H99 O6 | TG(18:1/18:2/20:5) | Glycerolipids, GL | Triglyceride | 903.7436165 | 13.43 |
| pos_1341 | C59 H97 O6 | TG(20:5/18:2/18:2) | Glycerolipids, GL | Triglyceride | 901.7279665 | 12.6506 |
| pos_1343 | C60 H118 O6 N1 | TG(16:0/18:1/23:0) | Glycerolipids, GL | Triglyceride | 948.8953655 | 15.3441 |
| pos_1345 | C60 H96 O6 Na1 | TG(18:4/17:1/22:5) | Glycerolipids, GL | Triglyceride | 935.7099115 | 11.5603 |
| pos_1351 | C60 H116 O6 N1 | TG(16:0/18:2/23:0) | Glycerolipids, GL | Triglyceride | 946.8797155 | 15.1014 |
| pos_1352 | C60 H118 O5 N1 | TG(16:1e/18:1/23:0) | Glycerolipids, GL | Triglyceride | 932.9004505 | 13.9576 |
| pos_1353 | C60 H114 O6 N1 | TG(18:1/18:2/21:0) | Glycerolipids, GL | Triglyceride | 944.8640655 | 12.577 |
| pos_1354 | C60 H114 O6 N1 | TG(18:1/18:1/21:1) | Glycerolipids, GL | Triglyceride | 944.8640655 | 14.3702 |
| pos_1355 | C60 H116 O5 N1 | TG(14:1e/19:1/24:1) | Glycerolipids, GL | Triglyceride | 930.8848005 | 13.7687 |
| pos_1356 | C60 H116 O5 N1 | TG(16:2e/18:1/23:0) | Glycerolipids, GL | Triglyceride | 930.8848005 | 14.1709 |
| pos_1357 | C60 H108 O6 Na1 | TG(18:1/18:2/21:1) | Glycerolipids, GL | Triglyceride | 947.8038115 | 9.537 |
| pos_1358 | C60 H112 O6 N1 | TG(19:1/18:2/20:1) | Glycerolipids, GL | Triglyceride | 942.8484155 | 12.6634 |
| pos_1359 | C60 H110 O5 Na1 | TG(20:2e/18:1/19:1) | Glycerolipids, GL | Triglyceride | 933.8245465 | 11.728 |
| pos_1361 | C60 H106 O6 Li1 | TG(19:0/18:1/20:4) | Glycerolipids, GL | Triglyceride | 929.8143915 | 13.0361 |
| pos_1363 | C60 H110 O6 N1 | TG(18:2/18:2/21:1) | Glycerolipids, GL | Triglyceride | 940.8327655 | 13.9038 |
| pos_1366 | C60 H102 O6 Li1 | TG(18:3/18:3/21:1) | Glycerolipids, GL | Triglyceride | 925.7830915 | 12.2861 |
| pos_1367 | C60 H102 O6 Li1 | TG(18:4/18:2/21:1) | Glycerolipids, GL | Triglyceride | 925.7830915 | 12.4417 |
| pos_1368 | C66 H118 O6 N2 | TG(18:1/17:1/22:5) | Glycerolipids, GL | Triglyceride | 1034.898439 | 12.889 |
| pos_1369 | C60 H102 O6 Li1 | TG(19:1/16:0/22:6) | Glycerolipids, GL | Triglyceride | 925.7830915 | 13.0286 |
| pos_1371 | C66 H116 O6 N2 | TG(18:4/18:3/21:1) | Glycerolipids, GL | Triglyceride | 1032.882789 | 12.2612 |
| pos_1373 | C60 H98 O6 Li1 | TG(18:2/17:1/22:6) | Glycerolipids, GL | Triglyceride | 921.7517915 | 12.0498 |
| pos_1374 | C61 H122 O6 N1 | TG(26:0/16:0/16:0) | Glycerolipids, GL | Triglyceride | 964.9266655 | 15.8954 |
| pos_1375 | C61 H116 O6 K1 | TG(28:0/6:0/24:1) | Glycerolipids, GL | Triglyceride | 983.8403495 | 14.61 |
| pos_1376 | C61 H120 O6 N1 | TG(16:0/18:1/24:0) | Glycerolipids, GL | Triglyceride | 962.9110155 | 15.4931 |
| pos_1377 | C61 H96 O6 K1 | TG(18:1/20:5/20:5) | Glycerolipids, GL | Triglyceride | 963.6838495 | 7.1858 |
| pos_1378 | C61 H94 O6 K1 | TG(18:4/18:2/22:6) | Glycerolipids, GL | Triglyceride | 961.6681995 | 6.496 |
| pos_1379 | C61 H92 O6 K1 | TG(18:4/18:3/22:6) | Glycerolipids, GL | Triglyceride | 959.6525495 | 5.8261 |
| pos_1380 | C61 H114 O6 Na1 | TG(16:0/18:1/24:1) | Glycerolipids, GL | Triglyceride | 965.8507615 | 15.1344 |
| pos_1381 | C61 H118 O6 N1 | TG(16:0/18:2/24:0) | Glycerolipids, GL | Triglyceride | 960.8953655 | 15.2673 |
| pos_1384 | C61 H112 O6 Na1 | TG(18:1/18:1/22:1) | Glycerolipids, GL | Triglyceride | 963.8351115 | 14.7482 |
| pos_1387 | C61 H109 O6 | TG(20:1/18:3/20:1) | Glycerolipids, GL | Triglyceride | 937.8218665 | 11.5577 |
| pos_1389 | C61 H109 O6 | TG(22:0/18:2/18:3) | Glycerolipids, GL | Triglyceride | 937.8218665 | 12.9533 |
| pos_1390 | C61 H112 O6 N1 | TG(22:1/18:2/18:2) | Glycerolipids, GL | Triglyceride | 954.8484155 | 14.169 |
| pos_1395 | C61 H105 O6 | TG(22:3/18:2/18:2) | Glycerolipids, GL | Triglyceride | 933.7905665 | 9.5636 |
| pos_1397 | C61 H101 O6 | TG(18:3/18:3/22:3) | Glycerolipids, GL | Triglyceride | 929.7592665 | 8.4237 |
| pos_1398 | C62 H122 O6 N1 | TG(25:0/16:0/18:1) | Glycerolipids, GL | Triglyceride | 976.9266655 | 15.6386 |
| pos_1400 | C62 H120 O6 N1 | TG(25:1/16:0/18:1) | Glycerolipids, GL | Triglyceride | 974.9110155 | 15.2897 |
| pos_1401 | C62 H120 O6 N1 | TG(25:0/16:0/18:2) | Glycerolipids, GL | Triglyceride | 974.9110155 | 15.4201 |
| pos_1402 | C62 H118 O6 N1 | TG(18:1/18:2/23:0) | Glycerolipids, GL | Triglyceride | 972.8953655 | 14.637 |
| pos_1403 | C62 H118 O6 N1 | TG(18:1/18:1/23:1) | Glycerolipids, GL | Triglyceride | 972.8953655 | 14.9294 |
| pos_1404 | C62 H112 O6 Na1 | TG(18:1/18:2/23:1) | Glycerolipids, GL | Triglyceride | 975.8351115 | 10.7828 |
| pos_1405 | C62 H116 O6 N1 | TG(18:2/18:2/23:0) | Glycerolipids, GL | Triglyceride | 970.8797155 | 14.8203 |
| pos_1406 | C62 H114 O5 K1 | TG(18:3e/18:1/23:0) | Glycerolipids, GL | Triglyceride | 977.8297845 | 15.487 |
| pos_1407 | C62 H110 O6 Na1 | TG(18:2/18:2/23:1) | Glycerolipids, GL | Triglyceride | 973.8194615 | 9.548 |
| pos_1408 | C62 H112 O5 K1 | TG(18:3e/18:2/23:0) | Glycerolipids, GL | Triglyceride | 975.8141345 | 15.268 |
| pos_1409 | C62 H107 O6 | TG(18:3/18:3/23:1) | Glycerolipids, GL | Triglyceride | 947.8062165 | 9.527 |
| pos_1411 | C62 H107 O6 | TG(19:1/20:1/20:5) | Glycerolipids, GL | Triglyceride | 947.8062165 | 12.393 |
| pos_1413 | C62 H105 O6 | TG(29:1/12:4/18:3) | Glycerolipids, GL | Triglyceride | 945.7905665 | 9.883 |
| pos_1414 | C62 H104 O6 Li1 | TG(19:1/18:1/22:6) | Glycerolipids, GL | Triglyceride | 951.7987415 | 13.0212 |
| pos_1416 | C62 H102 O6 Li1 | TG(19:1/18:2/22:6) | Glycerolipids, GL | Triglyceride | 949.7830915 | 12.9524 |
| pos_1417 | C63 H126 O6 N1 | TG(26:0/16:0/18:0) | Glycerolipids, GL | Triglyceride | 992.9579655 | 16.2125 |
| pos_1418 | C63 H124 O6 N1 | TG(26:0/16:0/18:1) | Glycerolipids, GL | Triglyceride | 990.9423155 | 15.7705 |
| pos_1420 | C63 H98 O6 K1 | TG(18:2/20:4/22:6) | Glycerolipids, GL | Triglyceride | 989.6994995 | 7.352 |
| pos_1421 | C63 H122 O6 N1 | TG(18:1/18:1/24:0) | Glycerolipids, GL | Triglyceride | 988.9266655 | 15.4483 |
| pos_1422 | C63 H122 O6 N1 | TG(26:0/16:0/18:2) | Glycerolipids, GL | Triglyceride | 988.9266655 | 15.5742 |
| pos_1424 | C63 H120 O6 N1 | TG(18:1/18:1/24:1) | Glycerolipids, GL | Triglyceride | 986.9110155 | 15.0604 |
| pos_1425 | C63 H115 O6 | TG(18:1/18:3/24:0) | Glycerolipids, GL | Triglyceride | 967.8688165 | 13.9024 |
| pos_1426 | C63 H118 O6 N1 | TG(18:1/18:2/24:1) | Glycerolipids, GL | Triglyceride | 984.8953655 | 14.8079 |
| pos_1427 | C63 H118 O6 N1 | TG(24:0/18:2/18:2) | Glycerolipids, GL | Triglyceride | 984.8953655 | 14.9988 |
| pos_1428 | C63 H114 O6 Li1 | TG(18:1/18:1/24:2) | Glycerolipids, GL | Triglyceride | 973.8769915 | 15.488 |
| pos_1430 | C63 H113 O6 | TG(24:0/18:2/18:3) | Glycerolipids, GL | Triglyceride | 965.8531665 | 13.5037 |
| pos_1431 | C63 H116 O6 N1 | TG(24:1/18:2/18:2) | Glycerolipids, GL | Triglyceride | 982.8797155 | 14.5599 |
| pos_1432 | C63 H114 O5 K1 | TG(18:2e/18:2/24:1) | Glycerolipids, GL | Triglyceride | 989.8297845 | 15.3723 |
| pos_1434 | C63 H111 O6 | TG(18:4/20:1/22:1) | Glycerolipids, GL | Triglyceride | 963.8375165 | 12.9222 |
| pos_1435 | C63 H114 O6 N1 | TG(24:1/18:2/18:3) | Glycerolipids, GL | Triglyceride | 980.8640655 | 14.2839 |
| pos_1439 | C64 H126 O6 N1 | TG(27:0/16:0/18:1) | Glycerolipids, GL | Triglyceride | 1004.957965 | 15.8895 |
| pos_1440 | C64 H124 O6 N1 | TG(27:1/16:0/18:1) | Glycerolipids, GL | Triglyceride | 1002.942315 | 15.438 |
| pos_1443 | C64 H122 O6 N1 | TG(25:0/18:1/18:2) | Glycerolipids, GL | Triglyceride | 1000.926665 | 15.3887 |
| pos_1444 | C64 H120 O6 N1 | TG(25:1/18:1/18:2) | Glycerolipids, GL | Triglyceride | 998.9110155 | 14.9798 |
| pos_1445 | C64 H120 O6 N1 | TG(25:0/18:2/18:2) | Glycerolipids, GL | Triglyceride | 998.9110155 | 15.1592 |
| pos_1446 | C64 H118 O6 N1 | TG(25:1/18:2/18:2) | Glycerolipids, GL | Triglyceride | 996.8953655 | 14.7157 |
| pos_1447 | C64 H116 O5 Na1 | TG(20:4e/18:1/23:0) | Glycerolipids, GL | Triglyceride | 987.8714965 | 13.019 |
| pos_1448 | C64 H111 O6 | TG(18:1/21:0/22:6) | Glycerolipids, GL | Triglyceride | 975.8375165 | 10.7766 |
| pos_1450 | C64 H111 O5 | TG(20:4e/18:3/23:1) | Glycerolipids, GL | Triglyceride | 959.8426015 | 12.43 |
| pos_1451 | C65 H130 O6 N1 | TG(26:0/18:0/18:0) | Glycerolipids, GL | Triglyceride | 1020.989265 | 16.678 |
| pos_1452 | C65 H128 O6 N1 | TG(26:0/18:0/18:1) | Glycerolipids, GL | Triglyceride | 1018.973615 | 16.0294 |
| pos_1453 | C65 H104 O6 K1 | TG(22:3/18:2/22:6) | Glycerolipids, GL | Triglyceride | 1019.746449 | 9.306 |
| pos_1454 | C65 H126 O6 N1 | TG(26:0/18:1/18:1) | Glycerolipids, GL | Triglyceride | 1016.957965 | 15.7244 |
| pos_1455 | C65 H120 O6 Na1 | TG(26:1/18:1/18:1) | Glycerolipids, GL | Triglyceride | 1019.897711 | 15.331 |
| pos_1456 | C65 H124 O6 N1 | TG(26:0/18:1/18:2) | Glycerolipids, GL | Triglyceride | 1014.942315 | 15.5325 |
| pos_1458 | C65 H119 O6 | TG(26:0/18:1/18:3) | Glycerolipids, GL | Triglyceride | 995.9001165 | 14.339 |
| pos_1459 | C65 H118 O6 Na1 | TG(22:1/18:2/22:1) | Glycerolipids, GL | Triglyceride | 1017.882061 | 15.0966 |
| pos_1460 | C65 H118 O6 Na1 | TG(26:0/18:2/18:2) | Glycerolipids, GL | Triglyceride | 1017.882061 | 15.3142 |
| pos_1462 | C65 H120 O6 N1 | TG(18:3/22:1/22:1) | Glycerolipids, GL | Triglyceride | 1010.911015 | 14.8904 |
| pos_1463 | C65 H117 O6 | TG(26:0/18:1/18:4) | Glycerolipids, GL | Triglyceride | 993.8844665 | 15.589 |
| pos_1464 | C65 H115 O6 | TG(18:4/22:1/22:1) | Glycerolipids, GL | Triglyceride | 991.8688165 | 13.4948 |
| pos_1465 | C65 H118 O6 N1 | TG(18:3/22:1/22:2) | Glycerolipids, GL | Triglyceride | 1008.895365 | 14.6058 |
| pos_1466 | C65 H115 O5 | TG(18:0e/22:1/22:6) | Glycerolipids, GL | Triglyceride | 975.8739015 | 13.891 |
| pos_1467 | C66 H130 O6 N1 | TG(27:0/18:0/18:1) | Glycerolipids, GL | Triglyceride | 1032.989265 | 16.0207 |
| pos_1468 | C66 H128 O6 N1 | TG(27:0/18:1/18:1) | Glycerolipids, GL | Triglyceride | 1030.973615 | 15.7209 |
| pos_1469 | C66 H126 O6 N1 | TG(27:0/18:1/18:2) | Glycerolipids, GL | Triglyceride | 1028.957965 | 15.5432 |
| pos_1470 | C66 H124 O6 N1 | TG(27:1/18:1/18:2) | Glycerolipids, GL | Triglyceride | 1026.942315 | 15.2804 |
| pos_1471 | C66 H124 O6 N1 | TG(27:0/18:2/18:2) | Glycerolipids, GL | Triglyceride | 1026.942315 | 15.4616 |
| pos_1472 | C67 H132 O6 N1 | TG(28:0/18:0/18:1) | Glycerolipids, GL | Triglyceride | 1047.004915 | 16.2997 |
| pos_1473 | C67 H130 O6 N1 | TG(28:0/18:1/18:1) | Glycerolipids, GL | Triglyceride | 1044.989265 | 15.9763 |
| pos_1474 | C67 H128 O6 N1 | TG(18:1/22:1/24:1) | Glycerolipids, GL | Triglyceride | 1042.973615 | 15.589 |
| pos_1475 | C67 H128 O6 N1 | TG(28:0/18:1/18:2) | Glycerolipids, GL | Triglyceride | 1042.973615 | 15.8022 |
| pos_1476 | C67 H126 O6 N1 | TG(24:1/18:2/22:1) | Glycerolipids, GL | Triglyceride | 1040.957965 | 15.3813 |
| pos_1477 | C67 H126 O6 N1 | TG(28:0/18:2/18:2) | Glycerolipids, GL | Triglyceride | 1040.957965 | 15.6081 |
| pos_1478 | C67 H123 O6 | TG(28:0/18:1/18:3) | Glycerolipids, GL | Triglyceride | 1023.931416 | 16.042 |
| pos_1479 | C67 H124 O6 N1 | TG(24:1/18:3/22:1) | Glycerolipids, GL | Triglyceride | 1038.942315 | 15.1941 |
| pos_1480 | C73 H132 O6 N2 | TG(28:1/18:3/18:3) | Glycerolipids, GL | Triglyceride | 1133.007989 | 12.661 |
| pos_1482 | C67 H117 O5 | TG(20:4e/22:1/22:3) | Glycerolipids, GL | Triglyceride | 1001.889551 | 13.767 |
| pos_1484 | C68 H132 O6 N1 | TG(29:0/18:1/18:1) | Glycerolipids, GL | Triglyceride | 1059.004915 | 16.149 |
| pos_1485 | C74 H136 O6 N2 | TG(29:1/18:2/18:3) | Glycerolipids, GL | Triglyceride | 1149.039289 | 14.3258 |
| pos_1486 | C74 H134 O6 N2 | TG(29:1/18:3/18:3) | Glycerolipids, GL | Triglyceride | 1147.023639 | 14.0844 |
| pos_1487 | C74 H132 O6 N2 | TG(29:1/18:3/18:4) | Glycerolipids, GL | Triglyceride | 1145.007989 | 13.519 |
| pos_1489 | C69 H136 O6 N1 | TG(18:1/24:0/24:0) | Glycerolipids, GL | Triglyceride | 1075.036215 | 16.6425 |
| pos_1490 | C69 H134 O6 N1 | TG(30:0/18:1/18:1) | Glycerolipids, GL | Triglyceride | 1073.020565 | 16.1685 |
| pos_1492 | C69 H130 O6 N1 | TG(30:1/18:1/18:2) | Glycerolipids, GL | Triglyceride | 1068.989265 | 15.6434 |
| pos_1493 | C69 H130 O6 N1 | TG(30:0/18:2/18:2) | Glycerolipids, GL | Triglyceride | 1068.989265 | 15.8768 |
| pos_1494 | C75 H136 O6 N2 | TG(30:1/18:2/18:4) | Glycerolipids, GL | Triglyceride | 1161.039289 | 13.2499 |
| pos_1495 | C75 H134 O6 N2 | TG(30:1/18:3/18:4) | Glycerolipids, GL | Triglyceride | 1159.023639 | 12.816 |
| pos_1497 | C76 H132 O6 N2 | TG(27:1/18:3/22:6) | Glycerolipids, GL | Triglyceride | 1169.007989 | 13.453 |
| pos_1499 | C76 H130 O6 N2 | TG(27:1/18:4/22:6) | Glycerolipids, GL | Triglyceride | 1166.992339 | 12.9559 |
| pos_1501 | C76 H138 O6 N2 | TG(27:0/18:3/22:4) | Glycerolipids, GL | Triglyceride | 1175.054939 | 14.321 |
| pos_1502 | C76 H138 O6 N2 | TG(29:0/18:3/20:4) | Glycerolipids, GL | Triglyceride | 1175.054939 | 14.7446 |
| pos_1504 | C76 H136 O6 N2 | TG(27:0/18:3/22:5) | Glycerolipids, GL | Triglyceride | 1173.039289 | 14.0675 |
| pos_1505 | C76 H136 O6 N2 | TG(27:1/18:1/22:6) | Glycerolipids, GL | Triglyceride | 1173.039289 | 14.555 |
| pos_1506 | C76 H134 O6 N2 | TG(29:1/18:3/20:5) | Glycerolipids, GL | Triglyceride | 1171.023639 | 13.4492 |
| pos_1507 | C76 H134 O6 N2 | TG(27:1/18:2/22:6) | Glycerolipids, GL | Triglyceride | 1171.023639 | 13.7754 |
| pos_1509 | C71 H140 O6 N1 | TG(26:0/18:1/24:0) | Glycerolipids, GL | Triglyceride | 1103.067515 | 17.1001 |
| pos_1510 | C71 H138 O6 N1 | TG(26:0/18:1/24:1) | Glycerolipids, GL | Triglyceride | 1101.051865 | 16.4016 |
| pos_1511 | C71 H138 O6 N1 | TG(26:0/18:2/24:0) | Glycerolipids, GL | Triglyceride | 1101.051865 | 16.8023 |
| pos_1513 | C71 H136 O6 N1 | TG(26:1/18:1/24:1) | Glycerolipids, GL | Triglyceride | 1099.036215 | 16.3292 |
| pos_1514 | C71 H134 O6 N1 | TG(26:1/18:1/24:2) | Glycerolipids, GL | Triglyceride | 1097.020565 | 15.9221 |
| pos_1515 | C78 H136 O6 N2 | TG(29:1/18:3/22:6) | Glycerolipids, GL | Triglyceride | 1197.039289 | 13.468 |
| pos_1516 | C78 H136 O6 N2 | TG(29:0/18:4/22:6) | Glycerolipids, GL | Triglyceride | 1197.039289 | 14.3115 |
| pos_1517 | C78 H134 O6 N2 | TG(29:1/18:4/22:6) | Glycerolipids, GL | Triglyceride | 1195.023639 | 12.9838 |
| pos_1518 | C78 H132 O6 N2 | TG(25:0/22:6/22:6) | Glycerolipids, GL | Triglyceride | 1193.007989 | 12.971 |
| pos_1519 | C72 H134 O6 Na1 | TG(28:1/18:1/23:1) | Glycerolipids, GL | Triglyceride | 1118.007261 | 13.889 |
| pos_1520 | C72 H126 O6 Na1 | TG(29:1/18:3/22:3) | Glycerolipids, GL | Triglyceride | 1109.944661 | 12.7781 |
| pos_1521 | C78 H142 O6 N2 | TG(29:0/18:1/22:6) | Glycerolipids, GL | Triglyceride | 1203.086239 | 14.6339 |
| pos_1522 | C78 H140 O6 N2 | TG(29:1/18:1/22:6) | Glycerolipids, GL | Triglyceride | 1201.070589 | 14.3143 |
| pos_1523 | C78 H138 O6 N2 | TG(29:1/18:2/22:6) | Glycerolipids, GL | Triglyceride | 1199.054939 | 14.0584 |
| pos_1525 | C79 H138 O6 N2 | TG(30:1/18:3/22:6) | Glycerolipids, GL | Triglyceride | 1211.054939 | 13.746 |
| pos_1526 | C79 H136 O6 N2 | TG(30:1/18:4/22:6) | Glycerolipids, GL | Triglyceride | 1209.039289 | 13.427 |
| pos_1527 | C73 H140 O6 N1 | TG(30:1/16:0/24:2) | Glycerolipids, GL | Triglyceride | 1127.067515 | 15.0446 |
| pos_1528 | C73 H140 O6 N1 | TG(28:0/18:1/24:2) | Glycerolipids, GL | Triglyceride | 1127.067515 | 16.344 |
| pos_1529 | C73 H138 O6 N1 | TG(28:0/18:2/24:2) | Glycerolipids, GL | Triglyceride | 1125.051865 | 16.164 |
| pos_1531 | C73 H127 O6 | TG(30:0/18:3/22:5) | Glycerolipids, GL | Triglyceride | 1099.962716 | 14.4043 |
| pos_1532 | C80 H138 O6 N2 | TG(29:1/20:4/22:6) | Glycerolipids, GL | Triglyceride | 1223.054939 | 14.1443 |
| pos_1533 | C80 H144 O6 N2 | TG(29:1/20:1/22:6) | Glycerolipids, GL | Triglyceride | 1229.101889 | 14.602 |
| pos_1534 | C80 H142 O6 N2 | TG(29:1/20:2/22:6) | Glycerolipids, GL | Triglyceride | 1227.086239 | 14.3645 |
| pos_1535 | C75 H137 O6 | TG(30:1/18:2/24:2) | Glycerolipids, GL | Triglyceride | 1134.040966 | 15.497 |
| pos_1538 | H44 C21 O2 N1 | WE(3:0/18:1) | Wax esters, WE | Wax esters | 342.3366555 | 1.5885 |
| pos_1539 | H42 C22 O2 N1 | WE(6:0/16:3) | Wax esters, WE | Wax esters | 352.3210055 | 1.958 |
| pos_1540 | H48 C23 O2 N1 | WE(3:0/20:1) | Wax esters, WE | Wax esters | 370.3679555 | 2.3369 |
| pos_1541 | H46 C23 O2 N1 | WE(3:0/20:2) | Wax esters, WE | Wax esters | 368.3523055 | 1.6837 |
| pos_1542 | H44 C23 O2 N1 | WE(3:0/20:3) | Wax esters, WE | Wax esters | 366.3366555 | 1.2801 |
| pos_1543 | H42 C23 O2 N1 | WE(3:0/20:4) | Wax esters, WE | Wax esters | 364.3210055 | 1.0231 |
| pos_1544 | H50 C25 O2 N1 | WE(3:0/22:2) | Wax esters, WE | Wax esters | 396.3836055 | 2.3839 |
| pos_1545 | H59 C31 O2 | WE(13:0/18:2) | Wax esters, WE | Wax esters | 463.4509565 | 7.657 |
| pos_1546 | C43 H71 O2 | ZyE(16:2) | Sterol Lipids, ST | Zymosteryl | 619.5448565 | 7.626 |
| pos_1547 | C48 H84 O2 N1 | ZyE(21:2) | Sterol Lipids, ST | Zymosteryl | 706.6496555 | 14.2871 |
| pos_1549 | C49 H86 O2 N1 | ZyE(22:2) | Sterol Lipids, ST | Zymosteryl | 720.6653055 | 14.416 |
| pos_1550 | C60 H102 O2 N1 | ZyE(33:5) | Sterol Lipids, ST | Zymosteryl | 868.7905055 | 11.76 |
| pos_1551 | C60 H100 O2 N1 | ZyE(33:6) | Sterol Lipids, ST | Zymosteryl | 866.7748555 | 11.514 |
| pos_1552 | C62 H101 O2 | ZyE(35:6) | Sterol Lipids, ST | Zymosteryl | 877.7796065 | 12.538 |
| pos_1553 | C63 H110 O2 N1 | ZyE(36:4) | Sterol Lipids, ST | Zymosteryl | 912.8531055 | 15.019 |
| pos_1554 | C64 H108 O2 N1 | ZyE(37:6) | Sterol Lipids, ST | Zymosteryl | 922.8374555 | 13.0398 |
| pos_204 | C37 H74 O5 N1 | DG(16:0/18:1) | Glycerolipids, GL | Diglyceride | 612.5561505 | 2.5772 |
| pos_374 | C26 H55 O7 N1 P1 | LPC(18:0) | Glycerophospholipids, GP | Lyso-phosphatidylcholine | 524.3710685 | 2.4386 |
| neg_1 | C41 H73 O10 N3 S1 P1 | BiotinylPE(10:1/16:0) | Glycerophospholipids, GP | Biotinyl phosphatidylethanolamine | 830.4759805 | 5.6764 |
| neg_2 | C43 H73 O10 N3 S1 P1 | BiotinylPE(10:1/18:2) | Glycerophospholipids, GP | Biotinyl phosphatidylethanolamine | 854.4759805 | 5.1869 |
| neg_4 | C65 H125 O17 P2 | CL(8:0/16:0/16:0/16:0) | Glycerophospholipids, GP | Cardiolipins | 1239.839755 | 9.7192 |
| neg_5 | C67 H129 O17 P2 | CL(6:0/16:0/18:0/18:0) | Glycerophospholipids, GP | Cardiolipins | 1267.871055 | 10.217 |
| neg_6 | C74 H123 O17 P2 | CL(11:4/18:2/18:2/18:2) | Glycerophospholipids, GP | Cardiolipins | 1345.824105 | 8.9929 |
| neg_7 | C75 H142 O17 P2 | CL(22:1/14:0/16:0/14:0) | Glycerophospholipids, GP | Cardiolipins | 688.486664 | 5.2289 |
| neg_8 | C75 H138 O17 P2 | CL(18:2/14:0/14:0/20:1) | Glycerophospholipids, GP | Cardiolipins | 686.471014 | 4.6896 |
| neg_9 | C77 H142 O17 P2 | CL(22:2/15:0/16:0/15:1) | Glycerophospholipids, GP | Cardiolipins | 700.486664 | 3.2642 |
| neg_11 | C77 H132 O17 P2 | CL(14:4/18:1/18:1/18:2) | Glycerophospholipids, GP | Cardiolipins | 695.447539 | 7.6709 |
| neg_12 | C79 H150 O17 P2 | CL(18:1/16:0/16:0/20:0) | Glycerophospholipids, GP | Cardiolipins | 716.517964 | 6.0265 |
| neg_13 | C79 H148 O17 P2 | CL(18:2/16:0/16:0/20:0) | Glycerophospholipids, GP | Cardiolipins | 715.510139 | 5.4415 |
| neg_14 | C79 H147 O17 P2 | CL(22:3/16:0/16:0/16:0) | Glycerophospholipids, GP | Cardiolipins | 1430.011905 | 5.4436 |
| neg_16 | C79 H142 O17 P2 | CL(18:2/16:1/18:1/18:1) | Glycerophospholipids, GP | Cardiolipins | 712.486664 | 4.7992 |
| neg_19 | C79 H142 O17 P2 | CL(18:3/16:0/18:0/18:2) | Glycerophospholipids, GP | Cardiolipins | 712.486664 | 6.1308 |
| neg_20 | C81 H136 O17 P2 | CL(18:2/14:1/18:1/22:6) | Glycerophospholipids, GP | Cardiolipins | 721.463189 | 2.675 |
| neg_21 | C81 H149 O17 P2 | CL(18:2/18:1/20:1/16:0) | Glycerophospholipids, GP | Cardiolipins | 1456.027555 | 5.472 |
| neg_22 | C81 H143 O17 P2 | CL(18:2/18:1/18:2/18:2) | Glycerophospholipids, GP | Cardiolipins | 1449.980605 | 12.295 |
| neg_24 | C82 H157 O17 P2 | CL(23:0/16:0/16:0/18:1) | Glycerophospholipids, GP | Cardiolipins | 1476.090155 | 7.422 |
| neg_25 | C82 H155 O17 P2 | CL(23:0/16:0/16:0/18:2) | Glycerophospholipids, GP | Cardiolipins | 1474.074505 | 6.6528 |
| neg_26 | C82 H153 O17 P2 | CL(23:1/16:0/16:0/18:2) | Glycerophospholipids, GP | Cardiolipins | 1472.058855 | 6.6998 |
| neg_27 | C83 H158 O17 P2 | CL(24:0/16:0/16:0/18:1) | Glycerophospholipids, GP | Cardiolipins | 744.549264 | 7.4313 |
| neg_28 | C83 H140 O17 P2 | CL(18:2/16:0/18:2/22:6) | Glycerophospholipids, GP | Cardiolipins | 735.478839 | 2.7478 |
| neg_29 | C83 H156 O17 P2 | CL(24:0/16:0/18:2/16:0) | Glycerophospholipids, GP | Cardiolipins | 743.541439 | 6.129 |
| neg_30 | C83 H154 O17 P2 | CL(18:2/16:0/24:1/16:0) | Glycerophospholipids, GP | Cardiolipins | 742.533614 | 6.1224 |
| neg_31 | C83 H154 O17 P2 | CL(18:2/16:0/16:0/24:1) | Glycerophospholipids, GP | Cardiolipins | 742.533614 | 6.6872 |
| neg_32 | C83 H154 O17 P2 | CL(18:1/18:1/18:1/20:0) | Glycerophospholipids, GP | Cardiolipins | 742.533614 | 7.4964 |
| neg_33 | C83 H154 O17 P2 | CL(18:2/18:0/18:1/20:0) | Glycerophospholipids, GP | Cardiolipins | 742.533614 | 7.707 |
| neg_34 | C83 H150 O17 P2 | CL(18:2/18:1/18:1/20:1) | Glycerophospholipids, GP | Cardiolipins | 740.517964 | 5.516 |
| neg_35 | C83 H147 O17 P2 | CL(18:2/18:2/20:1/18:2) | Glycerophospholipids, GP | Cardiolipins | 1478.011905 | 4.975 |
| neg_36 | C83 H146 O17 P2 | CL(18:2/18:2/18:2/20:1) | Glycerophospholipids, GP | Cardiolipins | 738.502314 | 6.083 |
| neg_37 | C83 H146 O17 P2 | CL(18:3/18:1/18:2/20:1) | Glycerophospholipids, GP | Cardiolipins | 738.502314 | 6.266 |
| neg_38 | C83 H142 O17 P2 | CL(18:3/18:0/18:2/20:4) | Glycerophospholipids, GP | Cardiolipins | 736.486664 | 4.505 |
| neg_39 | C84 H161 O17 P2 | CL(23:0/16:0/18:0/18:1) | Glycerophospholipids, GP | Cardiolipins | 1504.121455 | 7.2167 |
| neg_40 | C84 H161 O17 P2 | CL(21:0/18:0/18:0/18:1) | Glycerophospholipids, GP | Cardiolipins | 1504.121455 | 7.4327 |
| neg_41 | C84 H159 O17 P2 | CL(23:0/16:0/18:1/18:1) | Glycerophospholipids, GP | Cardiolipins | 1502.105805 | 7.504 |
| neg_44 | C84 H155 O17 P2 | CL(23:1/16:0/18:1/18:2) | Glycerophospholipids, GP | Cardiolipins | 1498.074505 | 6.7436 |
| neg_45 | C84 H155 O17 P2 | CL(21:0/18:1/18:1/18:2) | Glycerophospholipids, GP | Cardiolipins | 1498.074505 | 11.8348 |
| neg_46 | C84 H153 O17 P2 | CL(23:0/16:1/18:2/18:2) | Glycerophospholipids, GP | Cardiolipins | 1496.058855 | 11.4131 |
| neg_47 | C84 H151 O17 P2 | CL(21:1/18:1/18:2/18:2) | Glycerophospholipids, GP | Cardiolipins | 1494.043205 | 12.8732 |
| neg_48 | C85 H156 O17 P2 | CL(18:2/16:0/18:1/24:1) | Glycerophospholipids, GP | Cardiolipins | 755.541439 | 8.636 |
| neg_49 | C86 H157 O17 P2 | CL(23:0/18:1/18:2/18:2) | Glycerophospholipids, GP | Cardiolipins | 1524.090155 | 6.775 |
| neg_51 | C86 H153 O17 P2 | CL(23:1/18:2/18:2/18:2) | Glycerophospholipids, GP | Cardiolipins | 1520.058855 | 6.0706 |
| neg_52 | C86 H153 O17 P2 | CL(19:1/18:1/18:2/22:3) | Glycerophospholipids, GP | Cardiolipins | 1520.058855 | 12.9039 |
| neg_53 | C86 H151 O17 P2 | CL(19:0/18:1/18:2/22:5) | Glycerophospholipids, GP | Cardiolipins | 1518.043205 | 12.4965 |
| neg_54 | C86 H149 O17 P2 | CL(19:0/18:1/18:2/22:6) | Glycerophospholipids, GP | Cardiolipins | 1516.027555 | 12.0797 |
| neg_56 | C87 H148 O17 P2 | CL(18:2/16:0/22:2/22:6) | Glycerophospholipids, GP | Cardiolipins | 763.510139 | 4.8231 |
| neg_57 | C87 H144 O17 P2 | CL(22:6/16:0/18:1/22:5) | Glycerophospholipids, GP | Cardiolipins | 761.494489 | 2.766 |
| neg_59 | C87 H144 O17 P2 | CL(18:3/22:3/22:6/16:0) | Glycerophospholipids, GP | Cardiolipins | 761.494489 | 4.022 |
| neg_60 | C87 H140 O17 P2 | CL(18:2/18:2/20:4/22:6) | Glycerophospholipids, GP | Cardiolipins | 759.478839 | 2.258 |
| neg_61 | C87 H162 O17 P2 | CL(24:0/18:1/18:1/18:1) | Glycerophospholipids, GP | Cardiolipins | 770.564914 | 7.5224 |
| neg_62 | C87 H158 O17 P2 | CL(18:2/18:1/18:1/24:1) | Glycerophospholipids, GP | Cardiolipins | 768.549264 | 6.2793 |
| neg_63 | C87 H154 O17 P2 | CL(18:2/18:2/18:2/24:1) | Glycerophospholipids, GP | Cardiolipins | 766.533614 | 6.0431 |
| neg_64 | C87 H150 O17 P2 | CL(18:3/18:2/20:0/22:4) | Glycerophospholipids, GP | Cardiolipins | 764.517964 | 5.506 |
| neg_65 | C88 H160 O17 P2 | CL(23:0/16:0/18:1/22:4) | Glycerophospholipids, GP | Cardiolipins | 775.557089 | 7.352 |
| neg_66 | C88 H160 O17 P2 | CL(23:0/20:4/18:1/18:0) | Glycerophospholipids, GP | Cardiolipins | 775.557089 | 7.6715 |
| neg_67 | C89 H152 O17 P2 | CL(18:2/18:1/22:1/22:6) | Glycerophospholipids, GP | Cardiolipins | 777.525789 | 7.2389 |
| neg_68 | C89 H168 O17 P2 | CL(24:0/16:0/18:2/22:0) | Glycerophospholipids, GP | Cardiolipins | 785.588389 | 7.792 |
| neg_69 | C89 H160 O17 P2 | CL(24:2/18:1/18:2/20:1) | Glycerophospholipids, GP | Cardiolipins | 781.557089 | 8.6499 |
| neg_72 | C89 H157 O17 P2 | CL(18:3/18:1/20:3/24:1) | Glycerophospholipids, GP | Cardiolipins | 1560.090155 | 11.8193 |
| neg_73 | C89 H155 O17 P2 | CL(18:3/18:2/20:3/24:1) | Glycerophospholipids, GP | Cardiolipins | 1558.074505 | 11.3599 |
| neg_75 | C91 H174 O17 P2 | CL(22:0/18:1/20:0/22:0) | Glycerophospholipids, GP | Cardiolipins | 800.611864 | 10.356 |
| neg_76 | C91 H156 O17 P2 | CL(18:2/18:1/22:6/24:1) | Glycerophospholipids, GP | Cardiolipins | 791.541439 | 7.7579 |
| neg_79 | C91 H144 O17 P2 | CL(18:2/20:3/22:5/22:6) | Glycerophospholipids, GP | Cardiolipins | 785.494489 | 3.4608 |
| neg_80 | C91 H144 O17 P2 | CL(22:6/20:3/22:5/18:2) | Glycerophospholipids, GP | Cardiolipins | 785.494489 | 3.8702 |
| neg_81 | C91 H170 O17 P2 | CL(18:2/20:1/22:0/22:0) | Glycerophospholipids, GP | Cardiolipins | 798.596214 | 9.7284 |
| neg_82 | C91 H164 O17 P2 | CL(18:2/18:1/22:2/24:1) | Glycerophospholipids, GP | Cardiolipins | 795.572739 | 8.9621 |
| neg_83 | C92 H164 O17 P2 | CL(23:0/18:1/20:1/22:5) | Glycerophospholipids, GP | Cardiolipins | 801.572739 | 7.4268 |
| neg_84 | C92 H164 O17 P2 | CL(23:1/16:0/22:3/22:3) | Glycerophospholipids, GP | Cardiolipins | 801.572739 | 7.7422 |
| neg_87 | C35 H70 O5 N1 | Cer(d18:0/16:0) | Sphingolipids, SP | Ceramide | 584.5259475 | 7.3982 |
| neg_88 | C37 H70 O5 N1 | Cer(d18:0/18:2) | Sphingolipids, SP | Ceramide | 608.5259475 | 6.7918 |
| neg_89 | C37 H70 O5 N1 | Cer(d16:2/20:0) | Sphingolipids, SP | Ceramide | 608.5259475 | 8.1489 |
| neg_91 | C39 H78 O5 N1 | Cer(d18:0/20:0) | Sphingolipids, SP | Ceramide | 640.5885475 | 9.4415 |
| neg_92 | C41 H80 O5 N1 | Cer(d17:1/22:0) | Sphingolipids, SP | Ceramide | 666.6041975 | 9.291 |
| neg_93 | C42 H82 O5 N1 | Cer(d18:1/22:0) | Sphingolipids, SP | Ceramide | 680.6198475 | 9.0824 |
| neg_94 | C42 H80 O5 N1 | Cer(d17:1/23:1) | Sphingolipids, SP | Ceramide | 678.6041975 | 8.6416 |
| neg_95 | C43 H82 O5 N1 | Cer(d17:1/24:1) | Sphingolipids, SP | Ceramide | 692.6198475 | 9.1589 |
| neg_96 | C43 H86 O5 N1 | Cer(d18:0/24:0) | Sphingolipids, SP | Ceramide | 696.6511475 | 11.3392 |
| neg_97 | C43 H84 O5 N1 | Cer(d16:1/26:0) | Sphingolipids, SP | Ceramide | 694.6354975 | 11.412 |
| neg_98 | C44 H84 O5 N1 | Cer(d17:1/25:1) | Sphingolipids, SP | Ceramide | 706.6354975 | 9.617 |
| neg_99 | C43 H82 O5 N1 | Cer(d16:2/26:0) | Sphingolipids, SP | Ceramide | 692.6198475 | 10.9548 |
| neg_100 | C43 H82 O7 N1 | Cer(d24:0/18:2+2O) | Sphingolipids, SP | Ceramide | 724.6096775 | 8.6487 |
| neg_101 | C45 H88 O5 N1 | Cer(d18:1/26:0) | Sphingolipids, SP | Ceramide | 722.6667975 | 12.252 |
| neg_102 | C39 H78 O5 N1 | Cer(m18:0/20:0+O) | Sphingolipids, SP | Ceramide | 640.5885475 | 9.461 |
| neg_103 | C42 H80 O4 N1 | Cer(m17:1/24:1) | Sphingolipids, SP | Ceramide | 662.6092825 | 9.023 |
| neg_104 | C35 H68 O6 N1 | Cer(t17:1/17:0) | Sphingolipids, SP | Ceramide | 598.5052125 | 6.0847 |
| neg_105 | C37 H68 O6 N1 | Cer(t18:1/18:2) | Sphingolipids, SP | Ceramide | 622.5052125 | 5.55 |
| neg_106 | C39 H76 O6 N1 | Cer(t17:1/20:0) | Sphingolipids, SP | Ceramide | 654.5678125 | 8.1424 |
| neg_107 | C38 H76 O5 N1 | Cer(t18:0/20:0+O) | Sphingolipids, SP | Ceramide | 626.5728975 | 8.1865 |
| neg_108 | C38 H74 O4 N1 | Cer(t18:1/20:0) | Sphingolipids, SP | Ceramide | 608.5623325 | 8.1178 |
| neg_109 | C40 H78 O6 N1 | Cer(t17:1/21:0) | Sphingolipids, SP | Ceramide | 668.5834625 | 8.641 |
| neg_110 | C40 H80 O6 N1 | Cer(t18:0/21:0) | Sphingolipids, SP | Ceramide | 670.5991125 | 9.0793 |
| neg_111 | C41 H82 O6 N1 | Cer(t17:0/22:0) | Sphingolipids, SP | Ceramide | 684.6147625 | 9.62 |
| neg_112 | C40 H78 O6 N1 | Cer(t18:1/21:0) | Sphingolipids, SP | Ceramide | 668.5834625 | 8.6466 |
| neg_113 | C41 H80 O6 N1 | Cer(t17:1/22:0) | Sphingolipids, SP | Ceramide | 682.5991125 | 9.134 |
| neg_114 | C39 H76 O5 N1 | Cer(t18:1/21:0+O) | Sphingolipids, SP | Ceramide | 638.5728975 | 8.286 |
| neg_115 | C42 H84 O6 N1 | Cer(t17:0/23:0) | Sphingolipids, SP | Ceramide | 698.6304125 | 10.082 |
| neg_116 | C41 H82 O7 N1 | Cer(t17:0/23:0+O) | Sphingolipids, SP | Ceramide | 700.6096775 | 9.2253 |
| neg_117 | C42 H82 O6 N1 | Cer(t17:1/23:0) | Sphingolipids, SP | Ceramide | 696.6147625 | 9.642 |
| neg_118 | C41 H80 O7 N1 | Cer(t17:1/23:0+O) | Sphingolipids, SP | Ceramide | 698.5940275 | 8.7926 |
| neg_119 | C42 H84 O7 N1 | Cer(t17:0/24:0+O) | Sphingolipids, SP | Ceramide | 714.6253275 | 9.7015 |
| neg_120 | C42 H82 O6 N1 | Cer(t18:0/23:1) | Sphingolipids, SP | Ceramide | 696.6147625 | 8.9594 |
| neg_121 | C43 H84 O6 N1 | Cer(t17:0/24:1) | Sphingolipids, SP | Ceramide | 710.6304125 | 9.428 |
| neg_122 | C43 H84 O6 N1 | Cer(t17:1/24:0) | Sphingolipids, SP | Ceramide | 710.6304125 | 10.1699 |
| neg_123 | C42 H82 O7 N1 | Cer(t17:1/24:0+O) | Sphingolipids, SP | Ceramide | 712.6096775 | 9.2919 |
| neg_124 | C43 H82 O6 N1 | Cer(t17:1/24:1) | Sphingolipids, SP | Ceramide | 708.6147625 | 9.0222 |
| neg_125 | C44 H88 O6 N1 | Cer(t17:0/25:0) | Sphingolipids, SP | Ceramide | 726.6617125 | 11.054 |
| neg_126 | C43 H86 O7 N1 | Cer(t17:0/25:0+O) | Sphingolipids, SP | Ceramide | 728.6409775 | 10.1969 |
| neg_127 | C43 H84 O6 N1 | Cer(t18:0/24:1) | Sphingolipids, SP | Ceramide | 710.6304125 | 9.4434 |
| neg_128 | C44 H86 O6 N1 | Cer(t17:0/25:1) | Sphingolipids, SP | Ceramide | 724.6460625 | 9.8967 |
| neg_129 | C44 H86 O6 N1 | Cer(t17:1/25:0) | Sphingolipids, SP | Ceramide | 724.6460625 | 10.6269 |
| neg_130 | C43 H84 O7 N1 | Cer(t17:1/25:0+O) | Sphingolipids, SP | Ceramide | 726.6253275 | 9.7971 |
| neg_131 | C42 H80 O4 N1 | Cer(t18:1/24:1) | Sphingolipids, SP | Ceramide | 662.6092825 | 9.0253 |
| neg_132 | C45 H90 O6 N1 | Cer(t17:0/26:0) | Sphingolipids, SP | Ceramide | 740.6773625 | 11.4395 |
| neg_133 | C43 H86 O5 N1 | Cer(t18:0/25:0+O) | Sphingolipids, SP | Ceramide | 696.6511475 | 10.6659 |
| neg_134 | C44 H86 O6 N1 | Cer(t18:0/25:1) | Sphingolipids, SP | Ceramide | 724.6460625 | 9.9015 |
| neg_135 | C45 H88 O6 N1 | Cer(t17:0/26:1) | Sphingolipids, SP | Ceramide | 738.6617125 | 10.388 |
| neg_136 | C45 H88 O6 N1 | Cer(t17:1/26:0) | Sphingolipids, SP | Ceramide | 738.6617125 | 11.0919 |
| neg_137 | C43 H84 O5 N1 | Cer(t18:1/25:0+O) | Sphingolipids, SP | Ceramide | 694.6354975 | 9.5537 |
| neg_138 | C44 H86 O7 N1 | Cer(t17:1/26:0+O) | Sphingolipids, SP | Ceramide | 740.6409775 | 10.2743 |
| neg_139 | C44 H84 O6 N1 | Cer(t18:1/25:1) | Sphingolipids, SP | Ceramide | 722.6304125 | 9.5137 |
| neg_140 | C44 H88 O5 N1 | Cer(t18:0/26:0+O) | Sphingolipids, SP | Ceramide | 710.6667975 | 11.1201 |
| neg_141 | C45 H88 O6 N1 | Cer(t18:0/26:1) | Sphingolipids, SP | Ceramide | 738.6617125 | 10.3511 |
| neg_142 | C52 H95 O18 N2 | CerG2GNAc1(d16:1/16:0) | Saccharolipids, SL | Simple Glc series | 1035.658542 | 1.787 |
| neg_143 | C55 H102 O18 N2 | CerG2GNAc1(d19:0/16:0) | Saccharolipids, SL | Simple Glc series | 539.3569325 | 1.513 |
| neg_144 | C56 H103 O18 N2 | CerG2GNAc1(d18:1/18:0) | Saccharolipids, SL | Simple Glc series | 1091.721142 | 2.6529 |
| neg_145 | C56 H99 O18 N2 | CerG2GNAc1(d18:2/18:1) | Saccharolipids, SL | Simple Glc series | 1087.689842 | 1.8971 |
| neg_146 | C57 H101 O17 N2 | CerG2GNAc1(m19:1/18:2) | Saccharolipids, SL | Simple Glc series | 1085.710577 | 9.187 |
| neg_147 | C34 H59 O17 | DGDG(4:0/13:1) | Saccharolipids, SL | Digalactosyl diglyceride | 739.3757785 | 0.672 |
| neg_148 | C49 H89 O17 | DGDG(16:0/16:1) | Saccharolipids, SL | Digalactosyl diglyceride | 949.6105285 | 1.326 |
| neg_149 | C49 H87 O17 | DGDG(16:0/16:2) | Saccharolipids, SL | Digalactosyl diglyceride | 947.5948785 | 3.7726 |
| neg_151 | C51 H89 O17 | DGDG(18:1/16:2) | Saccharolipids, SL | Digalactosyl diglyceride | 973.6105285 | 3.7878 |
| neg_153 | C51 H87 O17 | DGDG(20:2/14:2) | Saccharolipids, SL | Digalactosyl diglyceride | 971.5948785 | 1.8514 |
| neg_154 | C51 H87 O17 | DGDG(18:2/16:2) | Saccharolipids, SL | Digalactosyl diglyceride | 971.5948785 | 3.2772 |
| neg_155 | C49 H79 O15 | DGDG(18:2/16:4) | Saccharolipids, SL | Digalactosyl diglyceride | 907.5424485 | 1.441 |
| neg_156 | C51 H93 O17 | DGDG(18:1/17:0) | Saccharolipids, SL | Digalactosyl diglyceride | 977.6418285 | 7.6189 |
| neg_157 | C53 H91 O17 | DGDG(18:2/19:2) | Saccharolipids, SL | Digalactosyl diglyceride | 999.6261785 | 5.8404 |
| neg_159 | C53 H77 O15 | DGDG(18:2/20:9) | Saccharolipids, SL | Digalactosyl diglyceride | 953.5267985 | 4.1684 |
| neg_160 | C54 H97 O17 | DGDG(20:0/18:2) | Saccharolipids, SL | Digalactosyl diglyceride | 1017.673129 | 8.3024 |
| neg_161 | C57 H87 O15 | DGDG(16:0/26:10) | Saccharolipids, SL | Digalactosyl diglyceride | 1011.605049 | 2.733 |
| neg_162 | C59 H89 O15 | DGDG(18:1/26:10) | Saccharolipids, SL | Digalactosyl diglyceride | 1037.620699 | 2.7713 |
| neg_163 | C59 H87 O15 | DGDG(18:2/26:10) | Saccharolipids, SL | Digalactosyl diglyceride | 1035.605049 | 2.1734 |
| neg_164 | C60 H109 O17 | DGDG(18:2/26:0) | Saccharolipids, SL | Digalactosyl diglyceride | 1101.767029 | 11.1681 |
| neg_165 | C60 H101 O15 | DGDG(18:2/27:4) | Saccharolipids, SL | Digalactosyl diglyceride | 1061.714599 | 9.7619 |
| neg_166 | C63 H97 O17 | DGDG(18:1/28:10) | Saccharolipids, SL | Digalactosyl diglyceride | 1125.673129 | 6.7101 |
| neg_167 | C63 H95 O17 | DGDG(18:2/28:10) | Saccharolipids, SL | Digalactosyl diglyceride | 1123.657479 | 6.0365 |
| neg_168 | C63 H97 O17 | DGDG(18:1/29:10) | Saccharolipids, SL | Digalactosyl diglyceride | 1125.673129 | 6.7114 |
| neg_169 | C63 H95 O17 | DGDG(18:2/29:10) | Saccharolipids, SL | Digalactosyl diglyceride | 1123.657479 | 6.0425 |
| neg_170 | C63 H93 O17 | DGDG(18:3/29:10) | Saccharolipids, SL | Digalactosyl diglyceride | 1121.641829 | 5.5185 |
| neg_171 | C62 H101 O15 | DGDG(18:2/29:6) | Saccharolipids, SL | Digalactosyl diglyceride | 1085.714599 | 9.1855 |
| neg_172 | C65 H99 O17 | DGDG(18:2/30:10) | Saccharolipids, SL | Digalactosyl diglyceride | 1151.688779 | 6.8079 |
| neg_173 | C65 H97 O17 | DGDG(22:5/26:8) | Saccharolipids, SL | Digalactosyl diglyceride | 1149.673129 | 6.1217 |
| neg_174 | C65 H95 O17 | DGDG(20:4/28:10) | Saccharolipids, SL | Digalactosyl diglyceride | 1147.657479 | 5.4808 |
| neg_175 | C65 H93 O17 | DGDG(22:6/26:9) | Saccharolipids, SL | Digalactosyl diglyceride | 1145.641829 | 5.0069 |
| neg_176 | C65 H91 O17 | DGDG(22:6/26:10) | Saccharolipids, SL | Digalactosyl diglyceride | 1143.626179 | 4.501 |
| neg_177 | C64 H97 O15 | DGDG(20:4/29:8) | Saccharolipids, SL | Digalactosyl diglyceride | 1105.683299 | 6.8174 |
| neg_178 | C65 H97 O17 | DGDG(20:4/29:9) | Saccharolipids, SL | Digalactosyl diglyceride | 1149.673129 | 6.1292 |
| neg_179 | C65 H95 O17 | DGDG(20:4/29:10) | Saccharolipids, SL | Digalactosyl diglyceride | 1147.657479 | 5.4809 |
| neg_180 | C65 H93 O17 | DGDG(22:6/27:9) | Saccharolipids, SL | Digalactosyl diglyceride | 1145.641829 | 4.9909 |
| neg_181 | C65 H91 O17 | DGDG(22:6/27:10) | Saccharolipids, SL | Digalactosyl diglyceride | 1143.626179 | 4.4826 |
| neg_182 | C67 H121 O17 | DGDG(32:1/18:2) | Saccharolipids, SL | Digalactosyl diglyceride | 1197.860929 | 6.496 |
| neg_183 | C67 H119 O17 | DGDG(32:1/18:3) | Saccharolipids, SL | Digalactosyl diglyceride | 1195.845279 | 10.2012 |
| neg_184 | C67 H119 O17 | DGDG(33:1/18:3) | Saccharolipids, SL | Digalactosyl diglyceride | 1195.845279 | 10.1979 |
| neg_185 | C69 H121 O17 | DGDG(22:0/30:5) | Saccharolipids, SL | Digalactosyl diglyceride | 1221.860929 | 5.8506 |
| neg_188 | C71 H131 O17 | DGDG(36:0/18:2) | Saccharolipids, SL | Digalactosyl diglyceride | 1255.939179 | 6.495 |
| neg_189 | C71 H131 O17 | DGDG(37:0/18:2) | Saccharolipids, SL | Digalactosyl diglyceride | 1255.939179 | 6.509 |
| neg_191 | C72 H133 O17 | DGDG(38:1/18:1) | Saccharolipids, SL | Digalactosyl diglyceride | 1269.954829 | 6.613 |
| neg_192 | C72 H133 O17 | DGDG(38:0/18:2) | Saccharolipids, SL | Digalactosyl diglyceride | 1269.954829 | 12.9389 |
| neg_193 | C73 H133 O17 | DGDG(38:1/18:2) | Saccharolipids, SL | Digalactosyl diglyceride | 1281.954829 | 6.5215 |
| neg_194 | C73 H127 O17 | DGDG(26:0/30:6) | Saccharolipids, SL | Digalactosyl diglyceride | 1275.907879 | 5.3102 |
| neg_195 | C73 H117 O17 | DGDG(28:1/29:10) | Saccharolipids, SL | Digalactosyl diglyceride | 1265.829629 | 9.651 |
| neg_196 | C32 H59 O16 | DGMG(16:0) | Saccharolipids, SL | Digalactosylmonoacylglycerol | 699.3808635 | 1.5488 |
| neg_198 | C34 H63 O16 | DGMG(18:0) | Saccharolipids, SL | Digalactosylmonoacylglycerol | 727.4121635 | 2.3113 |
| neg_200 | C34 H59 O16 | DGMG(18:2) | Saccharolipids, SL | Digalactosylmonoacylglycerol | 723.3808635 | 1.3461 |
| neg_201 | C34 H57 O16 | DGMG(18:3) | Saccharolipids, SL | Digalactosylmonoacylglycerol | 721.3652135 | 1.0701 |
| neg_202 | O2 H27 C18 | FA(18:4) | Fatty Acyls, FA | Fatty Acyls | 275.2016535 | 0.9509 |
| neg_203 | C59 H104 O20 N2 | GM3(m18:1/18:1) | Sphingolipids, SP | Ganglioside | 580.3596725 | 1.8984 |
| neg_204 | C61 H108 O20 N2 | GM3(m18:1/20:1) | Sphingolipids, SP | Ganglioside | 594.3753225 | 2.7062 |
| neg_205 | C39 H72 O10 N1 | Hex1Cer(d14:1/18:1) | Sphingolipids, SP | Hexosylceramide | 714.5161725 | 7.301 |
| neg_206 | C43 H80 O10 N1 | Hex1Cer(d16:1/20:1) | Sphingolipids, SP | Hexosylceramide | 770.5787725 | 6.9036 |
| neg_207 | C49 H92 O10 N1 | Hex1Cer(d16:2/26:0) | Sphingolipids, SP | Hexosylceramide | 854.6726725 | 9.954 |
| neg_208 | C42 H78 O9 N1 | Hex1Cer(m17:0/18:2) | Sphingolipids, SP | Hexosylceramide | 740.5682075 | 6.5991 |
| neg_210 | C41 H76 O11 N1 | Hex1Cer(t16:1/18:1) | Sphingolipids, SP | Hexosylceramide | 758.5423875 | 5.645 |
| neg_211 | C42 H78 O9 N1 | Hex1Cer(t18:0/18:2) | Sphingolipids, SP | Hexosylceramide | 740.5682075 | 6.5711 |
| neg_212 | C44 H84 O9 N1 | Hex1Cer(t20:0/18:1) | Sphingolipids, SP | Hexosylceramide | 770.6151575 | 8.347 |
| neg_213 | C46 H86 O11 N1 | Hex1Cer(t20:1/18:1) | Sphingolipids, SP | Hexosylceramide | 828.6206375 | 7.6139 |
| neg_214 | C55 H102 O15 N1 | Hex2Cer(d23:0/18:2) | Sphingolipids, SP | Hexosylceramide | 1016.725498 | 6.9661 |
| neg_216 | C49 H90 O18 N1 | Hex3Cer(d13:0/18:1) | Sphingolipids, SP | Hexosylceramide | 980.6163425 | 7.166 |
| neg_218 | C56 H102 O19 N1 | Hex3Cer(m19:0/18:2) | Sphingolipids, SP | Hexosylceramide | 1092.705158 | 9.9396 |
| neg_219 | C56 H102 O19 N1 | Hex3Cer(t20:0/18:2) | Sphingolipids, SP | Hexosylceramide | 1092.705158 | 9.9433 |
| neg_220 | C38 H72 O10 P1 | LBPA(16:0/16:1) | Glycerophospholipids, GP | lysobisphosphatidic acid | 719.4868615 | 7.6032 |
| neg_221 | C40 H78 O10 P1 | LBPA(16:0/18:0) | Glycerophospholipids, GP | lysobisphosphatidic acid | 749.5338115 | 7.543 |
| neg_222 | C42 H80 O10 P1 | LBPA(16:0/20:1) | Glycerophospholipids, GP | lysobisphosphatidic acid | 775.5494615 | 7.657 |
| neg_223 | C19 H39 O7 N1 P1 | LPE(14:0) | Glycerophospholipids, GP | Lyso-phosphatidylethanolamine | 424.2469655 | 1.1929 |
| neg_224 | C25 H49 O7 N1 P1 | LPE(20:1) | Glycerophospholipids, GP | Lyso-phosphatidylethanolamine | 506.3252155 | 2.7051 |
| neg_225 | C24 H48 O9 N0 P1 | LPG(18:0) | Glycerophospholipids, GP | Lyso-phosphatidylglycerol | 511.3041465 | 2.3555 |
| neg_226 | C22 H40 O7 N0 P1 | LPMe(18:2) | Glycerophospholipids, GP | Lyso-phosphatidylmethanol | 447.2517165 | 1.4167 |
| neg_227 | C22 H38 O7 N0 P1 | LPMe(18:3) | Glycerophospholipids, GP | Lyso-phosphatidylmethanol | 445.2360665 | 1.1333 |
| neg_229 | C24 H46 O7 N0 P1 | LPMe(20:1) | Glycerophospholipids, GP | Lyso-phosphatidylmethanol | 477.2986665 | 2.641 |
| neg_230 | C21 H43 O7 N1 P1 | LdMePE(14:0) | Glycerophospholipids, GP | Lysodimethylphosphatidylethanolamine | 452.2782655 | 1.1989 |
| neg_231 | C23 H47 O7 N1 P1 | LdMePE(16:0) | Glycerophospholipids, GP | Lysodimethylphosphatidylethanolamine | 480.3095655 | 1.6461 |
| neg_232 | C25 H51 O7 N1 P1 | LdMePE(18:0) | Glycerophospholipids, GP | Lysodimethylphosphatidylethanolamine | 508.3408655 | 2.4478 |
| neg_233 | C25 H45 O7 N1 P1 | LdMePE(18:3) | Glycerophospholipids, GP | Lysodimethylphosphatidylethanolamine | 502.2939155 | 1.1376 |
| neg_234 | C27 H53 O7 N1 P1 | LdMePE(20:1) | Glycerophospholipids, GP | Lysodimethylphosphatidylethanolamine | 534.3565155 | 2.7125 |
| neg_235 | C38 H71 O10 | MGDG(16:0/13:0) | Saccharolipids, SL | Monogalactosyldiacylglycerol | 687.5052735 | 3.8797 |
| neg_236 | C38 H59 O10 | MGDG(18:3/11:3) | Saccharolipids, SL | Monogalactosyldiacylglycerol | 675.4113735 | 1.143 |
| neg_237 | C40 H73 O10 | MGDG(18:1/13:0) | Saccharolipids, SL | Monogalactosyldiacylglycerol | 713.5209235 | 3.9375 |
| neg_238 | C40 H71 O10 | MGDG(18:2/13:0) | Saccharolipids, SL | Monogalactosyldiacylglycerol | 711.5052735 | 3.3025 |
| neg_239 | C44 H83 O12 | MGDG(16:0/18:0) | Saccharolipids, SL | Monogalactosyldiacylglycerol | 803.5890035 | 8.679 |
| neg_240 | C45 H79 O11 | MGDG(16:1e/18:3) | Saccharolipids, SL | Monogalactosyldiacylglycerol | 795.5627885 | 2.0972 |
| neg_241 | C46 H81 O12 | MGDG(17:1/18:2) | Saccharolipids, SL | Monogalactosyldiacylglycerol | 825.5733535 | 7.286 |
| neg_242 | C45 H81 O9 | MGDG(18:1e/18:2) | Saccharolipids, SL | Monogalactosyldiacylglycerol | 765.5886085 | 6.1169 |
| neg_244 | C48 H87 O12 | MGDG(19:0/18:2) | Saccharolipids, SL | Monogalactosyldiacylglycerol | 855.6203035 | 8.704 |
| neg_245 | C48 H85 O12 | MGDG(20:1/18:2) | Saccharolipids, SL | Monogalactosyldiacylglycerol | 853.6046535 | 7.9805 |
| neg_246 | C48 H81 O10 | MGDG(16:0/23:5) | Saccharolipids, SL | Monogalactosyldiacylglycerol | 817.5835235 | 4.694 |
| neg_247 | C49 H87 O10 | MGDG(18:3/22:0) | Saccharolipids, SL | Monogalactosyldiacylglycerol | 835.6304735 | 9.204 |
| neg_248 | C54 H79 O10 | MGDG(18:2/27:10) | Saccharolipids, SL | Monogalactosyldiacylglycerol | 887.5678735 | 5.528 |
| neg_250 | C57 H101 O12 | MGDG(18:1/28:3) | Saccharolipids, SL | Monogalactosyldiacylglycerol | 977.7298535 | 9.2417 |
| neg_256 | C59 H107 O12 | MGDG(18:2/30:1) | Saccharolipids, SL | Monogalactosyldiacylglycerol | 1007.776804 | 10.326 |
| neg_258 | C26 H49 O11 | MGMG(16:0) | Saccharolipids, SL | Monogalactosylmonoacylglycerol | 537.3280385 | 1.8744 |
| neg_259 | C28 H53 O11 | MGMG(18:0) | Saccharolipids, SL | Monogalactosylmonoacylglycerol | 565.3593385 | 2.9266 |
| neg_260 | C28 H51 O11 | MGMG(18:1) | Saccharolipids, SL | Monogalactosylmonoacylglycerol | 563.3436885 | 1.9963 |
| neg_261 | C28 H49 O11 | MGMG(18:2) | Saccharolipids, SL | Monogalactosylmonoacylglycerol | 561.3280385 | 1.6024 |
| neg_262 | C28 H47 O11 | MGMG(18:3) | Saccharolipids, SL | Monogalactosylmonoacylglycerol | 559.3123885 | 1.2653 |
| neg_263 | C53 H93 O16 P2 | MLCL(10:3/16:0/18:2) | Glycerophospholipids, GP | Monolysocardiolipin | 1047.59444 | 7.293 |
| neg_264 | C54 H94 O16 P2 | MLCL(11:4/16:0/18:1) | Glycerophospholipids, GP | Monolysocardiolipin | 530.3014065 | 1.643 |
| neg_265 | C55 H102 O16 P2 | MLCL(14:2/16:0/16:0) | Glycerophospholipids, GP | Monolysocardiolipin | 540.3327065 | 1.789 |
| neg_266 | C58 H98 O16 P2 | MLCL(11:2/18:1/20:4) | Glycerophospholipids, GP | Monolysocardiolipin | 556.3170565 | 1.9029 |
| neg_267 | C58 H94 O16 P2 | MLCL(11:3/18:2/20:4) | Glycerophospholipids, GP | Monolysocardiolipin | 554.3014065 | 1.4258 |
| neg_268 | C59 H106 O16 P2 | MLCL(14:2/18:1/18:1) | Glycerophospholipids, GP | Monolysocardiolipin | 566.3483565 | 1.898 |
| neg_269 | C60 H117 O16 P2 | MLCL(17:0/16:0/18:0) | Glycerophospholipids, GP | Monolysocardiolipin | 1155.78224 | 11.4007 |
| neg_270 | C63 H114 O16 P2 | MLCL(14:2/20:1/20:1) | Glycerophospholipids, GP | Monolysocardiolipin | 594.3796565 | 2.681 |
| neg_271 | C66 H121 O16 P2 | MLCL(21:1/18:1/18:2) | Glycerophospholipids, GP | Monolysocardiolipin | 1231.81354 | 10.1893 |
| neg_272 | C66 H119 O16 P2 | MLCL(21:1/18:2/18:2) | Glycerophospholipids, GP | Monolysocardiolipin | 1229.79789 | 9.5488 |
| neg_273 | C67 H127 O16 P2 | MLCL(18:2/18:0/22:0) | Glycerophospholipids, GP | Monolysocardiolipin | 1249.86049 | 10.4725 |
| neg_274 | C67 H125 O16 P2 | MLCL(18:2/18:1/22:0) | Glycerophospholipids, GP | Monolysocardiolipin | 1247.84484 | 9.89 |
| neg_275 | C68 H132 O16 P2 | MLCL(23:0/14:0/22:0) | Glycerophospholipids, GP | Monolysocardiolipin | 633.4500815 | 6.142 |
| neg_276 | C68 H123 O16 P2 | MLCL(23:1/18:2/18:2) | Glycerophospholipids, GP | Monolysocardiolipin | 1257.82919 | 10.195 |
| neg_277 | C72 H132 O16 P2 | MLCL(23:0/18:2/22:2) | Glycerophospholipids, GP | Monolysocardiolipin | 657.4500815 | 5.5243 |
| neg_280 | C32 H61 O4 | OAHFA(16:0/16:0) | Fatty Acyls, FA | (O-acyl)-1-hydroxy fatty acid | 509.4575335 | 7.8565 |
| neg_281 | C32 H59 O4 | OAHFA(18:1/14:0) | Fatty Acyls, FA | (O-acyl)-1-hydroxy fatty acid | 507.4418835 | 6.7117 |
| neg_284 | C34 H65 O4 | OAHFA(18:0/16:0) | Fatty Acyls, FA | (O-acyl)-1-hydroxy fatty acid | 537.4888335 | 8.9256 |
| neg_287 | C34 H61 O4 | OAHFA(18:2/16:0) | Fatty Acyls, FA | (O-acyl)-1-hydroxy fatty acid | 533.4575335 | 7.0157 |
| neg_288 | C34 H61 O4 | OAHFA(16:0/18:2) | Fatty Acyls, FA | (O-acyl)-1-hydroxy fatty acid | 533.4575335 | 7.261 |
| neg_289 | C34 H59 O4 | OAHFA(16:1/18:2) | Fatty Acyls, FA | (O-acyl)-1-hydroxy fatty acid | 531.4418835 | 6.2523 |
| neg_291 | C36 H67 O4 | OAHFA(18:1/18:0) | Fatty Acyls, FA | (O-acyl)-1-hydroxy fatty acid | 563.5044835 | 3.3028 |
| neg_292 | C36 H67 O4 | OAHFA(16:0/20:1) | Fatty Acyls, FA | (O-acyl)-1-hydroxy fatty acid | 563.5044835 | 8.8464 |
| neg_293 | C36 H67 O4 | OAHFA(18:0/18:1) | Fatty Acyls, FA | (O-acyl)-1-hydroxy fatty acid | 563.5044835 | 9.0426 |
| neg_294 | C36 H65 O4 | OAHFA(18:1/18:1) | Fatty Acyls, FA | (O-acyl)-1-hydroxy fatty acid | 561.4888335 | 7.9199 |
| neg_295 | C36 H65 O4 | OAHFA(18:0/18:2) | Fatty Acyls, FA | (O-acyl)-1-hydroxy fatty acid | 561.4888335 | 8.5577 |
| neg_296 | C36 H63 O4 | OAHFA(18:2/18:1) | Fatty Acyls, FA | (O-acyl)-1-hydroxy fatty acid | 559.4731835 | 2.5892 |
| neg_297 | C36 H63 O4 | OAHFA(18:1/18:2) | Fatty Acyls, FA | (O-acyl)-1-hydroxy fatty acid | 559.4731835 | 7.4267 |
| neg_298 | C36 H61 O4 | OAHFA(18:2/18:2) | Fatty Acyls, FA | (O-acyl)-1-hydroxy fatty acid | 557.4575335 | 6.4225 |
| neg_299 | C38 H73 O4 | OAHFA(16:0/22:0) | Fatty Acyls, FA | (O-acyl)-1-hydroxy fatty acid | 593.5514335 | 10.7877 |
| neg_300 | C38 H71 O4 | OAHFA(18:1/20:0) | Fatty Acyls, FA | (O-acyl)-1-hydroxy fatty acid | 591.5357835 | 9.4374 |
| neg_301 | C38 H71 O4 | OAHFA(20:0/18:1) | Fatty Acyls, FA | (O-acyl)-1-hydroxy fatty acid | 591.5357835 | 10.0642 |
| neg_302 | C38 H69 O4 | OAHFA(18:2/20:0) | Fatty Acyls, FA | (O-acyl)-1-hydroxy fatty acid | 589.5201335 | 8.6621 |
| neg_304 | C38 H69 O4 | OAHFA(20:0/18:2) | Fatty Acyls, FA | (O-acyl)-1-hydroxy fatty acid | 589.5201335 | 9.385 |
| neg_305 | C38 H67 O4 | OAHFA(18:2/20:1) | Fatty Acyls, FA | (O-acyl)-1-hydroxy fatty acid | 587.5044835 | 7.8699 |
| neg_306 | C38 H67 O4 | OAHFA(20:1/18:2) | Fatty Acyls, FA | (O-acyl)-1-hydroxy fatty acid | 587.5044835 | 8.671 |
| neg_307 | C38 H65 O4 | OAHFA(18:2/20:2) | Fatty Acyls, FA | (O-acyl)-1-hydroxy fatty acid | 585.4888335 | 7.264 |
| neg_308 | C40 H75 O4 | OAHFA(20:1/20:0) | Fatty Acyls, FA | (O-acyl)-1-hydroxy fatty acid | 619.5670835 | 4.3137 |
| neg_309 | C40 H75 O4 | OAHFA(18:1/22:0) | Fatty Acyls, FA | (O-acyl)-1-hydroxy fatty acid | 619.5670835 | 10.2785 |
| neg_310 | C40 H75 O4 | OAHFA(22:0/18:1) | Fatty Acyls, FA | (O-acyl)-1-hydroxy fatty acid | 619.5670835 | 11.015 |
| neg_311 | C40 H73 O4 | OAHFA(18:2/22:0) | Fatty Acyls, FA | (O-acyl)-1-hydroxy fatty acid | 617.5514335 | 9.5409 |
| neg_312 | C40 H73 O4 | OAHFA(22:0/18:2) | Fatty Acyls, FA | (O-acyl)-1-hydroxy fatty acid | 617.5514335 | 10.6001 |
| neg_313 | C40 H71 O4 | OAHFA(18:2/22:1) | Fatty Acyls, FA | (O-acyl)-1-hydroxy fatty acid | 615.5357835 | 8.7776 |
| neg_314 | C40 H71 O4 | OAHFA(22:1/18:2) | Fatty Acyls, FA | (O-acyl)-1-hydroxy fatty acid | 615.5357835 | 9.2386 |
| neg_315 | C42 H81 O4 | OAHFA(16:0/26:0) | Fatty Acyls, FA | (O-acyl)-1-hydroxy fatty acid | 649.6140335 | 12.5333 |
| neg_316 | C42 H79 O4 | OAHFA(18:1/24:0) | Fatty Acyls, FA | (O-acyl)-1-hydroxy fatty acid | 647.5983835 | 11.105 |
| neg_317 | C42 H77 O4 | OAHFA(18:2/24:0) | Fatty Acyls, FA | (O-acyl)-1-hydroxy fatty acid | 645.5827335 | 10.4163 |
| neg_318 | C42 H77 O4 | OAHFA(18:1/24:1) | Fatty Acyls, FA | (O-acyl)-1-hydroxy fatty acid | 645.5827335 | 11.5365 |
| neg_319 | C42 H75 O4 | OAHFA(18:2/24:1) | Fatty Acyls, FA | (O-acyl)-1-hydroxy fatty acid | 643.5670835 | 9.8595 |
| neg_320 | C44 H85 O4 | OAHFA(16:0/28:0) | Fatty Acyls, FA | (O-acyl)-1-hydroxy fatty acid | 677.6453335 | 13.3127 |
| neg_321 | C44 H83 O4 | OAHFA(18:1/26:0) | Fatty Acyls, FA | (O-acyl)-1-hydroxy fatty acid | 675.6296835 | 12.3816 |
| neg_322 | C44 H81 O4 | OAHFA(18:2/26:0) | Fatty Acyls, FA | (O-acyl)-1-hydroxy fatty acid | 673.6140335 | 11.2528 |
| neg_323 | C46 H87 O4 | OAHFA(18:1/28:0) | Fatty Acyls, FA | (O-acyl)-1-hydroxy fatty acid | 703.6609835 | 13.1405 |
| neg_324 | C46 H85 O4 | OAHFA(18:2/28:0) | Fatty Acyls, FA | (O-acyl)-1-hydroxy fatty acid | 701.6453335 | 12.5877 |
| neg_325 | C49 H91 O4 | OAHFA(18:1/31:1) | Fatty Acyls, FA | (O-acyl)-1-hydroxy fatty acid | 743.6922835 | 15.8859 |
| neg_326 | C50 H93 O4 | OAHFA(18:1/32:1) | Fatty Acyls, FA | (O-acyl)-1-hydroxy fatty acid | 757.7079335 | 13.569 |
| neg_327 | C50 H91 O4 | OAHFA(18:2/32:1) | Fatty Acyls, FA | (O-acyl)-1-hydroxy fatty acid | 755.6922835 | 13.0814 |
| neg_328 | C41 H79 O8 N1 P1 | PC(18:0/16:1) | Glycerophospholipids, GP | Phosphatidylcholine | 744.5548805 | 6.904 |
| neg_329 | C45 H87 O10 N1 P1 | PC(16:0/20:1) | Glycerophospholipids, GP | Phosphatidylcholine | 832.6073105 | 8.54 |
| neg_330 | C51 H97 O10 N1 P1 | PC(24:0/18:2) | Glycerophospholipids, GP | Phosphatidylcholine | 914.6855605 | 11.094 |
| neg_331 | C53 H103 O10 N1 P1 | PC(26:0/18:1) | Glycerophospholipids, GP | Phosphatidylcholine | 944.7325105 | 12.8317 |
| neg_332 | C53 H101 O10 N1 P1 | PC(26:0/18:2) | Glycerophospholipids, GP | Phosphatidylcholine | 942.7168605 | 12.113 |
| neg_333 | C55 H110 O8 N1 P1 Cl1 | PC(31:0/16:0) | Glycerophospholipids, GP | Phosphatidylcholine | 978.7663085 | 9.764 |
| neg_334 | C37 H71 O8 N1 P1 | PE(18:1/14:0) | Glycerophospholipids, GP | Phosphatidyl ethanolamine | 688.4922805 | 5.232 |
| neg_335 | C38 H71 O8 N1 P1 | PE(15:0/18:2) | Glycerophospholipids, GP | Phosphatidyl ethanolamine | 700.4922805 | 3.2534 |
| neg_336 | C41 H77 O8 N1 P1 | PE(18:0/18:2) | Glycerophospholipids, GP | Phosphatidyl ethanolamine | 742.5392305 | 6.9128 |
| neg_337 | C43 H81 O8 N1 P1 | PE(20:1/18:1) | Glycerophospholipids, GP | Phosphatidyl ethanolamine | 770.5705305 | 7.76 |
| neg_338 | C43 H79 O8 N1 P1 | PE(20:1/18:2) | Glycerophospholipids, GP | Phosphatidyl ethanolamine | 768.5548805 | 6.287 |
| neg_339 | C45 H87 O8 N1 P1 | PE(18:1/22:0) | Glycerophospholipids, GP | Phosphatidyl ethanolamine | 800.6174805 | 10.356 |
| neg_340 | C45 H85 O8 N1 P1 | PE(22:0/18:2) | Glycerophospholipids, GP | Phosphatidyl ethanolamine | 798.6018305 | 9.7293 |
| neg_342 | C38 H70 O8 N0 P1 | PEt(15:0/18:2) | Glycerophospholipids, GP | Phosphatidylethanol | 685.4813815 | 6.365 |
| neg_343 | C41 H76 O8 N0 P1 | PEt(18:1/18:1) | Glycerophospholipids, GP | Phosphatidylethanol | 727.5283315 | 7.27 |
| neg_344 | C41 H74 O8 N0 P1 | PEt(18:1/18:2) | Glycerophospholipids, GP | Phosphatidylethanol | 725.5126815 | 6.6069 |
| neg_345 | C41 H72 O8 N0 P1 | PEt(18:2/18:2) | Glycerophospholipids, GP | Phosphatidylethanol | 723.4970315 | 5.949 |
| neg_346 | C37 H72 O10 N0 P1 | PG(15:0/16:0) | Glycerophospholipids, GP | Phosphatidylglycerol | 707.4868615 | 4.0544 |
| neg_347 | C38 H70 O10 N0 P1 | PG(14:0/18:2) | Glycerophospholipids, GP | Phosphatidylglycerol | 717.4712115 | 4.9013 |
| neg_348 | C38 H72 O9 N0 P1 | PG(16:2e/16:0) | Glycerophospholipids, GP | Phosphatidylglycerol | 703.4919465 | 5.3787 |
| neg_349 | C39 H74 O10 N0 P1 | PG(15:0/18:1) | Glycerophospholipids, GP | Phosphatidylglycerol | 733.5025115 | 4.1193 |
| neg_350 | C39 H70 O10 N0 P1 | PG(15:0/18:3) | Glycerophospholipids, GP | Phosphatidylglycerol | 729.4712115 | 2.547 |
| neg_351 | C40 H78 O10 N0 P1 | PG(18:0/16:0) | Glycerophospholipids, GP | Phosphatidylglycerol | 749.5338115 | 7.55 |
| neg_352 | C40 H74 O9 N0 P1 | PG(16:2e/18:1) | Glycerophospholipids, GP | Phosphatidylglycerol | 729.5075965 | 5.2627 |
| neg_354 | C42 H78 O10 N0 P1 | PG(18:0/18:2) | Glycerophospholipids, GP | Phosphatidylglycerol | 773.5338115 | 6.5094 |
| neg_355 | C42 H72 O10 N0 P1 | PG(18:3/18:2) | Glycerophospholipids, GP | Phosphatidylglycerol | 767.4868615 | 4.7389 |
| neg_356 | C58 H110 O10 N0 P1 | PG(34:0/18:2) | Glycerophospholipids, GP | Phosphatidylglycerol | 997.7842115 | 8.859 |
| neg_357 | C59 H110 O10 N0 P1 | PG(35:1/18:2) | Glycerophospholipids, GP | Phosphatidylglycerol | 1009.784212 | 10.81 |
| neg_358 | C43 H80 O13 N0 P1 | PI(16:0/18:1) | Glycerophospholipids, GP | Phosphatidylinositol | 835.5342065 | 6.4644 |
| neg_359 | C43 H76 O13 N0 P1 | PI(16:0/18:3) | Glycerophospholipids, GP | Phosphatidylinositol | 831.5029065 | 5.3235 |
| neg_360 | C45 H82 O13 N0 P1 | PI(18:1/18:1) | Glycerophospholipids, GP | Phosphatidylinositol | 861.5498565 | 6.538 |
| neg_361 | C45 H76 O13 N0 P1 | PI(18:3/18:2) | Glycerophospholipids, GP | Phosphatidylinositol | 855.5029065 | 4.815 |
| neg_362 | C58 H110 O13 N0 P1 | PI(33:1/16:0) | Glycerophospholipids, GP | Phosphatidylinositol | 1045.768957 | 8.298 |
| neg_363 | C58 H108 O13 N0 P1 | PI(31:0/18:2) | Glycerophospholipids, GP | Phosphatidylinositol | 1043.753307 | 7.68 |
| neg_364 | C34 H66 O8 N0 P1 | PMe(16:0/14:0) | Glycerophospholipids, GP | Phosphatidylmethanol | 633.4500815 | 6.126 |
| neg_365 | C36 H70 O8 N0 P1 | PMe(16:0/16:0) | Glycerophospholipids, GP | Phosphatidylmethanol | 661.4813815 | 7.0016 |
| neg_366 | C36 H68 O8 N0 P1 | PMe(18:1/14:0) | Glycerophospholipids, GP | Phosphatidylmethanol | 659.4657315 | 6.1783 |
| neg_367 | C36 H66 O8 N0 P1 | PMe(14:0/18:2) | Glycerophospholipids, GP | Phosphatidylmethanol | 657.4500815 | 5.5243 |
| neg_369 | C38 H74 O8 N0 P1 | PMe(18:0/16:0) | Glycerophospholipids, GP | Phosphatidylmethanol | 689.5126815 | 7.9611 |
| neg_370 | C38 H68 O8 N0 P1 | PMe(16:1/18:2) | Glycerophospholipids, GP | Phosphatidylmethanol | 683.4657315 | 5.6146 |
| neg_371 | C38 H68 O8 N0 P1 | PMe(16:0/18:3) | Glycerophospholipids, GP | Phosphatidylmethanol | 683.4657315 | 5.8523 |
| neg_373 | C40 H76 O8 N0 P1 | PMe(18:0/18:1) | Glycerophospholipids, GP | Phosphatidylmethanol | 715.5283315 | 8.0055 |
| neg_374 | C40 H74 O8 N0 P1 | PMe(18:1/18:1) | Glycerophospholipids, GP | Phosphatidylmethanol | 713.5126815 | 7.1014 |
| neg_375 | C40 H70 O8 N0 P1 | PMe(18:2/18:2) | Glycerophospholipids, GP | Phosphatidylmethanol | 709.4813815 | 5.78 |
| neg_376 | C42 H78 O8 N0 P1 | PMe(20:1/18:1) | Glycerophospholipids, GP | Phosphatidylmethanol | 741.5439815 | 7.9597 |
| neg_377 | C42 H76 O8 N0 P1 | PMe(20:1/18:2) | Glycerophospholipids, GP | Phosphatidylmethanol | 739.5283315 | 7.3095 |
| neg_381 | C42 H75 O10 N1 P1 | PS(16:0/20:3) | Glycerophospholipids, GP | Phosphatidylserine | 784.5134105 | 6.036 |
| neg_383 | C42 H73 O10 N1 P1 | PS(16:0/20:4) | Glycerophospholipids, GP | Phosphatidylserine | 782.4977605 | 5.4355 |
| neg_384 | C42 H73 O10 N1 P1 | PS(18:2/18:2) | Glycerophospholipids, GP | Phosphatidylserine | 782.4977605 | 6.7338 |
| neg_385 | C44 H81 O10 N1 P1 | PS(20:0/18:2) | Glycerophospholipids, GP | Phosphatidylserine | 814.5603605 | 1.4 |
| neg_387 | C44 H73 O10 N1 P1 | PS(18:2/20:4) | Glycerophospholipids, GP | Phosphatidylserine | 806.4977605 | 4.9751 |
| neg_388 | C45 H85 O10 N1 P1 | PS(18:1/21:0) | Glycerophospholipids, GP | Phosphatidylserine | 830.5916605 | 7.5214 |
| neg_389 | C45 H83 O10 N1 P1 | PS(18:2/21:0) | Glycerophospholipids, GP | Phosphatidylserine | 828.5760105 | 6.7446 |
| neg_390 | C45 H81 O10 N1 P1 | PS(18:2/21:1) | Glycerophospholipids, GP | Phosphatidylserine | 826.5603605 | 6.0424 |
| neg_391 | C46 H87 O10 N1 P1 | PS(18:1/22:0) | Glycerophospholipids, GP | Phosphatidylserine | 844.6073105 | 7.5264 |
| neg_392 | C46 H83 O10 N1 P1 | PS(22:1/18:2) | Glycerophospholipids, GP | Phosphatidylserine | 840.5760105 | 6.027 |
| neg_393 | C43 H79 O12 S1 | SQDG(16:0/18:1) | Saccharolipids, SL | Sulfoquinovosyldiacylglycerol | 819.5297755 | 6.328 |
| neg_394 | C43 H77 O12 S1 | SQDG(16:0/18:2) | Saccharolipids, SL | Sulfoquinovosyldiacylglycerol | 817.5141255 | 5.7091 |
| neg_396 | C45 H73 O12 S1 | SQDG(18:2/18:4) | Saccharolipids, SL | Sulfoquinovosyldiacylglycerol | 837.4828255 | 5.196 |
| neg_397 | C50 H91 O14 S1 | SQDG(16:0/23:2) | Saccharolipids, SL | Sulfoquinovosyldiacylglycerol | 947.6135055 | 1.7945 |
| neg_398 | C51 H85 O14 S1 | SQDG(18:2/22:4) | Saccharolipids, SL | Sulfoquinovosyldiacylglycerol | 953.5665555 | 1.433 |
| neg_399 | C54 H95 O14 S1 | SQDG(18:1/25:3) | Saccharolipids, SL | Sulfoquinovosyldiacylglycerol | 999.6448055 | 1.903 |
| neg_400 | C54 H91 O14 S1 | SQDG(18:2/25:4) | Saccharolipids, SL | Sulfoquinovosyldiacylglycerol | 995.6135055 | 1.4348 |
| neg_401 | C54 H91 O14 S1 | SQDG(18:2/26:4) | Saccharolipids, SL | Sulfoquinovosyldiacylglycerol | 995.6135055 | 1.436 |
| neg_403 | C59 H101 O14 S1 | SQDG(18:2/30:4) | Saccharolipids, SL | Sulfoquinovosyldiacylglycerol | 1065.691756 | 9.9432 |
| neg_404 | C61 H113 O14 S1 | SQDG(32:0/18:2) | Saccharolipids, SL | Sulfoquinovosyldiacylglycerol | 1101.785656 | 9.564 |
| neg_405 | C60 H113 O12 S1 | SQDG(33:0/18:1) | Saccharolipids, SL | Sulfoquinovosyldiacylglycerol | 1057.795826 | 11.0719 |
| neg_406 | C60 H111 O12 S1 | SQDG(33:1/18:1) | Saccharolipids, SL | Sulfoquinovosyldiacylglycerol | 1055.780176 | 10.5296 |
| neg_407 | C60 H109 O12 S1 | SQDG(33:1/18:2) | Saccharolipids, SL | Sulfoquinovosyldiacylglycerol | 1053.764526 | 10.0064 |
| neg_408 | C19 H36 O6 N0 P1 | cPA(16:0) | Glycerophospholipids, GP | Cyclic phosphatidic acid | 391.2255015 | 1.8104 |
| neg_409 | C21 H38 O6 N0 P1 | cPA(18:1) | Glycerophospholipids, GP | Cyclic phosphatidic acid | 417.2411515 | 1.9089 |
| neg_410 | C21 H36 O6 N0 P1 | cPA(18:2) | Glycerophospholipids, GP | Cyclic phosphatidic acid | 415.2255015 | 1.4404 |
| neg_411 | C37 H69 O8 N1 P1 | dMePE(12:0/18:2) | Glycerophospholipids, GP | Dimethylphosphatidylethanolamine | 686.4766305 | 5.742 |
| neg_412 | C39 H77 O8 N1 P1 | dMePE(16:0/16:0) | Glycerophospholipids, GP | Dimethylphosphatidylethanolamine | 718.5392305 | 7.369 |
| neg_413 | C41 H79 O8 N1 P1 | dMePE(16:0/18:1) | Glycerophospholipids, GP | Dimethylphosphatidylethanolamine | 744.5548805 | 7.4399 |
| neg_414 | C41 H77 O8 N1 P1 | dMePE(16:0/18:2) | Glycerophospholipids, GP | Dimethylphosphatidylethanolamine | 742.5392305 | 6.1444 |
| neg_415 | C41 H75 O8 N1 P1 | dMePE(16:1/18:2) | Glycerophospholipids, GP | Dimethylphosphatidylethanolamine | 740.5235805 | 5.5187 |
| neg_416 | C43 H83 O8 N1 P1 | dMePE(18:0/18:1) | Glycerophospholipids, GP | Dimethylphosphatidylethanolamine | 772.5861805 | 8.5254 |
| neg_417 | C43 H81 O8 N1 P1 | dMePE(18:1/18:1) | Glycerophospholipids, GP | Dimethylphosphatidylethanolamine | 770.5705305 | 7.5189 |
| neg_418 | C43 H79 O8 N1 P1 | dMePE(18:1/18:2) | Glycerophospholipids, GP | Dimethylphosphatidylethanolamine | 768.5548805 | 6.7604 |
| neg_419 | C43 H77 O8 N1 P1 | dMePE(18:2/18:2) | Glycerophospholipids, GP | Dimethylphosphatidylethanolamine | 766.5392305 | 6.0439 |
| neg_420 | C43 H75 O8 N1 P1 | dMePE(18:3/18:2) | Glycerophospholipids, GP | Dimethylphosphatidylethanolamine | 764.5235805 | 5.496 |
| neg_422 | C41 H79 O7 N2 P1 Cl1 | phSM(d18:1/18:2) | Sphingolipids, SP | Sphingomyelin(phytosphingosine) | 777.5318925 | 7.2389 |
| neg_423 | C42 H85 O7 N2 P1 Cl1 | phSM(d19:0/18:1) | Sphingolipids, SP | Sphingomyelin(phytosphingosine) | 795.5788425 | 8.9621 |

Table S2 C0 vs S0 group differential metabolites

| ID | Metabolite | Regulate | VIP | FC(C0/S0) | P_value | FDR | C0_mean | C0_SD | S0_mean | S0_SD |
| --- | --- | --- | --- | --- | --- | --- | --- | --- | --- | --- |
| neg_9 | CL(22:2/15:0/16:0/15:1) | down | 1.4058 | 0.8206 | 3.65E-07 | 6.85E-07 | 6.128 | 0.2598 | 7.468 | 0.1047 |
| neg_11 | CL(14:4/18:1/18:1/18:2) | down | 1.4824 | 0.7744 | 6.44E-08 | 1.51E-07 | 5.048 | 0.2295 | 6.519 | 0.1135 |
| neg_28 | CL(18:2/16:0/18:2/22:6) | down | 2.015 | 0.6491 | 2.47E-12 | 3.75E-11 | 4.946 | 0.161 | 7.62 | 0.03638 |
| neg_57 | CL(22:6/16:0/18:1/22:5) | down | 1.9285 | 0.6526 | 8.08E-16 | 3.03E-14 | 4.586 | 0.06435 | 7.027 | 0.02002 |
| neg_60 | CL(18:2/18:2/20:4/22:6) | down | 3.2091 | 0.0252 | 5.05E-25 | 1.84E-22 | 0.1743 | 0.01998 | 6.928 | 0.01007 |
| neg_97 | Cer(d16:1/26:0) | up | 1.4965 | 1.2471 | 2.54E-10 | 1.70E-09 | 7.475 | 0.05832 | 5.994 | 0.1337 |
| neg_99 | Cer(d16:2/26:0) | up | 1.6144 | 1.3215 | 2.63E-08 | 6.89E-08 | 7.148 | 0.1101 | 5.409 | 0.2528 |
| neg_101 | Cer(d18:1/26:0) | up | 1.5642 | 1.307 | 7.12E-09 | 2.39E-08 | 6.931 | 0.0777 | 5.303 | 0.2117 |
| neg_144 | CerG2GNAc1(d18:1/18:0) | down | 1.5325 | 0.7871 | 1.34E-14 | 4.07E-13 | 5.698 | 0.02661 | 7.239 | 0.0497 |
| neg_155 | DGDG(18:2/16:4) | down | 3.165 | 0.0153 | 1.21E-17 | 1.10E-15 | 0.1023 | 0.01255 | 6.671 | 0.1184 |
| neg_159 | DGDG(18:2/20:9) | up | 1.4602 | 1.2376 | 1.67E-10 | 1.22E-09 | 7.345 | 0.02995 | 5.935 | 0.1297 |
| neg_194 | DGDG(26:0/30:6) | down | 1.3998 | 0.8095 | 7.17E-07 | 1.26E-06 | 5.711 | 0.301 | 7.055 | 0.02329 |
| neg_198 | DGMG(18:0) | down | 1.2655 | 0.8305 | 3.36E-06 | 5.35E-06 | 5.405 | 0.2924 | 6.508 | 0.02523 |
| neg_205 | Hex1Cer(d14:1/18:1) | down | 1.7314 | 0.7346 | 1.54E-06 | 2.59E-06 | 5.672 | 0.4093 | 7.721 | 0.2878 |
| neg_226 | LPMe(18:2) | down | 2.4663 | 0.5813 | 6.40E-12 | 8.04E-11 | 5.563 | 0.2714 | 9.57 | 0.0207 |
| neg_240 | MGDG(16:1e/18:3) | down | 1.4908 | 0.7976 | 2.57E-10 | 1.70E-09 | 5.772 | 0.1435 | 7.237 | 0.01765 |
| neg_277 | MLCL(23:0/18:2/22:2) | down | 3.1066 | 0.1334 | 1.16E-05 | 1.70E-05 | 1.043 | 2.07 | 7.818 | 0.04317 |
| neg_309 | OAHFA(18:1/22:0) | down | 1.2915 | 0.8291 | 1.18E-06 | 2.02E-06 | 5.582 | 0.2621 | 6.733 | 0.07585 |
| neg_324 | OAHFA(18:2/28:0) | down | 1.375 | 0.8054 | 2.16E-10 | 1.48E-09 | 5.172 | 0.1158 | 6.422 | 0.03568 |
| neg_335 | PE(15:0/18:2) | down | 1.4311 | 0.8156 | 3.11E-07 | 5.94E-07 | 6.132 | 0.2734 | 7.518 | 0.08029 |
| neg_342 | PEt(15:0/18:2) | down | 2.3259 | 0.6176 | 8.78E-09 | 2.88E-08 | 5.829 | 0.51 | 9.438 | 0.02939 |
| neg_344 | PEt(18:1/18:2) | up | 3.3554 | 51.8595 | 3.37E-19 | 6.14E-17 | 7.53 | 0.06266 | 0.1452 | 0.06951 |
| neg_345 | PEt(18:2/18:2) | up | 3.0615 | 7.9778 | 1.31E-05 | 1.90E-05 | 7.539 | 0.02936 | 0.945 | 2.044 |
| neg_348 | PG(16:2e/16:0) | down | 1.6768 | 0.7659 | 8.68E-14 | 2.11E-12 | 6.05 | 0.06435 | 7.899 | 0.05006 |
| neg_352 | PG(16:2e/18:1) | down | 3.2966 | 0.0272 | 5.07E-18 | 6.15E-16 | 0.1996 | 0.02234 | 7.328 | 0.1163 |
| neg_358 | PI(16:0/18:1) | up | 1.535 | 1.2905 | 9.71E-06 | 1.44E-05 | 7.409 | 0.4517 | 5.741 | 0.2135 |
| neg_359 | PI(16:0/18:3) | up | 1.5861 | 1.3253 | 3.03E-07 | 5.83E-07 | 6.958 | 0.2908 | 5.25 | 0.1948 |
| neg_360 | PI(18:1/18:1) | up | 1.3204 | 1.2409 | 0.0001245 | 0.0001684 | 6.686 | 0.5259 | 5.388 | 0.0153 |
| neg_361 | PI(18:3/18:2) | up | 2.4927 | 3.618 | 0.0006835 | 0.0008433 | 6.715 | 0.29 | 1.856 | 2.443 |
| neg_365 | PMe(16:0/16:0) | down | 2.0517 | 0.6776 | 2.21E-16 | 1.28E-14 | 5.808 | 0.06435 | 8.571 | 0.01857 |
| neg_366 | PMe(18:1/14:0) | down | 2.0239 | 0.6535 | 1.02E-15 | 3.39E-14 | 5.072 | 0.06435 | 7.761 | 0.04044 |
| neg_367 | PMe(14:0/18:2) | down | 3.1066 | 0.1334 | 1.16E-05 | 1.70E-05 | 1.043 | 2.07 | 7.818 | 0.04317 |
| neg_370 | PMe(16:1/18:2) | down | 2.0119 | 0.6662 | 3.73E-16 | 1.70E-14 | 5.303 | 0.06435 | 7.96 | 0.02157 |
| neg_373 | PMe(18:0/18:1) | down | 2.1459 | 0.6267 | 8.17E-17 | 5.95E-15 | 5.073 | 0.06435 | 8.095 | 0.01606 |
| neg_374 | PMe(18:1/18:1) | down | 2.3374 | 0.599 | 4.71E-14 | 1.32E-12 | 5.362 | 0.1481 | 8.952 | 0.01571 |
| neg_375 | PMe(18:2/18:2) | down | 2.2876 | 0.6338 | 1.37E-09 | 6.67E-09 | 6.017 | 0.4065 | 9.493 | 0.01393 |
| neg_376 | PMe(20:1/18:1) | down | 2.0063 | 0.6412 | 8.34E-16 | 3.03E-14 | 4.723 | 0.06435 | 7.366 | 0.03483 |
| pos_38 | BisMeLPA(22:0) | up | 1.5579 | 1.2155 | 5.76E-12 | 7.97E-11 | 7.259 | 0.04456 | 5.972 | 0.0742 |
| pos_41 | BisMePA(15:0/18:3) | down | 2.264 | 0.6398 | 5.16E-11 | 4.60E-10 | 4.838 | 0.2016 | 7.562 | 0.1075 |
| pos_121 | ChE(0:0) | down | 1.6713 | 0.8053 | 1.61E-11 | 1.77E-10 | 6.139 | 0.106 | 7.623 | 0.03178 |
| pos_141 | DG(10:0/11:3) | down | 1.4063 | 0.8272 | 2.22E-08 | 8.27E-08 | 5.107 | 0.01339 | 6.174 | 0.1656 |
| pos_142 | DG(9:0/12:4) | down | 1.2621 | 0.8307 | 0.001239 | 0.001856 | 5.098 | 0.4464 | 6.137 | 0.3581 |
| pos_163 | DG(18:1/11:3) | up | 1.3661 | 1.2066 | 0.0002476 | 0.0004078 | 6.635 | 0.3943 | 5.499 | 0.3115 |
| pos_177 | DG(18:2/13:0) | down | 1.4125 | 0.8255 | 9.69E-11 | 7.45E-10 | 5.029 | 0.07006 | 6.092 | 0.06414 |
| pos_198 | DG(18:4/15:0) | down | 1.4639 | 0.8287 | 7.48E-06 | 1.65E-05 | 5.85 | 0.1604 | 7.059 | 0.313 |
| pos_272 | DG(18:3/22:6) | down | 2.7932 | 0.3724 | 0.0005887 | 0.0009169 | 2.91 | 2.43 | 7.814 | 0.1092 |
| pos_274 | DG(18:1/24:1) | down | 1.5962 | 0.8297 | 1.47E-07 | 4.46E-07 | 6.774 | 0.2631 | 8.164 | 0.01768 |
| pos_298 | DGDG(8:0e/10:0) | down | 1.8411 | 0.7671 | 1.52E-09 | 7.62E-09 | 5.967 | 0.2133 | 7.779 | 0.01972 |
| pos_302 | DGDG(8:0e/12:3) | down | 1.8331 | 0.768 | 3.29E-09 | 1.51E-08 | 5.959 | 0.2285 | 7.759 | 0.02814 |
| pos_345 | Hex1Cer(d18:2/24:2) | up | 2.6978 | 3.2471 | 0.0002633 | 0.0004312 | 6.4 | 0.1156 | 1.971 | 1.971 |
| pos_377 | LPC(18:2e) | down | 1.6639 | 0.7972 | 3.17E-08 | 1.15E-07 | 5.872 | 0.2379 | 7.366 | 0.04133 |
| pos_387 | LPC(22:4) | down | 1.5932 | 0.8271 | 1.25E-10 | 9.36E-10 | 6.462 | 0.06091 | 7.813 | 0.1079 |
| pos_399 | LPMe(16:0) | down | 2.4759 | 0.5977 | 2.48E-19 | 5.84E-17 | 4.815 | 0.02874 | 8.056 | 0.02756 |
| pos_400 | LPMe(18:0) | down | 2.2659 | 0.6552 | 2.36E-18 | 3.47E-16 | 5.157 | 0.02874 | 7.871 | 0.03032 |
| pos_401 | LPMe(18:1) | down | 2.1875 | 0.6711 | 2.69E-14 | 1.32E-12 | 5.168 | 0.08093 | 7.701 | 0.05759 |
| pos_411 | MG(20:2) | down | 1.6748 | 0.8047 | 2.90E-12 | 4.69E-11 | 6.128 | 0.05192 | 7.615 | 0.07744 |
| pos_412 | MG(20:4) | up | 1.6866 | 1.2296 | 2.07E-12 | 3.70E-11 | 8.08 | 0.06906 | 6.571 | 0.06001 |
| pos_450 | PC(11:0/16:1) | up | 1.7837 | 1.3164 | 4.84E-06 | 1.11E-05 | 7.443 | 0.09146 | 5.654 | 0.4868 |
| pos_452 | PC(16:0/12:0) | up | 1.7229 | 1.2611 | 1.35E-08 | 5.30E-08 | 7.708 | 0.04898 | 6.112 | 0.231 |
| pos_460 | PC(18:1/14:0) | down | 1.9554 | 0.7698 | 1.42E-07 | 4.34E-07 | 6.935 | 0.3921 | 9.009 | 0.01261 |
| pos_464 | PC(18:2/14:1) | down | 2.5079 | 0.6437 | 7.56E-11 | 6.09E-10 | 6.04 | 0.2909 | 9.383 | 0.01715 |
| pos_511 | PE(17:1/18:1) | down | 2.2845 | 0.7059 | 3.27E-12 | 5.06E-11 | 6.647 | 0.167 | 9.416 | 0.05477 |
| pos_512 | PE(17:1/18:2) | down | 2.5226 | 0.6404 | 6.35E-11 | 5.29E-10 | 6.02 | 0.2856 | 9.401 | 0.04752 |
| pos_513 | PE(17:1/18:3) | down | 2.4111 | 0.629 | 3.53E-10 | 2.17E-09 | 5.258 | 0.3123 | 8.359 | 0.04795 |
| pos_516 | PE(18:1/18:2) | down | 1.8169 | 0.7661 | 4.95E-08 | 1.68E-07 | 5.851 | 0.2563 | 7.637 | 0.1608 |
| pos_519 | PE(18:3e/18:1) | down | 2.0717 | 0.7475 | 1.30E-09 | 6.69E-09 | 6.781 | 0.2575 | 9.072 | 0.06896 |
| pos_521 | PE(18:4/18:2) | down | 2.3304 | 0.6249 | 9.88E-13 | 2.07E-11 | 4.797 | 0.1594 | 7.676 | 0.02942 |
| pos_526 | PEt(19:1/18:1) | down | 1.6299 | 0.8252 | 6.23E-14 | 2.44E-12 | 6.638 | 0.03668 | 8.044 | 0.04746 |
| pos_531 | PG(15:0/18:2) | down | 3.1589 | 0.166 | 7.93E-06 | 1.74E-05 | 1.124 | 1.652 | 6.771 | 0.05109 |
| pos_534 | PG(16:0/18:3) | down | 2.1847 | 0.6719 | 3.14E-15 | 2.05E-13 | 5.171 | 0.02874 | 7.696 | 0.07451 |
| pos_535 | PG(18:4/16:0) | down | 1.9724 | 0.7209 | 1.07E-09 | 5.71E-09 | 5.371 | 0.1791 | 7.45 | 0.1555 |
| pos_536 | PG(18:1/18:1) | up | 1.6036 | 1.2419 | 5.30E-09 | 2.30E-08 | 7.086 | 0.1429 | 5.706 | 0.1182 |
| pos_545 | PG(28:1/16:0) | up | 1.548 | 1.2236 | 7.70E-08 | 2.48E-07 | 7.115 | 0.1468 | 5.815 | 0.1776 |
| pos_553 | PI(16:0/18:2) | up | 1.4821 | 1.2287 | 0.0001509 | 0.0002591 | 7.118 | 0.5499 | 5.793 | 0.01099 |
| pos_555 | PI(18:2/18:2) | up | 1.5719 | 1.251 | 1.23E-05 | 2.60E-05 | 7.033 | 0.4251 | 5.622 | 0.08893 |
| pos_560 | PMe(16:0/18:1) | down | 2.2617 | 0.6825 | 1.40E-19 | 5.18E-17 | 5.813 | 0.02874 | 8.517 | 0.01258 |
| pos_561 | PMe(16:0/18:2) | down | 1.7594 | 0.7927 | 7.11E-10 | 4.06E-09 | 6.308 | 0.1792 | 7.958 | 0.02156 |
| pos_564 | PMe(18:1/18:3) | down | 2.2617 | 0.6825 | 1.40E-19 | 5.18E-17 | 5.813 | 0.02874 | 8.517 | 0.01258 |
| pos_565 | PMe(18:3/18:2) | down | 1.7804 | 0.7888 | 1.23E-11 | 1.47E-10 | 6.277 | 0.12 | 7.958 | 0.02156 |
| pos_566 | PMe(18:3/18:3) | down | 2.3309 | 0.6017 | 3.86E-17 | 4.13E-15 | 4.339 | 0.02874 | 7.211 | 0.05094 |
| pos_567 | PMe(17:1/20:5) | down | 2.15 | 0.6932 | 1.81E-09 | 8.86E-09 | 5.581 | 0.293 | 8.051 | 0.05037 |
| pos_597 | SiE(20:5) | down | 1.6417 | 0.8089 | 6.00E-14 | 2.43E-12 | 6.042 | 0.03865 | 7.469 | 0.04673 |
| pos_601 | StE(19:2) | down | 1.5308 | 0.8298 | 9.37E-14 | 3.24E-12 | 6.045 | 0.02874 | 7.285 | 0.04705 |
| pos_604 | TG(6:0/6:0/9:0) | down | 1.9158 | 0.7331 | 3.96E-11 | 3.75E-10 | 5.355 | 0.1441 | 7.305 | 0.06777 |
| pos_642 | TG(15:0/6:0/10:0) | down | 1.8241 | 0.5495 | 0.01575 | 0.0203 | 3.152 | 2.156 | 5.736 | 0.3232 |
| pos_645 | TG(14:0e/8:0/9:0) | down | 3.0965 | 0.1388 | 9.00E-12 | 1.15E-10 | 0.8201 | 0.02428 | 5.91 | 0.357 |
| pos_649 | TG(12:0e/9:0/10:1) | down | 1.4964 | 0.8156 | 1.50E-07 | 4.53E-07 | 5.383 | 0.2237 | 6.6 | 0.0588 |
| pos_652 | TG(14:1e/6:0/11:0) | down | 1.5518 | 0.8149 | 3.63E-09 | 1.65E-08 | 5.679 | 0.1514 | 6.969 | 0.06978 |
| pos_656 | TG(6:0/11:3/14:2) | up | 1.9523 | 1.38 | 4.74E-18 | 6.19E-16 | 7.318 | 0.0321 | 5.303 | 0.008702 |
| pos_679 | TG(14:0e/9:0/10:2) | down | 1.2799 | 0.8296 | 0.002904 | 0.004155 | 5.388 | 0.6796 | 6.495 | 0.1349 |
| pos_711 | TG(12:1e/11:0/11:0) | down | 2.0013 | 0.7154 | 6.50E-10 | 3.75E-09 | 5.378 | 0.2315 | 7.517 | 0.01341 |
| pos_754 | TG(18:4/6:0/11:2) | up | 1.9413 | 1.3556 | 1.86E-06 | 4.58E-06 | 7.964 | 0.1668 | 5.875 | 0.4931 |
| pos_815 | TG(6:0/13:0/18:2) | down | 1.7982 | 0.7877 | 1.04E-06 | 2.68E-06 | 6.623 | 0.417 | 8.408 | 0.01795 |
| pos_817 | TG(6:0/13:0/18:3) | down | 2.311 | 0.6368 | 1.52E-12 | 2.93E-11 | 4.961 | 0.1139 | 7.791 | 0.1213 |
| pos_819 | TG(8:0/11:1/18:3) | down | 1.469 | 0.7857 | 0.000288 | 0.0004691 | 4.808 | 0.56 | 6.119 | 0.1892 |
| pos_841 | TG(18:1/10:1/10:1) | up | 1.5718 | 1.2125 | 5.97E-08 | 1.97E-07 | 7.64 | 0.06332 | 6.301 | 0.2223 |
| pos_842 | TG(10:0/10:1/18:2) | up | 1.7586 | 1.2374 | 1.33E-08 | 5.23E-08 | 8.672 | 0.09085 | 7.008 | 0.2283 |
| pos_854 | TG(8:0/12:2/18:3) | up | 1.9009 | 1.3374 | 5.37E-12 | 7.60E-11 | 7.594 | 0.01804 | 5.678 | 0.1266 |
| pos_860 | TG(11:0/10:1/18:1) | down | 2.0128 | 0.7323 | 7.40E-13 | 1.67E-11 | 5.874 | 0.1164 | 8.021 | 0.01554 |
| pos_866 | TG(16:0/11:3/12:2) | down | 1.8118 | 0.7803 | 3.10E-10 | 1.99E-09 | 6.21 | 0.1744 | 7.958 | 0.02156 |
| pos_867 | TG(18:2/10:1/11:2) | down | 2.5006 | 0.6121 | 4.14E-14 | 1.87E-12 | 5.225 | 0.1311 | 8.536 | 0.03452 |
| pos_868 | TG(10:0/11:3/18:2) | down | 2.3751 | 0.633 | 7.10E-15 | 3.63E-13 | 5.148 | 0.04172 | 8.133 | 0.09357 |
| pos_869 | TG(18:2/10:1/11:3) | down | 2.4844 | 0.5767 | 5.69E-20 | 5.18E-17 | 4.445 | 0.02874 | 7.708 | 0.01927 |
| pos_880 | TG(18:2/10:2/12:2) | down | 2.2712 | 0.6668 | 2.30E-18 | 3.47E-16 | 5.458 | 0.03101 | 8.185 | 0.02813 |
| pos_884 | TG(18:3/11:2/11:2) | down | 2.3496 | 0.648 | 1.11E-15 | 7.65E-14 | 5.376 | 0.07761 | 8.296 | 0.02998 |
| pos_898 | TG(18:2/11:2/12:4) | down | 2.3258 | 0.6499 | 1.76E-19 | 5.18E-17 | 5.309 | 0.02874 | 8.169 | 0.01808 |
| pos_902 | TG(18:4/10:3/14:3) | down | 3.1262 | 0.2469 | 0.0001902 | 0.0003182 | 1.925 | 2.511 | 7.798 | 0.01393 |
| pos_903 | TG(18:3/12:3/12:4) | down | 2.3496 | 0.648 | 1.11E-15 | 7.65E-14 | 5.376 | 0.07761 | 8.296 | 0.02998 |
| pos_912 | TG(18:2/12:2/12:3) | down | 2.3361 | 0.6532 | 5.12E-19 | 1.00E-16 | 5.433 | 0.02874 | 8.318 | 0.02504 |
| pos_913 | TG(18:1/12:3/12:3) | up | 2.5846 | 6.14 | 0.0006775 | 0.001048 | 5.001 | 2.117 | 0.8145 | 0.007372 |
| pos_914 | TG(18:2/10:1/14:4) | up | 2.1941 | 2.3615 | 0.00846 | 0.01133 | 6.147 | 0.2879 | 2.603 | 2.64 |
| pos_918 | TG(18:1/10:3/14:4) | down | 2.395 | 0.649 | 5.50E-18 | 6.47E-16 | 5.609 | 0.02874 | 8.642 | 0.0419 |
| pos_922 | TG(18:3/12:3/12:3) | down | 2.192 | 0.7116 | 2.24E-11 | 2.33E-10 | 6.295 | 0.1878 | 8.846 | 0.05824 |
| pos_937 | TG(6:0/18:1/20:2) | up | 2.666 | 3.0851 | 0.001518 | 0.002229 | 6.886 | 0.05492 | 2.232 | 2.64 |
| pos_968 | TG(22:6/12:4/12:4) | down | 1.8597 | 0.7466 | 1.23E-16 | 1.11E-14 | 5.393 | 0.02874 | 7.223 | 0.03038 |
| pos_1060 | TG(18:4/13:0/18:4) | up | 1.6986 | 1.2324 | 6.21E-09 | 2.64E-08 | 8.209 | 0.09808 | 6.661 | 0.1873 |
| pos_1115 | TG(16:0/17:1/18:3) | down | 1.9376 | 0.732 | 8.74E-08 | 2.76E-07 | 5.556 | 0.3408 | 7.59 | 0.1319 |
| pos_1132 | TG(18:3/11:1/22:3) | down | 1.713 | 0.8125 | 2.37E-11 | 2.44E-10 | 6.75 | 0.1198 | 8.308 | 0.01545 |
| pos_1210 | TG(17:0/18:2/18:3) | down | 2.7403 | 0.338 | 8.50E-05 | 0.0001502 | 2.267 | 1.707 | 6.707 | 0.1776 |
| pos_1219 | TG(18:4/17:1/18:3) | up | 1.9525 | 1.347 | 2.70E-13 | 7.23E-12 | 7.838 | 0.06531 | 5.819 | 0.07545 |
| pos_1232 | TG(18:4/18:3/18:3) | down | 2.0436 | 0.7142 | 2.27E-08 | 8.39E-08 | 5.633 | 0.2407 | 7.887 | 0.2566 |
| pos_1258 | TG(18:3/18:3/18:3) | up | 2.0754 | 1.4105 | 3.39E-08 | 1.22E-07 | 7.999 | 0.2488 | 5.671 | 0.2858 |
| pos_1266 | TG(22:4/11:1/22:5) | up | 1.6948 | 1.2678 | 5.18E-06 | 1.17E-05 | 7.642 | 0.1434 | 6.028 | 0.427 |
| pos_1274 | TG(15:0/18:2/22:1) | down | 2.337 | 0.3723 | 0.0004791 | 0.0007573 | 2.021 | 0.02846 | 5.428 | 1.643 |
| pos_1278 | TG(19:0/18:2/18:2) | down | 1.6307 | 0.7991 | 7.10E-09 | 2.94E-08 | 5.681 | 0.02874 | 7.109 | 0.1956 |
| pos_1279 | TG(19:0/18:1/18:3) | down | 2.9607 | 0.3286 | 1.36E-11 | 1.58E-10 | 2.277 | 0.02858 | 6.93 | 0.3398 |
| pos_1296 | TG(20:5/17:1/18:2) | up | 1.7931 | 1.2317 | 2.61E-10 | 1.72E-09 | 9.112 | 0.01279 | 7.398 | 0.1688 |
| pos_1353 | TG(18:1/18:2/21:0) | down | 1.7307 | 0.1059 | 0.04726 | 0.05717 | 0.3219 | 0.01484 | 3.041 | 2.945 |
| pos_1355 | TG(14:1e/19:1/24:1) | down | 3.3577 | 0.0464 | 2.92E-14 | 1.37E-12 | 0.2902 | 0.0138 | 6.258 | 0.2356 |
| pos_1358 | TG(19:1/18:2/20:1) | down | 2.647 | 0.2149 | 0.0004791 | 0.0007573 | 1.192 | 0.02684 | 5.546 | 2.1 |
| pos_1406 | TG(18:3e/18:1/23:0) | down | 1.8236 | 0.7507 | 6.30E-10 | 3.67E-09 | 5.351 | 0.1645 | 7.128 | 0.09897 |
| pos_1451 | TG(26:0/18:0/18:0) | down | 1.3835 | 0.7866 | 0.01274 | 0.01665 | 5.33 | 1.13 | 6.776 | 0.3052 |
| pos_1525 | TG(30:1/18:3/22:6) | down | 2.0916 | 0.3239 | 0.01903 | 0.02428 | 1.675 | 2.075 | 5.172 | 2.259 |
| pos_1533 | TG(29:1/20:1/22:6) | down | 1.5232 | 0.8326 | 2.29E-06 | 5.56E-06 | 6.411 | 0.2999 | 7.7 | 0.1346 |
| pos_1547 | ZyE(21:2) | down | 1.5317 | 0.8295 | 9.24E-14 | 3.24E-12 | 6.043 | 0.02874 | 7.285 | 0.04704 |
| pos_204 | DG(16:0/18:1) | down | 2.0525 | 0.6919 | 5.78E-07 | 1.54E-06 | 5.203 | 0.5073 | 7.52 | 0.03723 |

Table S3 S9 vs S0 group differential metabolites

| ID | Metabolite | Regulate | VIP | FC(S9/S0) | P_value | FDR | S9_mean | S9_SD | S0_mean | S0_SD |
| --- | --- | --- | --- | --- | --- | --- | --- | --- | --- | --- |
| neg_22 | CL(18:2/18:1/18:2/18:2) | down | 2.463 | 0.4523 | 0.01558 | 0.01872 | 2.436 | 2.482 | 5.386 | 0.09281 |
| neg_68 | CL(24:0/16:0/18:2/22:0) | down | 2.1994 | 0.7461 | 1.10E-11 | 3.64E-10 | 4.819 | 0.105 | 6.459 | 0.05298 |
| neg_99 | Cer(d16:2/26:0) | up | 2.1745 | 1.3019 | 2.34E-08 | 9.05E-08 | 7.042 | 0.03909 | 5.409 | 0.2528 |
| neg_325 | OAHFA(18:1/31:1) | down | 1.888 | 0.8196 | 1.88E-12 | 1.22E-10 | 5.479 | 0.0713 | 6.685 | 0.01276 |
| neg_356 | PG(34:0/18:2) | up | 1.5536 | 1.215 | 0.007023 | 0.008754 | 6.352 | 0.506 | 5.228 | 0.6382 |
| pos_39 | BisMePA(18:0/12:0) | down | 1.855 | 0.7355 | 5.91E-08 | 1.55E-07 | 6.299 | 0.3682 | 8.564 | 0.1307 |
| pos_40 | BisMePA(15:0/18:2) | up | 1.6077 | 1.3102 | 9.04E-09 | 2.82E-08 | 7.129 | 0.1975 | 5.441 | 0.1358 |
| pos_126 | CmE(20:5) | up | 1.7155 | 1.3126 | 2.98E-12 | 2.71E-11 | 7.961 | 0.08018 | 6.065 | 0.08825 |
| pos_133 | DG(6:0/10:0) | down | 1.477 | 0.7966 | 2.47E-05 | 3.97E-05 | 5.959 | 0.5069 | 7.481 | 0.02967 |
| pos_141 | DG(10:0/11:3) | up | 1.6277 | 1.2781 | 3.84E-10 | 1.72E-09 | 7.891 | 0.06072 | 6.174 | 0.1656 |
| pos_142 | DG(9:0/12:4) | up | 1.5501 | 1.2622 | 8.71E-07 | 1.82E-06 | 7.746 | 0.08963 | 6.137 | 0.3581 |
| pos_149 | DG(19:0/6:0) | down | 1.6036 | 0.7998 | 1.33E-09 | 5.08E-09 | 6.671 | 0.1729 | 8.341 | 0.08942 |
| pos_150 | DG(19:1/6:0) | down | 1.4926 | 0.8291 | 1.03E-09 | 4.03E-09 | 7.015 | 0.1618 | 8.461 | 0.02862 |
| pos_161 | DG(15:0/14:0) | up | 1.6645 | 1.3065 | 2.08E-08 | 6.12E-08 | 7.733 | 0.04039 | 5.919 | 0.2776 |
| pos_166 | DG(16:0/14:0) | down | 1.4741 | 0.8123 | 2.04E-05 | 3.33E-05 | 6.563 | 0.4946 | 8.08 | 0.01499 |
| pos_168 | DG(18:3/12:1) | down | 1.6599 | 0.7769 | 1.10E-07 | 2.66E-07 | 6.332 | 0.331 | 8.15 | 0.04777 |
| pos_173 | DG(15:0/16:0) | up | 1.7332 | 1.3066 | 8.51E-08 | 2.12E-07 | 8.442 | 0.1625 | 6.461 | 0.3156 |
| pos_177 | DG(18:2/13:0) | up | 1.5301 | 1.248 | 2.01E-11 | 1.38E-10 | 7.603 | 0.09568 | 6.092 | 0.06414 |
| pos_194 | DG(27:0/6:0) | up | 1.6389 | 1.2568 | 8.02E-12 | 6.09E-11 | 8.472 | 0.02762 | 6.741 | 0.1171 |
| pos_249 | DG(19:1/18:2) | down | 1.6657 | 0.7841 | 4.03E-11 | 2.42E-10 | 6.503 | 0.1444 | 8.294 | 0.02485 |
| pos_254 | DG(18:1/20:2) | down | 1.5032 | 0.82 | 8.80E-09 | 2.75E-08 | 6.715 | 0.2071 | 8.189 | 0.02515 |
| pos_262 | DG(20:4e/18:2) | up | 1.8344 | 1.3767 | 4.61E-12 | 3.76E-11 | 7.93 | 0.03793 | 5.76 | 0.1374 |
| pos_328 | Hex1Cer(d12:0/18:1) | down | 1.5915 | 0.7845 | 1.03E-06 | 2.11E-06 | 6.191 | 0.3953 | 7.892 | 0.04078 |
| pos_360 | Hex1SPH(t18:1) | up | 1.7249 | 1.3351 | 3.84E-12 | 3.27E-11 | 7.638 | 0.07589 | 5.721 | 0.09773 |
| pos_411 | MG(20:2) | down | 1.599 | 0.7796 | 4.28E-08 | 1.16E-07 | 5.937 | 0.2688 | 7.615 | 0.07744 |
| pos_412 | MG(20:4) | down | 1.798 | 0.5386 | 0.0143 | 0.01732 | 3.539 | 2.509 | 6.571 | 0.06001 |
| pos_451 | PC(9:0/18:2) | down | 1.2954 | 0.8154 | 8.57E-05 | 0.0001311 | 5.332 | 0.4074 | 6.539 | 0.2284 |
| pos_549 | PG(28:0/18:3) | down | 1.3499 | 0.8316 | 3.65E-12 | 3.18E-11 | 5.799 | 0.06115 | 6.973 | 0.04414 |
| pos_550 | PG(28:1/18:2) | down | 1.4863 | 0.8002 | 7.24E-09 | 2.32E-08 | 5.773 | 0.1973 | 7.214 | 0.03244 |
| pos_603 | StE(37:6) | down | 1.5545 | 0.7993 | 1.99E-10 | 9.46E-10 | 6.229 | 0.1425 | 7.793 | 0.04788 |
| pos_620 | TG(11:0/6:0/10:2) | down | 1.6941 | 0.7652 | 6.18E-15 | 3.03E-13 | 6.012 | 0.06115 | 7.857 | 0.01258 |
| pos_628 | TG(6:0/9:0/12:3) | down | 1.5675 | 0.7975 | 9.54E-14 | 1.97E-12 | 6.223 | 0.06115 | 7.803 | 0.03481 |
| pos_632 | TG(14:0e/6:0/9:0) | up | 1.6418 | 1.2849 | 6.70E-10 | 2.74E-09 | 7.883 | 0.04098 | 6.135 | 0.1857 |
| pos_638 | TG(12:1e/8:0/10:3) | down | 1.6705 | 0.7747 | 8.09E-08 | 2.04E-07 | 6.319 | 0.3236 | 8.157 | 0.05098 |
| pos_642 | TG(15:0/6:0/10:0) | up | 1.6368 | 1.3089 | 1.41E-07 | 3.33E-07 | 7.508 | 0.0879 | 5.736 | 0.3232 |
| pos_645 | TG(14:0e/8:0/9:0) | up | 1.7112 | 1.3294 | 4.04E-07 | 8.90E-07 | 7.857 | 0.2045 | 5.91 | 0.357 |
| pos_649 | TG(12:0e/9:0/10:1) | up | 1.4631 | 1.2088 | 5.06E-13 | 6.69E-12 | 7.978 | 0.04249 | 6.6 | 0.0588 |
| pos_652 | TG(14:1e/6:0/11:0) | up | 1.62 | 1.2424 | 7.26E-13 | 8.81E-12 | 8.658 | 0.06024 | 6.969 | 0.06978 |
| pos_660 | TG(12:0e/10:0/10:2) | down | 1.578 | 0.7865 | 1.23E-10 | 6.10E-10 | 5.928 | 0.1285 | 7.537 | 0.07215 |
| pos_665 | TG(15:0/9:0/9:0) | up | 1.6559 | 1.2578 | 9.05E-13 | 1.02E-11 | 8.617 | 0.03484 | 6.851 | 0.09218 |
| pos_666 | TG(15:0/6:0/12:0) | up | 1.6389 | 1.2541 | 1.98E-14 | 7.28E-13 | 8.523 | 0.04951 | 6.796 | 0.04315 |
| pos_669 | TG(15:0/6:0/12:1) | up | 1.6818 | 1.2596 | 1.40E-12 | 1.42E-11 | 8.836 | 0.0674 | 7.015 | 0.08198 |
| pos_673 | TG(12:1e/6:0/15:0) | up | 1.8766 | 1.3811 | 1.27E-08 | 3.85E-08 | 8.342 | 0.0485 | 6.04 | 0.335 |
| pos_674 | TG(9:0/12:1/12:1) | up | 1.5013 | 1.2109 | 6.13E-11 | 3.34E-10 | 8.365 | 0.1148 | 6.908 | 0.04771 |
| pos_681 | TG(6:0/10:2/17:1) | up | 1.4114 | 1.2135 | 1.35E-07 | 3.19E-07 | 7.492 | 0.03098 | 6.174 | 0.2459 |
| pos_683 | TG(4:0/11:1/18:2) | up | 1.5447 | 1.2631 | 1.12E-06 | 2.27E-06 | 7.686 | 0.0601 | 6.085 | 0.3727 |
| pos_703 | TG(14:1e/9:0/10:4) | up | 1.6673 | 1.2772 | 1.04E-05 | 1.79E-05 | 8.815 | 0.04446 | 6.902 | 0.5755 |
| pos_706 | TG(16:0/9:0/9:0) | down | 1.6565 | 0.7847 | 1.64E-10 | 7.89E-10 | 6.47 | 0.1642 | 8.245 | 0.03165 |
| pos_722 | TG(14:0e/10:0/10:2) | down | 1.5344 | 0.7303 | 0.002728 | 0.003576 | 5.213 | 1.175 | 7.138 | 0.2099 |
| pos_735 | TG(4:0/12:3/18:3) | down | 1.4213 | 0.8298 | 6.70E-06 | 1.22E-05 | 6.755 | 0.3975 | 8.141 | 0.02454 |
| pos_737 | TG(16:0/9:0/10:0) | down | 1.4592 | 0.8248 | 9.10E-08 | 2.25E-07 | 6.607 | 0.1521 | 8.01 | 0.2025 |
| pos_739 | TG(14:0e/9:0/12:1) | up | 1.5654 | 1.233 | 1.59E-13 | 2.74E-12 | 8.34 | 0.02556 | 6.764 | 0.06927 |
| pos_743 | TG(16:0e/9:0/10:2) | up | 1.8718 | 1.3773 | 5.74E-11 | 3.16E-10 | 8.261 | 0.02398 | 5.998 | 0.1903 |
| pos_745 | TG(14:1e/9:0/12:1) | up | 1.574 | 1.247 | 2.14E-09 | 7.77E-09 | 8.139 | 0.1748 | 6.527 | 0.09135 |
| pos_758 | TG(4:0/16:0/16:0) | down | 1.4291 | 0.8313 | 5.43E-09 | 1.78E-08 | 6.565 | 0.1785 | 7.897 | 0.01841 |
| pos_760 | TG(18:0/9:0/9:0) | down | 1.6107 | 0.783 | 4.42E-08 | 1.19E-07 | 6.149 | 0.2833 | 7.853 | 0.03219 |
| pos_776 | TG(9:0/9:0/18:3) | down | 1.4503 | 0.8206 | 1.30E-05 | 2.20E-05 | 6.646 | 0.3926 | 8.099 | 0.2196 |
| pos_790 | TG(6:0/12:1/18:3) | down | 1.6368 | 0.7829 | 4.65E-08 | 1.25E-07 | 6.354 | 0.2936 | 8.116 | 0.04038 |
| pos_814 | TG(9:0/10:0/18:2) | down | 1.6008 | 0.8133 | 3.25E-09 | 1.13E-08 | 7.267 | 0.1387 | 8.935 | 0.1619 |
| pos_819 | TG(8:0/11:1/18:3) | up | 1.6553 | 1.2956 | 8.64E-08 | 2.14E-07 | 7.928 | 0.264 | 6.119 | 0.1892 |
| pos_830 | TG(20:0/6:0/12:0) | down | 1.6668 | 0.7685 | 1.71E-09 | 6.35E-09 | 5.986 | 0.2124 | 7.789 | 0.03813 |
| pos_831 | TG(6:0/14:0/18:1) | down | 1.7197 | 0.7578 | 1.56E-13 | 2.74E-12 | 5.952 | 0.08574 | 7.854 | 0.02385 |
| pos_832 | TG(20:1/6:0/12:0) | down | 1.7743 | 0.7469 | 2.77E-14 | 8.15E-13 | 5.974 | 0.06087 | 7.998 | 0.05133 |
| pos_835 | TG(4:0/16:0/18:2) | down | 1.508 | 0.8249 | 3.07E-10 | 1.40E-09 | 6.929 | 0.1177 | 8.4 | 0.08943 |
| pos_838 | TG(18:0/10:1/10:1) | down | 1.5306 | 0.7804 | 3.11E-06 | 5.95E-06 | 5.654 | 0.406 | 7.245 | 0.1045 |
| pos_844 | TG(12:1e/6:0/20:2) | down | 1.506 | 0.8195 | 8.39E-09 | 2.65E-08 | 6.715 | 0.2071 | 8.194 | 0.02288 |
| pos_845 | TG(14:0e/6:0/18:3) | down | 1.668 | 0.7797 | 2.82E-09 | 9.91E-09 | 6.402 | 0.2076 | 8.211 | 0.09356 |
| pos_871 | TG(18:3/10:1/11:2) | down | 1.522 | 0.7883 | 8.96E-08 | 2.22E-07 | 5.681 | 0.2001 | 7.207 | 0.1886 |
| pos_873 | TG(18:2/10:2/11:4) | down | 1.5185 | 0.8192 | 1.10E-05 | 1.88E-05 | 7.2 | 0.3955 | 8.789 | 0.2765 |
| pos_876 | TG(6:0/12:0/22:1) | down | 1.726 | 0.7485 | 1.32E-07 | 3.14E-07 | 5.848 | 0.3628 | 7.813 | 0.06687 |
| pos_928 | TG(16:0/11:3/16:0) | down | 1.5465 | 0.7836 | 6.91E-09 | 2.23E-08 | 5.652 | 0.2114 | 7.213 | 0.04188 |
| pos_929 | TG(16:0/11:4/16:0) | down | 2.016 | 0.6551 | 1.37E-15 | 1.01E-13 | 4.959 | 0.06115 | 7.57 | 0.04513 |
| pos_942 | TG(6:0/18:1/20:4) | down | 1.6376 | 0.7696 | 1.28E-11 | 9.27E-11 | 5.776 | 0.1121 | 7.505 | 0.05746 |
| pos_950 | TG(18:1/10:1/17:1) | down | 1.4366 | 0.8145 | 5.58E-08 | 1.48E-07 | 5.963 | 0.1864 | 7.321 | 0.1397 |
| pos_957 | TG(18:4/9:0/18:1) | down | 1.8584 | 0.7221 | 6.62E-14 | 1.54E-12 | 5.771 | 0.09476 | 7.992 | 0.01061 |
| pos_983 | TG(18:3/10:2/18:2) | down | 1.4625 | 0.8199 | 9.65E-10 | 3.80E-09 | 6.313 | 0.1552 | 7.7 | 0.02057 |
| pos_984 | TG(18:2/10:3/18:2) | down | 1.56 | 0.7893 | 2.89E-08 | 8.13E-08 | 5.98 | 0.244 | 7.576 | 0.07599 |
| pos_988 | TG(15:0/16:0/16:0) | up | 2.0817 | 1.4827 | 8.13E-15 | 3.41E-13 | 8.558 | 0.06521 | 5.772 | 0.07166 |
| pos_991 | TG(15:0/14:0/18:1) | up | 2.0453 | 1.5085 | 4.43E-15 | 2.37E-13 | 7.977 | 0.04689 | 5.288 | 0.0745 |
| pos_1006 | TG(18:3/11:2/18:2) | down | 1.5538 | 0.7861 | 1.71E-10 | 8.21E-10 | 5.737 | 0.1325 | 7.298 | 0.06546 |
| pos_1007 | TG(18:3e/11:1/18:3) | down | 1.6412 | 0.7628 | 5.74E-09 | 1.87E-08 | 5.64 | 0.2009 | 7.394 | 0.127 |
| pos_1012 | TG(16:0/14:0/18:3) | down | 1.6884 | 0.7637 | 3.73E-11 | 2.29E-10 | 5.95 | 0.1396 | 7.791 | 0.05315 |
| pos_1016 | TG(18:1/12:1/18:3) | down | 1.3876 | 0.8272 | 2.80E-06 | 5.38E-06 | 6.234 | 0.2976 | 7.536 | 0.1635 |
| pos_1018 | TG(18:1/10:1/20:4) | down | 1.3005 | 0.828 | 7.49E-06 | 1.35E-05 | 5.584 | 0.06115 | 6.744 | 0.3319 |
| pos_1020 | TG(18:2/12:2/18:2) | down | 1.4032 | 0.8276 | 1.68E-07 | 3.93E-07 | 6.257 | 0.1466 | 7.56 | 0.2037 |
| pos_1027 | TG(15:0/16:0/18:1) | up | 1.8971 | 1.3244 | 1.00E-14 | 3.98E-13 | 9.448 | 0.04023 | 7.134 | 0.07167 |
| pos_1032 | TG(15:0/16:0/18:2) | up | 1.9036 | 1.3342 | 3.67E-12 | 3.18E-11 | 9.317 | 0.08121 | 6.983 | 0.1261 |
| pos_1042 | TG(18:3/10:1/21:0) | up | 1.695 | 1.2775 | 2.33E-12 | 2.19E-11 | 8.526 | 0.09155 | 6.674 | 0.06728 |
| pos_1070 | TG(16:2e/11:3/22:4) | down | 1.587 | 0.814 | 1.62E-09 | 6.14E-09 | 7.156 | 0.1746 | 8.791 | 0.08609 |
| pos_1074 | TG(15:0/17:0/18:1) | up | 3.0903 | 5.0536 | 1.14E-05 | 1.95E-05 | 8.197 | 0.03346 | 1.622 | 2.006 |
| pos_1077 | TG(16:0/16:1/18:1) | down | 1.4319 | 0.8149 | 1.42E-07 | 3.33E-07 | 5.957 | 0.2472 | 7.31 | 0.06656 |
| pos_1079 | TG(16:0/16:0/18:3) | down | 1.5397 | 0.8189 | 1.23E-09 | 4.75E-09 | 6.96 | 0.1771 | 8.499 | 0.0184 |
| pos_1088 | TG(18:4/16:0/16:0) | down | 1.5339 | 0.798 | 1.19E-09 | 4.61E-09 | 6.037 | 0.1276 | 7.565 | 0.1215 |
| pos_1092 | TG(16:0/17:0/18:1) | up | 3.2399 | 9.6558 | 2.10E-20 | 1.24E-17 | 7.519 | 0.06429 | 0.7787 | 0.007258 |
| pos_1099 | TG(18:3e/11:1/22:6) | down | 1.5893 | 0.8198 | 1.10E-05 | 1.89E-05 | 7.933 | 0.5297 | 9.677 | 0.01549 |
| pos_1103 | TG(15:0/18:1/18:1) | up | 1.9348 | 1.3278 | 4.02E-15 | 2.25E-13 | 9.745 | 0.04051 | 7.339 | 0.06664 |
| pos_1115 | TG(16:0/17:1/18:3) | up | 1.6458 | 1.2323 | 3.71E-09 | 1.27E-08 | 9.353 | 0.1864 | 7.59 | 0.1319 |
| pos_1129 | TG(18:4/15:0/18:2) | down | 1.3378 | 0.8316 | 7.05E-05 | 0.0001084 | 6.304 | 0.4163 | 7.581 | 0.2442 |
| pos_1166 | TG(16:1/18:1/18:2) | down | 2.0754 | 0.6795 | 7.26E-09 | 2.32E-08 | 5.955 | 0.3895 | 8.764 | 0.01592 |
| pos_1170 | TG(6:0/22:3/24:2) | up | 1.8164 | 1.3292 | 1.18E-07 | 2.84E-07 | 8.803 | 0.3955 | 6.623 | 0.08411 |
| pos_1174 | TG(16:2e/16:0/20:3) | down | 3.3235 | 0.1229 | 3.30E-21 | 3.88E-18 | 0.9936 | 0.05501 | 8.086 | 0.01322 |
| pos_1176 | TG(6:0/22:6/24:0) | up | 1.9375 | 1.3753 | 7.93E-08 | 2.02E-07 | 9.058 | 0.3376 | 6.586 | 0.2817 |
| pos_1177 | TG(16:0/18:3/18:3) | down | 1.5116 | 0.8267 | 1.65E-11 | 1.16E-10 | 7.026 | 0.1086 | 8.499 | 0.01842 |
| pos_1180 | TG(18:3e/16:0/18:3) | down | 1.4321 | 0.8268 | 3.92E-07 | 8.66E-07 | 6.501 | 0.2608 | 7.863 | 0.1196 |
| pos_1181 | TG(6:0/22:6/24:1) | up | 1.7758 | 1.3306 | 2.16E-09 | 7.84E-09 | 8.243 | 0.1001 | 6.195 | 0.2303 |
| pos_1184 | TG(16:1/18:3/18:3) | up | 1.6355 | 1.2344 | 1.96E-09 | 7.23E-09 | 9.157 | 0.1869 | 7.418 | 0.09799 |
| pos_1192 | TG(20:0/15:0/18:1) | up | 1.6415 | 1.2929 | 1.57E-12 | 1.56E-11 | 7.663 | 0.0922 | 5.927 | 0.04445 |
| pos_1201 | TG(15:0/18:2/20:1) | up | 1.7366 | 1.2962 | 2.70E-09 | 9.57E-09 | 8.591 | 0.05193 | 6.628 | 0.2406 |
| pos_1250 | TG(18:4/18:1/18:2) | down | 1.9488 | 0.7157 | 1.05E-08 | 3.24E-08 | 6.242 | 0.3449 | 8.721 | 0.09426 |
| pos_1261 | TG(18:3e/18:3/18:3) | up | 2.2741 | 1.6253 | 4.26E-17 | 5.56E-15 | 8.632 | 0.06773 | 5.311 | 0.008702 |
| pos_1271 | TG(19:0/18:1/18:1) | up | 1.5432 | 1.2406 | 4.76E-13 | 6.37E-12 | 7.899 | 0.07966 | 6.367 | 0.008702 |
| pos_1274 | TG(15:0/18:2/22:1) | up | 1.7138 | 1.4574 | 0.004127 | 0.005299 | 7.911 | 0.08438 | 5.428 | 1.643 |
| pos_1277 | TG(16:2e/18:1/21:0) | up | 2.4983 | 2.7266 | 0.001397 | 0.001902 | 7.82 | 0.133 | 2.868 | 2.772 |
| pos_1281 | TG(15:0/18:3/22:1) | down | 1.534 | 0.8106 | 9.03E-13 | 1.02E-11 | 6.485 | 0.07183 | 8 | 0.04454 |
| pos_1298 | TG(15:0/18:2/22:6) | down | 1.3957 | 0.8203 | 4.48E-07 | 9.80E-07 | 5.925 | 0.2724 | 7.223 | 0.05186 |
| pos_1305 | TG(19:1/18:4/18:4) | down | 1.3559 | 0.8274 | 1.87E-05 | 3.07E-05 | 6.144 | 0.3605 | 7.426 | 0.203 |
| pos_1318 | TG(20:1/18:1/18:1) | down | 1.6636 | 0.7694 | 2.55E-10 | 1.18E-09 | 5.975 | 0.1752 | 7.766 | 0.02199 |
| pos_1353 | TG(18:1/18:2/21:0) | up | 2.3248 | 2.4923 | 0.003707 | 0.004801 | 7.579 | 0.226 | 3.041 | 2.945 |
| pos_1355 | TG(14:1e/19:1/24:1) | up | 1.7592 | 1.3213 | 1.70E-09 | 6.35E-09 | 8.269 | 0.04821 | 6.258 | 0.2356 |
| pos_1356 | TG(16:2e/18:1/23:0) | up | 1.6608 | 1.2809 | 2.01E-11 | 1.38E-10 | 8.117 | 0.03945 | 6.337 | 0.1298 |
| pos_1358 | TG(19:1/18:2/20:1) | up | 1.6796 | 1.4704 | 0.01242 | 0.01512 | 8.155 | 0.06391 | 5.546 | 2.1 |
| pos_1361 | TG(19:0/18:1/20:4) | down | 1.7733 | 0.7261 | 2.77E-07 | 6.30E-07 | 5.54 | 0.4225 | 7.63 | 0.03789 |
| pos_1366 | TG(18:3/18:3/21:1) | down | 1.5678 | 0.777 | 7.19E-06 | 1.30E-05 | 5.87 | 0.4821 | 7.555 | 0.07454 |
| pos_1404 | TG(18:1/18:2/23:1) | down | 1.5213 | 0.8025 | 1.47E-06 | 2.92E-06 | 6.337 | 0.3627 | 7.897 | 0.1094 |
| pos_1406 | TG(18:3e/18:1/23:0) | up | 1.5036 | 1.2044 | 1.28E-11 | 9.27E-11 | 8.585 | 0.0385 | 7.128 | 0.09897 |
| pos_1408 | TG(18:3e/18:2/23:0) | up | 1.5717 | 1.2351 | 8.01E-12 | 6.09E-11 | 8.364 | 0.03159 | 6.772 | 0.106 |
| pos_1413 | TG(29:1/12:4/18:3) | down | 1.8151 | 0.7225 | 8.80E-10 | 3.50E-09 | 5.558 | 0.2161 | 7.693 | 0.1017 |
| pos_1432 | TG(18:2e/18:2/24:1) | up | 1.8014 | 1.3556 | 9.36E-18 | 1.57E-15 | 7.945 | 0.03577 | 5.861 | 0.008702 |
| pos_1447 | TG(20:4e/18:1/23:0) | down | 1.4101 | 0.8179 | 7.04E-06 | 1.27E-05 | 6.135 | 0.388 | 7.501 | 0.07201 |
| pos_1448 | TG(18:1/21:0/22:6) | down | 1.4456 | 0.8242 | 1.09E-07 | 2.64E-07 | 6.473 | 0.2095 | 7.854 | 0.1433 |
| pos_1451 | TG(26:0/18:0/18:0) | down | 1.6171 | 0.7425 | 6.39E-07 | 1.36E-06 | 5.031 | 0.2385 | 6.776 | 0.3052 |
| pos_1458 | TG(26:0/18:1/18:3) | down | 2.8172 | 0.2063 | 7.25E-05 | 0.0001114 | 1.474 | 2.15 | 7.146 | 0.06716 |
| pos_1462 | TG(18:3/22:1/22:1) | down | 1.5044 | 0.8313 | 3.34E-13 | 4.73E-12 | 7.171 | 0.07297 | 8.626 | 0.0088 |
| pos_1467 | TG(27:0/18:0/18:1) | down | 1.2972 | 0.8288 | 1.34E-06 | 2.69E-06 | 5.469 | 0.2079 | 6.599 | 0.1751 |
| pos_1471 | TG(27:0/18:2/18:2) | down | 1.4321 | 0.8222 | 2.28E-08 | 6.63E-08 | 6.204 | 0.2085 | 7.546 | 0.02304 |
| pos_1474 | TG(18:1/22:1/24:1) | down | 1.4595 | 0.8247 | 1.20E-10 | 6.00E-10 | 6.476 | 0.1063 | 7.853 | 0.06736 |
| pos_1476 | TG(24:1/18:2/22:1) | down | 1.5921 | 0.7941 | 3.05E-10 | 1.40E-09 | 6.328 | 0.1633 | 7.969 | 0.02173 |
| pos_1479 | TG(24:1/18:3/22:1) | down | 1.9025 | 0.6943 | 9.60E-08 | 2.35E-07 | 5.42 | 0.4296 | 7.806 | 0.05482 |
| pos_1484 | TG(29:0/18:1/18:1) | down | 1.3269 | 0.825 | 1.68E-07 | 3.93E-07 | 5.494 | 0.1038 | 6.659 | 0.1988 |
| pos_1489 | TG(18:1/24:0/24:0) | down | 1.4861 | 0.7933 | 2.14E-08 | 6.28E-08 | 5.551 | 0.1922 | 6.997 | 0.1158 |
| pos_1492 | TG(30:1/18:1/18:2) | down | 1.549 | 0.7825 | 9.75E-09 | 3.03E-08 | 5.641 | 0.2235 | 7.209 | 0.01905 |
| pos_1493 | TG(30:0/18:2/18:2) | down | 1.478 | 0.8039 | 8.10E-10 | 3.28E-09 | 5.804 | 0.1525 | 7.22 | 0.03743 |
| pos_1495 | TG(30:1/18:3/18:4) | up | 1.7952 | 1.3502 | 5.28E-08 | 1.40E-07 | 8.182 | 0.3615 | 6.06 | 0.008702 |
| pos_1509 | TG(26:0/18:1/24:0) | down | 2.5358 | 0.252 | 0.0007924 | 0.001107 | 1.672 | 2.564 | 6.634 | 0.05401 |
| pos_1510 | TG(26:0/18:1/24:1) | down | 1.4631 | 0.7982 | 8.77E-11 | 4.54E-10 | 5.471 | 0.1179 | 6.854 | 0.03253 |
| pos_1514 | TG(26:1/18:1/24:2) | down | 1.6276 | 0.7568 | 4.75E-09 | 1.58E-08 | 5.365 | 0.2277 | 7.089 | 0.02542 |
| pos_1525 | TG(30:1/18:3/22:6) | up | 1.7856 | 1.5607 | 0.01049 | 0.01289 | 8.072 | 0.1072 | 5.172 | 2.259 |
| pos_1528 | TG(28:0/18:1/24:2) | down | 1.4437 | 0.7938 | 6.39E-09 | 2.07E-08 | 5.232 | 0.06115 | 6.591 | 0.1758 |
| pos_1529 | TG(28:0/18:2/24:2) | down | 1.4474 | 0.7975 | 1.35E-09 | 5.16E-09 | 5.351 | 0.156 | 6.71 | 0.0295 |
| pos_1535 | TG(30:1/18:2/24:2) | down | 1.4579 | 0.8159 | 5.01E-10 | 2.15E-09 | 6.102 | 0.1326 | 7.479 | 0.05982 |
| pos_1540 | WE(3:0/20:1) | up | 1.5489 | 1.224 | 2.53E-13 | 4.08E-12 | 8.438 | 0.05401 | 6.894 | 0.05321 |
| pos_1544 | WE(3:0/22:2) | up | 1.5408 | 1.2299 | 1.03E-12 | 1.12E-11 | 8.181 | 0.05234 | 6.652 | 0.06878 |
| pos_1546 | ZyE(16:2) | up | 1.4965 | 1.2224 | 3.13E-08 | 8.76E-08 | 8.062 | 0.198 | 6.595 | 0.1301 |
| pos_1549 | ZyE(22:2) | up | 1.5522 | 1.2457 | 2.44E-08 | 7.00E-08 | 8 | 0.03882 | 6.422 | 0.2452 |
| pos_1552 | ZyE(35:6) | down | 1.443 | 0.8332 | 1.46E-11 | 1.04E-10 | 6.712 | 0.07445 | 8.056 | 0.06549 |

Table S4 C9 vs C0 group differential metabolites

| ID | Metabolite | Regulate | VIP | FC(C9/C0) | P_value | FDR | C9_mean | C9_SD | C0_mean | C0_SD |
| --- | --- | --- | --- | --- | --- | --- | --- | --- | --- | --- |
| neg_9 | CL(22:2/15:0/16:0/15:1) | up | 3.3281 | 1.2604 | 5.38E-08 | 1.12E-06 | 7.724 | 0.08248 | 6.128 | 0.2598 |
| neg_60 | CL(18:2/18:2/20:4/22:6) | up | 3.1191 | 13.8325 | 0.04759 | 0.09955 | 2.411 | 2.427 | 0.1743 | 0.01998 |
| neg_143 | CerG2GNAc1(d19:0/16:0) | up | 3.8992 | 1.3736 | 1.78E-12 | 6.48E-10 | 7.882 | 0.02921 | 5.738 | 0.1247 |
| neg_221 | LBPA(16:0/18:0) | up | 2.7922 | 1.2186 | 0.0009058 | 0.003545 | 7.414 | 0.2218 | 6.084 | 0.6645 |
| neg_246 | MGDG(16:0/23:5) | up | 3.711 | 1.3899 | 1.38E-05 | 9.84E-05 | 7.401 | 0.6248 | 5.325 | 0.1692 |
| neg_247 | MGDG(18:3/22:0) | up | 3.5353 | 1.2763 | 1.22E-11 | 2.23E-09 | 8.176 | 0.04135 | 6.406 | 0.1215 |
| neg_250 | MGDG(18:1/28:3) | up | 3.2454 | 1.2351 | 4.70E-09 | 2.44E-07 | 7.879 | 0.02739 | 6.379 | 0.1972 |
| neg_335 | PE(15:0/18:2) | up | 3.397 | 1.2707 | 5.02E-08 | 1.12E-06 | 7.792 | 0.06732 | 6.132 | 0.2734 |
| neg_346 | PG(15:0/16:0) | up | 3.2194 | 1.2558 | 1.06E-09 | 6.45E-08 | 7.25 | 0.02458 | 5.773 | 0.1666 |
| neg_349 | PG(15:0/18:1) | up | 3.3449 | 1.2981 | 9.62E-11 | 8.81E-09 | 6.924 | 0.06278 | 5.334 | 0.1273 |
| neg_351 | PG(18:0/16:0) | up | 2.7922 | 1.2186 | 0.0009058 | 0.003545 | 7.414 | 0.2218 | 6.084 | 0.6645 |
| pos_38 | BisMeLPA(22:0) | down | 2.3367 | 0.7803 | 1.47E-08 | 1.44E-07 | 5.664 | 0.2339 | 7.259 | 0.04456 |
| pos_40 | BisMePA(15:0/18:2) | up | 1.963 | 1.2331 | 8.98E-07 | 4.55E-06 | 6.125 | 0.2394 | 4.967 | 0.1173 |
| pos_141 | DG(10:0/11:3) | up | 2.0384 | 1.2356 | 8.32E-11 | 1.85E-09 | 6.31 | 0.105 | 5.107 | 0.01339 |
| pos_261 | DG(16:0/22:6) | up | 2.4455 | 1.2384 | 1.12E-13 | 1.28E-11 | 8.95 | 0.04655 | 7.227 | 0.06252 |
| pos_270 | DG(18:1/22:6) | up | 2.3925 | 1.2082 | 2.69E-16 | 7.90E-14 | 9.556 | 0.01567 | 7.909 | 0.03757 |
| pos_289 | DG(36:0/18:3) | down | 2.3699 | 0.7869 | 5.40E-09 | 6.10E-08 | 6.051 | 0.1796 | 7.69 | 0.1281 |
| pos_334 | Hex1Cer(d18:1/20:1) | up | 2.811 | 1.4372 | 1.95E-08 | 1.75E-07 | 7.613 | 0.05843 | 5.297 | 0.3511 |
| pos_344 | Hex1Cer(d18:2/24:1) | up | 2.4084 | 1.2144 | 1.49E-13 | 1.28E-11 | 9.47 | 0.0191 | 7.798 | 0.07544 |
| pos_357 | Hex1Cer(t18:1/24:1) | up | 2.4084 | 1.2144 | 1.49E-13 | 1.28E-11 | 9.47 | 0.0191 | 7.798 | 0.07544 |
| pos_531 | PG(15:0/18:2) | up | 4.2935 | 6.0516 | 7.58E-06 | 2.73E-05 | 6.802 | 0.07208 | 1.124 | 1.652 |
| pos_566 | PMe(18:3/18:3) | up | 1.8008 | 1.2459 | 0.000189 | 0.0004822 | 5.406 | 0.4549 | 4.339 | 0.02874 |
| pos_577 | SM(d18:1/24:6) | up | 2.6572 | 1.3238 | 7.36E-12 | 3.46E-10 | 8.34 | 0.04143 | 6.3 | 0.1343 |
| pos_610 | TG(6:0/6:0/13:0) | up | 2.0559 | 1.2211 | 5.19E-06 | 1.98E-05 | 7.168 | 0.2192 | 5.87 | 0.2884 |
| pos_613 | TG(8:0/8:0/10:0) | up | 2.4256 | 1.2777 | 2.51E-11 | 7.90E-10 | 7.826 | 0.025 | 6.125 | 0.1303 |
| pos_619 | TG(6:0/10:1/11:1) | up | 2.618 | 1.3201 | 1.41E-09 | 1.92E-08 | 8.22 | 0.1121 | 6.227 | 0.2052 |
| pos_639 | TG(6:0/12:2/12:3) | up | 2.8182 | 1.3952 | 1.68E-10 | 3.29E-09 | 8.106 | 0.03232 | 5.81 | 0.2146 |
| pos_641 | TG(6:0/12:3/12:3) | up | 2.9536 | 1.4898 | 2.20E-11 | 7.39E-10 | 7.671 | 0.1086 | 5.149 | 0.1607 |
| pos_662 | TG(6:0/12:2/14:4) | up | 2.908 | 1.4342 | 1.49E-10 | 3.06E-09 | 8.089 | 0.05466 | 5.64 | 0.222 |
| pos_663 | TG(8:0/10:2/14:4) | up | 2.6093 | 1.3568 | 7.93E-08 | 5.52E-07 | 7.617 | 0.1004 | 5.614 | 0.3418 |
| pos_819 | TG(8:0/11:1/18:3) | up | 2.6214 | 1.4359 | 5.71E-06 | 2.14E-05 | 6.904 | 0.1901 | 4.808 | 0.56 |
| pos_869 | TG(18:2/10:1/11:3) | up | 2.5338 | 1.4205 | 3.81E-09 | 4.66E-08 | 6.314 | 0.2411 | 4.445 | 0.02874 |
| pos_875 | TG(16:0/8:0/16:0) | up | 2.2633 | 1.2919 | 0.0004952 | 0.001149 | 7.648 | 0.05192 | 5.92 | 0.8359 |
| pos_881 | TG(18:2/10:3/12:1) | up | 4.6367 | 5.8487 | 3.23E-05 | 9.77E-05 | 8.159 | 0.04129 | 1.395 | 2.328 |
| pos_882 | TG(4:0/18:3/18:3) | up | 3.1697 | 1.5023 | 2.64E-11 | 7.96E-10 | 8.665 | 0.02371 | 5.768 | 0.2258 |
| pos_883 | TG(18:2/11:2/11:2) | up | 3.093 | 1.4639 | 4.23E-09 | 5.03E-08 | 8.808 | 0.01261 | 6.017 | 0.3663 |
| pos_885 | TG(18:2/11:1/11:4) | up | 4.3913 | 4.0142 | 8.44E-06 | 2.99E-05 | 7.94 | 0.3065 | 1.978 | 1.731 |
| pos_886 | TG(18:2/11:2/11:3) | up | 3.0054 | 1.4344 | 3.13E-11 | 8.77E-10 | 8.602 | 0.05432 | 5.997 | 0.2005 |
| pos_892 | TG(16:0/11:3/14:3) | up | 2.6953 | 1.3663 | 8.76E-15 | 1.29E-12 | 7.803 | 0.03294 | 5.711 | 0.06548 |
| pos_897 | TG(12:1e/11:2/18:4) | up | 2.6589 | 1.3167 | 2.70E-13 | 2.04E-11 | 8.461 | 0.01712 | 6.426 | 0.09908 |
| pos_900 | TG(12:1e/11:3/18:4) | up | 2.776 | 1.3765 | 1.08E-11 | 4.26E-10 | 8.134 | 0.1076 | 5.909 | 0.1176 |
| pos_905 | TG(16:0/10:2/16:0) | up | 2.2851 | 1.2289 | 3.09E-09 | 3.91E-08 | 8.177 | 0.1065 | 6.654 | 0.1617 |
| pos_909 | TG(12:0e/12:4/18:1) | down | 2.4189 | 0.6071 | 0.03514 | 0.05244 | 4.15 | 2.701 | 6.836 | 0.04675 |
| pos_913 | TG(18:1/12:3/12:3) | up | 3.0443 | 1.6775 | 0.002871 | 0.005646 | 8.389 | 0.04577 | 5.001 | 2.117 |
| pos_914 | TG(18:2/10:1/14:4) | up | 3.0211 | 1.4321 | 1.16E-09 | 1.62E-08 | 8.803 | 0.1019 | 6.147 | 0.2879 |
| pos_915 | TG(18:1/10:2/14:4) | up | 2.6409 | 1.2905 | 2.52E-11 | 7.90E-10 | 8.947 | 0.1477 | 6.933 | 0.05347 |
| pos_921 | TG(18:4/6:0/18:4) | up | 2.5792 | 1.2804 | 2.77E-13 | 2.04E-11 | 8.762 | 0.01912 | 6.843 | 0.09309 |
| pos_930 | TG(18:1/11:3/14:3) | up | 2.4638 | 1.2854 | 1.49E-13 | 1.28E-11 | 7.877 | 0.01737 | 6.128 | 0.0796 |
| pos_939 | TG(8:0/18:2/18:2) | up | 2.8087 | 1.3994 | 3.47E-15 | 5.83E-13 | 7.947 | 0.03821 | 5.679 | 0.06154 |
| pos_943 | TG(16:0/10:4/18:2) | up | 2.3578 | 1.2513 | 1.47E-10 | 3.06E-09 | 8.008 | 0.1182 | 6.4 | 0.09204 |
| pos_956 | TG(16:0/11:4/18:1) | up | 2.7342 | 1.3655 | 9.74E-16 | 1.91E-13 | 8.032 | 0.05322 | 5.882 | 0.02874 |
| pos_962 | TG(18:4/9:0/18:4) | up | 2.5334 | 1.2895 | 7.16E-10 | 1.07E-08 | 8.307 | 0.1542 | 6.442 | 0.1338 |
| pos_982 | TG(18:1/10:3/18:3) | up | 2.2113 | 1.2282 | 2.31E-07 | 1.39E-06 | 7.8 | 0.11 | 6.351 | 0.2669 |
| pos_1242 | TG(18:1/18:1/18:3) | up | 2.5614 | 1.2598 | 4.95E-13 | 3.24E-11 | 9.17 | 0.05267 | 7.279 | 0.08421 |
| pos_1301 | TG(18:3e/18:4/19:1) | up | 3.0818 | 1.5482 | 3.96E-16 | 9.32E-14 | 7.716 | 0.06406 | 4.984 | 0.02874 |
| pos_1305 | TG(19:1/18:4/18:4) | down | 2.2954 | 0.8157 | 1.65E-08 | 1.58E-07 | 6.825 | 0.2259 | 8.367 | 0.05676 |
| pos_1313 | TG(18:4/18:3/20:5) | down | 2.0788 | 0.8312 | 2.72E-07 | 1.58E-06 | 6.311 | 0.228 | 7.593 | 0.124 |
| pos_1371 | TG(18:4/18:3/21:1) | up | 2.8909 | 1.4215 | 2.69E-17 | 1.06E-14 | 8.104 | 0.03743 | 5.701 | 0.02874 |
| pos_1411 | TG(19:1/20:1/20:5) | up | 2.6416 | 1.362 | 8.32E-18 | 4.89E-15 | 7.547 | 0.02005 | 5.541 | 0.02874 |
| pos_1464 | TG(18:4/22:1/22:1) | up | 5.0379 | 19.7686 | 6.13E-20 | 7.21E-17 | 7.688 | 0.07616 | 0.3889 | 0.0168 |

Table S5 C9 vs S9 group differential metabolites

| ID | Metabolite | Regulate | VIP | FC(C9/S9) | P_value | FDR | C9_mean | C9_SD | S9_mean | S9_SD |
| --- | --- | --- | --- | --- | --- | --- | --- | --- | --- | --- |
| neg_11 | CL(14:4/18:1/18:1/18:2) | down | 1.4231 | 0.751 | 7.70E-10 | 2.06E-09 | 5.497 | 0.07807 | 7.32 | 0.1853 |
| neg_22 | CL(18:2/18:1/18:2/18:2) | up | 1.7308 | 2.4766 | 0.006127 | 0.006502 | 6.033 | 0.5689 | 2.436 | 2.482 |
| neg_24 | CL(23:0/16:0/16:0/18:1) | up | 1.4095 | 1.3264 | 7.46E-12 | 4.53E-11 | 7.218 | 0.04313 | 5.442 | 0.1147 |
| neg_28 | CL(18:2/16:0/18:2/22:6) | down | 1.6624 | 0.6708 | 8.56E-12 | 4.94E-11 | 5.033 | 0.1606 | 7.503 | 0.06394 |
| neg_41 | CL(23:0/16:0/18:1/18:1) | up | 1.219 | 1.2348 | 3.78E-08 | 6.84E-08 | 7.121 | 0.06198 | 5.767 | 0.2141 |
| neg_47 | CL(21:1/18:1/18:2/18:2) | down | 1.0994 | 0.8179 | 0.0001046 | 0.0001209 | 5.432 | 0.4016 | 6.641 | 0.2619 |
| neg_51 | CL(23:1/18:2/18:2/18:2) | up | 1.22 | 1.2172 | 4.68E-12 | 3.10E-11 | 7.465 | 0.01898 | 6.133 | 0.0856 |
| neg_54 | CL(19:0/18:1/18:2/22:6) | down | 1.1505 | 0.8302 | 1.49E-10 | 4.91E-10 | 5.81 | 0.1019 | 6.998 | 0.04368 |
| neg_57 | CL(22:6/16:0/18:1/22:5) | down | 1.6335 | 0.6636 | 1.08E-13 | 2.03E-12 | 4.694 | 0.06794 | 7.074 | 0.08302 |
| neg_60 | CL(18:2/18:2/20:4/22:6) | down | 1.9835 | 0.3577 | 0.001402 | 0.001533 | 2.411 | 2.427 | 6.74 | 0.05666 |
| neg_64 | CL(18:3/18:2/20:0/22:4) | up | 1.409 | 1.3118 | 4.89E-13 | 5.56E-12 | 7.456 | 0.06238 | 5.684 | 0.06888 |
| neg_68 | CL(24:0/16:0/18:2/22:0) | up | 1.9137 | 1.6775 | 1.09E-14 | 3.96E-13 | 8.084 | 0.05136 | 4.819 | 0.105 |
| neg_143 | CerG2GNAc1(d19:0/16:0) | up | 1.5948 | 1.409 | 4.56E-10 | 1.30E-09 | 7.882 | 0.02921 | 5.594 | 0.2374 |
| neg_144 | CerG2GNAc1(d18:1/18:0) | down | 1.1781 | 0.8225 | 1.61E-14 | 4.88E-13 | 5.736 | 0.04157 | 6.974 | 0.01995 |
| neg_149 | DGDG(16:0/16:2) | up | 1.4328 | 1.3395 | 1.56E-08 | 3.13E-08 | 7.366 | 0.229 | 5.499 | 0.1617 |
| neg_155 | DGDG(18:2/16:4) | down | 2.3592 | 0.1457 | 4.20E-05 | 5.03E-05 | 0.9325 | 1.928 | 6.4 | 0.2259 |
| neg_159 | DGDG(18:2/20:9) | up | 1.3221 | 1.2808 | 3.41E-08 | 6.24E-08 | 7.23 | 0.02167 | 5.645 | 0.2572 |
| neg_205 | Hex1Cer(d14:1/18:1) | down | 1.526 | 0.6968 | 5.78E-06 | 7.52E-06 | 5.082 | 0.3571 | 7.293 | 0.5124 |
| neg_226 | LPMe(18:2) | down | 2.0547 | 0.5962 | 1.54E-14 | 4.88E-13 | 5.56 | 0.1269 | 9.325 | 0.05813 |
| neg_235 | MGDG(16:0/13:0) | up | 1.291 | 1.2178 | 9.57E-13 | 8.50E-12 | 8.319 | 0.06832 | 6.831 | 0.04803 |
| neg_237 | MGDG(18:1/13:0) | up | 1.3919 | 1.2593 | 2.71E-13 | 3.61E-12 | 8.397 | 0.02241 | 6.668 | 0.08249 |
| neg_238 | MGDG(18:2/13:0) | up | 1.2647 | 1.2329 | 5.30E-05 | 6.28E-05 | 8.344 | 0.06706 | 6.768 | 0.5716 |
| neg_240 | MGDG(16:1e/18:3) | down | 1.2604 | 0.8002 | 9.00E-08 | 1.49E-07 | 5.826 | 0.1928 | 7.281 | 0.1779 |
| neg_245 | MGDG(20:1/18:2) | up | 1.245 | 1.2413 | 4.59E-08 | 7.91E-08 | 7.249 | 0.03612 | 5.84 | 0.234 |
| neg_246 | MGDG(16:0/23:5) | up | 1.6728 | 1.5454 | 1.42E-06 | 1.94E-06 | 7.401 | 0.6248 | 4.789 | 0.09464 |
| neg_247 | MGDG(18:3/22:0) | up | 1.6579 | 1.4286 | 1.13E-12 | 9.31E-12 | 8.176 | 0.04135 | 5.723 | 0.1337 |
| neg_250 | MGDG(18:1/28:3) | up | 1.4994 | 1.3427 | 3.64E-11 | 1.56E-10 | 7.879 | 0.02739 | 5.868 | 0.1605 |
| neg_277 | MLCL(23:0/18:2/22:2) | down | 2.5677 | 0.1738 | 1.35E-05 | 1.68E-05 | 1.329 | 1.964 | 7.647 | 0.07886 |
| neg_300 | OAHFA(18:1/20:0) | down | 1.208 | 0.8238 | 4.70E-09 | 1.03E-08 | 6.168 | 0.1423 | 7.487 | 0.1019 |
| neg_309 | OAHFA(18:1/22:0) | down | 1.3695 | 0.7704 | 4.84E-09 | 1.05E-08 | 5.691 | 0.1913 | 7.387 | 0.1198 |
| neg_316 | OAHFA(18:1/24:0) | down | 1.2589 | 0.8043 | 3.58E-11 | 1.55E-10 | 5.833 | 0.1075 | 7.252 | 0.03975 |
| neg_324 | OAHFA(18:2/28:0) | down | 1.1984 | 0.8205 | 2.35E-13 | 3.29E-12 | 5.861 | 0.0507 | 7.143 | 0.03653 |
| neg_330 | PC(24:0/18:2) | up | 1.1582 | 1.2074 | 2.91E-06 | 3.88E-06 | 7.305 | 0.01296 | 6.05 | 0.3283 |
| neg_333 | PC(31:0/16:0) | up | 1.2356 | 1.222 | 2.09E-11 | 1.00E-10 | 7.526 | 0.07987 | 6.159 | 0.06757 |
| neg_342 | PEt(15:0/18:2) | down | 1.9589 | 0.6263 | 3.13E-11 | 1.41E-10 | 5.753 | 0.2732 | 9.185 | 0.01445 |
| neg_344 | PEt(18:1/18:2) | up | 2.8924 | 86.0534 | 5.21E-22 | 1.90E-19 | 7.54 | 0.04663 | 0.08762 | 0.0164 |
| neg_345 | PEt(18:2/18:2) | up | 2.8534 | 31.345 | 1.06E-14 | 3.96E-13 | 7.504 | 0.03289 | 0.2394 | 0.2573 |
| neg_348 | PG(16:2e/16:0) | down | 1.3378 | 0.7928 | 1.70E-11 | 8.73E-11 | 6.131 | 0.04157 | 7.733 | 0.1127 |
| neg_352 | PG(16:2e/18:1) | down | 2.2211 | 0.2672 | 0.0005741 | 0.000641 | 1.905 | 2.582 | 7.129 | 0.0413 |
| neg_356 | PG(34:0/18:2) | down | 1.2476 | 0.7624 | 2.66E-05 | 3.24E-05 | 4.843 | 0.04157 | 6.352 | 0.506 |
| neg_358 | PI(16:0/18:1) | up | 1.3778 | 1.2947 | 2.23E-07 | 3.41E-07 | 7.666 | 0.2569 | 5.921 | 0.2319 |
| neg_359 | PI(16:0/18:3) | up | 1.5249 | 1.4181 | 5.70E-10 | 1.56E-09 | 7.096 | 0.1913 | 5.004 | 0.1161 |
| neg_360 | PI(18:1/18:1) | up | 1.417 | 1.348 | 2.39E-08 | 4.56E-08 | 7.069 | 0.2727 | 5.244 | 0.08782 |
| neg_361 | PI(18:3/18:2) | up | 2.4165 | 6.3491 | 5.87E-05 | 6.94E-05 | 6.838 | 0.1557 | 1.077 | 2.124 |
| neg_365 | PMe(16:0/16:0) | down | 1.6852 | 0.6994 | 4.02E-17 | 3.66E-15 | 5.889 | 0.04157 | 8.42 | 0.0308 |
| neg_366 | PMe(18:1/14:0) | down | 1.6973 | 0.6675 | 3.24E-17 | 3.66E-15 | 5.153 | 0.04157 | 7.72 | 0.03016 |
| neg_367 | PMe(14:0/18:2) | down | 2.5677 | 0.1738 | 1.35E-05 | 1.68E-05 | 1.329 | 1.964 | 7.647 | 0.07886 |
| neg_370 | PMe(16:1/18:2) | down | 1.6343 | 0.6937 | 1.36E-15 | 8.22E-14 | 5.389 | 0.04892 | 7.769 | 0.04894 |
| neg_373 | PMe(18:0/18:1) | down | 1.7592 | 0.6514 | 1.42E-17 | 2.58E-15 | 5.153 | 0.04157 | 7.911 | 0.02918 |
| neg_374 | PMe(18:1/18:1) | down | 1.9248 | 0.6209 | 4.31E-15 | 1.96E-13 | 5.41 | 0.104 | 8.713 | 0.02828 |
| neg_375 | PMe(18:2/18:2) | down | 2.019 | 0.6052 | 1.95E-12 | 1.48E-11 | 5.581 | 0.2157 | 9.221 | 0.04026 |
| neg_376 | PMe(20:1/18:1) | down | 1.6087 | 0.6781 | 3.71E-13 | 4.35E-12 | 4.864 | 0.1073 | 7.173 | 0.04861 |
| neg_391 | PS(18:1/22:0) | up | 1.1836 | 1.2005 | 1.51E-10 | 4.94E-10 | 7.526 | 0.03395 | 6.269 | 0.1125 |
| neg_393 | SQDG(16:0/18:1) | up | 1.1665 | 1.2046 | 3.11E-08 | 5.78E-08 | 7.288 | 0.02518 | 6.05 | 0.1982 |
| neg_412 | dMePE(16:0/16:0) | up | 1.4366 | 1.2942 | 5.89E-10 | 1.60E-09 | 8.16 | 0.03897 | 6.305 | 0.1952 |
| neg_420 | dMePE(18:3/18:2) | up | 1.4095 | 1.312 | 1.53E-13 | 2.52E-12 | 7.452 | 0.0477 | 5.68 | 0.06756 |
| neg_422 | phSM(d18:1/18:2) | down | 1.2594 | 0.7798 | 2.73E-05 | 3.31E-05 | 5.442 | 0.5144 | 6.979 | 0.06574 |
| pos_7 | AcHexChE(28:2) | up | 1.1853 | 1.2704 | 2.77E-12 | 1.51E-11 | 7.535 | 0.07927 | 5.931 | 0.06115 |
| pos_33 | AcHexZyE(24:2) | up | 1.2944 | 1.3046 | 3.74E-06 | 5.39E-06 | 8.635 | 0.4393 | 6.619 | 0.3185 |
| pos_39 | BisMePA(18:0/12:0) | up | 1.3414 | 1.3342 | 1.10E-07 | 1.98E-07 | 8.404 | 0.1208 | 6.299 | 0.3682 |
| pos_41 | BisMePA(15:0/18:3) | down | 1.4002 | 0.7139 | 1.18E-11 | 5.46E-11 | 5.59 | 0.1507 | 7.83 | 0.05908 |
| pos_66 | Cer(d20:0/18:1) | down | 1.1654 | 0.8149 | 5.75E-09 | 1.30E-08 | 6.908 | 0.2086 | 8.477 | 0.04094 |
| pos_68 | Cer(d20:0/18:2) | down | 1.3333 | 0.7531 | 1.21E-10 | 4.05E-10 | 6.208 | 0.1836 | 8.243 | 0.03002 |
| pos_76 | Cer(d22:0/18:1) | down | 1.137 | 0.8308 | 1.25E-12 | 7.68E-12 | 7.237 | 0.06933 | 8.711 | 0.04922 |
| pos_77 | Cer(d22:0/18:2) | down | 1.3234 | 0.763 | 2.12E-12 | 1.21E-11 | 6.433 | 0.118 | 8.431 | 0.02885 |
| pos_93 | Cer(t20:1/18:0) | down | 1.3137 | 0.7715 | 7.16E-12 | 3.58E-11 | 6.655 | 0.1315 | 8.626 | 0.03233 |
| pos_105 | Cer(t20:1/20:1) | down | 1.3407 | 0.7484 | 1.38E-08 | 2.88E-08 | 6.197 | 0.3015 | 8.28 | 0.0674 |
| pos_106 | Cer(t18:0/22:5) | down | 1.4567 | 0.7122 | 7.37E-10 | 1.99E-09 | 6.033 | 0.2361 | 8.471 | 0.1264 |
| pos_117 | Cer(t18:0/26:0) | down | 1.2882 | 0.7676 | 1.11E-10 | 3.79E-10 | 6.28 | 0.1675 | 8.181 | 0.04014 |
| pos_121 | ChE(0:0) | down | 1.4379 | 0.7196 | 1.58E-16 | 4.31E-15 | 6.038 | 0.03087 | 8.391 | 0.04571 |
| pos_123 | ChE(20:5) | down | 1.169 | 0.8 | 9.42E-14 | 9.39E-13 | 6.228 | 0.03087 | 7.785 | 0.06197 |
| pos_126 | CmE(20:5) | down | 1.3727 | 0.7304 | 3.31E-14 | 3.85E-13 | 5.815 | 0.03087 | 7.961 | 0.08018 |
| pos_127 | CmE(8:0) | down | 1.345 | 0.7686 | 2.02E-15 | 3.44E-14 | 6.839 | 0.04308 | 8.898 | 0.04502 |
| pos_136 | DG(8:1e/10:0) | down | 1.2387 | 0.7866 | 1.03E-09 | 2.69E-09 | 6.502 | 0.1756 | 8.266 | 0.09661 |
| pos_141 | DG(10:0/11:3) | down | 1.1757 | 0.7996 | 2.15E-11 | 9.03E-11 | 6.31 | 0.105 | 7.891 | 0.06072 |
| pos_142 | DG(9:0/12:4) | down | 1.2558 | 0.7472 | 2.71E-05 | 3.59E-05 | 5.788 | 0.654 | 7.746 | 0.08963 |
| pos_145 | DG(16:0/8:0) | down | 1.3691 | 0.7324 | 1.60E-08 | 3.26E-08 | 5.942 | 0.3199 | 8.113 | 0.06716 |
| pos_146 | DG(18:2e/6:0) | down | 1.0935 | 0.8254 | 4.99E-11 | 1.88E-10 | 6.465 | 0.1097 | 7.833 | 0.03219 |
| pos_148 | DG(18:3e/6:0) | down | 1.1764 | 0.8106 | 1.86E-12 | 1.10E-11 | 6.756 | 0.07573 | 8.335 | 0.0569 |
| pos_154 | DG(18:4/9:0) | up | 1.2692 | 1.2941 | 1.66E-07 | 2.92E-07 | 8.304 | 0.03195 | 6.417 | 0.3615 |
| pos_161 | DG(15:0/14:0) | down | 1.459 | 0.6867 | 8.95E-17 | 2.84E-15 | 5.31 | 0.03531 | 7.733 | 0.04039 |
| pos_163 | DG(18:1/11:3) | up | 1.479 | 1.4552 | 4.08E-15 | 6.76E-14 | 7.96 | 0.05289 | 5.47 | 0.06115 |
| pos_165 | DG(6:0/24:0) | down | 1.2272 | 0.7858 | 1.84E-08 | 3.72E-08 | 6.406 | 0.2472 | 8.152 | 0.1005 |
| pos_168 | DG(18:3/12:1) | up | 1.2023 | 1.2686 | 3.12E-07 | 5.27E-07 | 8.033 | 0.1127 | 6.332 | 0.331 |
| pos_175 | DG(15:0/16:1) | down | 1.2027 | 0.7993 | 6.56E-14 | 6.77E-13 | 6.564 | 0.04435 | 8.212 | 0.05502 |
| pos_176 | DG(20:0e/11:1) | down | 1.221 | 0.8308 | 3.96E-18 | 2.71E-16 | 8.325 | 0.01772 | 10.02 | 0.02103 |
| pos_177 | DG(18:2/13:0) | down | 1.3646 | 0.7175 | 3.43E-09 | 8.07E-09 | 5.455 | 0.259 | 7.603 | 0.09568 |
| pos_187 | DG(26:1/6:0) | down | 1.2902 | 0.7636 | 8.19E-12 | 3.98E-11 | 6.142 | 0.1111 | 8.043 | 0.07205 |
| pos_194 | DG(27:0/6:0) | down | 1.5503 | 0.6767 | 1.14E-13 | 1.08E-12 | 5.733 | 0.121 | 8.472 | 0.02762 |
| pos_195 | DG(15:0/18:1) | down | 1.4063 | 0.7743 | 2.14E-18 | 1.94E-16 | 7.72 | 0.03234 | 9.97 | 0.01146 |
| pos_196 | DG(15:0/18:2) | down | 1.3831 | 0.7821 | 1.20E-16 | 3.51E-15 | 7.816 | 0.04618 | 9.993 | 0.01816 |
| pos_197 | DG(15:0/18:3) | down | 1.2369 | 0.8031 | 2.08E-12 | 1.20E-11 | 7.118 | 0.06782 | 8.863 | 0.08135 |
| pos_198 | DG(18:4/15:0) | down | 1.5593 | 0.6524 | 9.07E-07 | 1.42E-06 | 5.414 | 0.6385 | 8.299 | 0.1854 |
| pos_239 | DG(18:2e/18:2) | down | 1.2785 | 0.7696 | 1.05E-14 | 1.47E-13 | 6.218 | 0.05791 | 8.08 | 0.03253 |
| pos_249 | DG(19:1/18:2) | up | 1.1246 | 1.2239 | 2.31E-09 | 5.65E-09 | 7.959 | 0.107 | 6.503 | 0.1444 |
| pos_261 | DG(16:0/22:6) | up | 1.2532 | 1.2498 | 4.97E-14 | 5.46E-13 | 8.95 | 0.04655 | 7.161 | 0.0583 |
| pos_262 | DG(20:4e/18:2) | down | 1.3255 | 0.7472 | 1.84E-12 | 1.09E-11 | 5.925 | 0.114 | 7.93 | 0.03793 |
| pos_270 | DG(18:1/22:6) | up | 1.3299 | 1.2702 | 1.08E-09 | 2.79E-09 | 9.556 | 0.01567 | 7.523 | 0.2315 |
| pos_272 | DG(18:3/22:6) | down | 2.3821 | 0.1636 | 1.57E-06 | 2.37E-06 | 1.324 | 1.652 | 8.092 | 0.1053 |
| pos_284 | DG(35:0/18:2) | down | 1.0762 | 0.8235 | 3.90E-13 | 2.96E-12 | 6.163 | 0.03087 | 7.484 | 0.06027 |
| pos_289 | DG(36:0/18:3) | down | 1.1553 | 0.797 | 6.10E-09 | 1.37E-08 | 6.051 | 0.1796 | 7.592 | 0.109 |
| pos_298 | DGDG(8:0e/10:0) | down | 1.4251 | 0.7201 | 3.31E-11 | 1.34E-10 | 5.974 | 0.169 | 8.296 | 0.0782 |
| pos_299 | DGDG(8:0e/10:1) | down | 1.1918 | 0.8075 | 2.18E-09 | 5.39E-09 | 6.86 | 0.1588 | 8.495 | 0.1226 |
| pos_301 | DGDG(8:0e/10:3) | down | 1.179 | 0.8139 | 2.91E-13 | 2.31E-12 | 6.933 | 0.05822 | 8.518 | 0.05326 |
| pos_302 | DGDG(8:0e/12:3) | down | 1.4296 | 0.7189 | 1.68E-11 | 7.38E-11 | 5.971 | 0.1669 | 8.306 | 0.05189 |
| pos_303 | DGDG(8:0e/12:4) | down | 1.1505 | 0.8311 | 2.93E-12 | 1.59E-11 | 7.436 | 0.07973 | 8.947 | 0.05138 |
| pos_305 | DGDG(8:1e/12:5) | down | 1.179 | 0.8139 | 2.91E-13 | 2.31E-12 | 6.933 | 0.05822 | 8.518 | 0.05326 |
| pos_328 | Hex1Cer(d12:0/18:1) | up | 1.0607 | 1.2211 | 7.89E-06 | 1.10E-05 | 7.56 | 0.06445 | 6.191 | 0.3953 |
| pos_334 | Hex1Cer(d18:1/20:1) | up | 1.3151 | 1.3583 | 2.35E-08 | 4.67E-08 | 7.613 | 0.05843 | 5.605 | 0.3091 |
| pos_344 | Hex1Cer(d18:2/24:1) | up | 1.3631 | 1.2877 | 3.71E-14 | 4.24E-13 | 9.47 | 0.0191 | 7.354 | 0.08355 |
| pos_345 | Hex1Cer(d18:2/24:2) | up | 2.017 | 3.4671 | 2.10E-05 | 2.81E-05 | 7.059 | 0.07625 | 2.036 | 1.643 |
| pos_357 | Hex1Cer(t18:1/24:1) | up | 1.3631 | 1.2877 | 3.71E-14 | 4.24E-13 | 9.47 | 0.0191 | 7.354 | 0.08355 |
| pos_360 | Hex1SPH(t18:1) | down | 1.1599 | 0.7958 | 2.24E-08 | 4.48E-08 | 6.078 | 0.2312 | 7.638 | 0.07589 |
| pos_377 | LPC(18:2e) | down | 1.2909 | 0.734 | 1.09E-06 | 1.68E-06 | 5.468 | 0.4525 | 7.45 | 0.1108 |
| pos_380 | LPC(20:0) | down | 1.0628 | 0.826 | 1.43E-08 | 2.96E-08 | 6.215 | 0.1829 | 7.524 | 0.0672 |
| pos_381 | LPC(20:1) | down | 1.1908 | 0.7958 | 9.41E-08 | 1.71E-07 | 6.457 | 0.2495 | 8.114 | 0.1667 |
| pos_383 | LPC(20:3) | down | 1.2742 | 0.7959 | 4.17E-16 | 9.62E-15 | 7.207 | 0.02936 | 9.055 | 0.03765 |
| pos_387 | LPC(22:4) | down | 1.3065 | 0.7609 | 7.19E-12 | 3.58E-11 | 6.201 | 0.1001 | 8.15 | 0.08903 |
| pos_397 | LPG(18:1) | down | 1.0556 | 0.8228 | 5.09E-09 | 1.16E-08 | 5.972 | 0.168 | 7.258 | 0.03723 |
| pos_399 | LPMe(16:0) | down | 1.68 | 0.6012 | 1.05E-13 | 1.02E-12 | 4.85 | 0.1323 | 8.067 | 0.05835 |
| pos_400 | LPMe(18:0) | down | 1.6129 | 0.6325 | 9.52E-17 | 2.87E-15 | 5.096 | 0.03087 | 8.057 | 0.0583 |
| pos_401 | LPMe(18:1) | down | 1.5265 | 0.6568 | 1.54E-16 | 4.30E-15 | 5.076 | 0.03087 | 7.728 | 0.05376 |
| pos_405 | MG(16:0) | down | 1.1611 | 0.8077 | 1.97E-12 | 1.15E-11 | 6.458 | 0.0714 | 7.996 | 0.05929 |
| pos_406 | MG(16:1) | down | 1.0981 | 0.8215 | 5.33E-10 | 1.48E-09 | 6.373 | 0.1444 | 7.758 | 0.02803 |
| pos_407 | MG(18:1) | down | 1.0996 | 0.8123 | 8.53E-09 | 1.85E-08 | 6.054 | 0.1862 | 7.453 | 0.06541 |
| pos_412 | MG(20:4) | up | 1.7306 | 2.2077 | 0.001916 | 0.002204 | 7.813 | 0.08764 | 3.539 | 2.509 |
| pos_415 | MG(22:1) | down | 1.3504 | 0.7548 | 9.63E-13 | 6.29E-12 | 6.402 | 0.1052 | 8.482 | 0.05067 |
| pos_422 | MGDG(18:1/18:1) | down | 1.1162 | 0.8281 | 4.22E-10 | 1.21E-09 | 6.882 | 0.1175 | 8.311 | 0.09052 |
| pos_444 | PC(4:0/14:0) | down | 1.2513 | 0.785 | 2.11E-12 | 1.21E-11 | 6.521 | 0.1046 | 8.307 | 0.02878 |
| pos_450 | PC(11:0/16:1) | up | 1.2624 | 1.3566 | 2.62E-09 | 6.32E-09 | 6.984 | 0.1017 | 5.148 | 0.2058 |
| pos_451 | PC(9:0/18:2) | up | 1.2544 | 1.3496 | 6.99E-07 | 1.11E-06 | 7.196 | 0.09132 | 5.332 | 0.4074 |
| pos_452 | PC(16:0/12:0) | up | 1.0541 | 1.2072 | 5.97E-09 | 1.35E-08 | 7.481 | 0.02884 | 6.197 | 0.1722 |
| pos_460 | PC(18:1/14:0) | down | 1.455 | 0.7342 | 4.60E-10 | 1.31E-09 | 6.71 | 0.2539 | 9.139 | 0.01406 |
| pos_464 | PC(18:2/14:1) | down | 1.9003 | 0.5666 | 9.24E-13 | 6.11E-12 | 5.385 | 0.2257 | 9.504 | 0.04589 |
| pos_500 | PE(6:0/12:1) | down | 1.131 | 0.805 | 6.93E-10 | 1.88E-09 | 6.067 | 0.153 | 7.537 | 0.04824 |
| pos_511 | PE(17:1/18:1) | down | 1.7536 | 0.6282 | 2.22E-10 | 6.99E-10 | 5.954 | 0.329 | 9.478 | 0.09559 |
| pos_512 | PE(17:1/18:2) | down | 1.8746 | 0.5782 | 2.64E-14 | 3.17E-13 | 5.485 | 0.1523 | 9.487 | 0.03687 |
| pos_513 | PE(17:1/18:3) | down | 1.7301 | 0.5909 | 1.23E-12 | 7.68E-12 | 4.931 | 0.186 | 8.345 | 0.06343 |
| pos_516 | PE(18:1/18:2) | down | 1.3393 | 0.7381 | 6.52E-09 | 1.45E-08 | 5.839 | 0.2705 | 7.911 | 0.08816 |
| pos_519 | PE(18:3e/18:1) | down | 1.51 | 0.7137 | 1.24E-09 | 3.17E-09 | 6.535 | 0.2929 | 9.157 | 0.0796 |
| pos_521 | PE(18:4/18:2) | down | 1.5499 | 0.6442 | 2.48E-10 | 7.60E-10 | 4.99 | 0.2685 | 7.746 | 0.03583 |
| pos_534 | PG(16:0/18:3) | down | 1.5686 | 0.6477 | 5.22E-13 | 3.77E-12 | 5.158 | 0.0947 | 7.963 | 0.1139 |
| pos_535 | PG(18:4/16:0) | down | 1.3598 | 0.7141 | 6.53E-07 | 1.04E-06 | 5.462 | 0.4345 | 7.649 | 0.2192 |
| pos_539 | PG(18:0/20:0) | down | 1.1225 | 0.8131 | 1.70E-06 | 2.56E-06 | 6.539 | 0.2603 | 8.042 | 0.2645 |
| pos_545 | PG(28:1/16:0) | up | 1.1551 | 1.2525 | 1.05E-08 | 2.25E-08 | 7.664 | 0.1038 | 6.119 | 0.1969 |
| pos_549 | PG(28:0/18:3) | up | 1.2952 | 1.3297 | 4.61E-13 | 3.43E-12 | 7.711 | 0.07877 | 5.799 | 0.06115 |
| pos_550 | PG(28:1/18:2) | up | 1.334 | 1.3535 | 3.20E-10 | 9.45E-10 | 7.814 | 0.05896 | 5.773 | 0.1973 |
| pos_558 | PI(31:0/17:1) | down | 1.2056 | 0.8042 | 6.08E-09 | 1.37E-08 | 6.894 | 0.1361 | 8.572 | 0.1838 |
| pos_560 | PMe(16:0/18:1) | down | 1.6028 | 0.6631 | 3.77E-17 | 1.39E-15 | 5.752 | 0.03087 | 8.675 | 0.05071 |
| pos_561 | PMe(16:0/18:2) | down | 1.34 | 0.7509 | 9.56E-09 | 2.07E-08 | 6.261 | 0.2845 | 8.338 | 0.08376 |
| pos_564 | PMe(18:1/18:3) | down | 1.6028 | 0.6631 | 3.77E-17 | 1.39E-15 | 5.752 | 0.03087 | 8.675 | 0.05071 |
| pos_565 | PMe(18:3/18:2) | down | 1.3373 | 0.7519 | 1.01E-08 | 2.17E-08 | 6.269 | 0.2851 | 8.338 | 0.08376 |
| pos_566 | PMe(18:3/18:3) | down | 1.333 | 0.7201 | 6.18E-07 | 9.93E-07 | 5.406 | 0.4549 | 7.507 | 0.09427 |
| pos_567 | PMe(17:1/20:5) | down | 1.5499 | 0.6619 | 1.42E-10 | 4.69E-10 | 5.385 | 0.2457 | 8.136 | 0.0706 |
| pos_577 | SM(d18:1/24:6) | up | 1.376 | 1.3543 | 3.15E-09 | 7.45E-09 | 8.34 | 0.04143 | 6.158 | 0.2749 |
| pos_593 | SiE(18:1) | down | 1.4532 | 0.7059 | 1.97E-15 | 3.41E-14 | 5.769 | 0.05124 | 8.173 | 0.05136 |
| pos_594 | SiE(18:2) | down | 1.3573 | 0.7628 | 8.06E-12 | 3.93E-11 | 6.769 | 0.1407 | 8.874 | 0.04024 |
| pos_597 | SiE(20:5) | down | 1.5682 | 0.6811 | 4.15E-18 | 2.71E-16 | 5.975 | 0.03087 | 8.773 | 0.03352 |
| pos_600 | StE(18:3) | down | 1.3359 | 0.7386 | 8.07E-08 | 1.49E-07 | 5.887 | 0.3228 | 7.971 | 0.1836 |
| pos_601 | StE(19:2) | down | 1.3474 | 0.7441 | 7.30E-14 | 7.47E-13 | 6.012 | 0.08677 | 8.08 | 0.02252 |
| pos_602 | StE(20:5) | down | 1.4808 | 0.7134 | 1.59E-15 | 2.79E-14 | 6.214 | 0.03087 | 8.71 | 0.06696 |
| pos_603 | StE(37:6) | up | 1.2796 | 1.3007 | 4.83E-11 | 1.83E-10 | 8.102 | 0.06354 | 6.229 | 0.1425 |
| pos_604 | TG(6:0/6:0/9:0) | down | 1.2176 | 0.7802 | 3.79E-11 | 1.51E-10 | 6.02 | 0.137 | 7.716 | 0.01474 |
| pos_606 | TG(4:0/6:0/11:2) | down | 1.3411 | 0.7564 | 9.73E-11 | 3.37E-10 | 6.393 | 0.1353 | 8.452 | 0.125 |
| pos_612 | TG(6:0/8:0/11:1) | up | 1.0614 | 1.2247 | 4.28E-06 | 6.11E-06 | 7.412 | 0.2125 | 6.052 | 0.3045 |
| pos_613 | TG(8:0/8:0/10:0) | up | 1.2055 | 1.2725 | 4.65E-09 | 1.07E-08 | 7.826 | 0.025 | 6.15 | 0.2209 |
| pos_618 | TG(11:0/6:0/10:1) | up | 1.0864 | 1.2043 | 7.94E-09 | 1.74E-08 | 8.041 | 0.02072 | 6.677 | 0.19 |
| pos_620 | TG(11:0/6:0/10:2) | up | 1.3121 | 1.327 | 6.53E-12 | 3.31E-11 | 7.978 | 0.1191 | 6.012 | 0.06115 |
| pos_622 | TG(6:0/10:0/11:2) | up | 1.2405 | 1.3002 | 1.03E-13 | 1.01E-12 | 7.596 | 0.04954 | 5.842 | 0.06115 |
| pos_628 | TG(6:0/9:0/12:3) | up | 1.1998 | 1.2635 | 1.41E-13 | 1.28E-12 | 7.863 | 0.0451 | 6.223 | 0.06115 |
| pos_632 | TG(14:0e/6:0/9:0) | down | 1.5538 | 0.6511 | 5.34E-14 | 5.76E-13 | 5.133 | 0.108 | 7.883 | 0.04098 |
| pos_638 | TG(12:1e/8:0/10:3) | up | 1.2081 | 1.2714 | 2.48E-07 | 4.25E-07 | 8.034 | 0.1164 | 6.319 | 0.3236 |
| pos_639 | TG(6:0/12:2/12:3) | up | 1.3691 | 1.3734 | 2.96E-07 | 5.02E-07 | 8.106 | 0.03232 | 5.902 | 0.4495 |
| pos_641 | TG(6:0/12:3/12:3) | up | 1.4415 | 1.4471 | 5.01E-13 | 3.66E-12 | 7.671 | 0.1086 | 5.301 | 0.06115 |
| pos_642 | TG(15:0/6:0/10:0) | down | 1.9771 | 0.3287 | 0.0001515 | 0.0001874 | 2.468 | 2.091 | 7.508 | 0.0879 |
| pos_644 | TG(16:0/6:0/9:0) | down | 1.1776 | 0.8233 | 7.98E-16 | 1.59E-14 | 7.359 | 0.04213 | 8.938 | 0.01087 |
| pos_645 | TG(14:0e/8:0/9:0) | down | 2.2637 | 0.1998 | 1.47E-05 | 1.99E-05 | 1.57 | 1.964 | 7.857 | 0.2045 |
| pos_649 | TG(12:0e/9:0/10:1) | down | 1.5313 | 0.6641 | 2.10E-11 | 8.90E-11 | 5.298 | 0.2008 | 7.978 | 0.04249 |
| pos_650 | TG(14:1e/8:0/9:0) | down | 1.3365 | 0.7787 | 5.39E-14 | 5.76E-13 | 7.162 | 0.08061 | 9.197 | 0.02866 |
| pos_652 | TG(14:1e/6:0/11:0) | down | 1.4684 | 0.7147 | 1.37E-10 | 4.52E-10 | 6.188 | 0.2205 | 8.658 | 0.06024 |
| pos_660 | TG(12:0e/10:0/10:2) | up | 1.2797 | 1.3161 | 5.59E-11 | 2.08E-10 | 7.802 | 0.09267 | 5.928 | 0.1285 |
| pos_662 | TG(6:0/12:2/14:4) | up | 1.6132 | 1.5836 | 1.29E-10 | 4.29E-10 | 8.089 | 0.05466 | 5.108 | 0.2687 |
| pos_663 | TG(8:0/10:2/14:4) | up | 1.3596 | 1.3839 | 1.45E-11 | 6.55E-11 | 7.617 | 0.1004 | 5.504 | 0.1192 |
| pos_665 | TG(15:0/9:0/9:0) | down | 1.2609 | 0.79 | 7.70E-16 | 1.59E-14 | 6.807 | 0.03546 | 8.617 | 0.03484 |
| pos_666 | TG(15:0/6:0/12:0) | down | 1.2246 | 0.7996 | 1.27E-14 | 1.69E-13 | 6.815 | 0.03753 | 8.523 | 0.04951 |
| pos_669 | TG(15:0/6:0/12:1) | down | 1.2643 | 0.7936 | 2.75E-12 | 1.51E-11 | 7.012 | 0.09173 | 8.836 | 0.0674 |
| pos_672 | TG(14:0e/9:0/10:1) | down | 1.4568 | 0.7572 | 1.35E-16 | 3.86E-15 | 7.532 | 0.05441 | 9.947 | 0.01203 |
| pos_673 | TG(12:1e/6:0/15:0) | down | 1.5869 | 0.6566 | 3.34E-17 | 1.38E-15 | 5.477 | 0.03087 | 8.342 | 0.0485 |
| pos_674 | TG(9:0/12:1/12:1) | down | 1.187 | 0.8068 | 2.52E-10 | 7.71E-10 | 6.749 | 0.1103 | 8.365 | 0.1148 |
| pos_676 | TG(16:0/6:0/11:2) | down | 1.2857 | 0.7537 | 4.25E-08 | 8.12E-08 | 5.888 | 0.2932 | 7.812 | 0.1299 |
| pos_679 | TG(14:0e/9:0/10:2) | down | 1.1857 | 0.7836 | 2.26E-09 | 5.54E-09 | 5.867 | 0.1576 | 7.487 | 0.1222 |
| pos_687 | TG(14:0e/8:0/11:3) | down | 1.2383 | 0.8049 | 2.25E-12 | 1.27E-11 | 7.218 | 0.05056 | 8.968 | 0.09429 |
| pos_703 | TG(14:1e/9:0/10:4) | down | 1.2602 | 0.7824 | 4.62E-06 | 6.58E-06 | 6.897 | 0.5266 | 8.815 | 0.04446 |
| pos_706 | TG(16:0/9:0/9:0) | up | 1.1533 | 1.2369 | 2.47E-09 | 6.01E-09 | 8.003 | 0.09638 | 6.47 | 0.1642 |
| pos_709 | TG(6:0/10:0/18:1) | down | 1.1288 | 0.8058 | 6.23E-07 | 1.00E-06 | 6.257 | 0.3324 | 7.765 | 0.03068 |
| pos_711 | TG(12:1e/11:0/11:0) | down | 1.0056 | 0.817 | 3.07E-05 | 4.04E-05 | 5.615 | 0.3987 | 6.873 | 0.1625 |
| pos_712 | TG(12:1e/6:0/16:0) | down | 1.4738 | 0.7166 | 6.88E-13 | 4.73E-12 | 6.264 | 0.1264 | 8.741 | 0.04589 |
| pos_722 | TG(14:0e/10:0/10:2) | up | 1.5803 | 1.6045 | 6.46E-05 | 8.23E-05 | 8.364 | 0.08293 | 5.213 | 1.175 |
| pos_728 | TG(16:1e/6:0/12:2) | down | 1.3379 | 0.7775 | 3.48E-10 | 1.02E-09 | 7.177 | 0.04966 | 9.231 | 0.203 |
| pos_732 | TG(14:0e/10:2/10:2) | down | 1.1818 | 0.8078 | 9.92E-10 | 2.60E-09 | 6.751 | 0.1788 | 8.357 | 0.03272 |
| pos_739 | TG(14:0e/9:0/12:1) | down | 1.4919 | 0.6964 | 3.11E-18 | 2.39E-16 | 5.808 | 0.03087 | 8.34 | 0.02556 |
| pos_743 | TG(16:0e/9:0/10:2) | down | 1.4918 | 0.6935 | 2.43E-18 | 2.04E-16 | 5.729 | 0.03087 | 8.261 | 0.02398 |
| pos_745 | TG(14:1e/9:0/12:1) | down | 1.3966 | 0.7239 | 2.50E-09 | 6.05E-09 | 5.892 | 0.218 | 8.139 | 0.1748 |
| pos_747 | TG(6:0/11:2/18:1) | down | 1.1299 | 0.8314 | 6.33E-16 | 1.38E-14 | 7.164 | 0.03381 | 8.617 | 0.01972 |
| pos_748 | TG(16:1e/9:0/10:2) | down | 1.1575 | 0.7933 | 2.20E-05 | 2.93E-05 | 6.354 | 0.4318 | 8.01 | 0.3321 |
| pos_749 | TG(6:0/11:1/18:3) | down | 1.3559 | 0.789 | 3.40E-17 | 1.38E-15 | 7.822 | 0.03885 | 9.914 | 0.0161 |
| pos_750 | TG(6:0/11:2/18:3) | down | 1.3372 | 0.7787 | 2.38E-10 | 7.37E-10 | 7.218 | 0.1834 | 9.269 | 0.08169 |
| pos_752 | TG(18:4/6:0/11:1) | down | 1.1947 | 0.8325 | 6.68E-17 | 2.18E-15 | 8.069 | 0.02697 | 9.693 | 0.0222 |
| pos_756 | TG(6:0/11:4/18:3) | up | 1.0874 | 1.2354 | 1.12E-06 | 1.73E-06 | 7.38 | 0.1727 | 5.974 | 0.2832 |
| pos_760 | TG(18:0/9:0/9:0) | up | 1.1694 | 1.2605 | 1.50E-07 | 2.63E-07 | 7.751 | 0.1118 | 6.149 | 0.2833 |
| pos_770 | TG(16:0/10:1/10:1) | up | 1.287 | 1.2661 | 1.09E-11 | 5.09E-11 | 9.001 | 0.04605 | 7.109 | 0.1276 |
| pos_776 | TG(9:0/9:0/18:3) | up | 1.224 | 1.2695 | 1.90E-06 | 2.85E-06 | 8.437 | 0.2143 | 6.646 | 0.3926 |
| pos_790 | TG(6:0/12:1/18:3) | up | 1.346 | 1.3322 | 5.59E-08 | 1.05E-07 | 8.465 | 0.2117 | 6.354 | 0.2936 |
| pos_791 | TG(6:0/12:3/18:1) | up | 1.2575 | 1.2773 | 3.00E-10 | 8.95E-10 | 8.351 | 0.1119 | 6.538 | 0.1432 |
| pos_792 | TG(8:0/10:3/18:1) | up | 1.2342 | 1.3099 | 4.64E-07 | 7.67E-07 | 7.604 | 0.3239 | 5.805 | 0.2096 |
| pos_806 | TG(14:1e/11:2/11:3) | up | 1.2091 | 1.2459 | 5.04E-10 | 1.42E-09 | 8.507 | 0.03792 | 6.828 | 0.1733 |
| pos_815 | TG(6:0/13:0/18:2) | down | 1.3125 | 0.7647 | 1.14E-08 | 2.41E-08 | 6.476 | 0.288 | 8.469 | 0.03249 |
| pos_817 | TG(6:0/13:0/18:3) | down | 1.6844 | 0.5993 | 1.02E-13 | 1.01E-12 | 4.835 | 0.03087 | 8.068 | 0.1416 |
| pos_830 | TG(20:0/6:0/12:0) | up | 1.2155 | 1.2843 | 2.71E-09 | 6.49E-09 | 7.688 | 0.02134 | 5.986 | 0.2124 |
| pos_831 | TG(6:0/14:0/18:1) | up | 1.1317 | 1.2461 | 4.57E-11 | 1.76E-10 | 7.417 | 0.08598 | 5.952 | 0.08574 |
| pos_832 | TG(20:1/6:0/12:0) | up | 1.2093 | 1.279 | 1.74E-13 | 1.51E-12 | 7.641 | 0.05012 | 5.974 | 0.06087 |
| pos_833 | TG(18:0/10:0/10:1) | up | 1.2796 | 1.3093 | 1.97E-14 | 2.46E-13 | 7.894 | 0.03584 | 6.029 | 0.06115 |
| pos_837 | TG(9:0/11:1/18:1) | up | 1.2597 | 1.2534 | 2.24E-12 | 1.27E-11 | 8.952 | 0.0282 | 7.142 | 0.107 |
| pos_838 | TG(18:0/10:1/10:1) | up | 1.5005 | 1.4622 | 2.28E-08 | 4.54E-08 | 8.267 | 0.04058 | 5.654 | 0.406 |
| pos_841 | TG(18:1/10:1/10:1) | up | 1.2304 | 1.3055 | 5.70E-14 | 5.99E-13 | 7.367 | 0.03974 | 5.643 | 0.06115 |
| pos_842 | TG(10:0/10:1/18:2) | up | 1.3358 | 1.3238 | 3.29E-09 | 7.75E-09 | 8.414 | 0.02973 | 6.356 | 0.2616 |
| pos_845 | TG(14:0e/6:0/18:3) | up | 1.1539 | 1.2415 | 2.51E-08 | 4.96E-08 | 7.948 | 0.1278 | 6.402 | 0.2076 |
| pos_848 | TG(18:2/10:1/10:1) | up | 1.3564 | 1.3104 | 9.84E-11 | 3.40E-10 | 8.894 | 0.05748 | 6.787 | 0.1796 |
| pos_854 | TG(8:0/12:2/18:3) | up | 1.1227 | 1.2485 | 9.79E-09 | 2.11E-08 | 7.325 | 0.07443 | 5.867 | 0.195 |
| pos_860 | TG(11:0/10:1/18:1) | down | 1.4527 | 0.7064 | 1.10E-18 | 1.30E-16 | 5.776 | 0.02977 | 8.177 | 0.01694 |
| pos_866 | TG(16:0/11:3/12:2) | down | 1.3373 | 0.7519 | 1.01E-08 | 2.17E-08 | 6.269 | 0.2851 | 8.338 | 0.08376 |
| pos_867 | TG(18:2/10:1/11:2) | down | 1.714 | 0.6115 | 1.51E-13 | 1.34E-12 | 5.269 | 0.1363 | 8.616 | 0.07587 |
| pos_868 | TG(10:0/11:3/18:2) | down | 1.5159 | 0.6717 | 8.00E-09 | 1.75E-08 | 5.434 | 0.3509 | 8.09 | 0.1245 |
| pos_869 | TG(18:2/10:1/11:3) | down | 1.1086 | 0.8151 | 5.68E-08 | 1.07E-07 | 6.314 | 0.2411 | 7.746 | 0.04816 |
| pos_871 | TG(18:3/10:1/11:2) | up | 1.3902 | 1.3897 | 1.30E-10 | 4.31E-10 | 7.895 | 0.03878 | 5.681 | 0.2001 |
| pos_875 | TG(16:0/8:0/16:0) | up | 1.3254 | 1.3704 | 3.01E-07 | 5.08E-07 | 7.648 | 0.05192 | 5.581 | 0.4201 |
| pos_876 | TG(6:0/12:0/22:1) | up | 1.0344 | 1.2223 | 7.80E-06 | 1.09E-05 | 7.148 | 0.1128 | 5.848 | 0.3628 |
| pos_880 | TG(18:2/10:2/12:2) | down | 1.6235 | 0.6419 | 3.73E-17 | 1.39E-15 | 5.375 | 0.03087 | 8.374 | 0.05243 |
| pos_881 | TG(18:2/10:3/12:1) | up | 2.5679 | 12.3978 | 5.70E-21 | 2.10E-18 | 8.159 | 0.04129 | 0.6581 | 0.04784 |
| pos_882 | TG(4:0/18:3/18:3) | up | 1.5415 | 1.4543 | 2.00E-14 | 2.47E-13 | 8.665 | 0.02371 | 5.958 | 0.1002 |
| pos_883 | TG(18:2/11:2/11:2) | up | 1.5782 | 1.4751 | 2.33E-14 | 2.82E-13 | 8.808 | 0.01261 | 5.971 | 0.1089 |
| pos_884 | TG(18:3/11:2/11:2) | down | 1.6536 | 0.6312 | 1.39E-15 | 2.52E-14 | 5.326 | 0.07323 | 8.438 | 0.05356 |
| pos_885 | TG(18:2/11:1/11:4) | up | 2.3682 | 5.1292 | 2.40E-13 | 1.96E-12 | 7.94 | 0.3065 | 1.548 | 0.05944 |
| pos_886 | TG(18:2/11:2/11:3) | up | 1.4752 | 1.4046 | 9.12E-15 | 1.31E-13 | 8.602 | 0.05432 | 6.124 | 0.0682 |
| pos_892 | TG(16:0/11:3/14:3) | up | 1.2675 | 1.3064 | 1.94E-14 | 2.46E-13 | 7.803 | 0.03294 | 5.973 | 0.06115 |
| pos_897 | TG(12:1e/11:2/18:4) | up | 1.2751 | 1.2802 | 7.07E-15 | 1.05E-13 | 8.461 | 0.01712 | 6.609 | 0.06115 |
| pos_898 | TG(18:2/11:2/12:4) | down | 1.5416 | 0.6601 | 1.71E-19 | 2.51E-17 | 5.25 | 0.02846 | 7.953 | 0.01465 |
| pos_900 | TG(12:1e/11:3/18:4) | up | 1.3227 | 1.3252 | 2.57E-12 | 1.43E-11 | 8.134 | 0.1076 | 6.138 | 0.06115 |
| pos_901 | TG(16:0/8:0/18:1) | up | 1.1605 | 1.2386 | 1.23E-08 | 2.60E-08 | 8.089 | 0.04734 | 6.531 | 0.2235 |
| pos_902 | TG(18:4/10:3/14:3) | down | 2.1381 | 0.2472 | 0.0002304 | 0.0002819 | 1.959 | 2.613 | 7.926 | 0.05903 |
| pos_903 | TG(18:3/12:3/12:4) | down | 1.6536 | 0.6312 | 1.39E-15 | 2.52E-14 | 5.326 | 0.07323 | 8.438 | 0.05356 |
| pos_905 | TG(16:0/10:2/16:0) | up | 1.4704 | 1.4315 | 4.45E-13 | 3.34E-12 | 8.177 | 0.1065 | 5.712 | 0.07112 |
| pos_909 | TG(12:0e/12:4/18:1) | down | 1.4353 | 0.5516 | 0.01226 | 0.01351 | 4.15 | 2.701 | 7.524 | 0.215 |
| pos_910 | TG(12:1e/10:3/20:2) | down | 1.4054 | 0.6767 | 3.02E-06 | 4.38E-06 | 4.968 | 0.187 | 7.341 | 0.595 |
| pos_912 | TG(18:2/12:2/12:3) | down | 1.6658 | 0.6299 | 1.65E-17 | 8.45E-16 | 5.372 | 0.03087 | 8.529 | 0.05034 |
| pos_913 | TG(18:1/12:3/12:3) | up | 2.549 | 8.4151 | 2.30E-20 | 5.30E-18 | 8.389 | 0.04577 | 0.9969 | 0.05506 |
| pos_914 | TG(18:2/10:1/14:4) | up | 2.604 | 8.091 | 1.93E-18 | 1.94E-16 | 8.803 | 0.1019 | 1.088 | 0.05621 |
| pos_915 | TG(18:1/10:2/14:4) | up | 1.5544 | 1.4517 | 2.92E-09 | 6.96E-09 | 8.947 | 0.1477 | 6.163 | 0.3193 |
| pos_918 | TG(18:1/10:3/14:4) | down | 1.6854 | 0.6319 | 4.46E-18 | 2.76E-16 | 5.548 | 0.03087 | 8.78 | 0.0431 |
| pos_921 | TG(18:4/6:0/18:4) | up | 1.5723 | 1.4741 | 1.76E-13 | 1.51E-12 | 8.762 | 0.01912 | 5.944 | 0.132 |
| pos_922 | TG(18:3/12:3/12:3) | down | 1.6258 | 0.6615 | 2.77E-10 | 8.32E-10 | 5.922 | 0.2931 | 8.953 | 0.06999 |
| pos_928 | TG(16:0/11:3/16:0) | up | 1.1326 | 1.2654 | 1.01E-07 | 1.84E-07 | 7.152 | 0.1739 | 5.652 | 0.2114 |
| pos_929 | TG(16:0/11:4/16:0) | up | 1.7835 | 1.7298 | 7.31E-18 | 4.30E-16 | 8.578 | 0.01234 | 4.959 | 0.06115 |
| pos_930 | TG(18:1/11:3/14:3) | up | 1.3662 | 1.3725 | 1.27E-10 | 4.26E-10 | 7.877 | 0.01737 | 5.739 | 0.1957 |
| pos_937 | TG(6:0/18:1/20:2) | up | 1.8514 | 2.4957 | 0.001686 | 0.001953 | 8.101 | 0.08192 | 3.246 | 2.796 |
| pos_939 | TG(8:0/18:2/18:2) | up | 1.3226 | 1.3345 | 1.21E-14 | 1.63E-13 | 7.947 | 0.03821 | 5.955 | 0.06115 |
| pos_942 | TG(6:0/18:1/20:4) | up | 1.3026 | 1.3352 | 2.56E-12 | 1.43E-11 | 7.712 | 0.04269 | 5.776 | 0.1121 |
| pos_943 | TG(16:0/10:4/18:2) | up | 1.3496 | 1.3575 | 1.23E-08 | 2.60E-08 | 8.008 | 0.1182 | 5.899 | 0.2855 |
| pos_950 | TG(18:1/10:1/17:1) | up | 1.144 | 1.2537 | 7.54E-09 | 1.66E-08 | 7.476 | 0.09848 | 5.963 | 0.1864 |
| pos_956 | TG(16:0/11:4/18:1) | up | 1.275 | 1.2997 | 8.04E-14 | 8.15E-13 | 8.032 | 0.05322 | 6.18 | 0.06115 |
| pos_957 | TG(18:4/9:0/18:1) | up | 1.6757 | 1.554 | 2.15E-15 | 3.60E-14 | 8.968 | 0.0221 | 5.771 | 0.09476 |
| pos_962 | TG(18:4/9:0/18:4) | up | 1.4861 | 1.4367 | 1.70E-11 | 7.43E-11 | 8.307 | 0.1542 | 5.782 | 0.1097 |
| pos_968 | TG(22:6/12:4/12:4) | down | 1.4367 | 0.6961 | 7.74E-16 | 1.59E-14 | 5.38 | 0.04865 | 7.729 | 0.04247 |
| pos_975 | TG(18:1/10:1/18:2) | up | 1.4288 | 1.4007 | 6.77E-10 | 1.84E-09 | 8.197 | 0.2478 | 5.852 | 0.06115 |
| pos_976 | TG(10:0/18:2/18:2) | up | 1.0979 | 1.2122 | 2.35E-10 | 7.28E-10 | 7.894 | 0.03185 | 6.512 | 0.1313 |
| pos_978 | TG(16:0/10:0/20:5) | up | 1.4053 | 1.4014 | 1.54E-08 | 3.17E-08 | 7.988 | 0.3376 | 5.7 | 0.06115 |
| pos_982 | TG(18:1/10:3/18:3) | up | 1.1586 | 1.245 | 4.13E-11 | 1.61E-10 | 7.8 | 0.11 | 6.265 | 0.06115 |
| pos_983 | TG(18:3/10:2/18:2) | up | 1.2213 | 1.2772 | 2.21E-07 | 3.84E-07 | 8.063 | 0.3103 | 6.313 | 0.1552 |
| pos_984 | TG(18:2/10:3/18:2) | up | 1.2072 | 1.2824 | 1.63E-08 | 3.32E-08 | 7.669 | 0.07333 | 5.98 | 0.244 |
| pos_988 | TG(15:0/16:0/16:0) | down | 1.619 | 0.6514 | 2.16E-16 | 5.76E-15 | 5.575 | 0.03087 | 8.558 | 0.06521 |
| pos_991 | TG(15:0/14:0/18:1) | down | 1.5881 | 0.6402 | 2.59E-17 | 1.27E-15 | 5.107 | 0.03087 | 7.977 | 0.04689 |
| pos_995 | TG(16:0/13:0/18:2) | up | 1.1697 | 1.2287 | 5.35E-11 | 2.00E-10 | 8.407 | 0.02398 | 6.842 | 0.1296 |
| pos_1006 | TG(18:3/11:2/18:2) | up | 1.3443 | 1.3629 | 2.64E-09 | 6.34E-09 | 7.819 | 0.2241 | 5.737 | 0.1325 |
| pos_1007 | TG(18:3e/11:1/18:3) | up | 1.4658 | 1.4365 | 1.93E-10 | 6.17E-10 | 8.102 | 0.1237 | 5.64 | 0.2009 |
| pos_1012 | TG(16:0/14:0/18:3) | up | 1.457 | 1.4071 | 1.29E-12 | 7.89E-12 | 8.372 | 0.01142 | 5.95 | 0.1396 |
| pos_1016 | TG(18:1/12:1/18:3) | up | 1.0781 | 1.2218 | 1.33E-06 | 2.02E-06 | 7.617 | 0.1482 | 6.234 | 0.2976 |
| pos_1018 | TG(18:1/10:1/20:4) | up | 1.4746 | 1.4463 | 1.54E-10 | 5.04E-10 | 8.076 | 0.2253 | 5.584 | 0.06115 |
| pos_1019 | TG(18:1/12:3/18:2) | up | 1.551 | 1.4835 | 4.59E-09 | 1.05E-08 | 8.514 | 0.2025 | 5.739 | 0.3066 |
| pos_1020 | TG(18:2/12:2/18:2) | up | 1.2407 | 1.2821 | 2.76E-10 | 8.32E-10 | 8.022 | 0.09626 | 6.257 | 0.1466 |
| pos_1021 | TG(18:3/12:2/18:2) | up | 1.4318 | 1.4021 | 4.39E-10 | 1.26E-09 | 8.205 | 0.2373 | 5.852 | 0.06115 |
| pos_1027 | TG(15:0/16:0/18:1) | down | 1.4992 | 0.7293 | 2.97E-17 | 1.34E-15 | 6.89 | 0.03087 | 9.448 | 0.04023 |
| pos_1032 | TG(15:0/16:0/18:2) | down | 1.6679 | 0.6589 | 1.39E-11 | 6.32E-11 | 6.139 | 0.2188 | 9.317 | 0.08121 |
| pos_1038 | TG(18:1/13:0/18:2) | down | 1.2149 | 0.8285 | 2.59E-16 | 6.34E-15 | 8.115 | 0.03929 | 9.795 | 0.01296 |
| pos_1041 | TG(14:0e/17:1/18:2) | down | 1.3511 | 0.746 | 1.16E-15 | 2.16E-14 | 6.102 | 0.04568 | 8.18 | 0.0381 |
| pos_1042 | TG(18:3/10:1/21:0) | down | 1.494 | 0.7002 | 7.83E-11 | 2.78E-10 | 5.97 | 0.2039 | 8.526 | 0.09155 |
| pos_1055 | TG(18:2/14:4/17:1) | up | 1.2165 | 1.233 | 1.78E-09 | 4.46E-09 | 9.011 | 0.1924 | 7.308 | 0.06971 |
| pos_1057 | TG(18:1/11:2/20:4) | up | 1.2051 | 1.2926 | 2.63E-05 | 3.49E-05 | 7.966 | 0.5728 | 6.163 | 0.1966 |
| pos_1061 | TG(18:3/11:3/20:2) | up | 1.2799 | 1.31 | 7.77E-09 | 1.70E-08 | 8 | 0.1081 | 6.107 | 0.2415 |
| pos_1066 | TG(20:3e/11:2/18:3) | up | 1.2554 | 1.2234 | 4.25E-15 | 6.85E-14 | 9.824 | 0.0236 | 8.03 | 0.05352 |
| pos_1067 | TG(20:4e/10:3/19:1) | up | 1.2954 | 1.242 | 3.89E-11 | 1.53E-10 | 9.85 | 0.1301 | 7.931 | 0.08675 |
| pos_1070 | TG(16:2e/11:3/22:4) | up | 1.3931 | 1.3127 | 3.87E-09 | 9.02E-09 | 9.394 | 0.233 | 7.156 | 0.1746 |
| pos_1074 | TG(15:0/17:0/18:1) | down | 2.5702 | 0.0833 | 7.12E-23 | 8.37E-20 | 0.6829 | 0.02342 | 8.197 | 0.03346 |
| pos_1077 | TG(16:0/16:1/18:1) | up | 1.2109 | 1.285 | 1.54E-08 | 3.17E-08 | 7.655 | 0.06163 | 5.957 | 0.2472 |
| pos_1079 | TG(16:0/16:0/18:3) | up | 1.5138 | 1.3759 | 6.27E-12 | 3.19E-11 | 9.576 | 0.01089 | 6.96 | 0.1771 |
| pos_1085 | TG(16:0/16:1/18:3) | up | 1.5121 | 1.4755 | 7.84E-11 | 2.78E-10 | 8.124 | 0.06925 | 5.506 | 0.2182 |
| pos_1087 | TG(18:1/14:0/18:3) | up | 1.2108 | 1.2322 | 1.90E-12 | 1.12E-11 | 8.877 | 0.01442 | 7.204 | 0.09951 |
| pos_1088 | TG(18:4/16:0/16:0) | up | 1.2188 | 1.2819 | 1.64E-10 | 5.33E-10 | 7.739 | 0.09719 | 6.037 | 0.1276 |
| pos_1092 | TG(16:0/17:0/18:1) | down | 2.456 | 0.0874 | 3.16E-20 | 5.30E-18 | 0.6571 | 0.02394 | 7.519 | 0.06429 |
| pos_1093 | TG(11:0/18:1/22:0) | up | 1.1554 | 1.2026 | 1.84E-11 | 7.99E-11 | 9.057 | 0.09418 | 7.531 | 0.06652 |
| pos_1094 | TG(18:3/11:1/22:6) | up | 1.4984 | 1.4256 | 3.23E-16 | 7.60E-15 | 8.562 | 0.02004 | 6.006 | 0.06115 |
| pos_1095 | TG(22:5/11:3/18:2) | up | 1.2612 | 1.301 | 3.97E-14 | 4.49E-13 | 7.832 | 0.04145 | 6.02 | 0.06115 |
| pos_1097 | TG(18:2/11:2/22:6) | up | 1.3941 | 1.3512 | 7.66E-07 | 1.20E-06 | 8.853 | 0.447 | 6.552 | 0.2672 |
| pos_1099 | TG(18:3e/11:1/22:6) | up | 1.4081 | 1.2959 | 7.47E-07 | 1.18E-06 | 10.28 | 0.02321 | 7.933 | 0.5297 |
| pos_1103 | TG(15:0/18:1/18:1) | down | 1.7994 | 0.622 | 8.07E-19 | 1.05E-16 | 6.061 | 0.03087 | 9.745 | 0.04051 |
| pos_1115 | TG(16:0/17:1/18:3) | down | 1.8643 | 0.5752 | 2.80E-11 | 1.15E-10 | 5.38 | 0.2517 | 9.353 | 0.1864 |
| pos_1116 | TG(18:3e/15:0/18:1) | down | 1.4849 | 0.7073 | 8.37E-12 | 4.05E-11 | 6.087 | 0.1754 | 8.606 | 0.01145 |
| pos_1118 | TG(16:2e/17:0/18:2) | down | 1.443 | 0.7148 | 2.08E-11 | 8.83E-11 | 5.967 | 0.03949 | 8.348 | 0.1778 |
| pos_1121 | TG(29:1/10:2/12:2) | down | 1.2309 | 0.8069 | 3.82E-11 | 1.52E-10 | 7.238 | 0.1394 | 8.97 | 0.0206 |
| pos_1122 | TG(15:0/18:2/18:3) | down | 1.3069 | 0.7937 | 6.43E-09 | 1.44E-08 | 7.59 | 0.08088 | 9.563 | 0.258 |
| pos_1129 | TG(18:4/15:0/18:2) | up | 1.101 | 1.2332 | 6.78E-06 | 9.51E-06 | 7.774 | 0.07481 | 6.304 | 0.4163 |
| pos_1132 | TG(18:3/11:1/22:3) | down | 1.1481 | 0.8301 | 8.84E-13 | 5.87E-12 | 7.342 | 0.0574 | 8.845 | 0.06091 |
| pos_1137 | TG(18:3/13:0/20:4) | down | 1.1927 | 0.8205 | 1.25E-12 | 7.68E-12 | 7.416 | 0.07196 | 9.038 | 0.05975 |
| pos_1138 | TG(18:3/10:3/23:1) | down | 1.2709 | 0.7783 | 1.24E-06 | 1.90E-06 | 6.746 | 0.1126 | 8.668 | 0.4444 |
| pos_1142 | TG(18:4/15:0/18:4) | up | 1.1117 | 1.2179 | 4.88E-06 | 6.94E-06 | 8.35 | 0.03127 | 6.856 | 0.4131 |
| pos_1147 | TG(18:3e/11:2/22:3) | up | 1.265 | 1.225 | 1.43E-10 | 4.70E-10 | 9.98 | 0.1588 | 8.147 | 0.06182 |
| pos_1148 | TG(18:3/11:1/22:5) | up | 1.3496 | 1.3327 | 1.58E-13 | 1.39E-12 | 8.316 | 0.07559 | 6.24 | 0.06115 |
| pos_1149 | TG(18:4/11:2/22:3) | up | 1.3508 | 1.3037 | 7.60E-11 | 2.72E-10 | 8.968 | 0.07425 | 6.879 | 0.1663 |
| pos_1157 | TG(12:1e/18:3/22:6) | down | 1.1927 | 0.8205 | 1.25E-12 | 7.68E-12 | 7.416 | 0.07196 | 9.038 | 0.05975 |
| pos_1166 | TG(16:1/18:1/18:2) | up | 1.7881 | 1.6165 | 5.37E-10 | 1.49E-09 | 9.626 | 0.02596 | 5.955 | 0.3895 |
| pos_1169 | TG(16:1/18:2/18:2) | up | 1.3357 | 1.2844 | 3.73E-10 | 1.09E-09 | 9.245 | 0.03721 | 7.198 | 0.2064 |
| pos_1170 | TG(6:0/22:3/24:2) | down | 1.4724 | 0.7121 | 1.09E-07 | 1.97E-07 | 6.269 | 0.2469 | 8.803 | 0.3955 |
| pos_1174 | TG(16:2e/16:0/20:3) | up | 2.5501 | 8.445 | 7.15E-21 | 2.10E-18 | 8.391 | 0.03223 | 0.9936 | 0.05501 |
| pos_1176 | TG(6:0/22:6/24:0) | down | 1.4789 | 0.7204 | 1.31E-08 | 2.73E-08 | 6.525 | 0.1596 | 9.058 | 0.3376 |
| pos_1177 | TG(16:0/18:3/18:3) | up | 1.4993 | 1.3644 | 6.32E-14 | 6.58E-13 | 9.586 | 0.01354 | 7.026 | 0.1086 |
| pos_1178 | TG(18:4/16:0/18:2) | down | 1.4489 | 0.7459 | 8.85E-12 | 4.20E-11 | 7.038 | 0.1424 | 9.436 | 0.08974 |
| pos_1179 | TG(16:1/18:2/18:3) | up | 1.2577 | 1.2758 | 6.79E-12 | 3.43E-11 | 8.355 | 0.03227 | 6.549 | 0.1191 |
| pos_1180 | TG(18:3e/16:0/18:3) | up | 1.2357 | 1.2727 | 3.17E-08 | 6.17E-08 | 8.274 | 0.1191 | 6.501 | 0.2608 |
| pos_1181 | TG(6:0/22:6/24:1) | down | 1.4256 | 0.7169 | 7.95E-10 | 2.14E-09 | 5.909 | 0.2382 | 8.243 | 0.1001 |
| pos_1183 | TG(18:4/16:0/18:3) | up | 1.4346 | 1.3933 | 1.99E-11 | 8.50E-11 | 8.335 | 0.009309 | 5.982 | 0.1789 |
| pos_1184 | TG(16:1/18:3/18:3) | down | 1.4197 | 0.7473 | 4.09E-10 | 1.18E-09 | 6.843 | 0.1494 | 9.157 | 0.1869 |
| pos_1192 | TG(20:0/15:0/18:1) | down | 1.293 | 0.7513 | 3.70E-13 | 2.86E-12 | 5.757 | 0.03087 | 7.663 | 0.0922 |
| pos_1195 | TG(18:4/13:0/22:6) | down | 1.1665 | 0.8264 | 4.89E-11 | 1.85E-10 | 7.407 | 0.09932 | 8.963 | 0.08361 |
| pos_1201 | TG(15:0/18:2/20:1) | down | 1.3321 | 0.7609 | 1.13E-08 | 2.40E-08 | 6.537 | 0.2938 | 8.591 | 0.05193 |
| pos_1210 | TG(17:0/18:2/18:3) | down | 2.3849 | 0.1893 | 6.26E-16 | 1.38E-14 | 1.511 | 0.0299 | 7.984 | 0.1716 |
| pos_1217 | TG(18:4/17:1/18:2) | up | 1.0304 | 1.2096 | 0.0002605 | 0.0003178 | 8.046 | 0.5252 | 6.652 | 0.3302 |
| pos_1219 | TG(18:4/17:1/18:3) | up | 1.2238 | 1.2877 | 2.34E-09 | 5.73E-09 | 7.72 | 0.2042 | 5.995 | 0.06115 |
| pos_1223 | TG(18:3/11:4/24:2) | up | 1.18 | 1.2461 | 2.08E-07 | 3.62E-07 | 8.273 | 0.1792 | 6.639 | 0.2673 |
| pos_1225 | TG(18:4/17:1/18:4) | down | 1.1274 | 0.8113 | 6.28E-07 | 1.01E-06 | 6.46 | 0.3253 | 7.963 | 0.07143 |
| pos_1232 | TG(18:4/18:3/18:3) | down | 1.2654 | 0.7411 | 4.57E-05 | 5.91E-05 | 5.751 | 0.3102 | 7.76 | 0.6503 |
| pos_1233 | TG(18:4/18:2/18:4) | down | 1.4459 | 0.7385 | 2.20E-14 | 2.69E-13 | 6.723 | 0.09062 | 9.104 | 0.01265 |
| pos_1235 | TG(18:4/18:3/18:4) | down | 1.1143 | 0.8302 | 4.72E-08 | 8.99E-08 | 7.058 | 0.07012 | 8.502 | 0.2331 |
| pos_1242 | TG(18:1/18:1/18:3) | up | 1.2448 | 1.2385 | 1.24E-13 | 1.15E-12 | 9.17 | 0.05267 | 7.404 | 0.06115 |
| pos_1249 | TG(18:3e/18:1/18:2) | up | 1.2296 | 1.227 | 2.74E-13 | 2.21E-12 | 9.312 | 0.0238 | 7.589 | 0.0819 |
| pos_1250 | TG(18:4/18:1/18:2) | up | 1.7203 | 1.5439 | 3.47E-10 | 1.02E-09 | 9.637 | 0.01712 | 6.242 | 0.3449 |
| pos_1254 | TG(18:3/18:2/18:3) | up | 1.3069 | 1.2782 | 7.50E-07 | 1.18E-06 | 9.305 | 0.387 | 7.28 | 0.2433 |
| pos_1255 | TG(18:4/18:2/18:2) | up | 1.3584 | 1.2829 | 1.36E-11 | 6.21E-11 | 9.56 | 0.01219 | 7.452 | 0.154 |
| pos_1258 | TG(18:3/18:3/18:3) | up | 1.5156 | 1.4652 | 1.57E-15 | 2.79E-14 | 8.236 | 0.04704 | 5.621 | 0.06115 |
| pos_1259 | TG(18:4/18:2/18:3) | up | 1.3586 | 1.3246 | 1.49E-11 | 6.64E-11 | 8.606 | 0.04389 | 6.497 | 0.1497 |
| pos_1261 | TG(18:3e/18:3/18:3) | down | 1.7468 | 0.5978 | 6.47E-17 | 2.17E-15 | 5.16 | 0.03087 | 8.632 | 0.06773 |
| pos_1266 | TG(22:4/11:1/22:5) | up | 1.2607 | 1.3377 | 5.03E-09 | 1.15E-08 | 7.273 | 0.2376 | 5.437 | 0.06115 |
| pos_1271 | TG(19:0/18:1/18:1) | down | 1.2149 | 0.7869 | 3.54E-13 | 2.76E-12 | 6.216 | 0.03087 | 7.899 | 0.07966 |
| pos_1273 | TG(11:0/22:1/22:1) | up | 1.1498 | 1.2226 | 1.39E-12 | 8.35E-12 | 8.282 | 0.07665 | 6.774 | 0.0429 |
| pos_1274 | TG(15:0/18:2/22:1) | down | 1.7069 | 0.4937 | 0.0009268 | 0.00109 | 3.906 | 2.114 | 7.911 | 0.08438 |
| pos_1277 | TG(16:2e/18:1/21:0) | down | 2.4589 | 0.1201 | 2.82E-17 | 1.33E-15 | 0.9394 | 0.02724 | 7.82 | 0.133 |
| pos_1278 | TG(19:0/18:2/18:2) | down | 1.445 | 0.7021 | 4.34E-12 | 2.27E-11 | 5.62 | 0.03087 | 8.004 | 0.1527 |
| pos_1279 | TG(19:0/18:1/18:3) | down | 2.2311 | 0.2807 | 1.25E-12 | 7.68E-12 | 2.216 | 0.03068 | 7.894 | 0.326 |
| pos_1281 | TG(15:0/18:3/22:1) | up | 1.2865 | 1.2907 | 4.34E-14 | 4.86E-13 | 8.37 | 0.02925 | 6.485 | 0.07183 |
| pos_1283 | TG(19:1/18:1/18:3) | down | 1.1103 | 0.812 | 1.55E-05 | 2.09E-05 | 6.549 | 0.4695 | 8.065 | 0.09467 |
| pos_1292 | TG(11:0/22:4/22:4) | up | 1.2496 | 1.3152 | 6.05E-07 | 9.74E-07 | 7.706 | 0.4029 | 5.859 | 0.06115 |
| pos_1301 | TG(18:3e/18:4/19:1) | up | 1.4618 | 1.4608 | 1.28E-14 | 1.69E-13 | 7.716 | 0.06406 | 5.282 | 0.06115 |
| pos_1304 | TG(22:4/11:1/22:4) | up | 1.2938 | 1.304 | 3.70E-06 | 5.34E-06 | 8.638 | 0.4414 | 6.624 | 0.3136 |
| pos_1318 | TG(20:1/18:1/18:1) | up | 1.4136 | 1.3823 | 2.44E-11 | 1.02E-10 | 8.259 | 0.02908 | 5.975 | 0.1752 |
| pos_1331 | TG(20:0/18:3/18:3) | up | 1.2737 | 1.2528 | 2.77E-07 | 4.72E-07 | 9.459 | 0.1161 | 7.55 | 0.3696 |
| pos_1335 | TG(20:1/18:3/18:3) | up | 1.0739 | 1.207 | 1.21E-05 | 1.65E-05 | 8.227 | 0.204 | 6.816 | 0.3824 |
| pos_1336 | TG(18:4/18:2/20:1) | up | 1.4659 | 1.4242 | 6.23E-07 | 1.00E-06 | 8.535 | 0.01426 | 5.993 | 0.5625 |
| pos_1352 | TG(16:1e/18:1/23:0) | down | 1.2286 | 0.7787 | 2.95E-10 | 8.84E-10 | 6.093 | 0.153 | 7.825 | 0.08124 |
| pos_1353 | TG(18:1/18:2/21:0) | down | 2.5064 | 0.055 | 5.25E-13 | 3.77E-12 | 0.4169 | 0.3035 | 7.579 | 0.226 |
| pos_1355 | TG(14:1e/19:1/24:1) | down | 2.6531 | 0.0316 | 2.93E-22 | 1.73E-19 | 0.2616 | 0.01378 | 8.269 | 0.04821 |
| pos_1356 | TG(16:2e/18:1/23:0) | down | 1.3261 | 0.7534 | 3.04E-16 | 7.29E-15 | 6.115 | 0.03087 | 8.117 | 0.03945 |
| pos_1358 | TG(19:1/18:2/20:1) | down | 2.484 | 0.1392 | 3.07E-20 | 5.30E-18 | 1.135 | 0.02856 | 8.155 | 0.06391 |
| pos_1361 | TG(19:0/18:1/20:4) | up | 1.515 | 1.4816 | 3.04E-08 | 5.92E-08 | 8.208 | 0.07613 | 5.54 | 0.4225 |
| pos_1366 | TG(18:3/18:3/21:1) | up | 1.3362 | 1.361 | 9.20E-07 | 1.44E-06 | 7.989 | 0.08325 | 5.87 | 0.4821 |
| pos_1371 | TG(18:4/18:3/21:1) | up | 1.3597 | 1.3509 | 6.59E-15 | 1.01E-13 | 8.104 | 0.03743 | 5.999 | 0.06115 |
| pos_1389 | TG(22:0/18:2/18:3) | up | 1.1472 | 1.2402 | 9.88E-10 | 2.60E-09 | 7.811 | 0.04759 | 6.298 | 0.1644 |
| pos_1397 | TG(18:3/18:3/22:3) | up | 1.4003 | 1.3028 | 2.43E-08 | 4.80E-08 | 9.787 | 0.02346 | 7.512 | 0.357 |
| pos_1398 | TG(25:0/16:0/18:1) | up | 1.138 | 1.2152 | 3.13E-10 | 9.30E-10 | 8.391 | 0.1362 | 6.905 | 0.0619 |
| pos_1401 | TG(25:0/16:0/18:2) | up | 1.1522 | 1.2206 | 2.25E-10 | 7.03E-10 | 8.421 | 0.1004 | 6.899 | 0.109 |
| pos_1404 | TG(18:1/18:2/23:1) | up | 1.1721 | 1.2569 | 6.97E-07 | 1.11E-06 | 7.965 | 0.03689 | 6.337 | 0.3627 |
| pos_1406 | TG(18:3e/18:1/23:0) | down | 1.6553 | 0.6332 | 8.44E-10 | 2.24E-09 | 5.436 | 0.3485 | 8.585 | 0.0385 |
| pos_1408 | TG(18:3e/18:2/23:0) | down | 1.5166 | 0.6868 | 2.90E-14 | 3.41E-13 | 5.744 | 0.09857 | 8.364 | 0.03159 |
| pos_1411 | TG(19:1/20:1/20:5) | up | 1.2248 | 1.2925 | 1.80E-14 | 2.32E-13 | 7.547 | 0.02005 | 5.839 | 0.06115 |
| pos_1413 | TG(29:1/12:4/18:3) | up | 1.5898 | 1.5196 | 2.28E-11 | 9.53E-11 | 8.446 | 0.05511 | 5.558 | 0.2161 |
| pos_1428 | TG(18:1/18:1/24:2) | down | 1.1432 | 0.8259 | 1.87E-10 | 6.03E-10 | 7.105 | 0.1373 | 8.603 | 0.04034 |
| pos_1430 | TG(24:0/18:2/18:3) | up | 1.2519 | 1.3104 | 5.55E-11 | 2.07E-10 | 7.573 | 0.09999 | 5.779 | 0.1138 |
| pos_1432 | TG(18:2e/18:2/24:1) | down | 1.4013 | 0.7188 | 5.64E-17 | 1.95E-15 | 5.711 | 0.03087 | 7.945 | 0.03577 |
| pos_1434 | TG(18:4/20:1/22:1) | up | 1.1121 | 1.2353 | 9.41E-07 | 1.46E-06 | 7.706 | 0.1342 | 6.238 | 0.3121 |
| pos_1435 | TG(24:1/18:2/18:3) | up | 1.1164 | 1.2074 | 1.55E-08 | 3.18E-08 | 8.405 | 0.01995 | 6.961 | 0.2159 |
| pos_1439 | TG(27:0/16:0/18:1) | up | 1.1083 | 1.215 | 2.78E-08 | 5.46E-08 | 8.058 | 0.2032 | 6.632 | 0.1021 |
| pos_1445 | TG(25:0/18:2/18:2) | up | 1.1004 | 1.201 | 8.95E-11 | 3.13E-10 | 8.282 | 0.05985 | 6.896 | 0.1073 |
| pos_1447 | TG(20:4e/18:1/23:0) | up | 1.1683 | 1.2652 | 1.33E-06 | 2.02E-06 | 7.762 | 0.04617 | 6.135 | 0.388 |
| pos_1448 | TG(18:1/21:0/22:6) | up | 1.1158 | 1.2243 | 6.53E-08 | 1.21E-07 | 7.925 | 0.1418 | 6.473 | 0.2095 |
| pos_1458 | TG(26:0/18:1/18:3) | up | 2.1784 | 5.0393 | 4.84E-05 | 6.24E-05 | 7.428 | 0.04877 | 1.474 | 2.15 |
| pos_1462 | TG(18:3/22:1/22:1) | up | 1.2443 | 1.246 | 8.33E-14 | 8.37E-13 | 8.935 | 0.026 | 7.171 | 0.07297 |
| pos_1464 | TG(18:4/22:1/22:1) | up | 2.2739 | 5.6076 | 1.08E-05 | 1.47E-05 | 7.688 | 0.07616 | 1.371 | 1.913 |
| pos_1466 | TG(18:0e/22:1/22:6) | up | 1.1164 | 1.2129 | 3.97E-10 | 1.15E-09 | 8.148 | 0.05267 | 6.718 | 0.1378 |
| pos_1467 | TG(27:0/18:0/18:1) | up | 1.0391 | 1.2333 | 5.79E-07 | 9.35E-07 | 6.745 | 0.188 | 5.469 | 0.2079 |
| pos_1471 | TG(27:0/18:2/18:2) | up | 1.1551 | 1.2494 | 2.21E-08 | 4.43E-08 | 7.751 | 0.1209 | 6.204 | 0.2085 |
| pos_1474 | TG(18:1/22:1/24:1) | up | 1.1891 | 1.2508 | 5.29E-10 | 1.47E-09 | 8.1 | 0.1357 | 6.476 | 0.1063 |
| pos_1476 | TG(24:1/18:2/22:1) | up | 1.3439 | 1.327 | 1.26E-10 | 4.23E-10 | 8.397 | 0.09695 | 6.328 | 0.1633 |
| pos_1479 | TG(24:1/18:3/22:1) | up | 1.5657 | 1.5244 | 1.78E-08 | 3.61E-08 | 8.262 | 0.05206 | 5.42 | 0.4296 |
| pos_1489 | TG(18:1/24:0/24:0) | up | 1.0843 | 1.2538 | 2.42E-06 | 3.55E-06 | 6.96 | 0.306 | 5.551 | 0.1922 |
| pos_1492 | TG(30:1/18:1/18:2) | up | 1.2374 | 1.3148 | 1.79E-08 | 3.62E-08 | 7.417 | 0.1523 | 5.641 | 0.2235 |
| pos_1493 | TG(30:0/18:2/18:2) | up | 1.1404 | 1.2602 | 2.35E-08 | 4.67E-08 | 7.314 | 0.1808 | 5.804 | 0.1525 |
| pos_1495 | TG(30:1/18:3/18:4) | down | 1.3984 | 0.7223 | 2.82E-08 | 5.52E-08 | 5.91 | 0.03087 | 8.182 | 0.3615 |
| pos_1509 | TG(26:0/18:1/24:0) | up | 1.9187 | 4.0078 | 0.0007702 | 0.0009139 | 6.701 | 0.3577 | 1.672 | 2.564 |
| pos_1510 | TG(26:0/18:1/24:1) | up | 1.0522 | 1.2391 | 5.31E-07 | 8.63E-07 | 6.779 | 0.2591 | 5.471 | 0.1179 |
| pos_1514 | TG(26:1/18:1/24:2) | up | 1.2262 | 1.3266 | 5.84E-08 | 1.09E-07 | 7.117 | 0.1982 | 5.365 | 0.2277 |
| pos_1520 | TG(29:1/18:3/22:3) | down | 1.272 | 0.7825 | 3.76E-13 | 2.88E-12 | 6.634 | 0.03087 | 8.478 | 0.08906 |
| pos_1523 | TG(29:1/18:2/22:6) | down | 1.2021 | 0.8228 | 2.76E-08 | 5.43E-08 | 7.791 | 0.2614 | 9.469 | 0.0566 |
| pos_1525 | TG(30:1/18:3/22:6) | down | 2.559 | 0.0764 | 8.12E-16 | 1.59E-14 | 0.6171 | 0.1758 | 8.072 | 0.1072 |
| pos_1528 | TG(28:0/18:1/24:2) | up | 1.0263 | 1.2504 | 2.99E-05 | 3.93E-05 | 6.542 | 0.4424 | 5.232 | 0.06115 |
| pos_1529 | TG(28:0/18:2/24:2) | up | 1.0673 | 1.2515 | 5.59E-07 | 9.05E-07 | 6.697 | 0.2498 | 5.351 | 0.156 |
| pos_1535 | TG(30:1/18:2/24:2) | up | 1.1246 | 1.2393 | 5.57E-09 | 1.27E-08 | 7.562 | 0.1461 | 6.102 | 0.1326 |
| pos_1538 | WE(3:0/18:1) | down | 1.2083 | 0.8197 | 5.60E-17 | 1.95E-15 | 7.553 | 0.02247 | 9.214 | 0.02697 |
| pos_1540 | WE(3:0/20:1) | down | 1.238 | 0.7931 | 2.89E-14 | 3.41E-13 | 6.692 | 0.04287 | 8.438 | 0.05401 |
| pos_1541 | WE(3:0/20:2) | down | 1.3007 | 0.8027 | 6.28E-16 | 1.38E-14 | 7.835 | 0.03637 | 9.761 | 0.03693 |
| pos_1542 | WE(3:0/20:3) | down | 1.3396 | 0.7882 | 8.96E-18 | 4.79E-16 | 7.603 | 0.02413 | 9.646 | 0.02661 |
| pos_1543 | WE(3:0/20:4) | down | 1.3469 | 0.7476 | 2.53E-11 | 1.05E-10 | 6.145 | 0.1563 | 8.22 | 0.04204 |
| pos_1544 | WE(3:0/22:2) | down | 1.3113 | 0.7607 | 9.98E-15 | 1.41E-13 | 6.223 | 0.04576 | 8.181 | 0.05234 |
| pos_1546 | ZyE(16:2) | down | 1.4201 | 0.7136 | 9.98E-11 | 3.43E-10 | 5.753 | 0.06037 | 8.062 | 0.198 |
| pos_1547 | ZyE(21:2) | down | 1.3513 | 0.7428 | 1.14E-13 | 1.08E-12 | 6.01 | 0.08671 | 8.091 | 0.03703 |
| pos_1549 | ZyE(22:2) | down | 1.3401 | 0.7445 | 2.23E-16 | 5.83E-15 | 5.956 | 0.03087 | 8 | 0.03882 |
| pos_1550 | ZyE(33:5) | up | 1.1762 | 1.2378 | 2.94E-09 | 6.99E-09 | 8.303 | 0.1228 | 6.708 | 0.16 |
| pos_1552 | ZyE(35:6) | up | 1.1754 | 1.2347 | 3.74E-13 | 2.88E-12 | 8.287 | 0.03044 | 6.712 | 0.07445 |
| pos_1553 | ZyE(36:4) | down | 1.1003 | 0.8122 | 1.39E-08 | 2.89E-08 | 6.064 | 0.2008 | 7.466 | 0.0545 |
| pos_1554 | ZyE(37:6) | up | 1.2206 | 1.2531 | 4.01E-08 | 7.68E-08 | 8.584 | 0.2518 | 6.85 | 0.1381 |
| pos_204 | DG(16:0/18:1) | down | 1.0693 | 0.7859 | 0.0001351 | 0.0001676 | 5.397 | 0.5666 | 6.867 | 0.2026 |
